# Unlocking the Nucleophilicity of Strong Alkyl C–H Bonds via Cu/Cr Catalysis

**DOI:** 10.1021/acscentsci.2c01389

**Published:** 2023-03-27

**Authors:** Pan Peng, Yifan Zhong, Cong Zhou, Yongsheng Tao, Dandan Li, Qingquan Lu

**Affiliations:** † The Institute for Advanced Studies (IAS), Wuhan University, Wuhan 430072, P. R. China; ‡ Key Laboratory of Micro-Nano Materials for Energy Storage and Conversion of Henan Province, Institute of Surface Micro and Nano Materials, College of Chemical and Materials Engineering, Xuchang University, Henan 461000, P. R. China

## Abstract

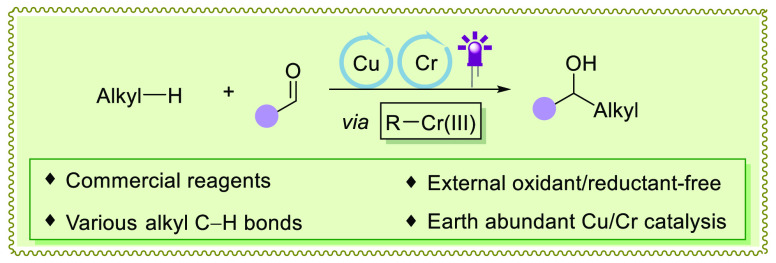

Direct functionalization of inert C–H bonds is one of the most attractive yet challenging strategies for constructing molecules in organic chemistry. Herein, we disclose an unprecedented and Earth abundant Cu/Cr catalytic system in which unreactive alkyl C–H bonds are transformed into nucleophilic alkyl–Cr(III) species at room temperature, enabling carbonyl addition reactions with strong alkyl C–H bonds. Various aryl alkyl alcohols are furnished under mild reaction conditions even on a gram scale. Moreover, this new radical-to-polar crossover approach is further applied to the 1,1-difunctionalization of aldehydes with alkanes and different nucleophiles. Mechanistic investigations reveal that the aldehyde not only acts as a reactant but also serves as a photosensitizer to recycle the Cu and Cr catalysts.

## Abstract 2

An unprecedented and Earth abundant Cu/Cr catalysis system was developed, enabling carbonyl addition reactions with strong alkyl C−H bonds.

## Introduction

**Figure 1:**
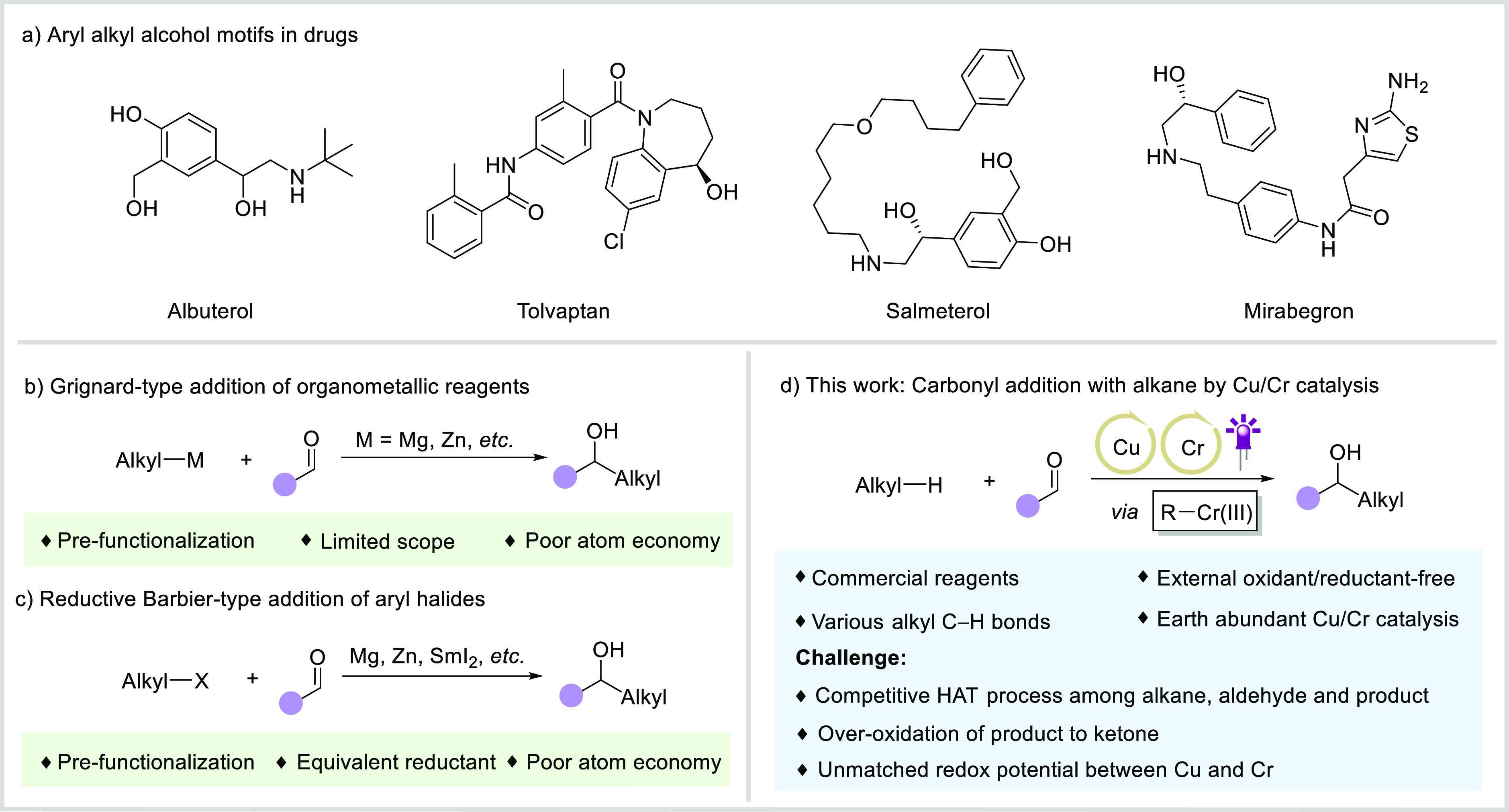
Application and synthesis of aryl alkyl alcohol motifs. a) Aryl alkyl alcohol motifs in drugs. b) Grignard-type addition of organometallic reagents. c) Reductive Barbier-type addition of aryl halides. d) This work: carbonyl addition with alkanes.

Alcohols are valuable and versatile building blocks and have wide applications in bulk/fine chemicals, agrochemicals, pharmaceuticals, and natural product synthesis (Figure 1a).^1^ Consequently, seeking efficient methodologies to access versatile alcohols has been a long-standing interest of the synthetic community. In this context, carbonyl addition reactions represent one of the most well-established methods for alcohol synthesis. Among them, Grignard-type additions of organometallic reagents (e.g., Mg, Zn)^2^ and Barbier-type additions of organic halides^3,4^ have been well-developed for many applications in organic synthesis (Figure 1b,c). However, organometallic reagents and organic halides are often prepared from the corresponding hydrocarbons, inevitably leading to labor-intensive multistep operations, unexpected side products, and poor atom and step economy. From the perspective of sustainability, direct nucleophilic addition of alkyl C–H bonds to carbonyls provides an appealing yet challenging pathway.

Research into the selective conversion of abundant alkanes into higher-value, functionalized chemical feedstocks is highly important.^5−8^ However, the strong bond-dissociation energy of the nonpolar alkyl C–H bonds in alkanes brings huge challenges in chemoselective control, where oxidative degradation of other functionalities and solvent functionalization are likely to occur prior to the desired C(sp^3^)–H functionalization. As a result, even though great progress has been made in C(sp^3^)–H functionalization,^9−16^ including transition-metal-catalyzed C–H activation,^17−23^ carbene/nitrene/oxene-induced C–H insertion,^24−26^ and radical process triggered by hydrogen atom transfer or oxidation,^27−35^ a catalytic and general method for the coupling of strong alkyl C–H bonds with carbonyls remains elusive. Recently, merging chromium catalysis and photocatalysis has begun to flourish for diverse transformations,^36−42^ in which a carbon radical is quickly intercepted by chromium(II) (*k* = 10^7^–10^8^ M^–1^·s^–1^)^43^ and further transforms into a nucleophilic organochromium(III) species. Through activating allylic C–H bonds by oxidation/deprotonation or hydrogen-atom transfer, the Glorius and Kanai group independently developed an innovative allylation of aldehydes with alkenes by the combination of photocatalysis and chromium catalysis.^41,44−46^ Mechanistically, the substrate scope depends on the oxidation potential of the excited *Ir^III^ photocatalyst (∼1.21 V vs SCE) or the BDEs of thiophosphoric imide (∼87 kcal/mol, HAT catalyst). As a result, in all cases, the substrates are limited to relatively reactive and weak allylic C(sp^3^)–H bonds (∼85 kcal/mol). Thus, the development of a catalytic and general method for the application of strong alkyl C–H bonds with high oxidative potential (often above 3.0 V vs SCE) and high bond-dissociation energy (∼96–101 kcal/mol) into carbonyl addition reactions is highly desirable.

On the other hand, chemoselective control is a big challenge in nucleophilic addition of strong alkyl C–H bonds to aldehydes. First, because alkyl C–H bonds often have higher bond-dissociation energy than aldehyde C–H bonds (e.g., 96.2 kcal/mol for cyclohexane vs 88.9 kcal/mol for benzaldehyde),^47^ aldehyde C–H bonds might be activated preferentially. Second, the desired products, aryl alkyl alcohols, have much lower oxidation potential than aldehydes and alkanes, and cannot survive from a strong oxidizing photoredox catalyst. Furthermore, aryl alkyl alcohols contain relatively weak and reactive benzylic C–H bonds (∼85.1 kcal/mol)^47^ as well as multiple alkyl C–H bonds; therefore, multifunctionalization is an issue which can result in poor reaction selectivity. On the basis of the above analysis, we proposed a multimetallic catalysis strategy to achieve the desired carbonyl addition reactions with alkyl C–H bonds. In this strategy, the visible-light-mediated ligand-to-metal charge transfer (LMCT, e.g., CuCl_2_) pathway generates a highly electrophilic chlorine radical to activate strong alkyl C–H bonds, and a chromium catalyst transforms the carbon radical to nucleophilic organochromium(III) species. Meanwhile, transition metal salts can serve as Lewis acids to deactivate aldehydes and aryl alkyl alcohols through coordinating with oxygen, thus ensuring reaction selectivity.

## Results and Discussion

**Figure 2:**
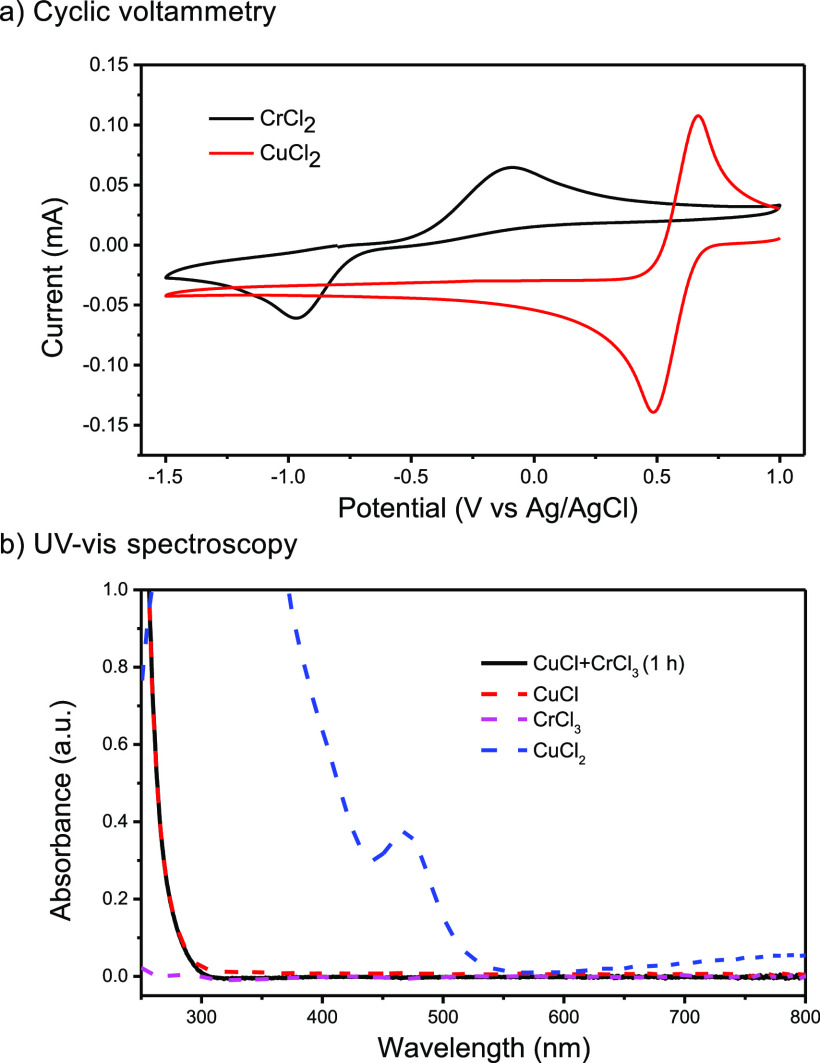
Initial mechanistic studies. a) Cyclic voltammetry. b) UV–vis spectroscopy.

To achieve the above-mentioned goal, the first challenge arises from how to merge oxidative catalysis and reductive catalysis into one system without the assistance of an additional photosensitizer. Initially, cyclic voltammetry experiments were performed to test whether the two catalysts (Cu and Cr) could realize a closed catalytic cycle with each other. As shown in Figure 2a, cyclic voltammetry experiments showed that the reduction of CuCl_2_ (*E*_p/2_ = 0.58 V in DMF) occurred at the more positive potential compared with CrCl_3_ (*E*_p/2_ = −0.84 V in DMF), illustrating that oxidation of Cu(I) by Cr(III) was thermodynamically unfavorable. Subsequently, the reaction between CuCl and CrCl_3_ was studied by UV–vis spectroscopy (Figure 2b). When CrCl_3_ was added to a CuCl solution and the mixture was stirred for 1 h, the UV–vis spectrum of the reaction mixture was almost the same as that of CuCl, and the signal of CuCl_2_ was not observed, thus implying that the redox reaction between CuCl and CrCl_3_ is unfavorable. To recycle both Cu/Cr catalysts and overcome the unfavorable electron transfer process caused by the unmatched redox potential between Cu/Cr catalysts, an extra photosensitizer could act as an electron shuttle. Instead of introducing an expensive photosensitizer, the aldehyde itself can serve as a photosensitizer because a carbonyl group is able to be excited to a relatively long-lived triplet state upon absorption of a photon.^48^ Further luminescence quenching experiments clearly showed that the emission intensity of aldehyde in the presence of CuCl was decreasing (Supporting Information, Figure S5), indicating that the excited aldehyde was reductively quenched by CuCl to recycle Cu(II). The quenching process afforded the corresponding α-OH or α-OCu(II) substituent benzylic radical. This benzyl radical has a low oxidative potential (for example, the oxidative potential for α-methoxybenzyl is −0.33 V vs SCE)^49^ and can be oxidized by Cr(III) to recycle Cr(II).

With the proof of the proposed multimetallic catalysis strategy, the carbonyl addition reaction between cyclooctane **1** and *p*-fluorobenzaldehyde **2** was next investigated. Delightfully, the desired product **3** was obtained in 41% yield using CuCl_2_ as the LMCT catalyst in the presence of CrCl_2_ under the irradiation of purple light (λ_max_ = 390 nm) in CH_3_CN (Table 1, entry 1). Other LMCT catalysts such as FeCl_3_ and CeCl_3_ were proven to be less effective (Table 1, entries 2–3).^50^ Under the conditions of entry 1, the separation of the two phases was observed, caused by the insolubility of cyclooctane in CH_3_CN, thus presumably hampering effective hydrogen atom transfer between the photogenerated chloride radical and the alkane. The mixed solvent (CH_3_CN/DCE = 1/1) was capable of enabling formation of a homogeneous reaction solution, and an increased yield of 61% was obtained with 89% conversion of *p*-fluorobenzaldehyde (entry 4). The bad reaction selectivity was partly caused by the chlorination of aldehyde and overoxidation of the generated benzylic alcohol. In addition, further investigation demonstrated that benzylic alcohols could suppress the desirable reaction (Supporting Information, Figure S16). Given that a Lewis acid could deactivate aldehydes and alcohols through coordination with oxygen,^51^ several Lewis acids were next evaluated to suppress competitive side reactions (entries 5–7). Delightfully, the yield was increased to 74% (entry 5) when Me(OH)_2_ was employed.^52^ Lowering the number of alkane equivalents to 5 also gave a synthetically useful yield, and bench-stable CrCl_3_ could also be used as a catalyst (entries 8–9). Control experiments indicated that CuCl_2_, CrCl_2_, and the light source were all essential for optimal efficiency (entries 10–13); no or trace product was formed in the absence of light or under blue light irradiation.

**Table 1:**
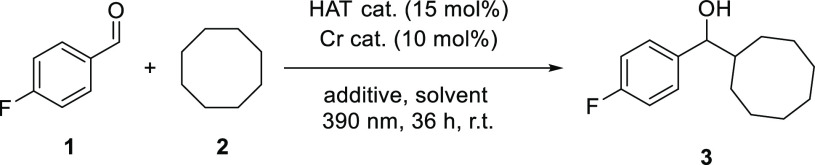
Optimization of the Reaction Conditions^a^

entry	HAT cat.	Cr cat.	additive	yield (%)^b^
1	CuCl_2_	CrCl_2_	-	41
2	CeCl_3_	CrCl_2_	-	18
3	FeCl_3_	CrCl_2_	-	19
4^c^	CuCl_2_	CrCl_2_	-	61
5^c^	CuCl_2_	CrCl_2_	Zn(OTf)_2_	4
6^c^	CuCl_2_	CrCl_2_	Mg(ClO_4_)_2_	44
7^c^	CuCl_2_	CrCl_2_	MeB(OH)_2_	74(73)
8^c^^,^^d^	CuCl_2_	CrCl_2_	MeB(OH)_2_	61
9^c^^,^^e^	CuCl_2_	CrCl_3_	MeB(OH)_2_	65
10^c^	-	CrCl_2_	MeB(OH)_2_	24
11^c^	CuCl_2_	-	MeB(OH)_2_	21
12^c^^,^^f^	CuCl_2_	CrCl_2_	MeB(OH)_2_	trace
13^c^^,^^g^	CuCl_2_	CrCl_2_	MeB(OH)_2_	n.d.

a Reaction conditions: aldehyde (0.3 mmol), alkane (3.0 mmol, 10 equiv), additive (0.3 mmol, 1.0 equiv), HAT catalyst (15 mol %), Cr catalyst (10 mol %), CH_3_CN (2.0 mL), purple light (λ_max_ = 390 nm).

b Yields were determined by ^19^F NMR analysis with 1-fluoronaphthalene as an internal standard; isolated yield is reported in parentheses.

c CH_3_CN/DCE (v/v = 1/1, 2.0 mL).

d Alkane (1.5 mmol, 5.0 equiv).

e CrCl_3_ (15 mol %).

f Blue light irradiation (λ_max_ = 452 nm).

g No light. n.d. = not detected.

**Figure 3:**
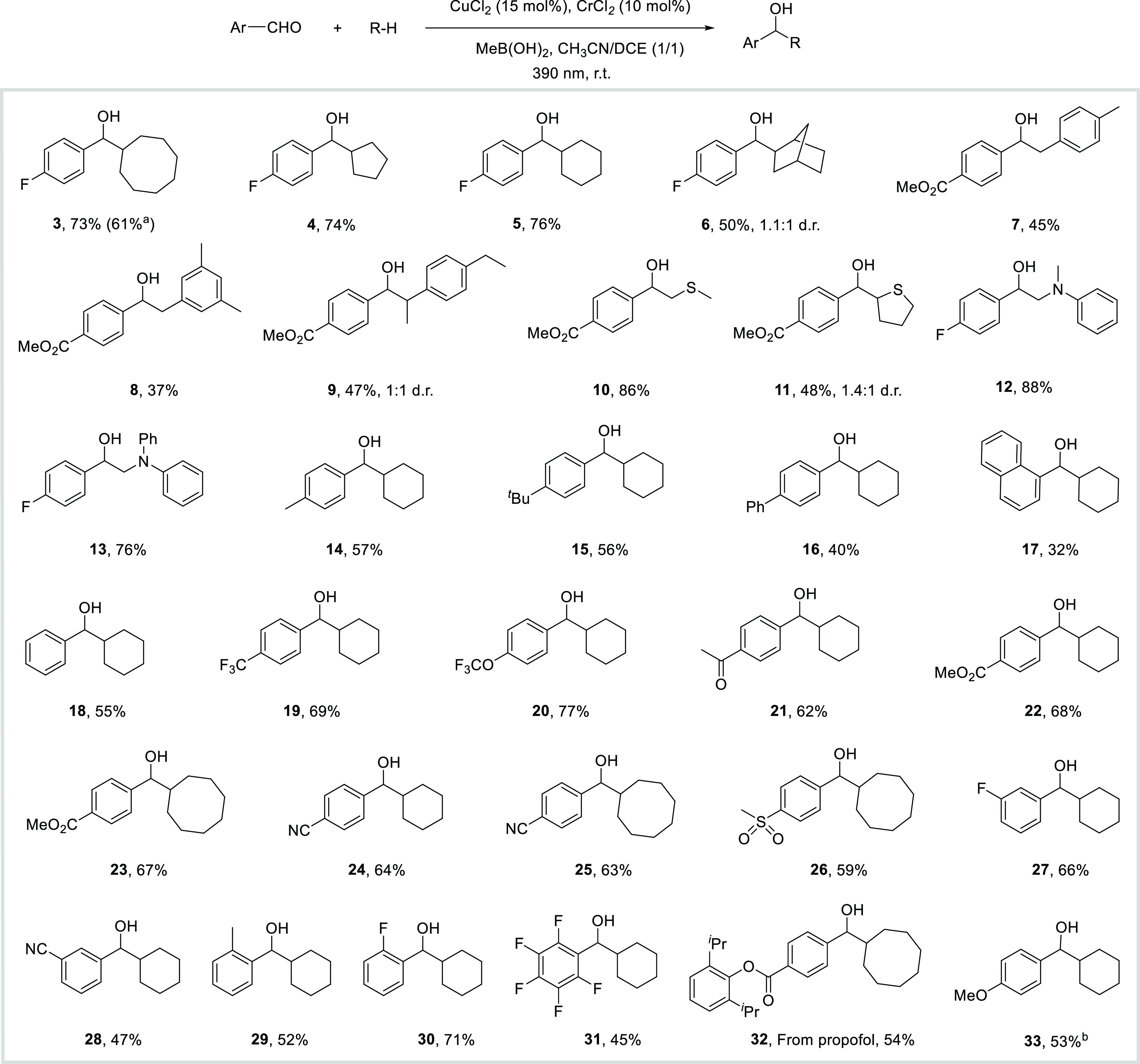
Substrate scope. Reaction conditions: aldehyde (0.3 mmol), alkane (5–30 equiv), MeB(OH)_2_ (1.0 equiv), CuCl_2_ (15 mol %), CrCl_2_ (10 mol %), CH_3_CN (1.0 mL), DCE (1.0 mL), 390 nm light irradiation, 36–96 h, for details, please see the Supporting Information. ^a^Alkane (5 equiv). ^b^Adding TESCl (2.0 equiv) instead of MeB(OH)_2_.

With the optimized conditions in hand, we sought to explore the scope of this transformation (Figure 3). First, unactivated alkyl C–H compounds were investigated. Cyclic alkanes such as cyclooctane, cyclopentane, and cyclohexane were proven to be effective coupling partners (**3**–**5**). Notably, norbornane gave the benzylic alcohol **6** with high regioselectivity. Then, π-system and heteroatom activated C–H bonds were investigated. Benzylic 1° and 2° C–H bonds were compatible in this catalytic carbonyl addition (**7**–**9**). Functionalization was observed at the benzylic position over the methyl position when 1,4-diethylbenzene was used (**9**). Heteroatom-adjacent (sulfur and nitrogen) C–H bonds were also amenable to this transformation (**10**–**13**). With tetrahydrothiophene, the functionalization selectively occurred at the weaker α-C–H bond over the β position (**11**).

Next, we explored the alkylation of various aldehydes under the catalytic conditions. As shown in Figure 3, aryl aldehydes with electron-donating groups and electron-withdrawing groups were well-tolerated. *para*-Methyl or *tert*-butyl substituted benzylaldehydes were suitable substrates with methyl and *tert*-butyl C–H bonds retained (**14**, **15**). *p*-Biphenylaldehyde, 1-naphthaldehyde, and benzaldehyde were amenable to this alkylation reaction (**16***–***18**). Trifluoromethyl and trifluoromethoxy groups, which are important motifs in drug molecules, were tolerated in good yields (**19**, **20**). Functional groups that are typically sensitive to Grignard reagents, such as carbonyls, esters, and cyano groups, also reacted efficiently (**21***–***25**). A strong electron-withdrawing group (mesyl) was a competent substrate (**26**). The reaction gave comparable yields when using *meta*- or *ortho*-substituted benzaldehydes (**27***–***30**). An *ortho*-methyl substituted aryl aldehyde delivered the addition product in 52% yield, implying that steric hindrance had little influence on this reaction (**29**). Polyfluoroarene benzylic alcohol **31** was obtained in 45% yield. Finally, the modification of a drug derivative was conducted to demonstrate the practicability of this transformation. Aldehydes containing a propofol motif delivered alkylation product **32** in a synthetically useful yield. When an electron-rich aryl aldehyde, *p*-anisaldehyde, was applied to this reaction, a low yield was obtained because the electron-rich benzylic alcohol underwent dehydroxylation via a benzylic cation intermediate under Lewis acid catalysis. The yield could increase to 53% by adding TESCl instead of MeB(OH)_2_ (**33**). Aliphatic aldehydes were not well tolerated in this transformation, possibly due to their weak eletrophilicity and ineffective excitation by the purple light employed in the reaction (see Supporting Information, Figure S17).

**Figure 4:**
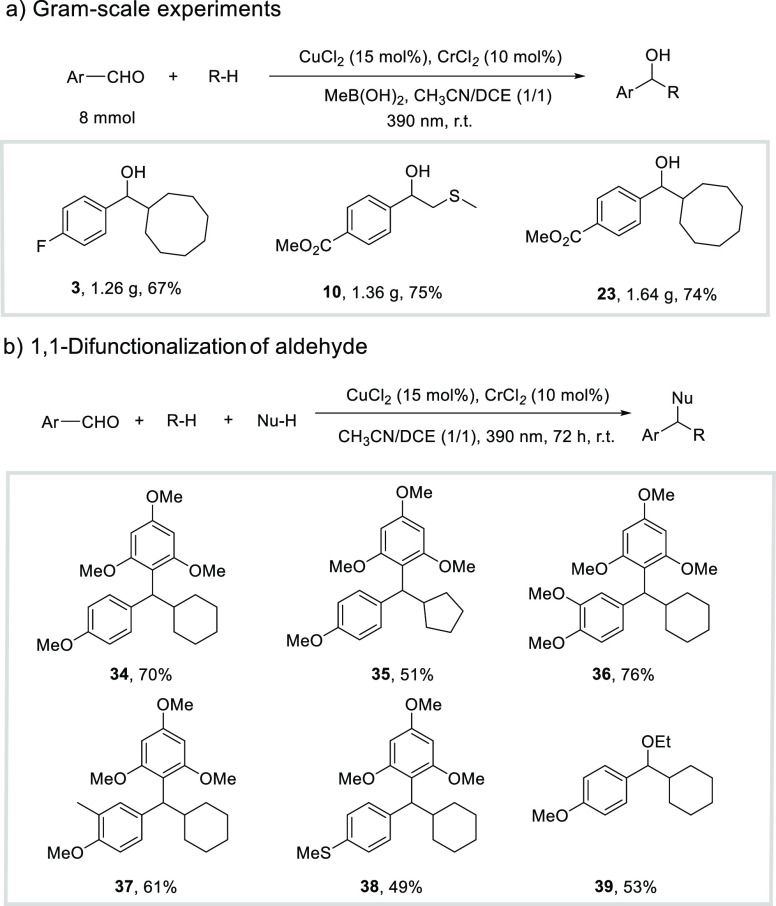
Applications. a) Gram-scale reactions. b) 1,1-Difunctionalization of aldehyde.

A common limitation of photoredox reactions is their scalability because of issues of light penetration.^53^ In contrast, this reaction could be readily scaled up to gram quantities with a similar efficiency. For example, the synthesis of **3**, **10**, and **23** was performed on a 8 mmol scale, delivering benzylic alcohols in 67, 75, and 74% yields, respectively (Figure 4a).

As electron-rich benzylic alcohol could undergo nucleophilic substitution via carbocations following the S_N_1 mechanism under Lewis acid catalysis, we further expanded the reaction to a tandem alkane addition and subsequent nucleophilic substitution processes, realizing deoxygenative 1,1-difunctionalization of aldehydes (Figure 4b, see Supporting Information, Figure S18). Irradiation of *p*-anisaldehyde, 1,3,5-trimethoxybenzene, and cyclohexane under CuCl_2_ and CrCl_2_ catalysts gave a 70% yield of the deoxygenative 1,1-dicarbofunctionalization product (**34**). Cyclopentane delivered the 1,1-dicarbofunctionalization product in a 51% yield (**35**). 3,4-Disubstituted aryl aldehydes were transformed into the desired products in 61–76% yields (**36**, **37**). Of note, 4-(methylthio)benzaldehyde was also a compatible reaction substrate, affording the corresponding product (**38**) with the methylthio group retained. Alcohols, as exemplified by ethanol, could also serve as suitable nucleophiles in this protocol, providing the deoxygenative 1,1-oxycarbonylation product in 53% yields (**39**). It is noteworthy that deoxygenative 1,1-difunctionalization of aldehydes via a tandem multicomponent reaction in one-pot is very challenging, because necleophiles can directly react with aldehydes (bimolecular reaction, for example, an acetal byproduct was detected when ethanol was used as a nucleophile) and/or deactivate metal catalysts through undesired coordination.

**Figure 5:**
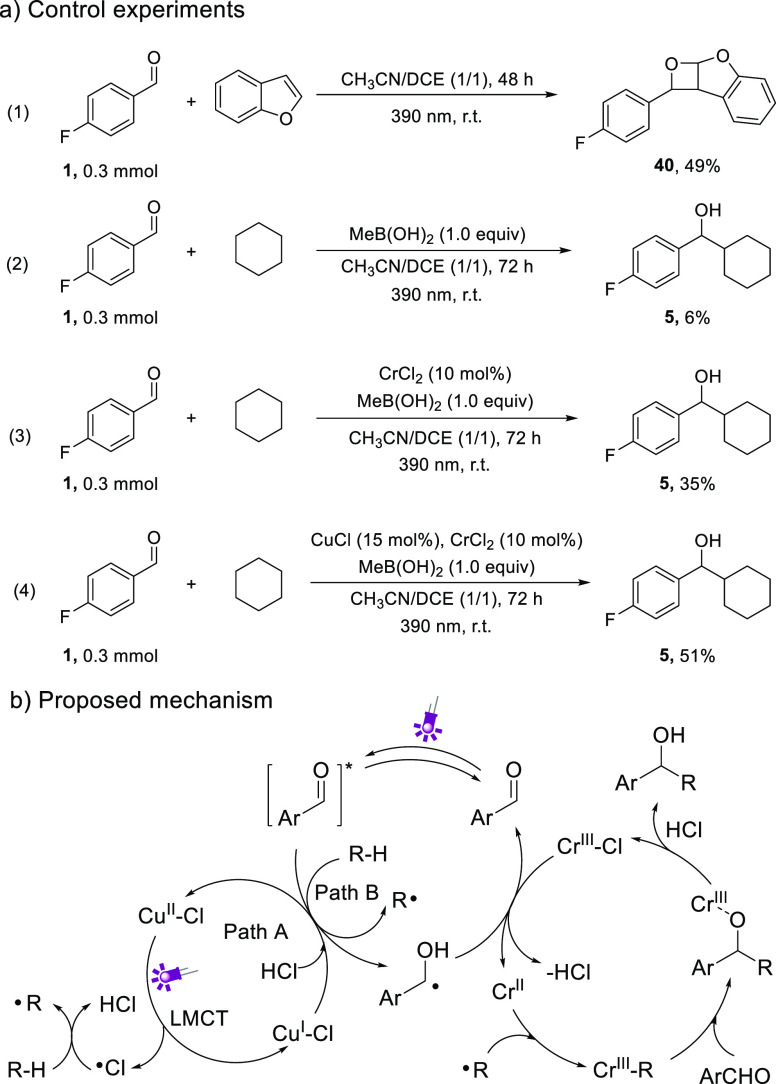
Mechanism. a) Control experiments. b) Proposed mechanism.

To gain more insight into the reaction mechanism, a series of experiments were conducted. UV–vis spectroscopy revealed that aldehyde **1** had an absorption in the wavelength range of the light source applied in this protocol, and a red shift was clearly observed in the presence of CuCl or CrCl_2_ (see Supporting Information, Figures S10–S15). When aldehyde **1** was irradiated in the presence of 2,3-benzofuran under purple light, the well-known Patern-Büchi reaction product **40** was obtained in 49% yield (Figure 5a, eq 1).^54^ Moreover, the irradiation of aldehyde **1** with alkane **2** under metal-free conditions also gave alcohol **5** in 6% yield (Figure 5a, eq 2). Alcohol **5** was probably generated through HAT of the alkane by excited aldehyde **1** and radical–radical coupling between the alkyl radical and the ketyl radical (Supporting Information, Figure S19). These results illustrated the existence of an excited aldehyde intermediate. Furthermore, the reaction between **1** and cyclohexane in the presence of CrCl_2_ led to an increased yield to 35%, illustrating the possibility of a carbonyl-photoredox/chromium dual catalysis cycle (Figure 5a, eqs 2–3). In addition, using CuCl instead of CuCl_2_ gave a 51% yield, revealing that Cu(I) might also be an active catalytic species in this protocol (Figure 5a, eq 4). Finally, an obvious interaction between benzyl alcohols **3** and MeB(OH)_2_ was observed by ^19^F NMR and ^11^B NMR (Supporting Information, Figures S6 and S7), implying that the coordination of MeB(OH)_2_ with alcohol played an important role in protecting alcohol products from overoxidation.

A detailed description of the proposed pathway for aliphatic C–H bond addition to carbonyls is outlined in Figure 5b. The process begins with electrophilic chloride radical formation via ligand-to-metal charge transfer of the excited CuCl_2_ catalyst.^55^ This chlorine radical will undergo HAT with alkane to form a reactive alkyl radical.^56,57^ The Cr(II) quickly intercepts the alkyl radical to form a stable organometallic alkyl–Cr(III). The generated alkyl–Cr(III) attacks the aldehyde via polar addition to form Cr(III)-alcoholate, which then undergoes protonation to afford benzylic alcohol. MeB(OH)_2_ can stabilize the benzylic alcohol from overoxidation. According to our mechanistic research, catalytic turnover through a direct redox reaction between Cu(I) and Cr(III) seems unfavorable. The excited aldehyde would serve as a photosensitizer and realize the catalytic turnover. That is, the excited aldehyde oxidizes the Cu(I) to Cu(II) and, in turn, is converted to a ketyl radical (Path A). The ketyl radical would then reduce Cr(III) to Cr(II) and convert back to the original aldehyde. Alternatively, the excited aldehyde can abstract a hydrogen atom from the alkyl C–H bond; thus, the aldehyde and chromium dual catalytic cycles are also able to realize this transformation (Path B).

## Conclusion

In summary, an Earth abundant Cu/Cr catalysis system is developed for the first time in which strong alkyl C–H bonds are converted to nucleophilic alkyl–Cr(III) through radical-to-polar crossover. Diverse inert alkyl C–H compounds, including feedstock alkanes, are smoothly added to carbonyls at room temperature under purple light irradiation. Various aryl alkyl alcohols are furnished under mild and simple reaction conditions, even on a gram scale. Mechanistic investigations reveal that the aldehyde not only acts as a reactant but also serves as a photosensitizer to recycle Cu and Cr catalysts, thus avoiding the use of expensive photosensitizers.

## Supporting Information Available

Experimental procedure, characterization data, and copies of ^1^H and ^13^C NMR spectra (PDF)
