# Supplementary material for: Unlocking the Nucleophilicity of Strong Alkyl C–H Bonds via Cu/Cr Catalysis
Source: ACS Cent Sci. 2023 Mar 27;9(4):756–62. doi: 10.1021/acscentsci.2c01389 (PMC10141608; doi:10.1021/acscentsci.2c01389)
Supplement: Supplementary file 1 — oc2c01389_si_001.pdf [file oc2c01389_si_001.pdf]

Supporting Information

Unlocking the Nucleophilicity of Strong Alkyl C–H Bonds via Cu/Cr Catalysis

Pan Peng[1], Yifan Zhong[1], Cong Zhou[1], Yongsheng Tao[1], Dandan Li[2] and Qingquan Lu[1,*]

[1]The Institute for Advanced Studies (IAS), Wuhan University, Wuhan 430072, P. R. China
[2]Key Laboratory of Micro-Nano Materials for Energy Storage and Conversion of Henan Province, Institute of Surface Micro and Nano Materials, College of Chemical and Materials Engineering, Xuchang University, Henan 461000, P. R. China.

*Corresponding author: Qingquan Lu, Email: gci2011@whu.edu.cn

This PDF file includes:

General information

Experimental section

Figures. S1 to S20

References (1 to 11)

NMR spectra of compounds

Table of Contents

1. General information S3
2. Experimental section S4
   2.1 Procedure for alkane addition to aldehydes S4
   2.2 Procedure for 1,1-difunctionalization of aldehydes S5
   2.3 Procedure of the gram scale experiments S6
   2.4 Luminescence spectroscopy S6
   2.5 Studies of interaction between alcohol **3** with $MeB(OH)_2$ by NMR experiments S7
   2.6 Cyclic voltammetry experiments S8
   2.7 UV-visible absorption spectra S9
   2.8 Control experiments S12
   2.9 The reactivity of aliphatic aldehydes S16
   2.10 Control experiments and proposed mechanism for 1,1-difunctionalization of aldehyde S16
   2.11 Detection of alkyl radical or benzyl radical S17
   2.12 Analytical data of compounds S19
3. References S39
4. NMR spectra of products S40

1. General information

All reactions were performed using PTFE-coated magnetic stirring bars in oven-dried glassware under argon, unless otherwise stated. Materials were obtained from commercial suppliers (Bidepharm, Innochem, Energy Chemical, etc.) and used without further purification. Anhydrous solvents were purchased from Energy Chemical and stored over molecular sieves. Thin layer chromatography (TLC) employed glass 0.25 mm silica gel plates. Flash chromatography columns were packed with 200-300 mesh silica gel. Gas chromatography (GC) was recorded on an Agilent 8890 Series spectrometer. GC-MS was recorded by an Agilent 8890 GC and an Agilent 5977B MSD Series spectrometer. All new compounds were characterized by ^{1}H NMR, ^{13}C NMR, ^{19}F NMR and HRMS. The known compounds were characterized by ^{1}H NMR, ^{13}C NMR and ^{19}F NMR. ^{1}H and ^{13}C NMR spectra were recorded on a Bruker AVANCE NEO 600 or JEOL 400 spectrometers operating at 600 MHz or 400MHz for proton, 151 MHz or 101 MHz for carbon nuclei and were calibrated using residual undeuterated solvent as an internal reference ($CDCl_3$: 7.26 ppm for ^{1}H NMR and 77.16 ppm for ^{13}C NMR). Multiplicity was recorded as follows: s = singlet, brs = broad singlet, d = doublet, t = triplet, q = quartet, quint = quintet, sex = sextet, sept = septet, and m = multiplet. All chemical shifts (δ) were reported in ppm and coupling constants (*J*) in Hz. EPR data were recorded on Bruker EMXmicro-6/1 x band. High resolution mass spectra (HRMS) were measured with Thermo Scientific Q Exactive Focus Orbitrap LC-MS/MS or Thermo Orbitrap Elite, accurate masses are reported for the M^+, molecular ion + proton ($[M+H]^+$) or molecular ion + Na^+ ($[M+Na]^+$).

Home-made light source

The home-made light source consists of an integrated led lamp bead (purple light, 20 W, λ_{max} = 390 nm), a fan and a constant current LED drive power supply.

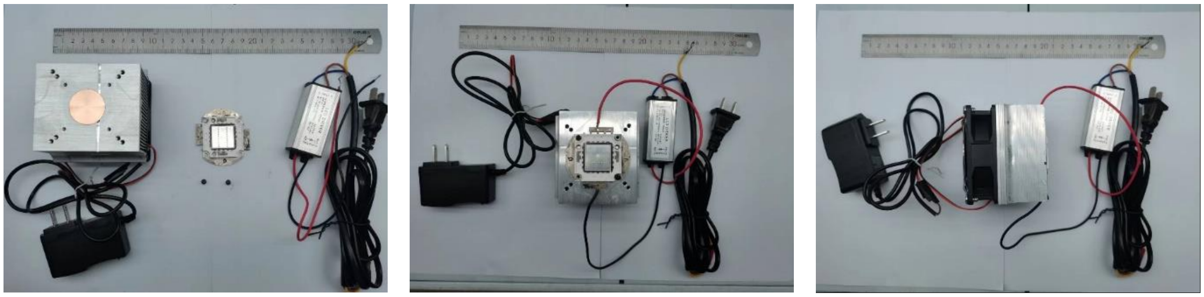

Figure S1. The home-made light source

The emission spectrum of integrated led lamp bead

The emission spectrum of the integrated led lamp bead (20 W, λ_{max} = 390 nm) was measured using Photo-Catalysis Spectrometer (WATTCAS^{TW}). The Photo-Catalysis Spectrometer was placed vertically directly above the light source, fan was enforced for effective thermal management to maintain luminous efficiency and life expectancy of 20 W LED light.

Table 1. Irradiance in relation to distance

Distance (cm)	1.0 cm
Irradiance (mW/cm^2)	523 mW/cm^2

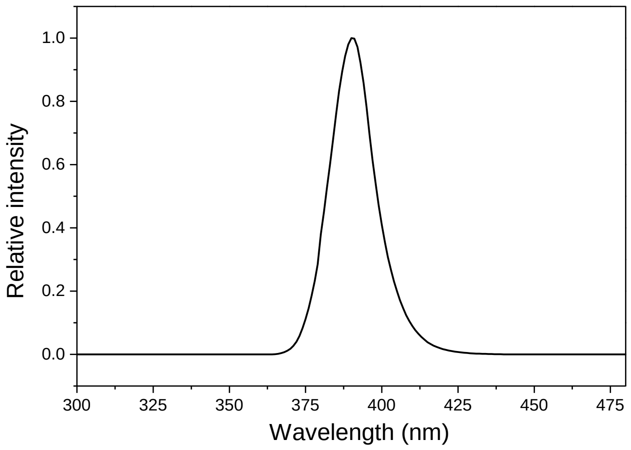

Figure S2. Measured emission spectrum of the integrated led lamp bead

2. Experimental section

2.1 Procedure for alkane addition to aldehydes

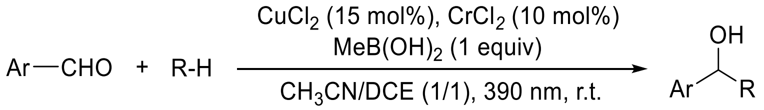

General procedure: In glove box, to an oven-dried 10 mL Schlenk tube equipped with a Teflon-coated magnetic stirring bar were added aldehyde (if solid, 0.3 mmol, 1.0 equiv), $MeB(OH)_2$ (0.3 mmol, 18.0 mg, 1.0 equiv) , $CuCl_2$ (0.045 mmol, 6.1 mg, 15 mol%), $CrCl_2$ (0.03 mmol, 3.7 mg, 10 mol%), CH_3CN (1.0 mL) and DCE (0.5 mL). Then take out of glove box, add aldehyde (if liquid), C-H compound (10-30 equiv) and DCE (0.5 mL) under positive argon pressure. The reaction mixture was stirred under purple light irradiation (20 W, λ_{max} = 390 nm) at room temperature (around 25 ^oC) for 36-96 hours. Then, the solvent was removed *in vacuo* and

corresponding product was purified by flash chromatography on silica gel. In each case, the light source was placed ~ 1 cm from the reaction vessel (the light intensity is about 523 mW/cm^2). The reaction temperature was measured not to exceed room temperature more than 10 °C. Light intensity has an impact for the reaction efficiency. For example, the reaction rate of standard reaction becomes slow when a light intensity of 365 mW/cm^2 is applied to replace the standard 523 mW/cm^2, and a longer reaction time (from 36 hours to 48 hours) is required to get a comparable yield of the standard reaction.

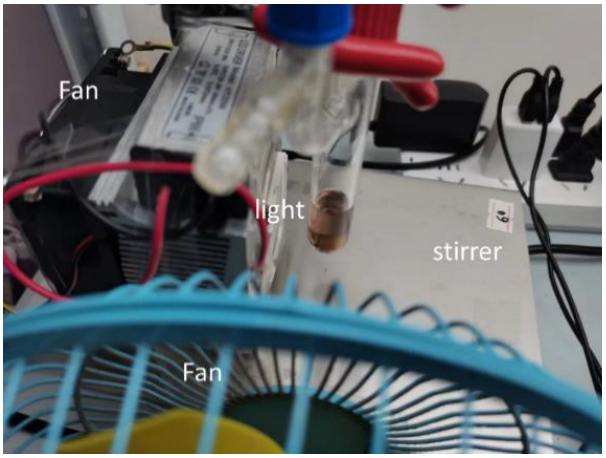

Figure S3. Photograph of the reaction apparatus

2.2 Procedure for 1,1-difunctionalization of aldehydes

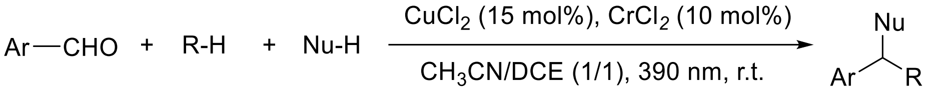

General procedure A: In glove box, to an oven-dried 10 mL Schlenk tube equipped with a Teflon-coated magnetic stirring bar were added aldehyde (if solid, 0.3 mmol, 1.0 equiv), nucleophile (if solid, 0.3 mmol, 1.0 equiv) , $CuCl_2$ (0.045 mmol, 6.1 mg, 15 mol%), $CrCl_2$ (0.03 mmol, 3.7 mg, 10 mol%), CH_3CN (1.0 mL) and DCE (0.5 mL). Then take out of glove box, add aldehyde (if liquid), C-H compound (10-30 equiv) and DCE (0.5 mL) under positive argon pressure. The reaction mixture was stirred under purple light irradiation (50 W, λ_{max} = 390 nm) at room temperature (around 25 ^{o}C) for 72 hours. Then, the solvent was removed *in vacuo* and corresponding product was purified by flash chromatography on silica gel. In each case, the light source was placed ~ 1 cm from the reaction vessel (the light intensity is about 1790 mW/cm^2). The reaction temperature was measured not to exceed room temperature more than 16 °C.

2.3 Procedure of the gram scale experiments

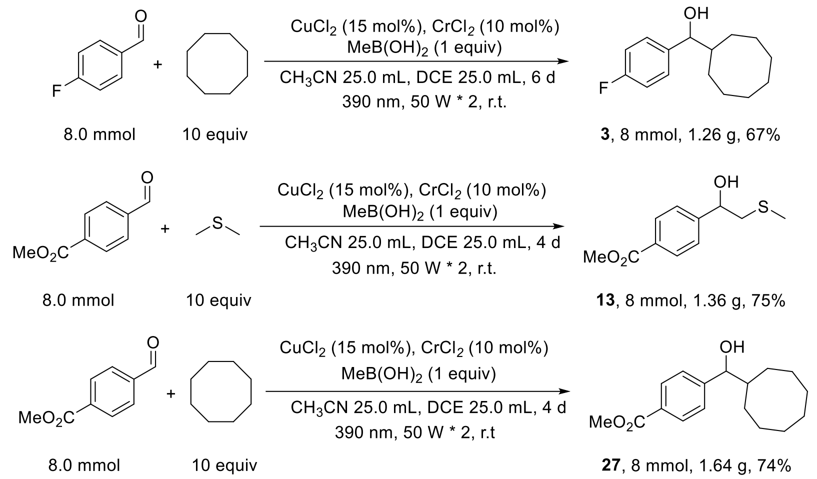

In glove box, to an oven-dried 150 mL Schlenk tube equipped with a Teflon-coated magnetic stirring bar were added aldehyde (if solid, 8.0 mmol, 1.0 equiv), $MeB(OH)_2$ (8.0 mmol, 478.9 mg, 1.0 equiv) , $CuCl_2$ (1.2 mmol, 161.3 mg, 15 mol%), $CrCl_2$ (0.8 mmol, 98.3 mg, 10 mol%), CH_3CN (25.0 mL). Then take out of glove box, add aldehyde (if liquid), C-H compound (10 equiv) and DCE (25.0 mL) under positive argon pressure. The reaction mixture was stirred under purple light irradiation (50 W * 2, λ_{max} = 390 nm) at room temperature (around 25 ^{o}C) for 4-6 days. Then, the solvent was removed *in vacuo* and corresponding product was purified by flash chromatography on silica gel. The distances between reaction tube and light sources were around 4 cm.

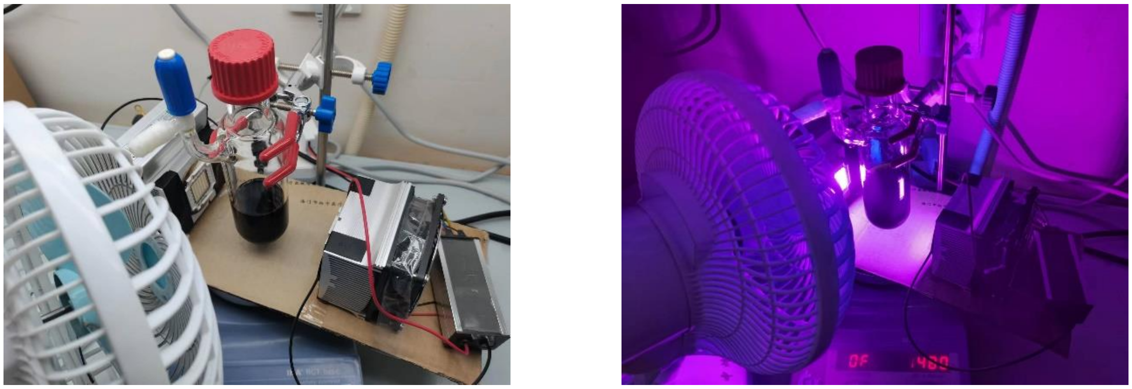

Figure S4. Photographs of the gram experiments

2.4 Luminescence spectroscopy

The emission spectra were recorded in a Fluorospectrophotometer F97Pro equipped with 150 W xenon light source. The samples in CH_3CN were prepared in glove box and recorded in 10×10 mm light path Quartz fluorescence cuvette. The excitation wavelength was 322 nm (incident light slit

width regulated to 2 mm). Emission light slit regulated to 10 mm. Parameters: Data interval = 1 nm, scan speed = 1000 nm/min, response time = 0.2 sec.

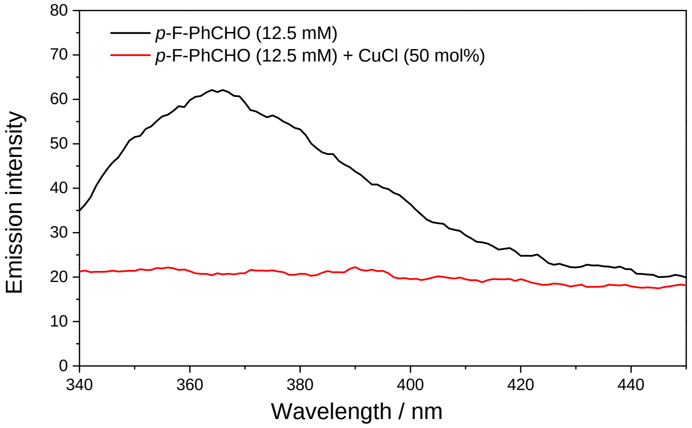

Figure S5. Quenching of the *p*-fluorobenzaldehyde emission by CuCl

The emission intensity decreased with the addition of 0.5 equivalent of CuCl, indicating that the excited aldehyde was reductively quenched by CuCl.

2.5 Studies of interaction between alcohol 3 with $MeB(OH)_2$ by NMR experiments

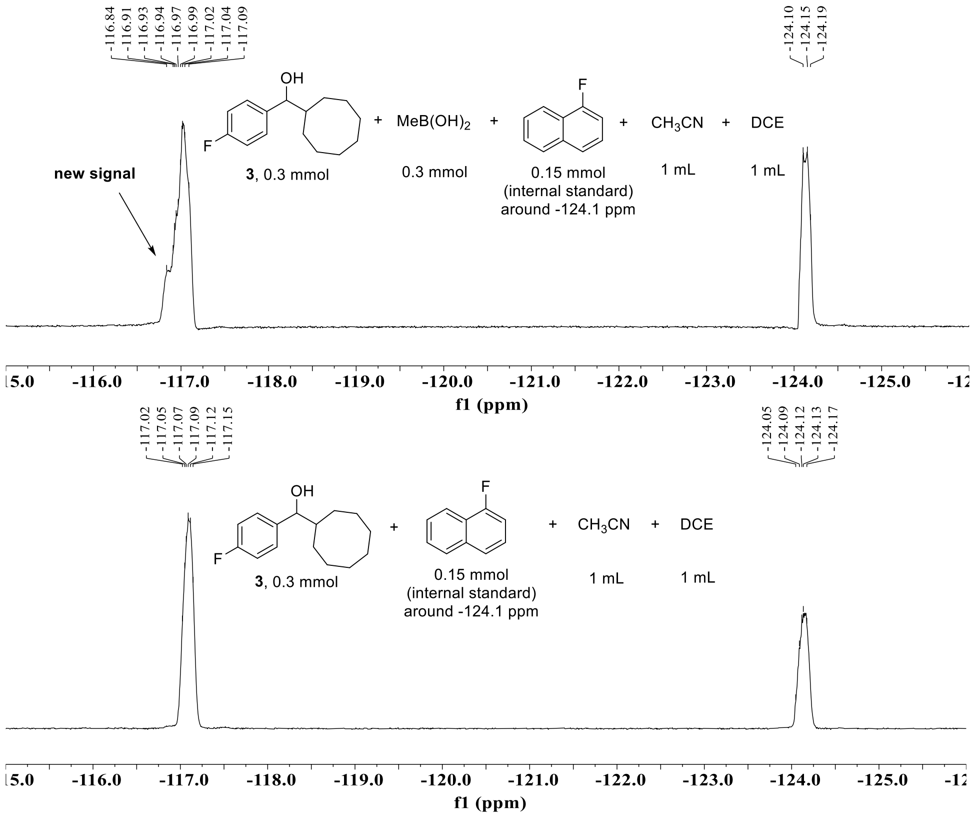

Figure S6. ^{19}F NMR spectra of alcohol **3** with/without $MeB(OH)_2$

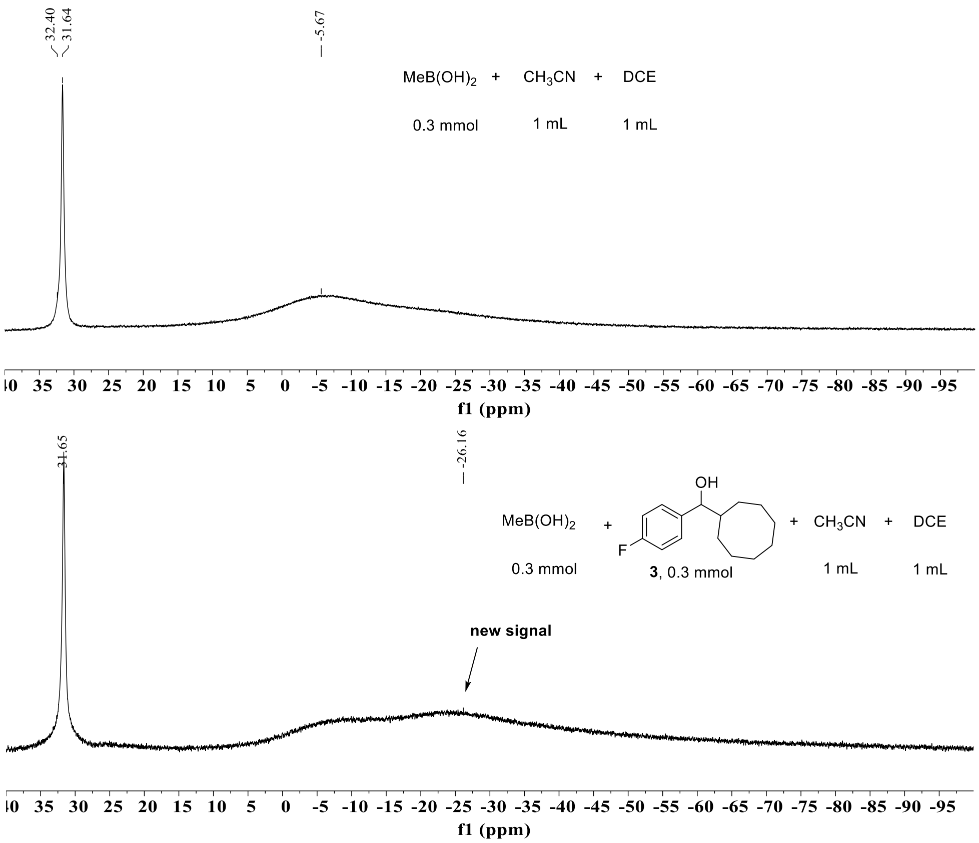

Figure S7. ^{11}B NMR spectra of $MeB(OH)_2$ with/without alcohol **3**

Benzylic alcohol **3** displayed a multiple peak around -117.08 ppm, a new peak (-116.84 ppm) appeared when it was mixed with $MeB(OH)_2$ (Figure S6), illustrating $MeB(OH)_2$ had an interaction with **3**. This was further identified by ^{11}B NMR. $MeB(OH)_2$ displayed a broad peak at -5.67 ppm, this peak became lower and a new peak at -26.16 ppm appeared (Figure S7).

2.6 Cyclic voltammetry experiments

Cyclic voltammetry (CV) experiments were conducted in a 20 mL three-necked cell set-up fitted with a glassy carbon working electrode (3 mm in diameter), an Ag/AgCl reference electrode (saturated KCl solution), and a platinum wire counter electrode. All measurements were carried out in dry DMF with an electrolyte ($^nBu_4NPF_6$, 0.1 M), using a scan rate of 100 mV/s.

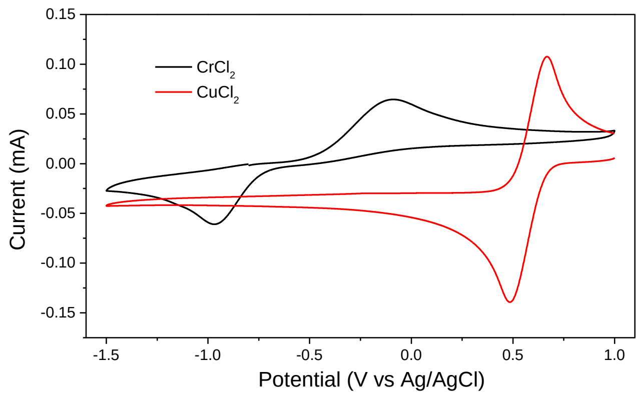

Figure S8. Blank line: $CrCl_2$ (0.06 mmol), $^nBu_4NPF_6$ (0.6 mmol) in 6.0 mL DMF. Red line: $CuCl_2$ (0.06 mmol), $^nBu_4NPF_6$ (0.6 mmol) in 6.0 mL DMF.

2.7 UV-visible absorption spectra

UV-visible absorption spectra were recorded on an Agilent Technologies Cary 60, equipped with a temperature control unit at 25 °C. The spectra were acquired from 200 to 800 nm (or 1000 nm) using 0.5 nm steps. All measurements were performed in CH_3CN and DCE mixed solution (1:1).

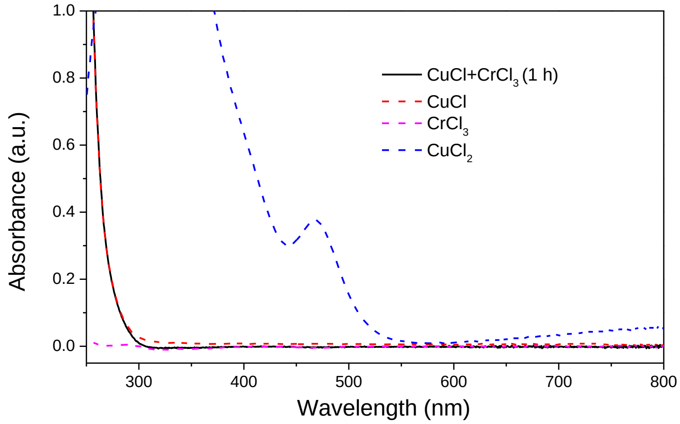

Figure S9. UV-Visible spectra: CuCl (22.5 mM); $CrCl_3$ (22.5 mM); $CuCl_2$ (22.5 mM); CuCl and $CrCl_3$ (22.5 M and 22.5 mM, respectively) stirred for 1 h.

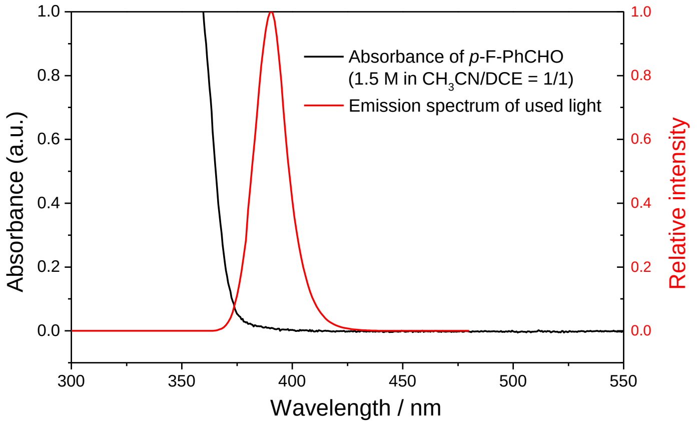

Figure S10. Comparison of UV-Vis spectrum of *p*-F-PhCHO with emission spectrum of used light

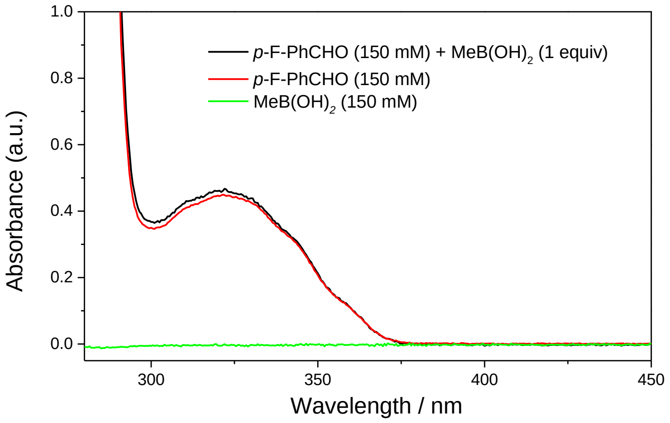

Figure S11. The influence of $MeB(OH)_2$ to *p*-F-PhCHO. $MeB(OH)_2$ and *p*-F-PhCHO (150 mM and 150 mM, respectively) stirred for 40 min.

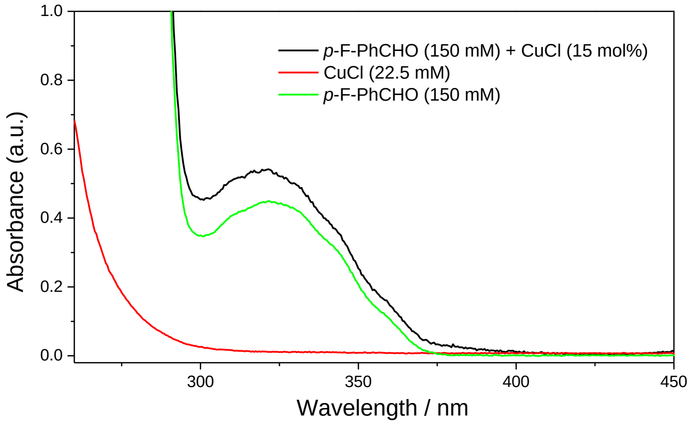

Figure S12. The influence of CuCl to *p*-F-PhCHO. CuCl and *p*-F-PhCHO (22.5 mM and 150 mM, respectively) stirred for 40 min.

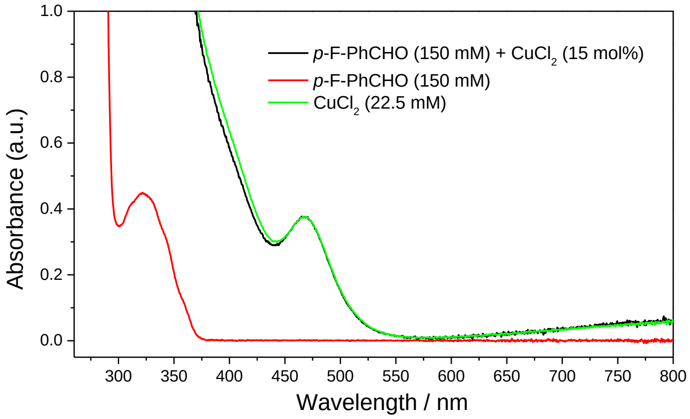

Figure S13. The influence of $CuCl_2$ to *p*-F-PhCHO. $CuCl_2$ and *p*-F-PhCHO (22.5 mM and 150 mM, respectively) stirred for 40 min.

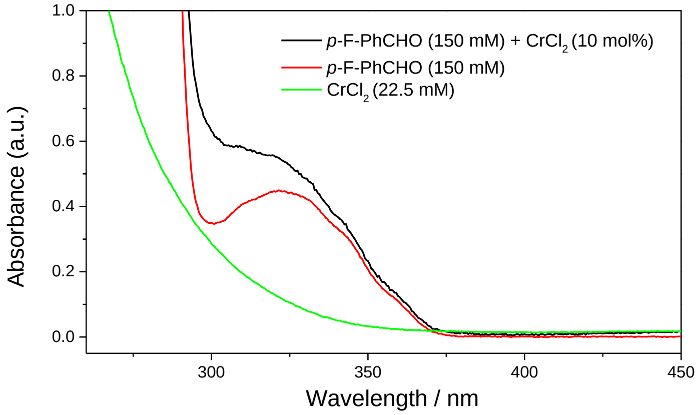

Figure S14. The influence of $CrCl_2$ to *p*-F-PhCHO. $CrCl_2$ and *p*-F-PhCHO (15 mM and 150 mM, respectively) stirred for 40 min.

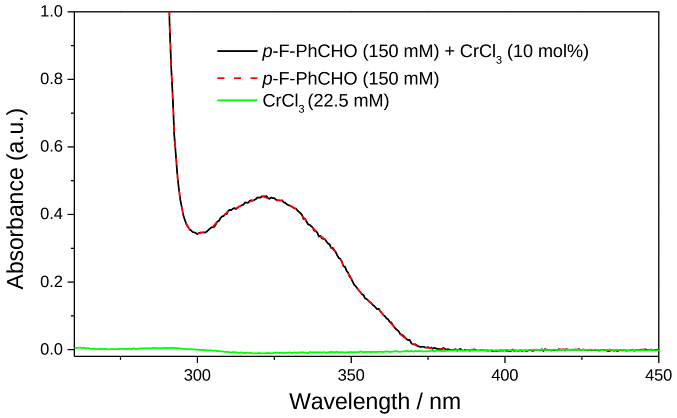

Figure S15. The influence of $CrCl_3$ to *p*-F-PhCHO. $CrCl_3$ and *p*-F-PhCHO (15 mM and 150 mM, respectively) stirred for 40 min.

Add $MeB(OH)_2$ or $CrCl_3$ to *p*-fluorobenzaldehyde, the UV-Vis spectra had no obvious changes (Figure S11, S15). When CuCl or $CrCl_2$ were added to *p*-fluorobenzaldehyde, UV-Vis spectra displayed a red shift (Figure S12, S14).

2.8 Control experiments

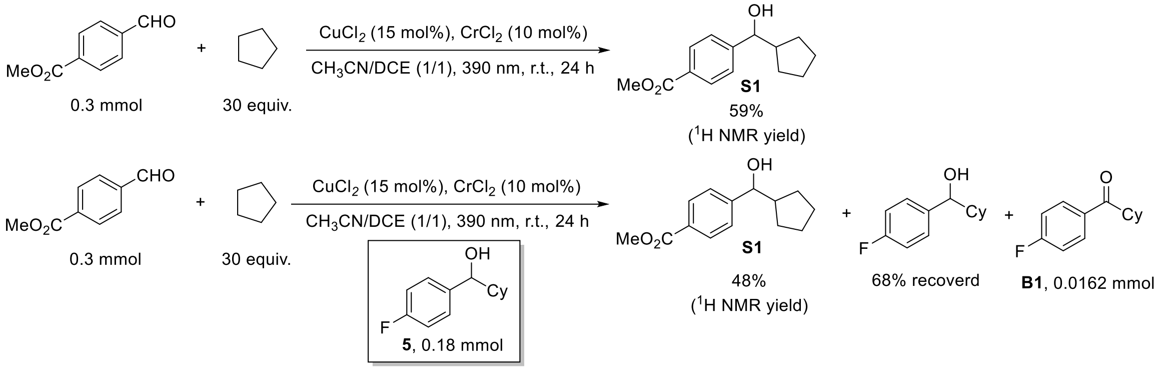

Figure S16. Control experiments

In glove box, to an oven-dried 10 mL Schlenk tube equipped with a Teflon-coated magnetic stirring bar were added methyl 4-formylbenzoate (0.3 mmol, 49.2 mg, 1.0 equiv), with or without **5**, $CuCl_2$ (0.045 mmol, 6.1 mg, 15 mol%), $CrCl_2$ (0.03 mmol, 3.7 mg, 10 mol%), CH_3CN (1.0 mL) and DCE (0.5 mL). Then take out of glove box, added cyclopentane (30 equiv) and DCE (0.5 mL) under positive argon pressure. The reaction mixture was stirred under purple light irradiation (20 W, λ_{max} = 390 nm) at room temperature (around 25 °C) for 24 hours. After the reaction, yields of **S1**, recovery of **5** and ketone byproduct **B1** were obtained by ^{1}H NMR or ^{19}F NMR.

After **5** was added, the yield of **S1** decreased, indicating that **5** inhibits the reaction. **5** was recovered in 68% yield and oxidative byproduct **B1** was detected, illustrating that **5** deteriorated to corresponding ketone in the reaction system.

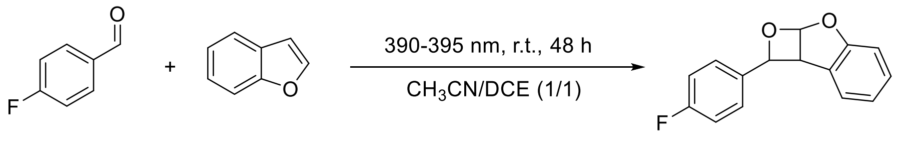

1, 0.3 mmol 0.3 mmol **40**, 49%

Procedure: The Schlenk tube was evacuated and backfilled with argon three times. Subsequently, add aldehyde **1** (0.3 mmol, 37.2 mg, 1.0 equiv), 2,3-benzofuran (0.3 mmol, 35.4 mg, 1.0 equiv), CH_3CN (1.0 mL) and DCE (1.0 mL). The reaction mixture was stirred under purple light irradiation (20 W, λ_{max} = 390 nm) for 48 hours. The volatiles were removed in vacuo, then the

residue was purified by flash column chromatography on silica [EtOAc: Petroleum ether = 1:30 v/v], affording **40** (35.7 mg, 49%) as a faint yellow oil.

^{1}H NMR (400 MHz, Chloroform-*d*) δ 7.44 – 7.34 (m, 2H), 7.26 – 7.15 (m, 2H), 7.10 – 7.02 (m, 2H), 7.01 – 6.90 (m, 2H), 6.64 (d, *J* = 4.2 Hz, 1H), 5.47 (d, *J* = 3.4 Hz, 1H), 4.13 (t, *J* = 3.9 Hz, 1H).

^{13}C NMR (151 MHz, Chloroform-*d*) δ 162.8 (d, *J* = 247.1 Hz), 160.7, 136.8 (d, *J* = 3.2 Hz), 129.7, 127.8, 127.4 (d, *J* = 8.2 Hz), 124.7, 122.2, 115.9 (d, *J* = 21.7 Hz), 111.6, 109.1, 90.4, 52.9.

^{19}F NMR (376 MHz, Chloroform-*d*) δ -113.20 – -113.34 (m).

HR-MS (ESI) *m/z* calcd. for $C_{15}H_{11}FNaO_2^+$ $[M+Na]^+$: 265.0635, found: 265.0642.

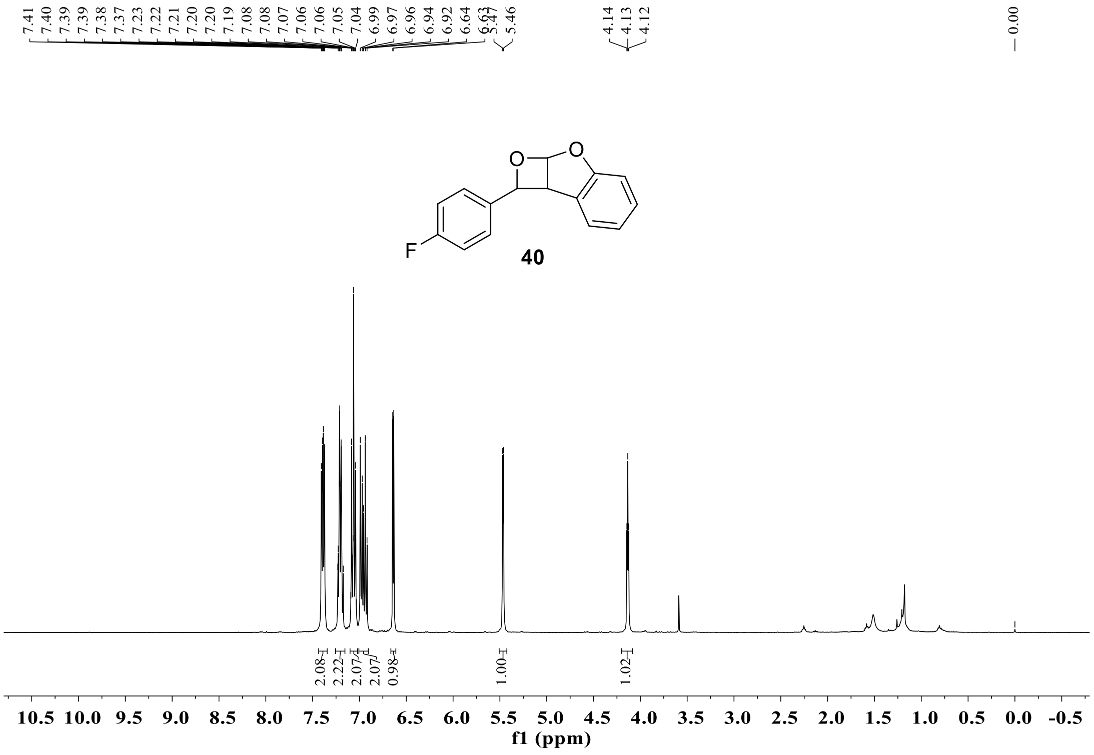

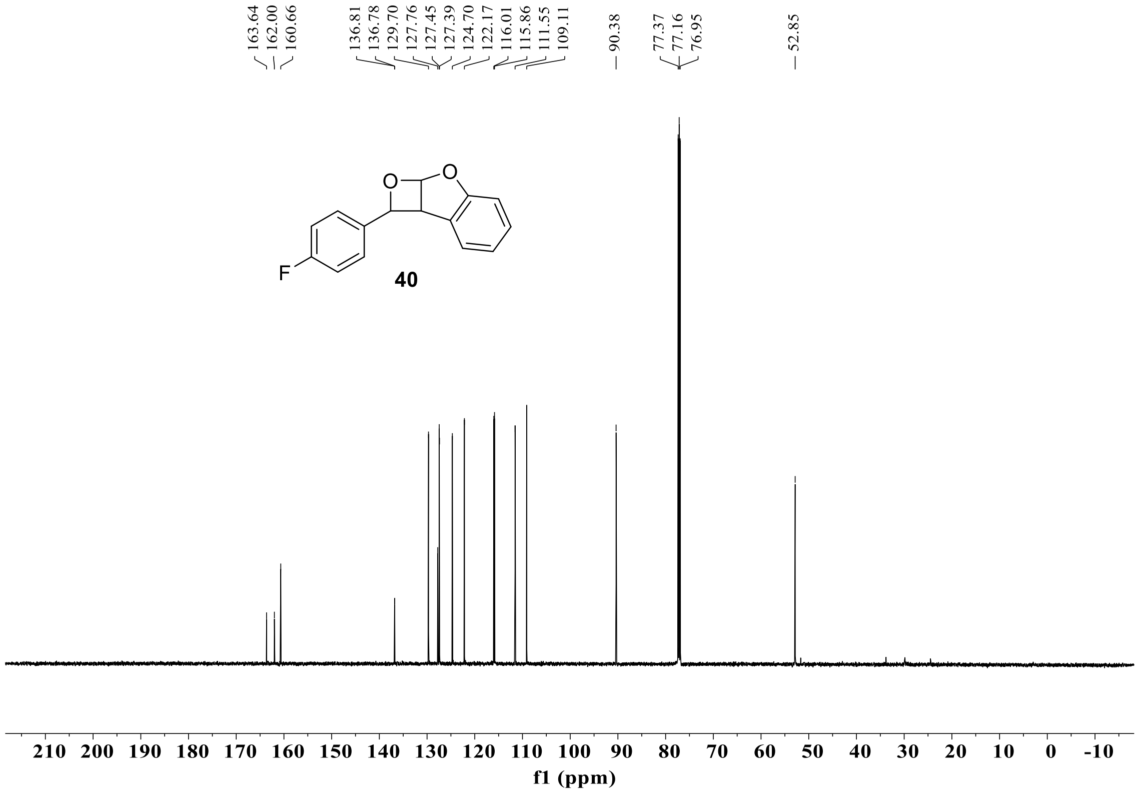
163.64
162.00
160.66
136.81
136.78
129.70
127.76
127.45
127.39
124.70
122.17
116.01
115.86
111.55
109.11
90.38
77.37
77.16
76.95
52.85
F
O
O
40
210 200 190 180 170 160 150 140 130 120 110 100 90 80 70 60 50 40 30 20 10 0 -10
f1 (ppm)

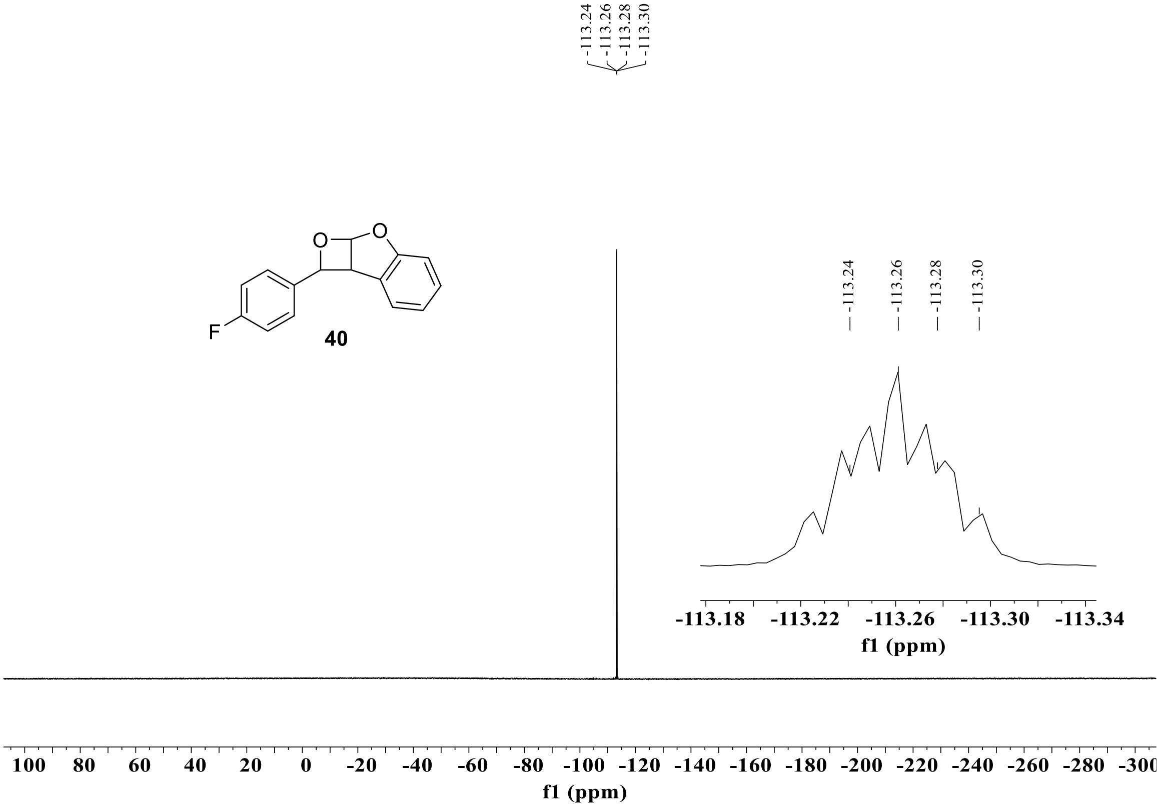
-113.24
-113.26
-113.28
-113.30
F
O
O
40
-113.18 -113.22 -113.26 -113.30 -113.34
f1 (ppm)
100 80 60 40 20 0 -20 -40 -60 -80 -100 -120 -140 -160 -180 -200 -220 -240 -260 -280 -300
f1 (ppm)

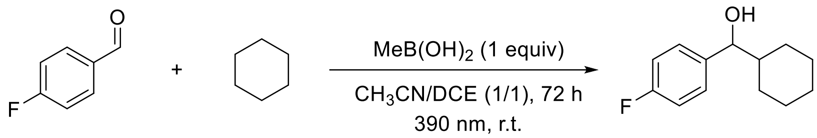

1, 0.3 mmol 30 equiv **5,** 6%

In glove box, to an oven-dried 10 mL Schlenk tube equipped with a Teflon-coated magnetic stirring bar were added $MeB(OH)_2$ (0.3 mmol, 18.0 mg, 1.0 equiv), CH_3CN (1.0 mL) and DCE (0.5 mL). Then take out of glove box, add *p*-fluorobenzaldehyde **1** (0.3 mmol, 37.2 mg, 1.0 equiv), cyclohexane (9.0 mmol, 972 uL, 30 equiv) and DCE (0.5 mL) under positive argon pressure. The reaction mixture was stirred at room temperature under purple light irradiation (20 W, λ_{max} = 390 nm) for 72 hours. The yield was analysed by ^{19}F NMR using 1-fluoronaphthalene as internal standard.

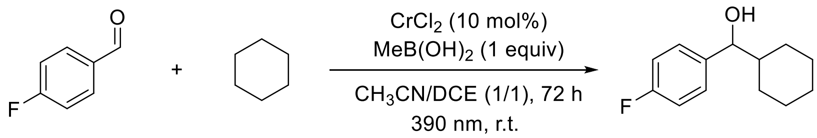

1, 0.3 mmol 30 equiv **5,** 35%

In glove box, to an oven-dried 10 mL Schlenk tube equipped with a Teflon-coated magnetic stirring bar were added $MeB(OH)_2$ (0.3 mmol, 18.0 mg, 1.0 equiv) , $CrCl_2$ (0.03 mmol, 3.7 mg, 10 mol%), CH_3CN (1.0 mL) and DCE (0.5 mL). Then take out of glove box, add *p*-fluorobenzaldehyde **1** (0.3 mmol, 37.2 mg, 1.0 equiv), cyclohexane (9.0 mmol, 972 uL, 30 equiv) and DCE (0.5 mL) under positive argon pressure. The reaction mixture was stirred at room temperature under purple light irradiation (20 W, λ_{max} = 390 nm) for 72 hours. The yield was analysed by ^{19}F NMR using 1-fluoronaphthalene as internal standard.

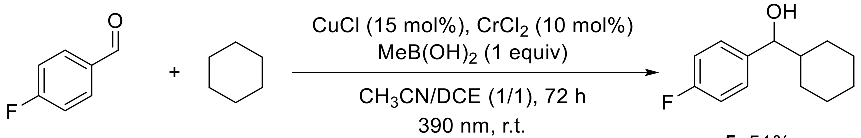

1, 0.3 mmol 30 equiv **5,** 51%

In glove box, to an oven-dried 10 mL Schlenk tube equipped with a Teflon-coated magnetic stirring bar were added $MeB(OH)_2$ (0.3 mmol, 18.0 mg, 1.0 equiv) , CuCl (0.045 mmol, 4.5 mg, 15 mol%), $CrCl_2$ (0.03 mmol, 3.7 mg, 10 mol%), CH_3CN (1.0 mL) and DCE (0.5 mL). Then take out of glove box, add *p*-fluorobenzaldehyde **1** (0.3 mmol, 37.2 mg, 1.0 equiv), cyclohexane (9.0 mmol, 972 uL, 30 equiv) and DCE (0.5 mL) under positive argon pressure. The reaction mixture

was stirred at room temperature under purple light irradiation (20 W, λ_{max} = 390 nm) for 72 hours. The yield was analysed by ^{19}F NMR using 1-fluoronaphthalene as internal standard.

2.9 The reactivity of aliphatic aldehydes

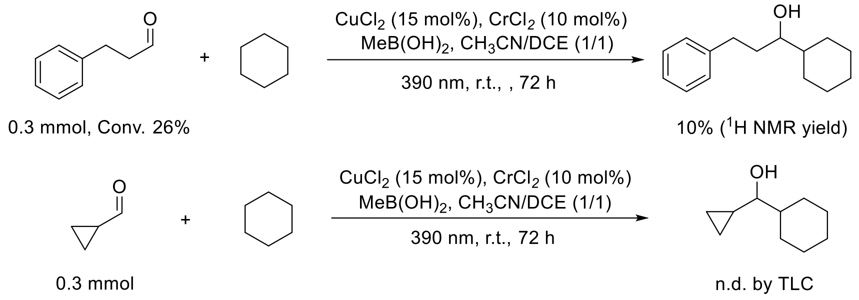

Figure S17. The reactivity of aliphatic aldehydes

2.10 Control experiments and proposed mechanism for 1,1-difunctionalization of aldehyde

Control experiments for 1,1-difunctionalization of aldehydes: The control experiments were conducted according to the general procedure A in the absence of $CuCl_2$, $CrCl_2$ or both. After the reactions, the reaction mixtures were analyzed by GC using 1-fluoronaphthalene as internal standard.

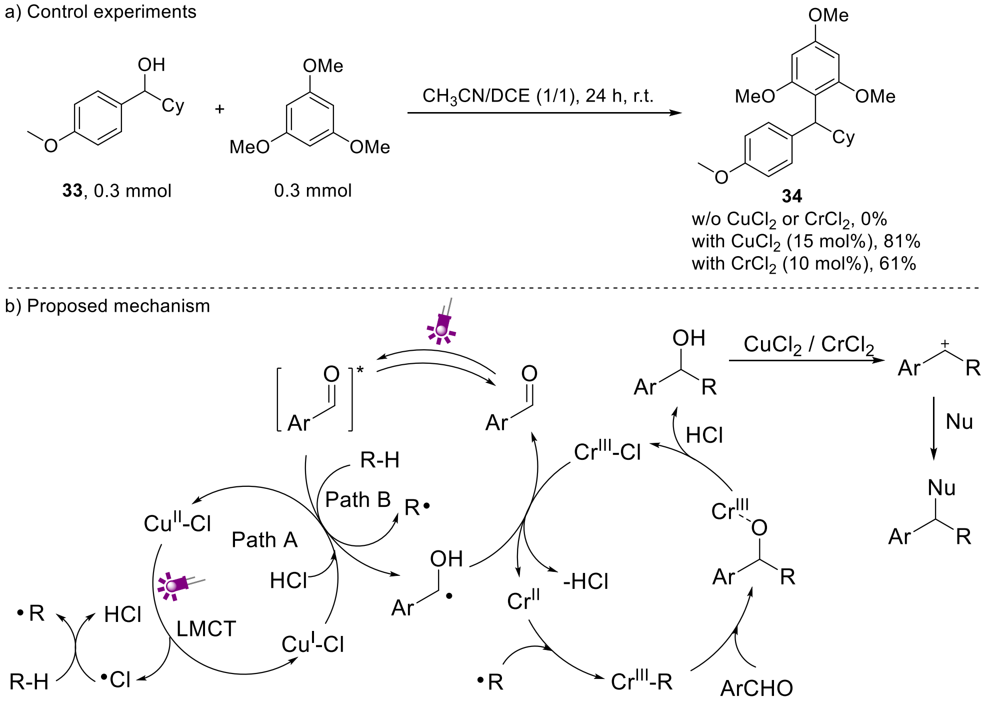

Figure S18. Control experiments and proposed mechanism for 1,1-difunctionalization of aldehyde

2.11 Detection of alkyl radical or benzyl radical

Radical trapping experiments: In glove box, to an oven-dried 10 mL Schlenk tube equipped with a Teflon-coated magnetic stirring bar were added methyl 2-((phenylsulfonyl)methyl)acrylate (0.3 mmol, 1.0 equiv), $MeB(OH)_2$ (0.3 mmol, 18.0 mg, 1.0 equiv), $CuCl_2$ (0.045 mmol, 6.1 mg, 15 mol%), $CrCl_2$ (0.03 mmol, 3.7 mg, 10 mol%), CH_3CN (1.0 mL) and DCE (0.5 mL). Then take out of glove box, add *p*-fluorobenzaldehyde (0.3 mmol, 37.2 mg, 1.0 equiv), cyclohexane (9.0 mmol, 972 uL, 30 equiv) and DCE (0.5 mL) under positive argon pressure. The reaction mixture was stirred under purple light irradiation (20 W, λ_{max} = 390 nm) at room temperature (around 25 ^oC) for 48 hours. Then, the reaction mixtures were analyzed by GC-MS.

The reaction of p-fluorobenzaldehyde and cyclohexane was strongly inhibited if the radical acceptor, methyl 2-((phenylsulfonyl)methyl)acrylate, was added to the standard reaction, where the cyclohexyl radical substitution product **R1** was clearly observed by GC-MS analysis (eqs 1).

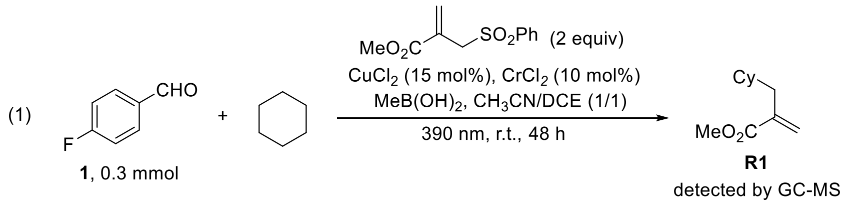

GC-MS data:

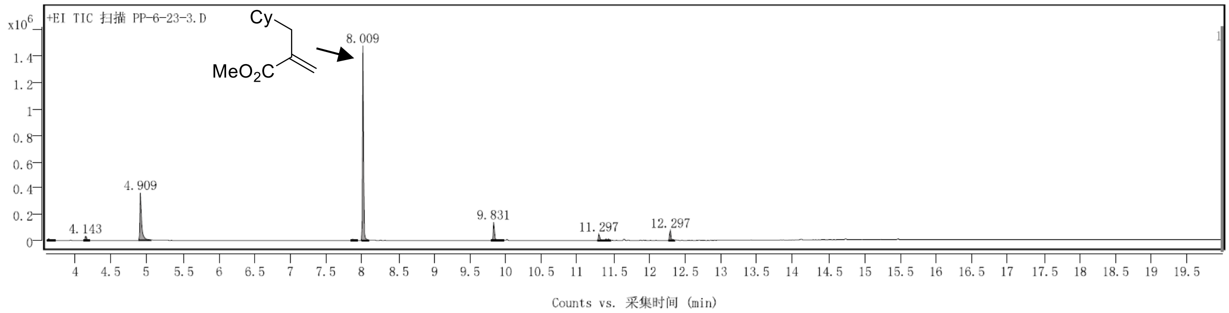

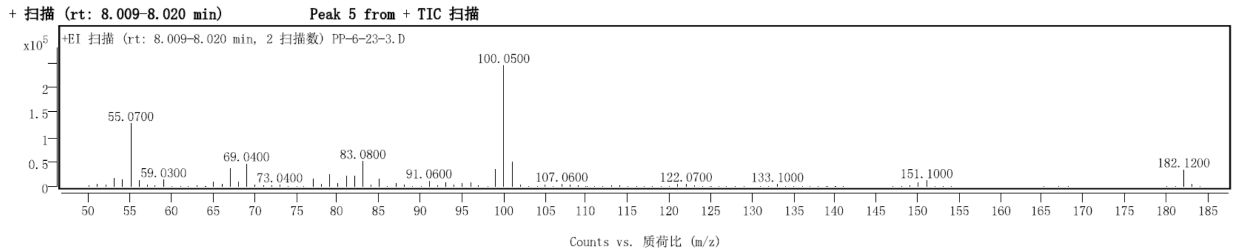

Furthermore, a series of EPR studies are also conducted. There is no obvious EPR signal is observed for the reaction of *p*-fluorobenzaldehyde and cyclohexane under standard conditions (eqs 2).

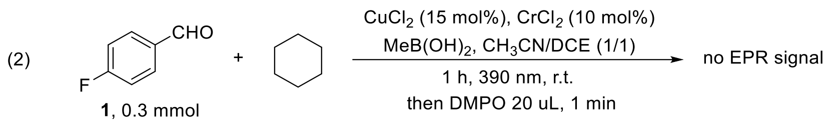

In contrast, a clearly EPR signal is observed when the reaction mixture of *p*-fluorobenzaldehyde and cyclohexane is directly irradiated by purple LEDs (eqs 3). A carbon radical-DMPO adduct was involved in this reaction through further data fitting (g = 2.0064 A_N = 14.41 G, A_H = 21.6 G). Moreover, both of the benzyl radical-DMPO adduct (**R3**) and the cyclohexyl radical-DMPO (**R2**) could be detected by HRMS analysis.

These results demonstrate that a carbon radical is generated in situ and aldehyde also serves as a photosensitizer in the reaction.

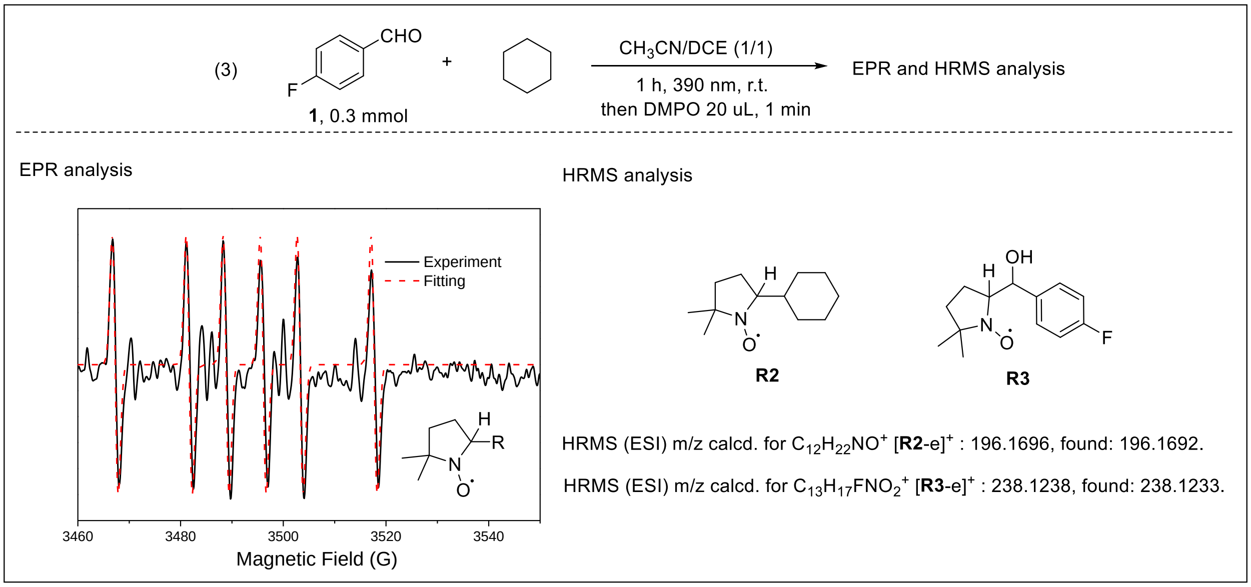

Figure S19. Detection of benzyl radical or alkyl radical by EPR and HRMS

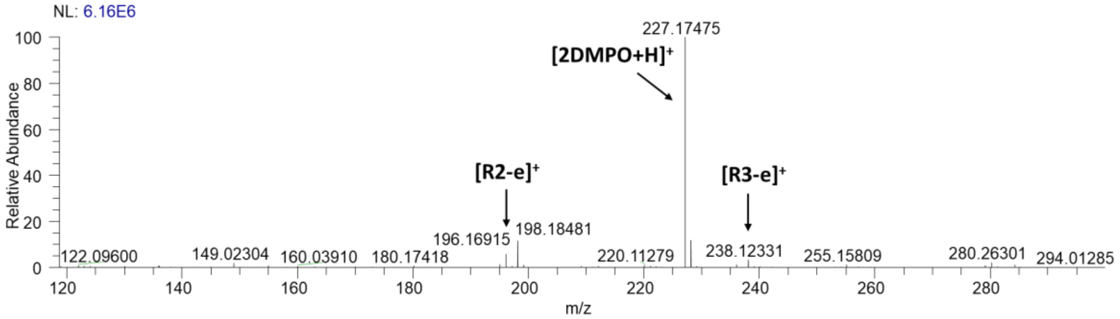

Figure S20. HRMS raw data

2.12 Analytical data of compounds

Cyclooctyl(4-fluorophenyl)methanol (3)

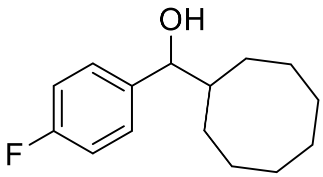

Prepared following the general procedure using methyl *p*-fluorobenzaldehyde (0.3 mmol, 37.2 mg, 1.0 equiv), cyclooctane (3.0 mmol, 404 uL, 10 equiv), methylboronic acid (0.3 mmol, 18.0 mg, 1.0 equiv), copper(II) chloride (0.045 mmol, 6.1 mg, 15 mol%), chromium(II) chloride (0.03 mmol, 3.7 mg, 10 mol%), acetonitrile (1.0 mL) and dichloroethane (1.0 mL). After 36 hours of irradiation, this compound was purified by column chromatography [EtOAc: Petroleum ether = 1:30 v/v] to give the product as a colorless oil (52.1 mg, 73%).

^{1}H NMR (600 MHz, Chloroform-*d*) δ 7.23 – 7.17 (m, 2H), 6.97 – 6.90 (m, 2H), 4.32 (d, *J* = 6.8 Hz, 1H), 1.84 (s, 1H), 1.81 – 1.71 (m, 2H), 1.66 – 1.58 (m, 1H), 1.56 – 1.44 (m, 4H), 1.43 –1.36 (m, 3H), 1.34 – 1.23 (m, 4H), 1.16 – 1.09 (m, 1H).

^{13}C NMR (151 MHz, Chloroform-*d*) δ 162.1 (d, *J* = 245.0 Hz), 139.8 (d, *J* = 3.2 Hz), 128.3 (d, *J* = 8.0 Hz), 115.1 (d, *J* = 21.2 Hz), 79.0, 44.4, 29.7, 27.9, 27.0, 26.8, 26.7, 25.9, 25.7.

^{19}F NMR (565 MHz, Chloroform-*d*) δ -115.41 – -115.49 (m).

HR-MS (ESI) *m/z* calcd. for $C_{15}H_{22}FO^{+}$ $[M+H]^{+}$: 237.1649, found: 237.1646.

Cyclopentyl(4-fluorophenyl)methanol (4)[1]

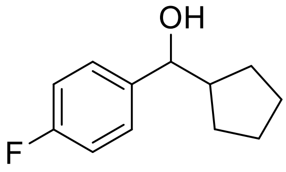

Prepared following the general procedure using methyl *p*-fluorobenzaldehyde (0.3 mmol, 37.2 mg, 1.0 equiv), cyclopentane (9.0 mmol, 840 uL, 30 equiv), methylboronic acid (0.3 mmol, 18.0 mg, 1.0 equiv), copper(II) chloride (0.045 mmol, 6.1 mg, 15 mol%), chromium(II) chloride (0.03 mmol, 3.7 mg, 10 mol%), acetonitrile (1.0 mL) and dichloroethane (1.0 mL). After 72 hours of irradiation, this compound was purified by column chromatography [EtOAc: Petroleum ether = 1:30 v/v] to give the product as a colorless oil (43.2 mg, 74%).

^{1}H NMR (600 MHz, Chloroform-*d*) δ 7.34 – 7.27 (m, 2H), 7.06 – 6.96 (m, 2H), 4.37 (d, *J* = 8.4 Hz, 1H), 2.17 (tdd, *J* = 16.5, 16.5, 8.2 Hz, 1H), 1.96 (brs, 1H), 1.91 – 1.82 (m, 1H), 1.70 – 1.62 (m, 1H), 1.62 – 1.54 (m, 2H), 1.52 – 1.43 (m, 2H), 1.35 (dtd, *J* = 12.1, 7.8, 4.0 Hz, 1H), 1.11 (dq, *J* = 12.7, 8.5 Hz, 1H).

^{13}C NMR (151 MHz, Chloroform-*d*) δ 162.3 (d, *J* = 245.2 Hz), 140.3 (d, *J* = 3.2 Hz), 128.2 (d, *J* = 8.1 Hz), 115.2 (d, *J* = 21.2 Hz), 78.6, 47.9, 29.6, 29.5, 25.6, 25.5.

^{19}F NMR (565 MHz, Chloroform-*d*) δ -115.17 – -115.25 (m).

Cyclohexyl(4-fluorophenyl)methanol (5)[2]

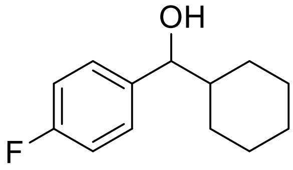

Prepared following the general procedure using methyl *p*-fluorobenzaldehyde (0.3 mmol, 37.2 mg, 1.0 equiv), cyclohexane (9.0 mmol, 972 uL, 30 equiv), methylboronic acid (0.3 mmol, 18.0 mg, 1.0 equiv), copper(II) chloride (0.045 mmol, 6.1 mg, 15 mol%), chromium(II) chloride (0.03 mmol, 3.7 mg, 10 mol%), acetonitrile (1.0 mL) and dichloroethane (1.0 mL). After 72 hours of irradiation, this compound was purified by column chromatography [EtOAc: Petroleum ether = 1:30 v/v] to give the product as a colorless oil (47.2 mg, 76%).

^{1}H NMR (600 MHz, Chloroform-*d*) δ 7.23 – 7.11 (m, 2H), 7.00 – 6.83 (m, 2H), 4.26 (d, *J* = 7.2 Hz, 1H), 1.93 – 1.78 (m, 2H), 1.72 – 1.66 (m, 1H), 1.62 – 1.53 (m, 2H), 1.52 – 1.45 (m, 1H), 1.31 – 1.24 (m, 1H), 1.18 – 0.99 (m, 3H), 0.98 – 0.89 (m, 1H), 0.86 – 0.77 (m, 1H);

^{13}C NMR (151 MHz, Chloroform-*d*) δ 162.2 (d, *J* = 245.1 Hz), 139.4 (d, *J* = 3.2 Hz), 128.3 (d, *J* = 8.0 Hz), 115.1 (d, *J* = 21.2 Hz), 78.8, 45.1, 29.3, 28.9, 26.5, 26.1, 26.1.

^{19}F NMR (565 MHz, Chloroform-*d*) δ -115.32 – -115.39 (m).

(Bicyclo[2.2.1]heptan-2-yl)(4-fluorophenyl)methanol (6)[3]

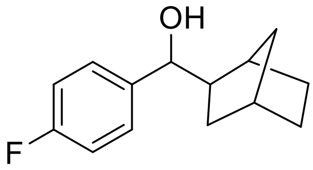

Prepared following the general procedure using *p*-fluorobenzaldehyde (0.3 mmol, 37.2 mg, 1.0 equiv), norbornane (3.0 mmol, 288.5 mg, 10 equiv), methylboronic acid (0.3 mmol, 18.0 mg, 1.0 equiv), copper(II) chloride (0.045 mmol, 6.1 mg, 15 mol%), chromium(II) chloride (0.03 mmol, 3.7 mg, 10 mol%), acetonitrile (1.0 mL) and dichloroethane (1.0 mL). After 72 hours of irradiation, this compound was purified by column chromatography [EtOAc: Petroleum ether = 1:30 v/v] to give the product as a colorless oil (33.1 mg, 50%, 1.1:1 mixture of diastereomers determined by ^{1}H NMR, 0.53H : 0.47H).

^{1}H NMR (600 MHz, Chloroform-*d*) of mixture of diastereomers: δ 7.32 – 7.26 (m, 2H), 7.06 – 6.98 (m, 2H), 4.23 (d, *J* = 9.6 Hz, 0.5H), 4.15 (d, *J* = 10.0 Hz, 0.5H), 2.50 (d, *J* = 4.3 Hz, 0.5H), 2.32 (t, *J* = 4.1 Hz, 0.5H), 2.17 (dd, *J* = 4.4, 4.4 Hz, 0.5H), 1.86 – 1.74 (m, 1.5H), 1.74 – 1.69 (m, 1H), 1.60 (tt, *J* = 12.1, 4.6 Hz, 0.5H), 1.55 – 1.44 (m, 2H), 1.42 – 1.34 (m, 1.5H), 1.29 – 1.22 (m, 0.5H), 1.19 – 1.14 (m, 1H), 1.14 – 1.09 (m, 0.5H), 1.09 – 1.03 (m, 1H), 0.98 (ddd, *J* = 12.6, 8.7,

2.5 Hz, 0.5H), 0.87 – 0.81 (m, 0.5H).

^{13}C NMR (151 MHz, Chloroform-*d*) of mixture of diastereomers: δ 162.4 (d, *J* = 245.7 Hz), 162.3(6) (d, *J* = 245.4 Hz), 140.4 (d, *J* = 3.1 Hz), 139.0 (d, *J* = 3.2 Hz), 129.0 (d, *J* = 7.9 Hz), 128.4 (d, *J* = 8.1 Hz), 115.3(2) (d, *J* = 21.2 Hz), 115.3(1) (d, *J* = 21.3 Hz), 78.4, 77.5, 50.5, 49.8, 39.0, 38.5, 37.1, 36.7, 36.0, 35.6, 35.3, 34.5, 30.3, 30.2, 28.8.

^{19}F NMR (565 MHz, Chloroform-*d*) mixture of diastereomers: (Major isomer) δ -114.79 – -114.89 (m), (Minor isomer) δ -114.98 – -115.09 (m).

HR-MS (ESI) *m/z* calcd. for $C_{14}H_{17}FNaO^+$ $[M+Na]^+$: 243.1156, found: 243.1156.

Methyl 4-(1-hydroxy-2-(p-tolyl)ethyl)benzoate (7)

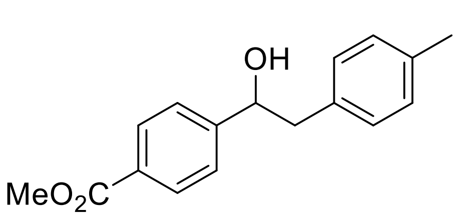

Prepared following the general procedure using methyl 4-formylbenzoate (0.3 mmol, 49.2 mg, 1.0 equiv), paraxylene (3.0 mmol, 370 uL, 10 equiv), methylboronic acid (0.3 mmol, 18.0 mg, 1.0 equiv), copper(II) chloride (0.045 mmol, 6.1 mg, 15 mol%), chromium(II) chloride (0.03 mmol, 3.7 mg, 10 mol%), acetonitrile (1.0 mL) and dichloroethane (1.0 mL). After 36 hours of irradiation, this compound was purified by column chromatography [EtOAc: Petroleum ether = 1:10 v/v] to give the product as a white solid (36.8 mg, 45%).

^{1}H NMR (600 MHz, Chloroform-*d*) δ 8.04 – 7.96 (m, 2H), 7.43 – 7.38 (m, 2H), 7.13 – 7.08 (m, 2H), 7.08 – 7.02 (m, 2H), 4.91 (dd, *J* = 8.5, 4.9 Hz, 1H), 3.91 (s, 3H), 3.04 – 2.90 (m, 2H), 2.33 (s, 3H), 2.17 (brs, 1H).

^{13}C NMR (151 MHz, Chloroform-*d*) δ 167.1, 149.1, 136.5, 134.3, 129.8, 129.5, 129.4, 129.3, 126.0, 75.0, 52.2, 45.7, 21.2.

HR-MS (ESI) *m/z* calcd. for $C_{17}H_{19}O_3^+$ $[M+H]^+$: 271.1329, found: 271.1330.

Methyl 4-(2-(3,5-dimethylphenyl)-1-hydroxyethyl)benzoate (8)

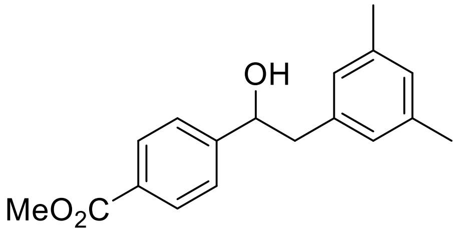

Prepared following the general procedure using methyl 4-formylbenzoate (0.3 mmol, 49.2 mg, 1.0 equiv), mesitylene (3.0 mmol, 417 uL, 10 equiv), methylboronic acid (0.3 mmol, 18.0 mg, 1.0 equiv), copper(II) chloride (0.045 mmol, 6.1 mg, 15 mol%), chromium(II) chloride (0.03 mmol, 3.7 mg,

10 mol%), acetonitrile (1.0 mL) and dichloroethane (1.0 mL). After 36 hours of irradiation, this compound was purified by column chromatography [EtOAc: Petroleum ether = 1:10 v/v] to give the product as a white solid (31.1 mg, 37%).

^{1}H NMR (600 MHz, Chloroform-*d*) δ 8.05 – 8.00 (m, 2H), 7.48 – 7.41 (m, 2H), 6.90 (s, 1H), 6.83 (s, 2H), 4.93 (dd, *J* = 9.2, 4.2 Hz, 1H), 3.92 (s, 3H), 3.01 – 2.81 (m, 2H), 2.30 (s, 6H), 2.04 (brs, 1H).

^{13}C NMR (151 MHz, Chloroform-*d*) δ 167.2, 149.2, 138.3, 137.4, 129.8, 129.3, 128.6, 127.4, 125.9, 74.9, 52.2, 46.2, 21.4.

HR-MS (ESI) *m/z* calcd. for $C_{18}H_{20}NaO_3^+$ $[M+Na]^+$: 307.1305, found: 307.1312.

Methyl 4-(2-(4-ethylphenyl)-1-hydroxypropyl)benzoate (9)

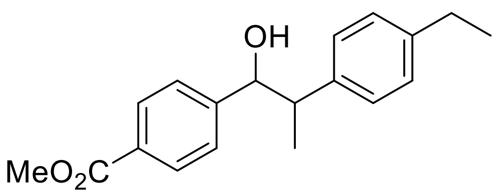

Prepared following the general procedure using methyl 4-formylbenzoate (0.3 mmol, 49.2 mg, 1.0 equiv), 1,4-diethylbenzene (3.0 mmol, 467 uL, 10 equiv), methylboronic acid (0.3 mmol, 18.0 mg, 1.0 equiv), copper(II) chloride (0.045 mmol, 6.1 mg, 15 mol%), chromium(II) chloride (0.03 mmol, 3.7 mg, 10 mol%), acetonitrile (1.0 mL) and dichloroethane (1.0 mL). After 48 hours of irradiation, this compound was purified by column chromatography [EtOAc: Petroleum ether = 1:10 v/v] to give the product as a white solid (42.1 mg, 47%, 1:1 mixture of diastereomers determined by ^{1}H NMR, 0.5H : 0.5H).

^{1}H NMR (600 MHz, Chloroform-*d*) δ 8.05 – 7.98 (m, 1H), 7.97 – 7.90 (m, 1H), 7.44 – 7.37 (m, 1H), 7.31 – 7.27 (m, 1H), 7.21 – 7.16 (m, 2H), 7.13 – 7.04 (m, 2H), 4.84 (d, *J* = 5.5 Hz, 0.5H), 4.68 (d, *J* = 8.4 Hz, 0.5H), 3.92 (s, 1.5H), 3.90 (s, 1.5H), 3.12 – 3.05 (m, 0.5H), 2.98 (dq, *J* = 8.4, 7.1 Hz, 0.5H), 2.66 (q, *J* = 7.7 Hz, 1H), 2.61 (q, *J* = 7.7 Hz, 1H), 2.08 (brs, 1H), 1.30 – 1.20 (m, 4.5H), 1.08 (d, *J* = 7.0 Hz, 1.5H).

^{13}C NMR (151 MHz, Chloroform-*d*) δ 167.2, 167.1, 148.3, 147.9, 143.1, 142.7, 140.3, 139.8, 129.6(3), 129.5(6), 129.4, 129.0, 128.3, 128.1, 128.0(1), 127.9(5), 127.1, 126.4, 79.3, 78.3, 52.2, 52.1, 47.8, 46.7, 28.5(4), 28.4(9), 18.2, 15.6(2), 15.5(9), 14.6.

HR-MS (ESI) m/z calcd. for $C_{19}H_{22}NaO_3^+$ $[M+Na]^+$: 321.1461, found: 321.1461.

4-(1-Hydroxy-2-(methylthio)ethyl)benzoate (10)

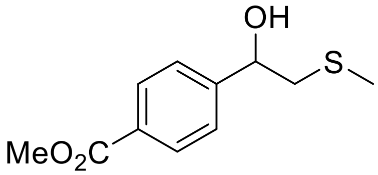

Prepared following the general procedure using methyl 4-formylbenzoate (0.3 mmol, 49.2 mg, 1.0 equiv), dimethyl sulfide (3.0 mmol, 220 uL, 10 equiv), methylboronic acid (0.3 mmol, 18.0 mg, 1.0 equiv), copper(II) chloride (0.045 mmol, 6.1 mg, 15 mol%), chromium(II) chloride (0.03 mmol, 3.7 mg, 10 mol%), acetonitrile (1.0 mL) and dichloroethane (1.0 mL). After 72 hours of irradiation, this compound was purified by column chromatography [EtOAc: Petroleum ether = 1:5 v/v] to give the product as a white solid (58.2 mg, 86%).

^{1}H NMR (600 MHz, Chloroform-*d*) δ 7.99 (d, *J* = 8.4 Hz, 2H), 7.42 (d, *J* = 8.2 Hz, 2H), 4.79 (dd, *J* = 9.2, 3.6 Hz, 1H), 3.88 (s, 3H), 3.18 (brs, 1H), 2.95 – 2.77 (m, 1H), 2.74 – 2.58 (m, 1H), 2.10 (s, 3H).

^{13}C NMR (151 MHz, Chloroform-*d*) δ 166.9, 147.7, 129.9, 129.6, 125.8, 70.8, 52.2, 43.9, 15.6.

HR-MS (ESI) *m/z* calcd. for $C_{11}H_{14}NaO_3S^+$ [M+Na]$^+$: 249.0556, found: 249.0553.

Methyl 4-(hydroxy(tetrahydrothiophen-2-yl)methyl)benzoate (11)

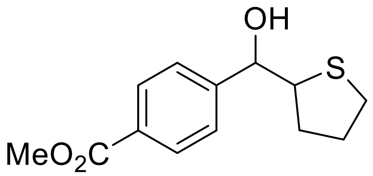

Prepared following the general procedure using methyl 4-formylbenzoate (0.3 mmol, 49.2 mg, 1.0 equiv), tetrahydrothiophene (6.0 mmol, 530 uL, 10 equiv), methylboronic acid (0.3 mmol, 18.0 mg, 1.0 equiv), copper(II) chloride (0.045 mmol, 6.1 mg, 15 mol%), chromium(II) chloride (0.03 mmol, 3.7 mg, 10 mol%), acetonitrile (1.0 mL) and dichloroethane (1.0 mL). After 48 hours of irradiation, this compound was purified by column chromatography [EtOAc: Petroleum ether = 1:8 v/v] to give the product as a colorless oil (36.2 mg, 48%, 1.4:1 mixture of diastereomers determined by ^{1}H NMR, 0.56H : 0.40H).

^{1}H NMR (400 MHz, Chloroform-*d*) δ 8.07 – 7.94 (m, 2H), 7.48 – 7.35 (m, 2H), 4.78 (d, *J* = 4.6 Hz, 0.4H), 4.61 (d, *J* = 6.0 Hz, 0.56H), 3.90 (s, 3H), 3.84 – 3.71 (m, 1H), 2.98 – 2.57 (m, 3H), 2.17 – 2.03 (m, 1H), 2.00 – 1.91 (m, 1H), 1.90 – 1.66 (m, 2H).

^{13}C NMR (151 MHz, Chloroform-*d*) δ 167.1, 167.0, 148.1, 147.1, 129.9, 129.8, 129.7, 129.5, 126.2(3), 126.1(5), 76.0, 74.1, 57.8, 56.3, 52.2(3), 52.2(1), 34.2, 33.3, 32.7, 31.4, 30.9, 30.8.

HR-MS (ESI) *m/z* calcd. for $C_{13}H_{16}NaO_3S^+$ [M+Na]$^+$: 275.0712, found: 275.0714.

1-(4-Fluorophenyl)-2-(methyl(phenyl)amino)ethan-1-ol (12)

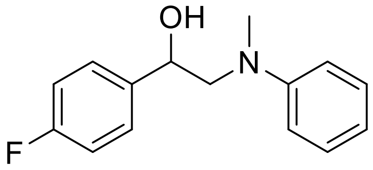

Prepared following the general procedure using 4-formylbenzoate (0.3 mmol, 37.2 mg, 1.0 equiv), N,N-dimethylaniline (3.0 mmol, 379 uL, 10 equiv), methylboronic acid (0.3 mmol, 18.0 mg, 1.0 equiv), copper(II) chloride (0.045 mmol, 6.1 mg, 15 mol%), chromium(II) chloride (0.03 mmol, 3.7 mg, 10 mol%), acetonitrile (1.0 mL) and dichloroethane (1.0 mL). After 72 hours of irradiation, this compound was purified by column chromatography [EtOAc: Petroleum ether = 1:20 v/v] to give the product as a colorless oil (64.4 mg, 88%).

^{1}H NMR (600 MHz, Chloroform-*d*) δ 7.42 – 7.37 (m, 2H), 7.32 – 7.27 (m, 2H), 7.11 – 7.05 (m, 2H), 6.88 – 6.84 (m, 2H), 6.81 (tt, *J* = 7.3, 1.0 Hz, 1H), 4.98 (dd, *J* = 8.9, 4.3 Hz, 1H), 3.52 – 3.38 (m, 2H), 2.94 (s, 3H), 2.68 (brs, 1H).

^{13}C NMR (151 MHz, Chloroform-*d*) δ 162.5 (d, *J* = 245.7 Hz), 150.0, 137.8 (d, *J* = 2.9 Hz), 129.4, 127.7 (d, *J* = 8.1 Hz), 117.8, 115.5 (d, *J* = 21.3 Hz), 113.5, 71.1, 62.2, 39.5.

^{19}F NMR (565 MHz, Chloroform-*d*) δ -115.54 – -114.67 (m).

HR-MS (ESI) *m/z* calcd. for $C_{15}H_{17}FNO^{+}$ $[M+H]^{+}$: 246.1289, found: 246.1286.

2-(Diphenylamino)-1-(4-fluorophenyl)ethan-1-ol (13)

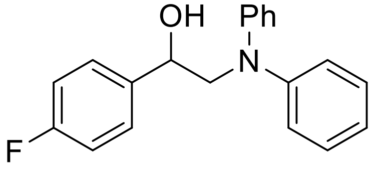

Prepared following the general procedure using 4-formylbenzoate (0.3 mmol, 37.2 mg, 1.0 equiv), N-methyldiphenylamine (3.0 mmol, 524 uL, 10 equiv), methylboronic acid (0.3 mmol, 18.0 mg, 1.0 equiv), copper(II) chloride (0.045 mmol, 6.1 mg, 15 mol%), chromium(II) chloride (0.03 mmol, 3.7 mg, 10 mol%), acetonitrile (1.0 mL) and dichloroethane (1.0 mL). After 48 hours of irradiation, this compound was purified by column chromatography [EtOAc: Petroleum ether = 1:20 v/v] to give the product as a faint yellow oil (69.5 mg, 76%).

^{1}H NMR (600 MHz, Chloroform-*d*) δ 7.40 – 7.36 (m, 2H), 7.32 – 7.27 (m, 4H), 7.08 7.03 (m, 6H), 7.01 (t, *J* = 7.4 Hz, 2H), 4.98 (dd, *J* = 9.0, 4.0 Hz, 1H), 3.97 – 3.84 (m, 2H), 2.52 (brs, 1H).

^{13}C NMR (151 MHz, Chloroform-*d*) δ 162.5 (d, *J* = 245.8 Hz), 148.3, 137.5 (d, *J* = 3.2 Hz), 129.6, 127.8 (d, *J* = 8.1 Hz), 122.2, 121.6, 115.5 (d, *J* = 21.3 Hz), 70.8, 60.9.

^{19}F NMR (565 MHz, Chloroform-*d*) δ -114.44 – -114.52 (m).

HR-MS *m/z* calcd. for $C_{20}H_{19}FNO^{+}$ $[M+H]^{+}$: 308.1445, found: 308.1445.

Cyclohexyl(p-tolyl)methanol (14)[2]

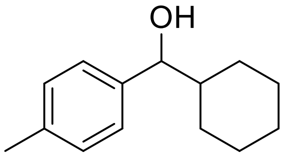

Prepared following the general procedure using methyl *p*-tolualdehyde (0.3 mmol, 36.0 mg, 1.0 equiv), cyclohexane (9.0 mmol, 972 uL, 30 equiv), methylboronic acid (0.3 mmol, 18.0 mg, 1.0 equiv), copper(II) chloride (0.045 mmol, 6.1 mg, 15 mol%), chromium(II) chloride (0.03 mmol, 3.7 mg, 10 mol%), acetonitrile (1.0 mL) and dichloroethane (1.0 mL). After 72 hours of irradiation, this compound was purified by column chromatography [EtOAc: Petroleum ether = 1:30 v/v] to give the product as a colorless oil (34.7 mg, 57%).

^{1}H NMR (600 MHz, Chloroform-*d*) δ 7.19 (d, *J* = 8.0 Hz, 2H), 7.15 (d, *J* = 7.8 Hz, 2H), 4.32 (d, *J* = 7.2 Hz, 1H), 2.35 (s, 3H), 2.04 – 1.97 (m, 1H), 1.82 (brs, 1H), 1.79 – 1.74 (m, 1H), 1.69 – 1.56 (m, 3H), 1.41 – 1.34 (m, 1H), 1.25 – 1.09 (m, 3H), 1.04 (qd, *J* = 12.4, 3.6 Hz, 1H), 0.96 – 0.88 (m, 1H).

^{13}C NMR (151 MHz, Chloroform-*d*) δ 140.8, 137.2, 129.0, 126.7, 79.4, 45.0, 29.4, 29.1, 26.6, 26.2, 26.2, 21.3.

(4-(Tert-butyl)phenyl)(cyclohexyl)methanol (15)

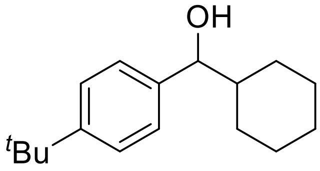

Prepared following the general procedure using methyl 4-*tert*-butylbenzaldehyde (0.3 mmol, 48.7 mg, 1.0 equiv), cyclohexane (9.0 mmol, 972 uL, 30 equiv), methylboronic acid (0.3 mmol, 18.0 mg, 1.0 equiv), copper(II) chloride (0.045 mmol, 6.1 mg, 15 mol%), chromium(II) chloride (0.03 mmol, 3.7 mg, 10 mol%), acetonitrile (1.0 mL) and dichloroethane (1.0 mL). After 72 hours of irradiation, this compound was purified by column chromatography [EtOAc: Petroleum ether = 1:30 v/v] to give the product as a white solid (41.6 mg, 56%).

^{1}H NMR (600 MHz, Chloroform-*d*) δ 7.39 – 7.34 (m, 2H), 7.25 – 7.21 (m, 2H), 4.33 (d, *J* = 7.4 Hz, 1H), 2.06 – 1.99 (m, 1H), 1.93 – 1.82 (m, 1H), 1.81 – 1.75 (m, 1H), 1.70 – 1.58 (m, 3H), 1.41 – 1.36 (m, 1H), 1.33 (s, 9H), 1.26 – 1.11 (m, 3H), 1.09 – 1.01 (m, 1H), 0.96 – 0.89 (m, 1H).

^{13}C NMR (151 MHz, Chloroform-*d*) δ 150.41, 140.78, 126.46, 125.21, 79.34, 44.93, 34.62, 31.51, 29.47, 29.07, 26.57, 26.23, 26.14.

HR-MS (ESI) *m/z* calcd. for $C_{17}H_{26}NaO^{+}$ $[M+Na]^{+}$: 269.1876, found: 269.1878.

[1,1'-biphenyl]-4-yl(cyclohexyl)methanol (16)[4]

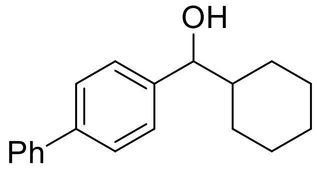

Prepared following the general procedure using methyl 4-biphenylcarboxaldehyde (0.3 mmol, 48.7 mg, 1.0 equiv), cyclohexane (9.0 mmol, 972 uL, 30 equiv), methylboronic acid (0.3 mmol, 18.0 mg, 1.0 equiv), copper(II) chloride (0.045 mmol, 6.1 mg, 15 mol%), chromium(II) chloride (0.03 mmol, 3.7 mg, 10 mol%), acetonitrile (1.0 mL) and dichloroethane (1.0 mL). After 72 hours of irradiation, this compound was purified by column chromatography [EtOAc: Petroleum ether = 1:30 v/v] to give the product as a white solid (32.0 mg, 40%).

^{1}H NMR (400 MHz, Chloroform-*d*) δ 7.65 – 7.54 (m, 4H), 7.45 (t, *J* = 7.5 Hz, 2H), 7.40 – 7.32 (m, 3H), 4.42 (d, *J* = 7.2 Hz, 1H), 2.08 – 1.98 (m, 1H), 1.90 (brs, 1H), 1.84 – 1.75 (m, 1H), 1.73 – 1.60 (m, 3H), 1.45 (d, *J* = 13.0 Hz, 1H), 1.29 – 1.14 (m, 3H), 1.13 – 1.05 (m, 1H), 1.05 – 0.94 (m, 1H).

^{13}C NMR (151 MHz, Chloroform-*d*) δ 142.8, 141.0, 140.4, 128.9, 127.4, 127.2, 127.2, 127.1, 79.3, 45.1, 29.4, 29.0, 26.5, 26.2, 26.1.

Cyclohexyl(naphthalen-1-yl)methanol (17)[5]

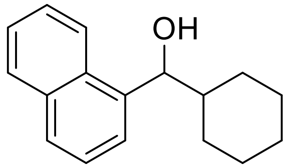

Prepared following the general procedure using 1-naphthaldehyde (0.3 mmol, 46.9 mg, 1.0 equiv), cyclohexane (9.0 mmol, 972 uL, 30 equiv), methylboronic acid (0.3 mmol, 18.0 mg, 1.0 equiv), copper(II) chloride (0.045 mmol, 6.1 mg, 15 mol%), chromium(II) chloride (0.03 mmol, 3.7 mg, 10 mol%), acetonitrile (1.0 mL) and dichloroethane (1.0 mL). After 72 hours of irradiation, this compound was purified by column chromatography [EtOAc: Petroleum ether = 1:30 v/v] to give the product as a faint yellow oil (23.1 mg, 32%).

^{1}H NMR (400 MHz, Chloroform-*d*) δ 8.20 – 8.12 (m, 1H), 7.90 – 7.84 (m, 1H), 7.78 (d, *J* = 8.1 Hz, 1H), 7.59 (d, *J* = 7.1 Hz, 1H), 7.54 – 7.44 (m, 3H), 5.20 (d, *J* = 6.4 Hz, 1H), 2.02 – 1.95 (m, 1H), 1.93 – 1.87 (m, 1H), 1.82 (brs, 1H), 1.80 – 1.72 (m, 1H), 1.69 – 1.62 (m, 2H), 1.46 – 1.37 (m, 1H), 1.24 – 1.10 (m, 5H).

^{13}C NMR (101 MHz, Chloroform-*d*) δ 139.6, 134.0, 131.0, 129.0, 128.0, 125.9, 125.6, 125.4, 124.3, 123.8, 76.2, 44.5, 30.4, 28.4, 26.6, 26.4, 26.2.

Cyclohexyl(phenyl)methanol (18)[2]

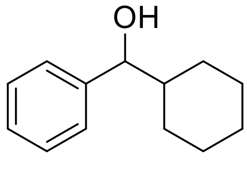

Prepared following the general procedure using benzaldehyde (0.3 mmol, 31.8 mg, 1.0 equiv), cyclohexane (9.0 mmol, 972 uL, 30 equiv), methylboronic acid (0.3 mmol, 18.0 mg, 1.0 equiv), copper(II) chloride (0.045 mmol, 6.1 mg, 15 mol%), chromium(II) chloride (0.03 mmol, 3.7 mg, 10 mol%), acetonitrile (1.0 mL) and dichloroethane (1.0 mL). After 72 hours of irradiation, this compound was purified by column chromatography [EtOAc: Petroleum ether = 1:30 v/v] to give the product as a colorless oil (31.2 mg, 55%).

^{1}H NMR (400 MHz, Chloroform-*d*) δ 7.32 – 7.13 (m, 5H), 4.27 (d, *J* = 7.2 Hz, 1H), 1.95 – 1.86 (m, 1H), 1.84 (brs, 1H) 1.73 – 1.64 (m, 1H), 1.62 – 1.47 (m, 3H), 1.35 – 1.24 (m, 1H), 1.20 – 1.03 (m, 3H), 1.01 – 0.93 (m, 1H), 0.92 – 0.80 (m, 1H).

^{13}C NMR (151 MHz, Chloroform-*d*) δ 143.7, 128.3, 127.5, 126.8, 79.5, 45.1, 29.4, 28.9, 26.5, 26.2, 26.1.

Cyclohexyl(4-(trifluoromethyl)phenyl)methanol (19)

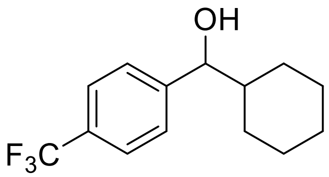

Prepared following the general procedure using 4-(trifluoromethyl)benzaldehyde (0.3 mmol, 52.2 mg, 1.0 equiv), cyclohexane (9.0 mmol, 972 uL, 30 equiv), methylboronic acid (0.3 mmol, 18.0 mg, 1.0 equiv), copper(II) chloride (0.045 mmol, 6.1 mg, 15 mol%), chromium(II) chloride (0.03 mmol, 3.7 mg, 10 mol%), acetonitrile (1.0 mL) and dichloroethane (1.0 mL). After 72 hours of irradiation, this compound was purified by column chromatography [EtOAc: Petroleum ether = 1:30 v/v] to give the product as colorless oil (53.2 mg, 69%).

^{1}H NMR (600 MHz, Chloroform-*d*) δ 7.59 (d, *J* = 8.0 Hz, 2H), 7.40 (d, *J* = 8.0 Hz, 2H), 4.44 (d, *J* = 6.8 Hz, 1H), 2.03 (brs, 1H), 1.92 – 1.86 (m, 1H), 1.79 – 1.73 (m, 1H), 1.72 – 1.56 (m, 3H), 1.42 – 1.36 (m, 1H), 1.25 – 1.08 (m, 3H), 1.07 – 1.00 (m, 1H), 0.99 – 0.92 (m, 1H).

^{13}C NMR (151 MHz, Chloroform-*d*) δ 147.64, 129.66 (q, *J* = 32.2 Hz), 127.03, 125.20 (q, *J* = 3.8 Hz), 124.3 (q, *J* = 272.0 Hz), 78.75, 45.15, 29.34, 28.49, 26.43, 26.16, 26.06.

^{19}F NMR (565 MHz, Chloroform-*d*) δ -62.41.

HR-MS *m/z* calcd. for $C_{14}H_{18}F_3O^+$ $[M+H]^+$: 259.1304, found: 259.1303.

Cyclohexyl(4-(trifluoromethoxy)phenyl)methanol (20)

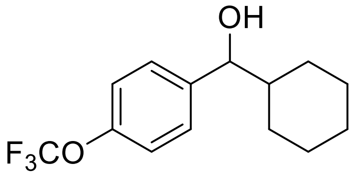

Prepared following the general procedure using 4-(trifluoromethoxy)benzaldehyde (0.3 mmol, 57.0 mg, 1.0 equiv), cyclohexane (9.0 mmol, 972 uL, 30 equiv), methylboronic acid (0.3 mmol, 18.0 mg, 1.0 equiv), copper(II) chloride (0.045 mmol, 6.1 mg, 15 mol%), chromium(II) chloride (0.03 mmol, 3.7 mg, 10 mol%), acetonitrile (1.0 mL) and dichloroethane (1.0 mL). After 72 hours of irradiation, this compound was purified by column chromatography [EtOAc: Petroleum ether = 1:30 v/v] to give the product as a colorless oil (63.1 mg, 77%).

^{1}H NMR (600 MHz, Chloroform-*d*) δ 7.33 – 7.29 (m, 2H), 7.20 – 7.15 (m, 2H), 4.38 (d, *J* = 7.0 Hz, 1H), 2.01 (brs, 1H), 1.96 – 1.90 (m, 1H), 1.79 – 1.73 (m, 1H), 1.71 – 1.62 (m, 2H), 1.61 – 1.53 (m, 1H), 1.40 – 1.34 (m, 1H), 1.25 – 1.08 (m, 3H), 1.06 – 0.99 (m, 1H), 0.96 – 0.88 (m, 1H).

^{13}C NMR (151 MHz, Chloroform-*d*) δ 148.5 (d, *J* = 2.1 Hz), 142.4, 128.1, 120.8, 120.6 (q, *J* = 256.8 Hz), 78.7, 45.1, 29.3, 28.7, 26.5, 26.2, 26.1.

^{19}F NMR (565 MHz, Chloroform-*d*) δ -57.88.

HR-MS (ESI) *m/z* calcd. for $C_{14}H_{18}F_3O_2^+$ $[M+H]^+$: 275.1253, found: 275.1252.

1-(4-(Cyclohexyl(hydroxy)methyl)phenyl)ethan-1-one (21)[6]

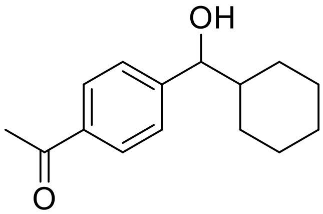

Prepared following the general procedure using methyl 4-acetylbenzaldehyde (0.3 mmol, 43.8 mg, 1.0 equiv), cyclohexane (9.0 mmol, 972 uL, 30 equiv), methylboronic acid (0.3 mmol, 18.0 mg, 1.0 equiv), copper(II) chloride (0.045 mmol, 6.1 mg, 15 mol%), chromium(II) chloride (0.03 mmol, 3.7 mg, 10 mol%), acetonitrile (1.0 mL) and dichloroethane (1.0 mL). After 72 hours of irradiation, this compound was purified by column chromatography [EtOAc: Petroleum ether = 1:8 v/v] to give the product as white solid (42.8 mg, 62%).

^{1}H NMR (600 MHz, Chloroform-*d*) δ 7.92 – 7.86 (m, 2H), 7.40 – 7.33 (m, 2H), 4.44 (d, *J* = 6.7 Hz, 1H), 2.57 (s, 3H), 2.29 (brs, 1H), 1.92 – 1.84 (m, 1H), 1.78 – 1.70 (m, 1H), 1.69 – 1.55 (m, 3H), 1.42 – 1.35 (m, 1H), 1.23 – 1.07 (m, 3H), 1.07 – 0.99 (m, 1H), 0.95 (qd, *J* = 12.4, 3.7 Hz, 1H).

^{13}C NMR (151 MHz, Chloroform-*d*) δ 198.1, 149.2, 136.3, 128.3, 126.9, 78.8, 45.1, 29.4, 28.5,

26.7, 26.4, 26.2, 26.1.

4-(Cyclohexyl(hydroxy)methyl)benzonitrile (22)[6]

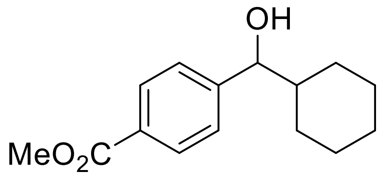

Prepared following the general procedure using methyl 4-formylbenzoate (0.3 mmol, 39.3 mg, 1.0 equiv), cyclohexane (9.0 mmol, 972 uL, 30 equiv), methylboronic acid (0.3 mmol, 18.0 mg, 1.0 equiv), copper(II) chloride (0.045 mmol, 6.1 mg, 15 mol%), chromium(II) chloride (0.03 mmol, 3.7 mg, 10 mol%), acetonitrile (1.0 mL) and dichloroethane (1.0 mL). After 72 hours of irradiation, this compound was purified by column chromatography [EtOAc: Petroleum ether = 1:8 v/v] to give the product as white solid (50.5 mg, 68%).

^{1}H NMR (600 MHz, Chloroform-*d*) δ 7.98 (d, *J* = 8.2 Hz, 2H), 7.35 (d, *J* = 8.2 Hz, 2H), 4.43 (d, *J* = 6.8 Hz, 1H), 3.89 (s, 3H), 2.13 (brs, 1H), 1.91 – 1.85 (m, 1H), 1.78 – 1.71 (m, 1H), 1.69 – 1.56 (m, 3H), 1.40 – 1.35 (m, 1H), 1.23 – 1.06 (m, 3H), 1.06 – 0.98 (m, 1H), 0.94 (qd, *J* = 12.2, 3.6 Hz, 1H).

^{13}C NMR (151 MHz, Chloroform-*d*) δ 167.2, 149.0, 129.5, 129.2, 126.7, 78.8, 52.2, 45.1, 29.3, 28.5, 26.4, 26.1, 26.1.

Methyl 4-(cyclooctyl(hydroxy)methyl)benzoate (23)

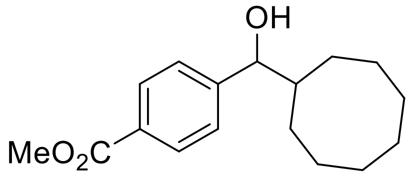

Prepared following the general procedure using methyl 4-formylbenzoate (0.3 mmol, 39.3 mg, 1.0 equiv), cyclooctane (3.0 mmol, 404 uL, 10 equiv), methylboronic acid (0.3 mmol, 18.0 mg, 1.0 equiv), copper(II) chloride (0.045 mmol, 6.1 mg, 15 mol%), chromium(II) chloride (0.03 mmol, 3.7 mg, 10 mol%), acetonitrile (1.0 mL) and dichloroethane (1.0 mL). After 72 hours of irradiation, this compound was purified by column chromatography [EtOAc: Petroleum ether = 1:8 v/v] to give the product as a white solid (55.4 mg, 67%).

^{1}H NMR (400 MHz, Chloroform-*d*) δ 7.96 – 7.85 (m, 2H), 7.36 – 7.25 (m, 2H), 4.42 (d, *J* = 6.4 Hz, 1H), 3.82 (s, 3H), 2.07 (brs, 1H), 1.89 – 1.77 (m, 1H), 1.74 – 1.65 (m, 1H), 1.63 – 1.14 (m, 13H).

^{13}C NMR (101 MHz, Chloroform-*d*) δ 167.2, 149.4, 129.5, 129.0, 126.7, 79.1, 52.1, 44.4, 29.8, 27.4, 26.9, 26.7, 26.6, 25.9, 25.6.

HR-MS (ESI) *m/z* calcd. for $C_{14}H_{20}NaO_3^+$ $[M+Na]^+$: 259.1305, found: 259.1304.

4-(Cyclohexyl(hydroxy)methyl)benzonitrile (24)[7]

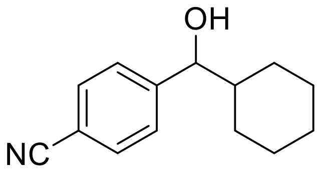

Prepared following the general procedure using 4-cyanobenzaldehyde (0.3 mmol, 39.3 mg, 1.0 equiv), cyclohexane (9.0 mmol, 972 uL, 30 equiv), methylboronic acid (0.3 mmol, 18.0 mg, 1.0 equiv), copper(II) chloride (0.045 mmol, 6.1 mg, 15 mol%), chromium(II) chloride (0.03 mmol, 3.7 mg, 10 mol%), acetonitrile (1.0 mL) and dichloroethane (1.0 mL). After 72 hours of irradiation, this compound was purified by column chromatography [EtOAc: Petroleum ether = 1:6 v/v] to give the product as a white solid (41.0 mg, 64%).

^{1}H NMR (600 MHz, Chloroform-*d*) δ 7.60 (d, *J* = 8.2 Hz, 2H), 7.40 (d, *J* = 8.2 Hz, 2H), 4.45 (d, *J* = 6.4 Hz, 1H), 2.12 (brs, 1H), 1.84 – 1.79 (m, 1H), 1.77 – 1.72 (m, 1H), 1.71 – 1.66 (m, 1H), 1.65 – 1.61 (m, 1H), 1.60 – 1.54 (m, 1H), 1.39 (m, 1H), 1.20 – 1.07 (m, 3H), 1.05 – 0.92 (m, 2H).

^{13}C NMR (151 MHz, Chloroform-*d*) δ 149.1, 132.0, 127.4, 119.0, 111.0, 78.5, 45.1, 29.3, 28.2, 26.3, 26.1, 26.0.

4-(Cyclooctyl(hydroxy)methyl)benzonitrile (25)

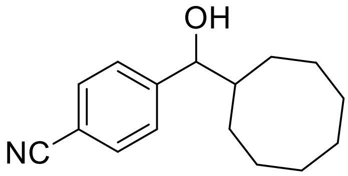

Prepared following the general procedure using 4-cyanobenzaldehyde (0.3 mmol, 39.3 mg, 1.0 equiv), cyclooctane (3.0 mmol, 404 uL, 10 equiv), methylboronic acid (0.3 mmol, 18.0 mg, 1.0 equiv), copper(II) chloride (0.045 mmol, 6.1 mg, 15 mol%), chromium(II) chloride (0.03 mmol, 3.7 mg, 10 mol%), acetonitrile (1.0 mL) and dichloroethane (1.0 mL). After 48 hours of irradiation, this compound was purified by column chromatography [EtOAc: Petroleum ether = 1:6 v/v] to give the product as a colorless oil (45.9 mg, 63%).

^{1}H NMR (600 MHz, Chloroform-*d*) δ 7.60 (d, *J* = 8.0 Hz, 2H), 7.43 (d, *J* = 8.0 Hz, 2H), 4.52 (d, *J* = 6.0 Hz, 1H), 2.06 (brs, 1H), 1.91 – 1.84 (m, 1H), 1.72 – 1.59 (m, 3H), 1.59 – 1.50 (m, 3H), 1.49 –1.41 (m, 4H), 1.40 – 1.32 (m, 3H), 1.32 1.24 (m, 1H).

^{13}C NMR (151 MHz, Chloroform-*d*) δ 149.5, 132.1, 127.4, 119.0, 110.9, 78.8, 44.4, 30.0, 27.2, 26.89, 26.6, 26.6, 25.9, 25.6.

HR-MS (ESI) *m/z* calcd. for $C_{16}H_{21}NNaO^+$ $[M+Na]^+$: 266.1515, found: 266.1517.

Cyclooctyl(4-(methylsulfonyl)phenyl)methanol (26)

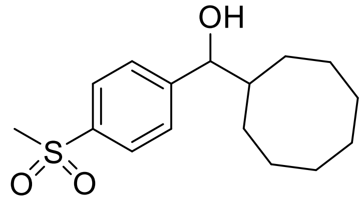

Prepared following the general procedure using 4-methylsulphonyl benzaldehyde (0.3 mmol, 55.3 mg, 1.0 equiv), cyclooctane (3.0 mmol, 404 uL, 10 equiv), methylboronic acid (0.3 mmol, 18.0 mg, 1.0 equiv), copper(II) chloride (0.045 mmol, 6.1 mg, 15 mol%), chromium(II) chloride (0.03 mmol, 3.7 mg, 10 mol%), acetonitrile (1.0 mL) and dichloroethane (1.0 mL). After 48 hours of irradiation, this compound was purified by column chromatography [EtOAc: Petroleum ether = 1:3 v/v] to give the product as a colorless oil (52.3 mg, 59%).

^{1}H NMR (600 MHz, Chloroform-*d*) δ 7.81 – 7.75 (m, 2H), 7.48 – 7.41 (m, 2H), 4.50 (d, *J* = 6.0 Hz, 1H), 2.97 (s, 3H), 2.18 (s, 1H), 1.87 – 1.79 (m, 1H), 1.68 – 1.54 (m, 3H), 1.52 – 1.44 (m, 3H), 1.43 – 1.35 (m, 4H), 1.35 – 1.26 (m, 3H), 1.26 – 1.16 (m, 1H).

^{13}C NMR (151 MHz, Chloroform-*d*) δ 150.6, 139.1, 127.6, 127.2, 78.7, 44.6, 44.5, 30.0, 27.2, 26.9, 26.6, 26.6, 25.9, 25.6.

HR-MS (ESI) *m/z* calcd. for $C_{16}H_{24}NaO_3S^+$ $[M+Na]^+$: 319.1338, found: 319.1346.

Cyclohexyl(3-fluorophenyl)methanol (27)[8]

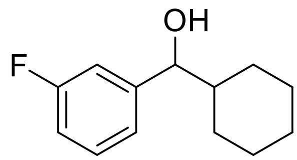

Prepared following the general procedure using 3-fluorobenzaldehyde (0.3 mmol, 37.2 mg, 1.0 equiv), cyclohexane (9.0 mmol, 972 uL, 30 equiv), methylboronic acid (0.3 mmol, 18.0 mg, 1.0 equiv), copper(II) chloride (0.045 mmol, 6.1 mg, 15 mol%), chromium(II) chloride (0.03 mmol, 3.7 mg, 10 mol%), acetonitrile (1.0 mL) and dichloroethane (1.0 mL). After 72 hours of irradiation, this compound was purified by column chromatography [EtOAc: Petroleum ether = 1:30 v/v] to give the product as a colorless oil (41.1 mg, 66%).

^{1}H NMR (600 MHz, Chloroform-*d*) δ 7.23 – 7.16 (m, 1H), 6.99 – 6.92 (m, 2H), 6.90 – 6.83 (m, 1H), 4.29 (d, *J* = 6.9 Hz, 1H), 1.90 (brs, 1H), 1.86 – 1.80 (m, 1H), 1.71 – 1.65 (m, 1H), 1.63 – 1.54 (m, 2H), 1.50 (tdd, *J* = 11.8, 6.8, 3.4 Hz, 1H), 1.34 – 1.28 (m, 1H), 1.17 – 0.99 (m, 3H), 0.95 (qd, *J* = 12.3, 3.6 Hz, 1H), 0.87 (qd, *J* = 12.3, 3.6 Hz, 1H).

^{13}C NMR (151 MHz, Chloroform-*d*) δ 163.0 (d, *J* = 245.9 Hz), 146.4 (d, *J* = 6.6 Hz), 129.7 (d, *J* = 8.2 Hz), 122.4 (d, *J* = 2.9 Hz), 114.3 (d, *J* = 21.2 Hz), 113.6 (d, *J* = 21.4 Hz), 78.8 (d, *J* = 1.9 Hz), 45.1, 29.4, 28.6, 26.5, 26.2, 26.1.

^{19}F NMR (565 MHz, Chloroform-*d*) δ -113.27 – -113.34 (m).

3-(Cyclohexyl(hydroxy)methyl)benzonitrile (28)[9]

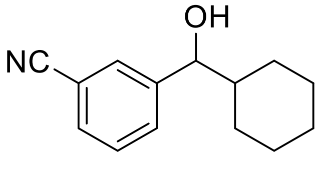

Prepared following the general procedure using 3-cyanobenzaldehyde (0.3 mmol, 39.3 mg, 1.0 equiv), cyclohexane (9.0 mmol, 972 uL, 30 equiv), methylboronic acid (0.3 mmol, 18.0 mg, 1.0 equiv), copper(II) chloride (0.045 mmol, 6.1 mg, 15 mol%), chromium(II) chloride (0.03 mmol, 3.7 mg, 10 mol%), acetonitrile (1.0 mL) and dichloroethane (1.0 mL). After 72 hours of irradiation, this compound was purified by column chromatography [EtOAc: Petroleum ether = 1:8 v/v] to give the product as a white solid (30.2 mg, 47%).

^{1}H NMR (600 MHz, Chloroform-*d*) δ 7.61 – 7.58 (m, 1H), 7.56 – 7.51 (m, 2H), 7.43 (t, *J* = 7.7 Hz, 1H), 4.44 (d, *J* = 6.5 Hz, 1H), 1.98 (brs, 1H), 1.88 – 1.81 (m, 1H), 1.79 – 1.73 (m, 1H), 1.72 – 1.67 (m, 1H), 1.67 – 1.62 (m, 1H), 1.61 – 1.54 (m, 1H), 1.43 – 1.37 (m, 1H), 1.24 – 1.07 (m, 3H), 1.06 – 0.91 (m, 2H).

^{13}C NMR (101 MHz, Chloroform-*d*) δ 145.1, 131.2, 131.1, 130.4, 129.0, 119.1, 112.3, 78.3, 45.2, 29.3, 28.3, 26.4, 26.1, 26.0.

Cyclohexyl(o-tolyl)methanol (29)[8]

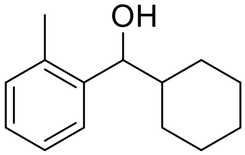

Prepared following the general procedure using 2-methylbenzaldehyde (0.3 mmol, 36.0 mg, 1.0 equiv), cyclohexane (9.0 mmol, 972 uL, 30 equiv), methylboronic acid (0.3 mmol, 18.0 mg, 1.0 equiv), copper(II) chloride (0.045 mmol, 6.1 mg, 15 mol%), chromium(II) chloride (0.03 mmol, 3.7 mg, 10 mol%), acetonitrile (1.0 mL) and dichloroethane (1.0 mL). After 72 hours of irradiation, this compound was purified by column chromatography [EtOAc: Petroleum ether = 1:30 v/v] to give the product as a white solid (31.6 mg, 52%).

^{1}H NMR (600 MHz, Chloroform-*d*) δ 7.41 (dd, *J* = 7.8, 1.4 Hz, 1H), 7.22 (td, *J* = 7.4, 1.6 Hz, 1H), 7.18 – 7.11 (m, 2H), 4.65 (d, *J* = 7.2 Hz, 1H), 2.34 (s, 3H), 2.05 – 2.00 (m, 1H), 1.81 – 1.76 (m, 1H), 1.74 (brs, 1H), 1.71 – 1.60 (m, 3H), 1.41 – 1.35 (m, 1H), 1.24 – 1.10 (m, 4H), 1.10 – 1.01 (m, 1H).

^{13}C NMR (151 MHz, Chloroform-*d*) δ 141.1, 134.2, 129.4, 126.1, 125.4, 125.2, 74.2, 43.6, 28.7, 27.7, 25.6, 25.4, 25.2, 18.6.

Cyclohexyl(2-fluorophenyl)methanol (30)

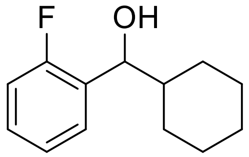

Prepared following the general procedure using 2-fluorobenzaldehyde (0.3 mmol, 37.2 mg, 1.0 equiv), cyclohexane (9.0 mmol, 972 uL, 30 equiv), methylboronic acid (0.3 mmol, 18.0 mg, 1.0 equiv), copper(II) chloride (0.045 mmol, 6.1 mg, 15 mol%), chromium(II) chloride (0.03 mmol, 3.7 mg, 10 mol%), acetonitrile (1.0 mL) and dichloroethane (1.0 mL). After 72 hours of irradiation, this compound was purified by column chromatography [EtOAc: Petroleum ether = 1:30 v/v] to give the product as a colorless oil (44.4 mg, 71%).

^{1}H NMR (600 MHz, Chloroform-*d*) δ 7.40 (td, *J* = 7.5, 1.8 Hz, 1H), 7.26 – 7.20 (m, 1H), 7.14 (td, *J* = 7.5, 1.2 Hz, 1H), 7.04 – 6.97 (m, 1H), 4.72 (d, *J* = 7.2 Hz, 1H), 2.01 – 1.92 (m, 2H), 1.80 – 1.73 (m, 1H), 1.71 – 1.61 (m, 3H), 1.44 – 1.37 (m, 1H), 1.26 – 1.11 (m, 3H), 1.11 – 0.98 (m, 2H).

^{13}C NMR (151 MHz, Chloroform-*d*) δ 160.2 (d, *J* = 245.1 Hz), 130.7 (d, *J* = 13.1 Hz), 128.8 (d, *J* = 8.2 Hz), 128.3 (d, *J* = 4.8 Hz), 124.2 (d, *J* = 3.5 Hz), 115.3 (d, *J* = 22.3 Hz), 73.0 (d, *J* = 1.8 Hz), 44.6, 29.2, 28.8, 26.5, 26.2, 26.1.

^{19}F NMR (565 MHz, Chloroform-*d*) δ -118.66 – -118.77 (m).

HR-MS (ESI) *m/z* calcd. for C13H18FO^{+} [M+H]$^{+}$: 209.1336, found: 209.1333.

Cyclohexyl(2-fluorophenyl)methanol (31)[10]

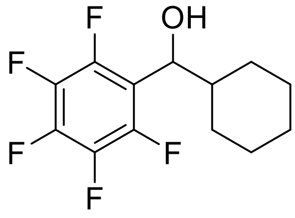

Prepared following the general procedure using pentafluorobenzaldehyde (0.3 mmol, 58.8 mg, 1.0 equiv), cyclohexane (9.0 mmol, 972 uL, 30 equiv), methylboronic acid (0.3 mmol, 18.0 mg, 1.0 equiv), copper(II) chloride (0.045 mmol, 6.1 mg, 15 mol%), chromium(II) chloride (0.03 mmol, 3.7 mg, 10 mol%), acetonitrile (1.0 mL) and dichloroethane (1.0 mL). After 72 hours of irradiation, this compound was purified by column chromatography [EtOAc: Petroleum ether = 1:30 v/v] to give the product as a white solid (38.1 mg, 45%).

^{1}H NMR (600 MHz, Chloroform-*d*) δ 4.69 (d, *J* = 9.2 Hz, 1H), 2.22 (brs, 1H), 2.19 – 2.13 (m, 1H), 1.87 – 1.78 (m, 2H), 1.72 – 1.63 (m, 2H), 1.30 – 1.12 (m, 4H), 1.06 (qd, *J* = 12.4, 3.6 Hz, 1H), 0.94 (qd, *J* = 12.2, 3.5 Hz, 1H).

^{13}C NMR (151 MHz, Chloroform-*d*) δ 144.9 (dm, *J* = 247.1 Hz), 140.6 (dm, 253.8 Hz), 137.6 (dm, 251.7 Hz), 116.64 (tm, *J* = 16.4 Hz), 71.58, 43.58, 29.89, 29.10, 26.24, 25.78, 25.65.

^{19}F NMR (565 MHz, Chloroform-*d*) δ -142.84 (dd, *J* = 22.9, 8.1 Hz, 2F), -155.47 (t, *J* = 20.7 Hz,

1F), -161.93 – -162.09 (m, 2F).

2,6-diisopropylphenyl 4-(cyclooctyl(hydroxy)methyl)benzoate (32)

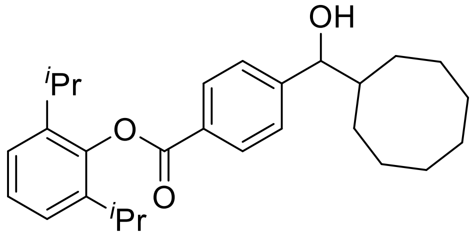

Prepared following the general procedure using 2,6-diisopropylphenyl 4-formylbenzoate (0.3 mmol, 93.1 mg, 1.0 equiv), cyclooctane (3.0 mmol, 404 uL, 10 equiv), methylboronic acid (0.3 mmol, 18.0 mg, 1.0 equiv), copper(II) chloride (0.045 mmol, 6.1 mg, 15 mol%), chromium(II) chloride (0.03 mmol, 3.7 mg, 10 mol%), acetonitrile (1.0 mL) and dichloroethane (1.0 mL). After 72 hours of irradiation, this compound was purified by column chromatography [EtOAc: Petroleum ether = 1:20 v/v] to give the product as faint yellow oil (67.8 mg, 54%).

^{1}H NMR (600 MHz, Chloroform-*d*) δ 8.15 – 8.10 (m, 2H), 7.43 – 7.39 (m, 2H), 7.19 – 7.15 (m, 1H), 7.14 – 7.10 (m, 2H), 4.47 (d, *J* = 6.4 Hz, 1H), 2.90 (hept, *J* = 6.9 Hz, 2H), 1.93 (brs, 1H), 1.91 – 1.85 (m, 1H), 1.77 – 1.68 (m, 1H), 1.66 – 1.55 (m, 2H), 1.54 – 1.46 (m, 3H), 1.46 – 1.38 (m, 4H), 1.37 – 1.30 (m, 3H), 1.28 – 1.21 (m, 1H), 1.12 (d, *J* = 7.0 Hz, 12H).

^{13}C NMR (151 MHz, Chloroform-*d*) δ 165.3, 150.1, 146.0, 140.7, 130.3, 128.4, 127.1, 126.7, 124.1, 79.3, 44.4, 30.1, 27.7, 27.5, 27.0, 26.8, 26.7, 26.0, 25.7, 24.1, 22.8.

HR-MS (ESI) *m/z* calcd. for $C_{28}H_{39}O_3^+$ $[M+H]^+$: 423.2894, found: 423.2899.

Cyclohexyl(4-methoxyphenyl)methanol (33)[11]

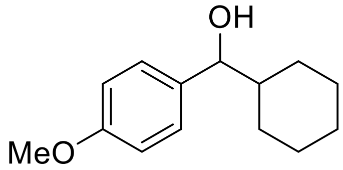

In glove box, to an oven-dried 10 mL Schlenk tube equipped with a Teflon-coated magnetic stirring bar were added $CuCl_2$ (0.045 mmol, 6.1 mg, 15 mol%), $CrCl_2$ (0.03 mmol, 3.7 mg, 10 mol%), CH_3CN (1.0 mL) and DCE (0.5 mL). Then take out of glove box, add *p*-anisaldehyde (0.3 mmol, 40.8 mg, 1.0 equiv), TESCl (0.3 mmol, 45.2 mg, 1.0 equiv), cyclohexane (9.0 mmol, 972 ul, 30 equiv) and DCE (0.5 mL) under positive argon pressure. The reaction mixture was stirred under purple light irradiation (20 W, λ_{max} = 390 nm) for 72 h. In this case, the light source was placed ~ 0.5 cm from the reaction vessel. This compound was purified by column chromatography [EtOAc: Petroleum ether = 1:20 v/v] to give the product as a white solid (26.4 mg, 40%).

^{1}H NMR (600 MHz, Chloroform-*d*) δ 7.24 – 7.19 (m, 2H), 6.90 – 6.85 (m, 2H), 4.30 (d, *J* = 7.4

Hz, 1H), 3.80 (s, 3H), 2.05 – 1.97 (m, 1H), 1.81 – 1.73 (m, 2H), 1.70 – 1.54 (m, 3H), 1.39 – 1.32 (m, 1H), 1.28 – 1.18 (m, 1H), 1.18 (m, 2H), 1.06 – 0.98 (m, 1H), 0.93 – 0.83 (m, 1H).

^{13}C NMR (151 MHz, Chloroform-*d*) δ 159.1, 136.0, 127.9, 113.7, 79.2, 55.4, 45.1, 29.4, 29.2, 26.6, 26.2, 26.1.

2-(Cyclohexyl(4-methoxyphenyl)methyl)-1,3,5-trimethoxybenzene (34)

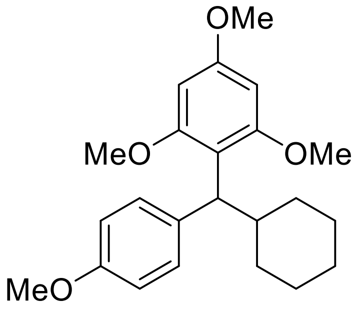

Prepared following the general procedure A using *p*-anisaldehyde (0.3 mmol, 40.8 mg, 1.0 equiv), cyclohexane (9.0 mmol, 972 uL, 30 equiv), 1,3,5-trimethoxybenzene (0.3 mmol, 50.5 mg, 1.0 equiv), copper(II) chloride (0.045 mmol, 6.1 mg, 15 mol%), chromium(II) chloride (0.03 mmol, 3.7 mg, 10 mol%), acetonitrile (1.0 mL) and dichloroethane (1.0 mL). After 72 hours of irradiation, this compound was purified by column chromatography [EtOAc: Petroleum ether = 1:30 v/v] to give the product as a colorless oil (77.6 mg, 70%).

^{1}H NMR (600 MHz, Chloroform-*d*) δ 7.41 – 7.34 (m, 2H), 6.82 – 6.74 (m, 2H), 6.11 (s, 2H), 4.16 (d, J = 11.1 Hz, 1H), 3.81 (s, 6H), 3.77 (d, J = 4.5 Hz, 6H), 2.56 (dtt, J = 11.2, 11.2, 3.4 Hz, 1H), 1.79 – 1.72 (m, 1H), 1.71 – 1.62 (m, 3H), 1.54 – 1.48 (m, 1H), 1.31 – 1.22 (m, 2H), 1.21 – 1.13 (m, 1H), 0.88 – 0.78 (m, 2H).

^{13}C NMR (151 MHz, Chloroform-*d*) δ 159.1, 157.3, 137.3, 129.9, 114.2, 113.1, 91.3, 55.8, 55.3, 55.2, 46.6, 38.1, 33.1, 32.0, 26.9, 26.8, 26.5.

HR-MS (ESI) *m/z* calcd. for $C_{23}H_{31}O_4^+$ $[M+H]^+$: 371.2217, found: 371.2212.

2-(Cyclopentyl(4-methoxyphenyl)methyl)-1,3,5-trimethoxybenzene (35)

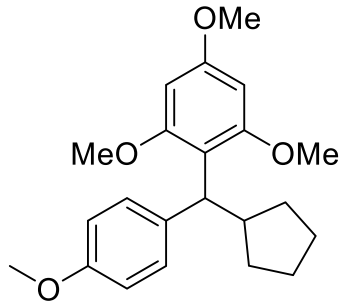

Prepared following the general procedure A using *p*-anisaldehyde (0.3 mmol, 40.8 mg, 1.0 equiv), cyclopentane (9.0 mmol, 840 uL, 30 equiv), 1,3,5-trimethoxybenzene (0.3 mmol, 50.5 mg, 1.0 equiv), copper(II) chloride (0.045 mmol, 6.1 mg, 15 mol%), chromium(II) chloride (0.03 mmol, 3.7 mg, 10 mol%), acetonitrile (1.0 mL) and dichloroethane (1.0 mL). After 72 hours of irradiation, the reaction mixture was extracted with saturated $NaHSO_3$ to remove unreacted *p*-anisaldehyde, then this compound was purified by column chromatography [EtOAc: Petroleum

ether = 1:30 v/v] to give the product as a colorless oil (54.5 mg, 51%).

^{1}H NMR (600 MHz, Chloroform-*d*) δ 7.38 – 7.31 (m, 2H), 6.80 – 6.72 (m, 2H), 6.10 (s, 2H), 4.24 (d, *J* = 11.4 Hz, 1H), 3.89 – 3.65 (m, 12H), 3.20 – 3.08 (m, 1H), 1.82 – 1.74 (m, 1H), 1.68 – 1.55 (m, 3H), 1.55 – 1.45 (m, 2H), 1.17 – 1.02 (m, 2H).

^{13}C NMR (151 MHz, Chloroform-*d*) δ 158.2, 156.2, 137.1, 128.8, 114.6, 111.9, 90.3, 54.3, 54.2, 44.8, 40.1, 32.0, 31.1, 24.8, 24.6.

HR-MS (ESI) *m/z* calcd. for $C_{22}H_{28}NaO_4^+$ $[M+Na]^+$: 379.1880, found: 379.1875.

2-(Cyclohexyl(3,4-dimethoxyphenyl)methyl)-1,3,5-trimethoxybenzene (36)

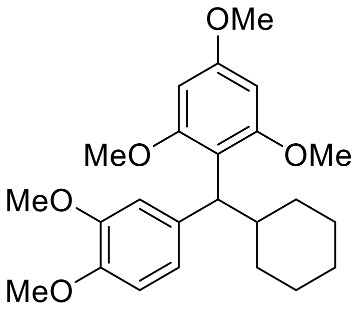

Prepared following the general procedure A using veratraldehyde (0.3 mmol, 49.9 mg, 1.0 equiv), cyclohexane (9.0 mmol, 972 uL, 30 equiv), 1,3,5-trimethoxybenzene (0.3 mmol, 50.5 mg, 1.0 equiv), copper(II) chloride (0.045 mmol, 6.1 mg, 15 mol%), chromium(II) chloride (0.03 mmol, 3.7 mg, 10 mol%), acetonitrile (1.0 mL) and dichloroethane (1.0 mL). After 72 hours of irradiation, the reaction mixture was extracted with saturated $NaHSO_3$ to remove unreacted veratraldehyde, then this compound was purified by column chromatography [EtOAc: Petroleum ether = 1:20 v/v] to give the product as a colorless oil (91.0 mg, 76%).

^{1}H NMR (400 MHz, Chloroform-*d*) δ 7.12 (d, *J* = 2.0 Hz, 1H), 6.97 (dd, *J* = 8.3, 2.0 Hz, 1H), 6.73 (d, *J* = 8.3 Hz, 1H), 6.11 (s, 2H), 4.13 (d, *J* = 11.2 Hz, 1H), 3.87 (s, 3H), 3.82 (s, 9H), 3.76 (s, 3H), 2.61 – 2.48 (m, 1H), 1.77 – 1.58 (m, 4H), 1.56 – 1.47 (m, 1H), 1.32 – 1.13 (m, 3H), 0.93 – 0.75 (m, 2H).

^{13}C NMR (101 MHz, Chloroform-*d*) δ 159.1, 158.9, 148.2, 146.7, 137.9, 121.1, 113.9, 112.5, 110.6, 91.3, 55.8, 55.7, 55.2, 47.1, 38.3, 33.0, 31.9, 26.8, 26.6, 26.4.

HR-MS (ESI) *m/z* calcd. for $C_{24}H_{32}NaO_5^+$ $[M+Na]^+$: 423.2142, found: 423.2137.

2-(Cyclohexyl(4-methoxy-3-methylphenyl)methyl)-1,3,5-trimethoxybenzene (37)

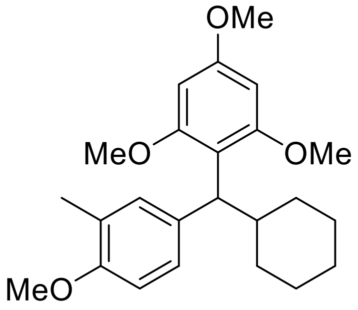

Prepared following the general procedure A using 3-methyl-4-anisaldehyde (0.3 mmol, 45.1 mg, 1.0 equiv), cyclohexane (9.0 mmol, 972 uL, 30 equiv), 1,3,5-trimethoxybenzene (0.3 mmol, 50.5 mg, 1.0 equiv), copper(II) chloride (0.045 mmol, 6.1 mg, 15 mol%), chromium(II) chloride (0.03 mmol, 3.7 mg, 10 mol%), acetonitrile (1.0 mL) and dichloroethane (1.0 mL). After 72 hours of irradiation, the reaction mixture was extracted with saturated $NaHSO_3$ to remove unreacted 3-methyl-4-anisaldehyde, then this compound was purified by column chromatography [EtOAc: Petroleum ether = 1:20 v/v] to give the product as a colorless oil (70.4 mg, 61%).

^{1}H NMR (400 MHz, Chloroform-*d*) δ 7.24 (dd, *J* = 8.3, 2.2 Hz, 1H), 7.20 (d, J = 2.2 Hz, 1H), 6.69 (d, *J* = 8.3 Hz, 1H), 6.10 (s, 2H), 4.11 (d, *J* = 11.2 Hz, 1H), 3.80 (s, 6H), 3.77 (s, 3H), 3.76 (s, 3H), 2.54 (tdd, *J* = 11.2, 7.8, 3.1 Hz, 1H), 2.18 (s, 3H), 1.78 – 1.70 (m, 1H), 1.70 1.61 (m, 3H), 1.54 – 1.46 (m, 1H), 1.30 – 1.17 (m, 3H), 0.90 – 0.79 (m, 2H).

^{13}C NMR (101 MHz, Chloroform-*d*) δ 159.1, 155.5, 136.8, 131.6, 127.0, 125.3, 114.4, 109.3, 91.4, 55.8, 55.2(9), 55.2(6), 46.6, 38.2, 33.1, 32.0, 26.9, 26.8, 26.5, 16.6.

HR-MS (ESI) *m/z* calcd. for $C_{24}H_{33}O_4^+$ $[M+H]^+$: 385.2373, found: 385.2371.

(4-(Cyclohexyl(2,4,6-trimethoxyphenyl)methyl)phenyl)(methyl)sulfane (38)

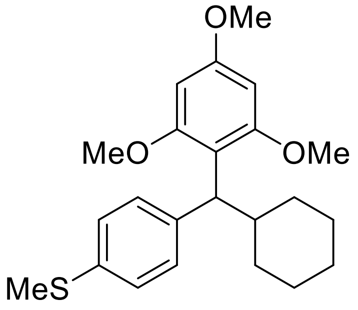

Prepared following the general procedure A using 4-(methylthio)benzaldehyde (0.3 mmol, 45.7 mg, 1.0 equiv), cyclohexane (9.0 mmol, 972 uL, 30 equiv), 1,3,5-trimethoxybenzene (0.3 mmol, 50.5 mg, 1.0 equiv), copper(II) chloride (0.045 mmol, 6.1 mg, 15 mol%), chromium(II) chloride (0.03 mmol, 3.7 mg, 10 mol%), acetonitrile (1.0 mL) and dichloroethane (1.0 mL). After 72 hours of irradiation, the reaction mixture was extracted with saturated $NaHSO_3$ to remove unreacted 4-(methylthio)benzaldehyde, then this compound was purified by column chromatography [EtOAc: Petroleum ether = 1:20 v/v] to give the product as a colorless oil (56.8 mg, 49%).

^{1}H NMR (400 MHz, Chloroform-*d*) δ 7.41 – 7.30 (m, 2H), 7.16 – 7.08 (m, 2H), 6.09 (d, *J* = 1.5

Hz, 2H), 4.15 (d, J = 11.2 Hz, 1H), 3.79 (s, 6H), 3.75 (s, 3H), 2.56 (tdd, J = 11.5, 6.7, 3.2 Hz, 1H), 2.43 (s, 3H), 1.77 – 1.60 (m, 4H), 1.53 – 1.45 (m, 1H), 1.28 – 1.14 (m, 3H), 0.88 – 0.77 (m, 2H).

^{13}C NMR (101 MHz, Chloroform-*d*) δ 159.3, 159.0, 142.4, 134.2, 129.7, 126.7, 113.6, 91.3, 55.8, 55.3, 46.9, 37.9, 33.0, 31.9, 26.9, 26.7, 26.5, 16.5.

HR-MS (ESI) *m/z* calcd. for $C_{23}H_{31}O_3S^+$ $[M+H]^+$: 387.1988, found: 387.1986.

1-(Cyclohexyl(ethoxy)methyl)-4-methoxybenzene (39)

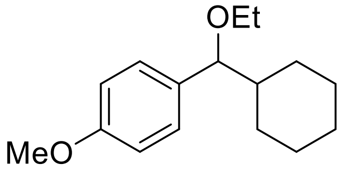

Prepared following the general procedure A using *p*-anisaldehyde (0.3 mmol, 40.8 mg, 1.0 equiv), cyclohexane (9.0 mmol, 972 uL, 30 equiv), ethanol (0.3 mmol, 13.8 mg, 1.0 equiv), copper(II) chloride (0.045 mmol, 6.1 mg, 15 mol%), chromium(II) chloride (0.03 mmol, 3.7 mg, 10 mol%), acetonitrile (1.0 mL) and dichloroethane (1.0 mL). After 72 hours of irradiation, this compound was purified by column chromatography [EtOAc: Petroleum ether = 1:150 v/v] to give the product as a colorless oil (39.6 mg, 53%).

^{1}H NMR (600 MHz, Chloroform-*d*) δ 7.19 – 7.12 (m, 2H), 6.90 – 6.81 (m, 2H), 3.83 –3.78 (m, 4H), 3.39 – 3.17 (m, 2H), 2.12 – 2.04 (m, 1H), 1.76 – 1.69 (m, 1H), 1.65 – 1.51 (m, 3H), 1.26 – 1.18 (m, 2H), 1.17 – 1.06 (m, 5H), 1.03 – 0.94 (m, 1H), 0.90 – 0.79 (m, 1H).

^{13}C NMR (151 MHz, Chloroform-*d*) δ 158.9 134.1, 128.6, 113.5, 86.7, 64.1, 55.3, 44.5, 30.1, 29.5, 26.8, 26.3, 26.2, 15.4.

HR-MS (ESI) *m/z* calcd. for $C_{16}H_{24}NaO_2^+$ $[M+Na]^+$: 271.1669, found: 271.1666.

3. References

[1] Kabalka, G. W.; Wu, Z.; Ju, Y. Alkylation of aromatic aldehydes with alkylboron chloride derivatives, *Tetrahedron* **2001**, *57*, 1663.

[2] Takahashi, R.; Hu, A.; Gao, P.; Gao, Y.; Pang, Y.; Seo, T.; Jiang, J.; Maeda, S.; Takaya, H.; Kubota, K.; Ito, H. Mechanochemical synthesis of magnesium-based carbon nucleophiles in air and their use in organic synthesis, *Nat. Commun.* **2021**, *12*, 6691.

[3] Ogata, K.; Toh, A.; Shimada, D.; Fukuzawa, S.-i. Nickel-catalyzed Diastereoselective Reductive Coupling Reaction of Norbornene with Aldehydes in the Presence of Triethylborane, *Chem. Lett.* **2012**, *41*, 157.

[4] Jiang, X.; Jiang, H.; Yang, Q.; Cheng, Y.; Lu, L.-Q.; Tunge, J. A.; Xiao, W.-J. Photoassisted Cobalt-Catalyzed Asymmetric Reductive Grignard-Type Addition of Aryl Iodides, *J. Am. Chem. Soc.* **2022**, *144*, 8347.

[5] Isbrandt, E. S.; Nasim, A.; Zhao, K.; Newman, S. G. Catalytic Aldehyde and Alcohol Arylation Reactions Facilitated by a 1,5-Diaza-3,7-diphosphacyclooctane Ligand, *J. Am. Chem. Soc.* **2021**, *143*, 14646.

[6] Yahata, K.; Sakurai, S.; Hori, S.; Yoshioka, S.; Kaneko, Y.; Hasegawa, K.; Akai, S. Coupling Reaction between Aldehydes and Non-Activated Hydrocarbons via the Reductive Radical-Polar Crossover Pathway, *Org. Lett.* **2020**, *22*, 1199.

[7] Kim, R. S.; Dinh-Nguyen, L. V.; Shimkin, K. W.; Watson, D. A. Copper-Catalyzed Propargylation of Nitroalkanes, *Org. Lett.* **2020**, *22*, 8106.

[8] Shen, Z.-L.; Yeo, Y.-L.; Loh, T.-P. Indium−Copper and Indium−Silver Mediated Barbier–Grignard-Type Alkylation Reaction of Aldehydes Using Unactivated Alkyl Halides in Water, *J. Org. Chem.* **2008**, *73*, 3922.

[9] DeBerardinis, A. M.; Turlington, M.; Pu, L. Activation of Functional Arylzincs Prepared from Aryl Iodides and Highly Enantioselective Addition to Aldehydes, *Org. Lett.* **2008**, *10*, 2709.

[10] Liu, Z.; Kole, G. K.; Budiman, Y. P.; Tian, Y.-M.; Friedrich, A.; Luo, X.; Westcott, S. A.; Radius, U.; Marder, T. B. Transition Metal Catalyst-Free, Base-Promoted 1,2-Additions of Polyfluorophenylboronates to Aldehydes and Ketones, *Angew. Chem. Int. Ed.* **2021**, *60*, 16529.

[11] Garcia, K. J.; Gilbert, M. M.; Weix, D. J. Nickel-Catalyzed Addition of Aryl Bromides to Aldehydes To Form Hindered Secondary Alcohols, *J. Am. Chem. Soc.* **2019**, *141*, 1823.

4. NMR spectra of products

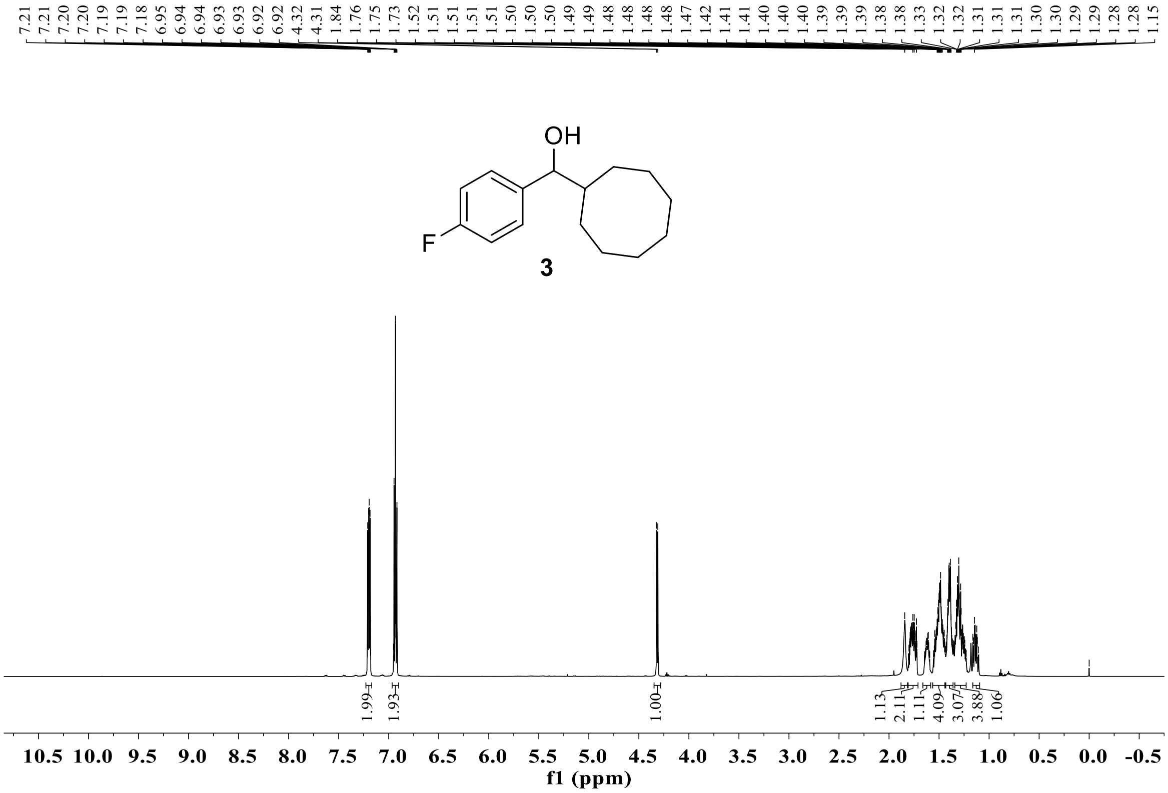

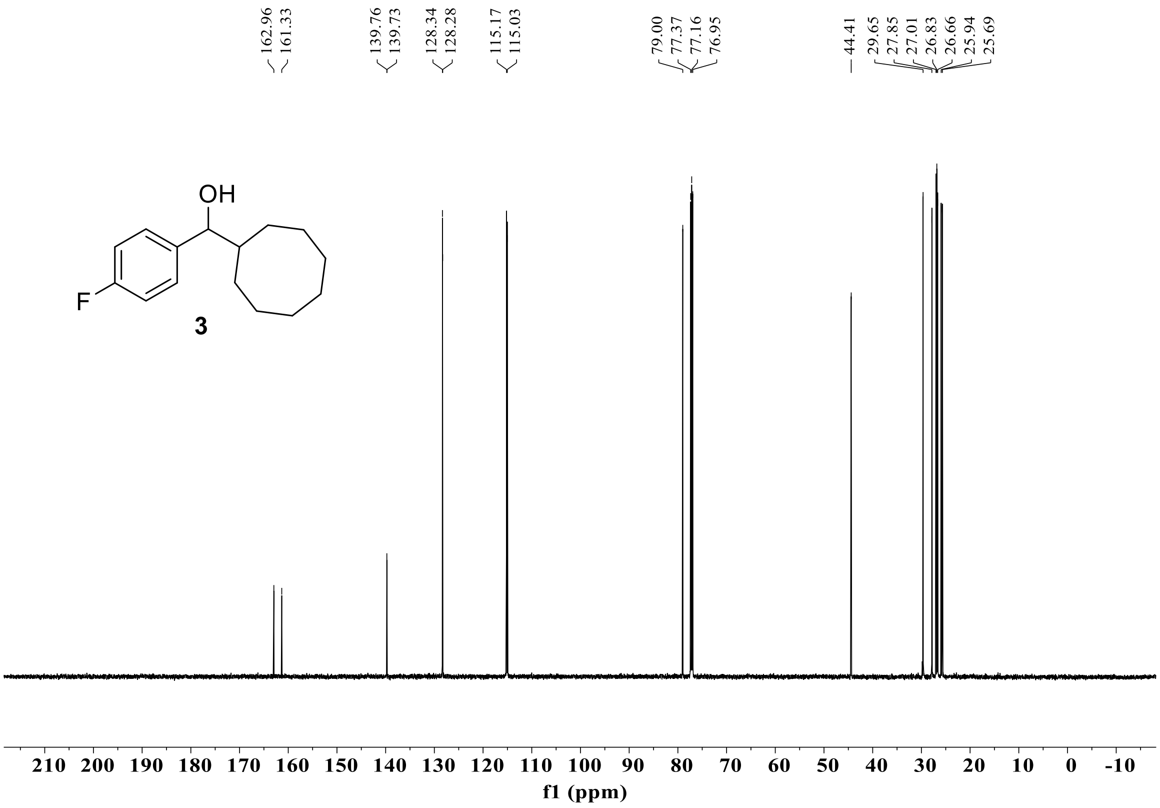

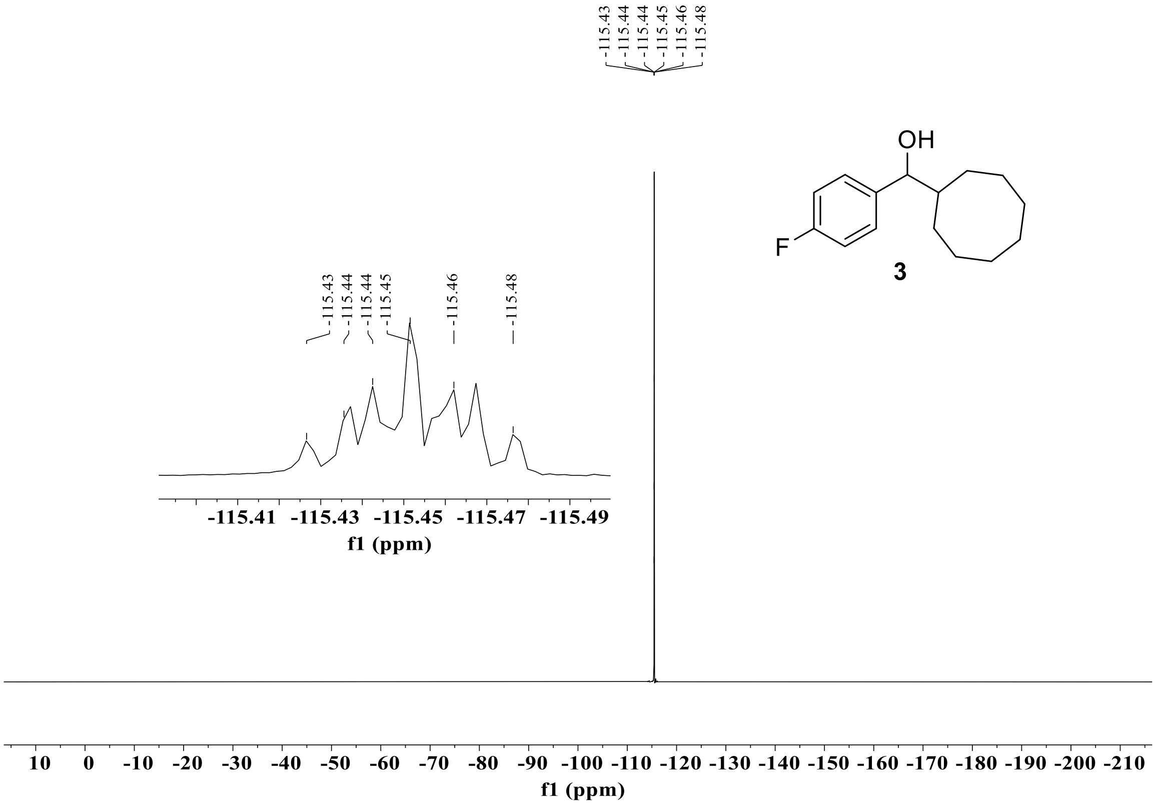
-115.43
-115.44
-115.44
-115.45
-115.46
-115.48
OH
F
3
-115.41 -115.43 -115.45 -115.47 -115.49
f1 (ppm)
10 0 -10 -20 -30 -40 -50 -60 -70 -80 -90 -100 -110 -120 -130 -140 -150 -160 -170 -180 -190 -200 -210
f1 (ppm)

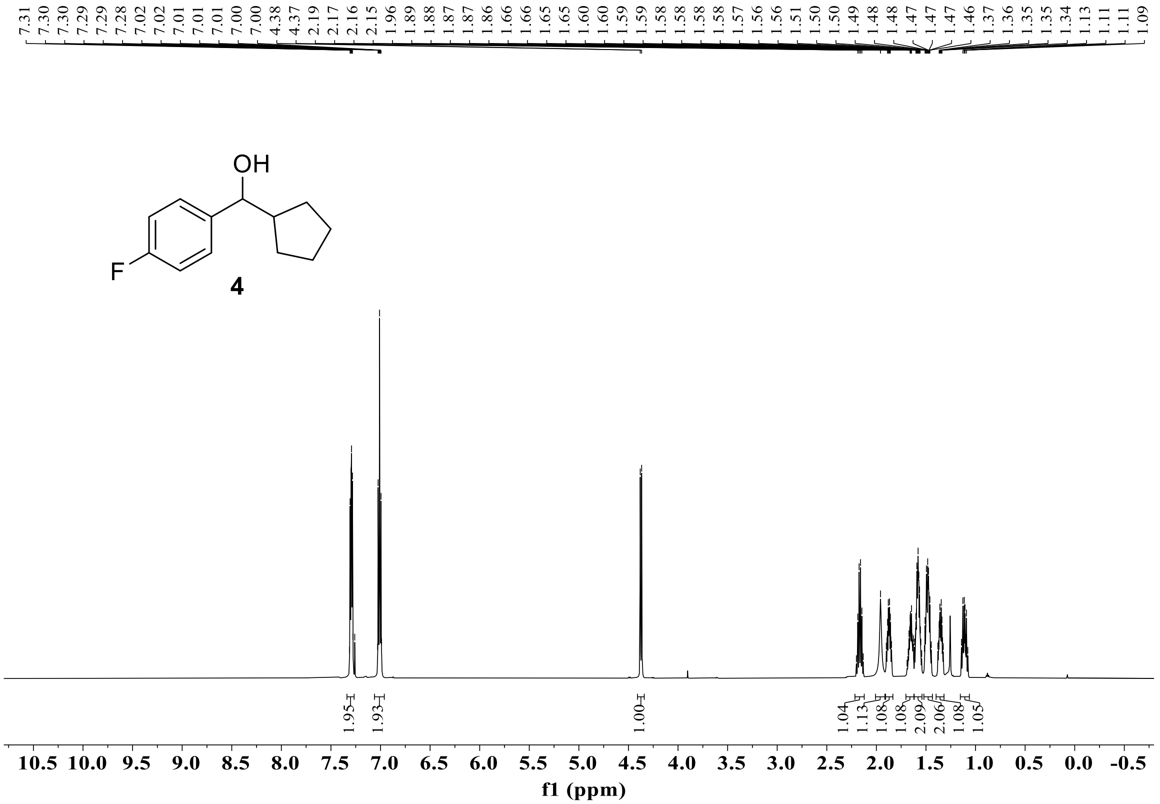

7.31 7.30 7.30 7.29 7.29 7.28 7.02 7.02 7.01 7.01 7.01 7.00 7.00 4.38 4.37 2.19 2.17 2.16 2.15 1.96 1.89 1.88 1.87 1.87 1.86 1.66 1.66 1.65 1.65 1.60 1.60 1.59 1.59 1.58 1.58 1.58 1.58 1.57 1.56 1.56 1.51 1.50 1.50 1.49 1.48 1.48 1.47 1.47 1.47 1.46 1.37 1.36 1.35 1.34 1.13 1.11 1.11 1.09
OH
F
4
1.95
1.93
1.00
1.04
1.13
1.08
1.08
2.09
2.06
1.08
1.05
10.5 10.0 9.5 9.0 8.5 8.0 7.5 7.0 6.5 6.0 5.5 5.0 4.5 4.0 3.5 3.0 2.5 2.0 1.5 1.0 0.5 0.0 -0.5
f1 (ppm)

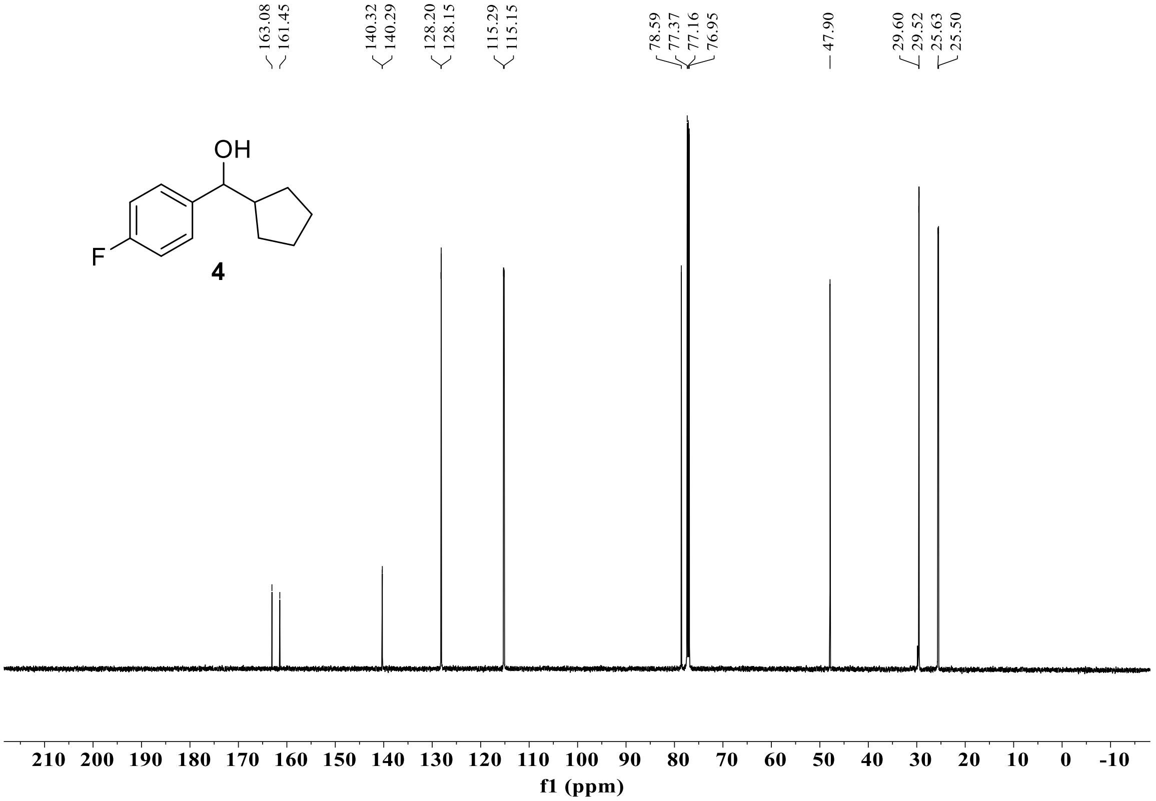

163.08 161.45
140.32 140.29
128.20 128.15
115.29 115.15
78.59 77.37 77.16 76.95
47.90
29.60 29.52 25.63 25.50
OH
F
4
210 200 190 180 170 160 150 140 130 120 110 100 90 80 70 60 50 40 30 20 10 0 -10
f1 (ppm)

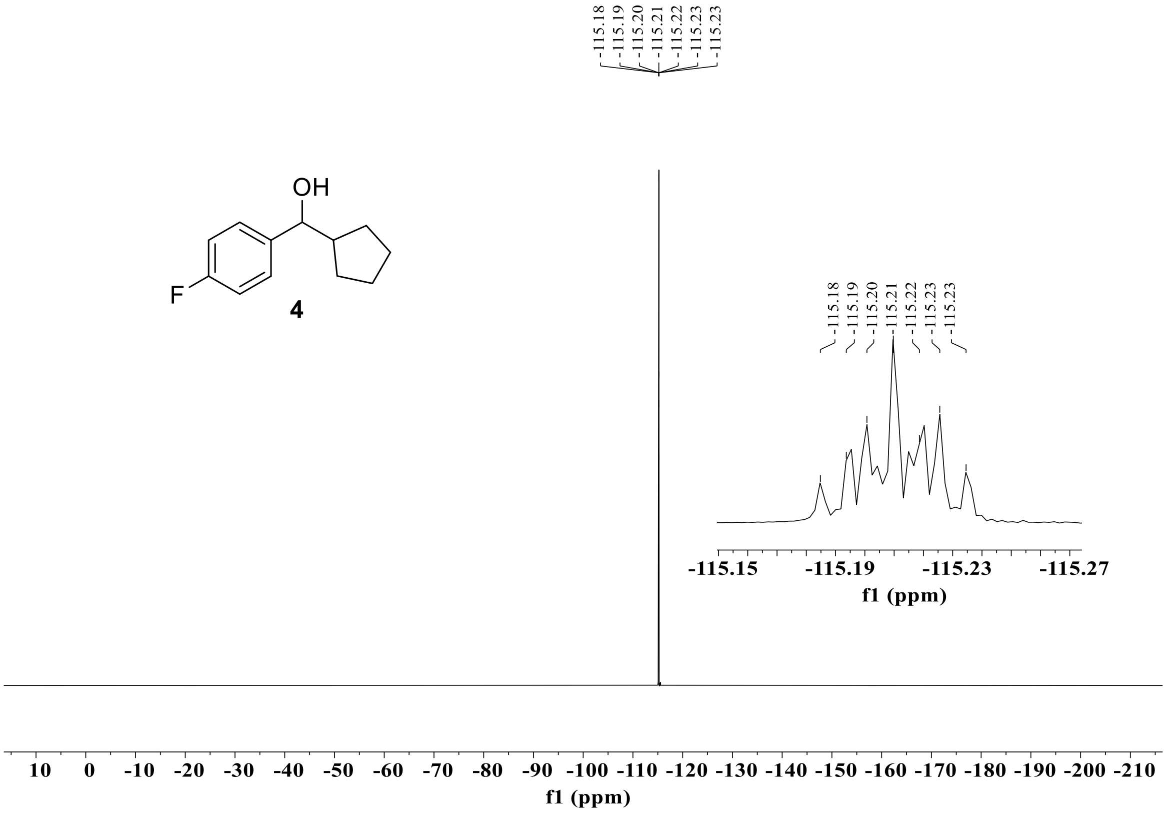

-115.18
-115.19
-115.20
-115.21
-115.22
-115.23
-115.23
OH
F
4
-115.15
-115.19
-115.23
-115.27
f1 (ppm)
10 0 -10 -20 -30 -40 -50 -60 -70 -80 -90 -100 -110 -120 -130 -140 -150 -160 -170 -180 -190 -200 -210
f1 (ppm)

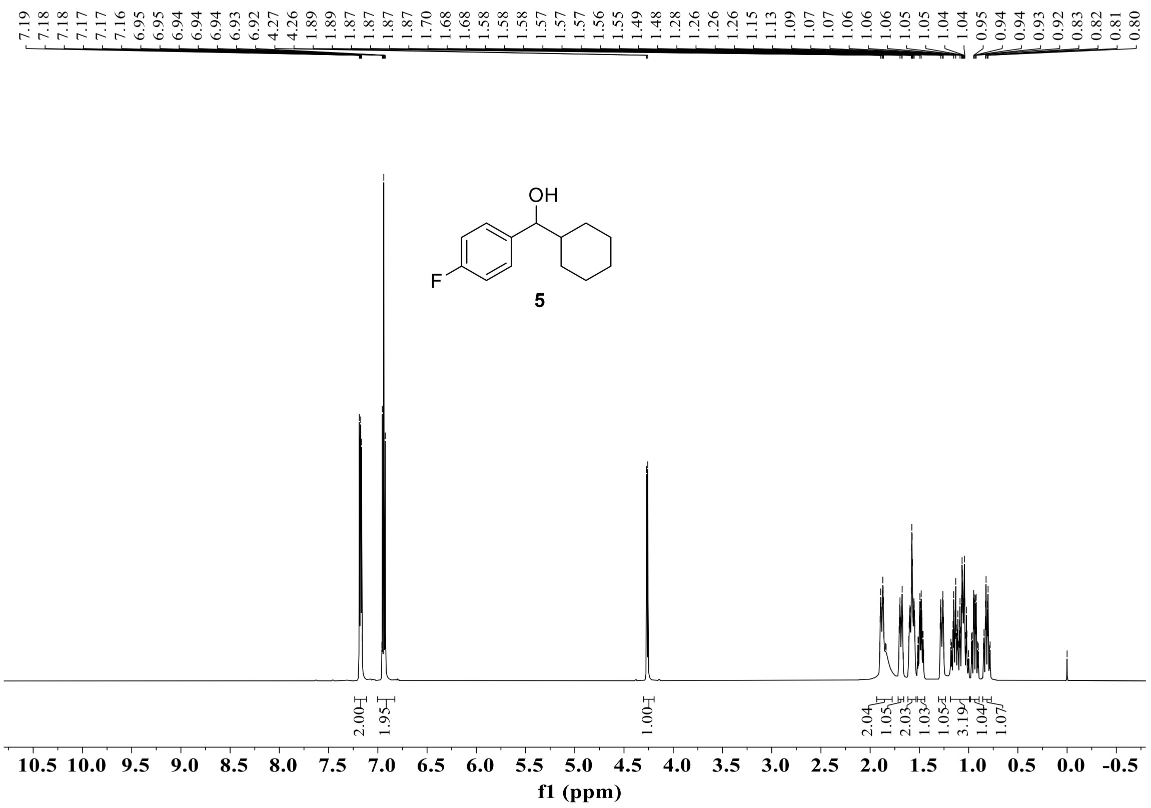

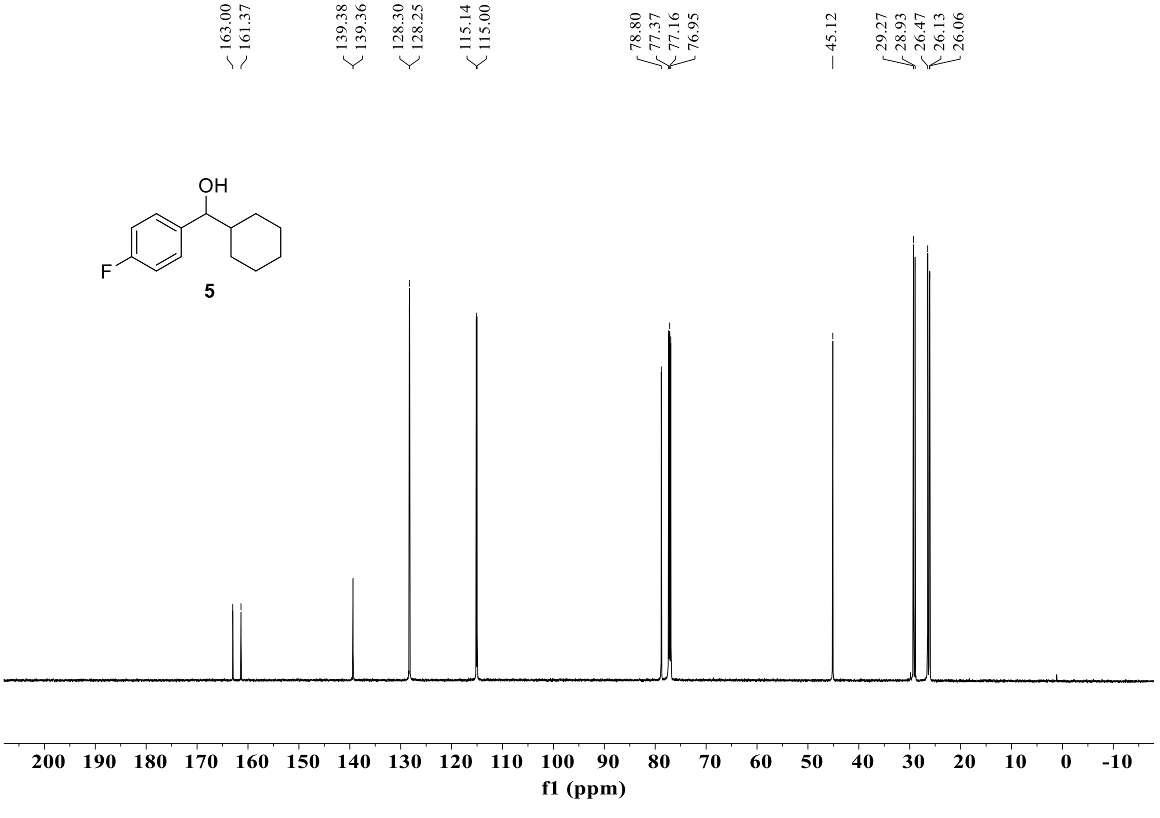

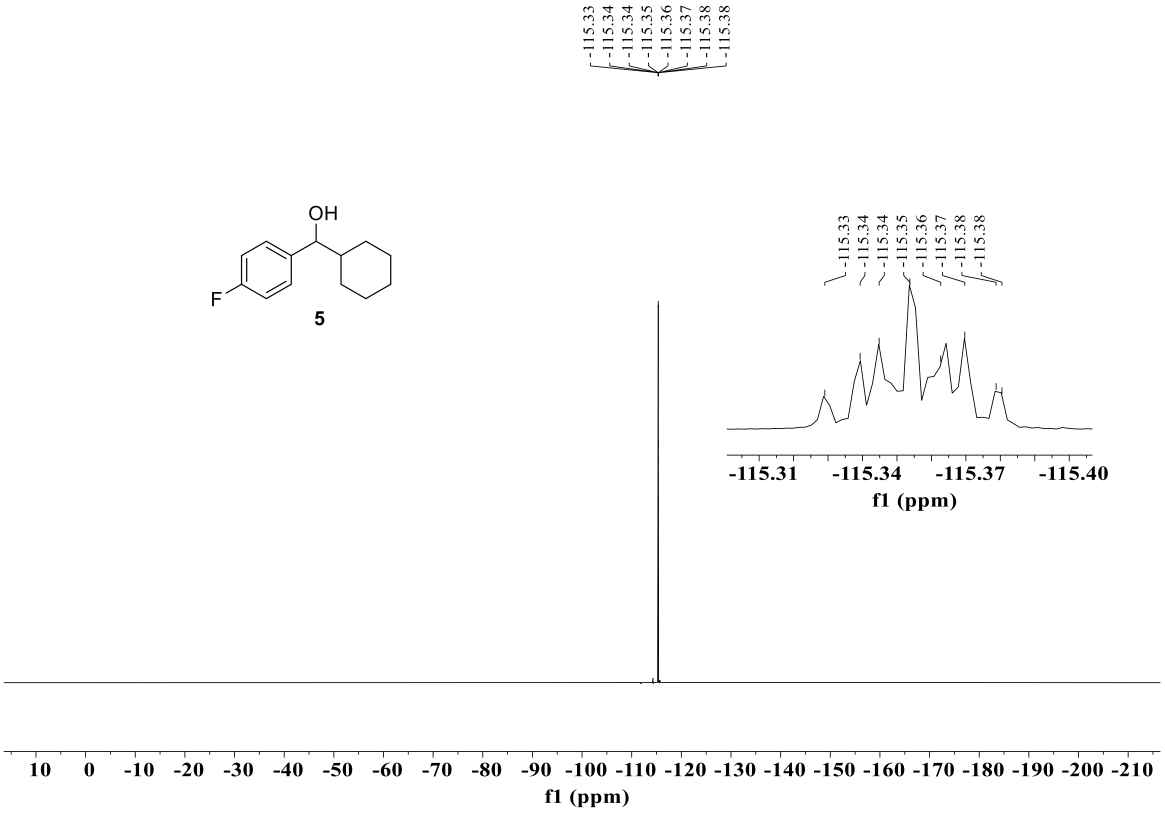
-115.33
-115.34
-115.34
-115.35
-115.36
-115.37
-115.38
-115.38
OH
F
5
-115.31
-115.34
-115.37
-115.40
f1 (ppm)
10 0 -10 -20 -30 -40 -50 -60 -70 -80 -90 -100 -110 -120 -130 -140 -150 -160 -170 -180 -190 -200 -210
f1 (ppm)

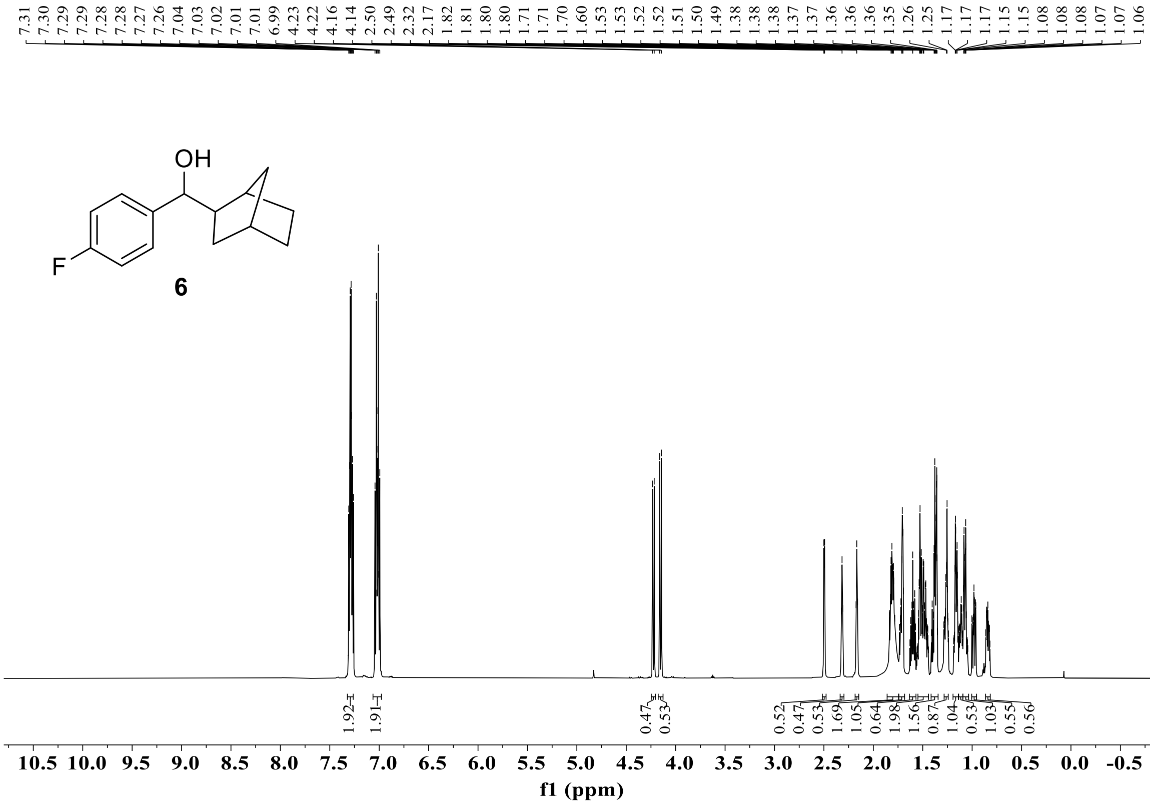
7.31 7.30 7.29 7.29 7.28 7.28 7.27 7.26 7.04 7.03 7.02 7.01 7.01 6.99 4.23 4.22 4.16 4.14 2.50 2.49 2.32 2.17 1.82 1.81 1.80 1.80 1.71 1.71 1.70 1.60 1.53 1.53 1.52 1.52 1.51 1.50 1.49 1.38 1.38 1.38 1.37 1.37 1.36 1.36 1.36 1.35 1.26 1.25 1.17 1.17 1.17 1.15 1.15 1.08 1.08 1.08 1.07 1.07 1.06
OH
F
6
1.92 1.91 0.47 0.53 0.52 0.47 0.53 1.69 1.05 0.64 1.98 1.56 0.87 1.04 0.53 1.03 0.55 0.56
10.5 10.0 9.5 9.0 8.5 8.0 7.5 7.0 6.5 6.0 5.5 5.0 4.5 4.0 3.5 3.0 2.5 2.0 1.5 1.0 0.5 0.0 -0.5
f1 (ppm)

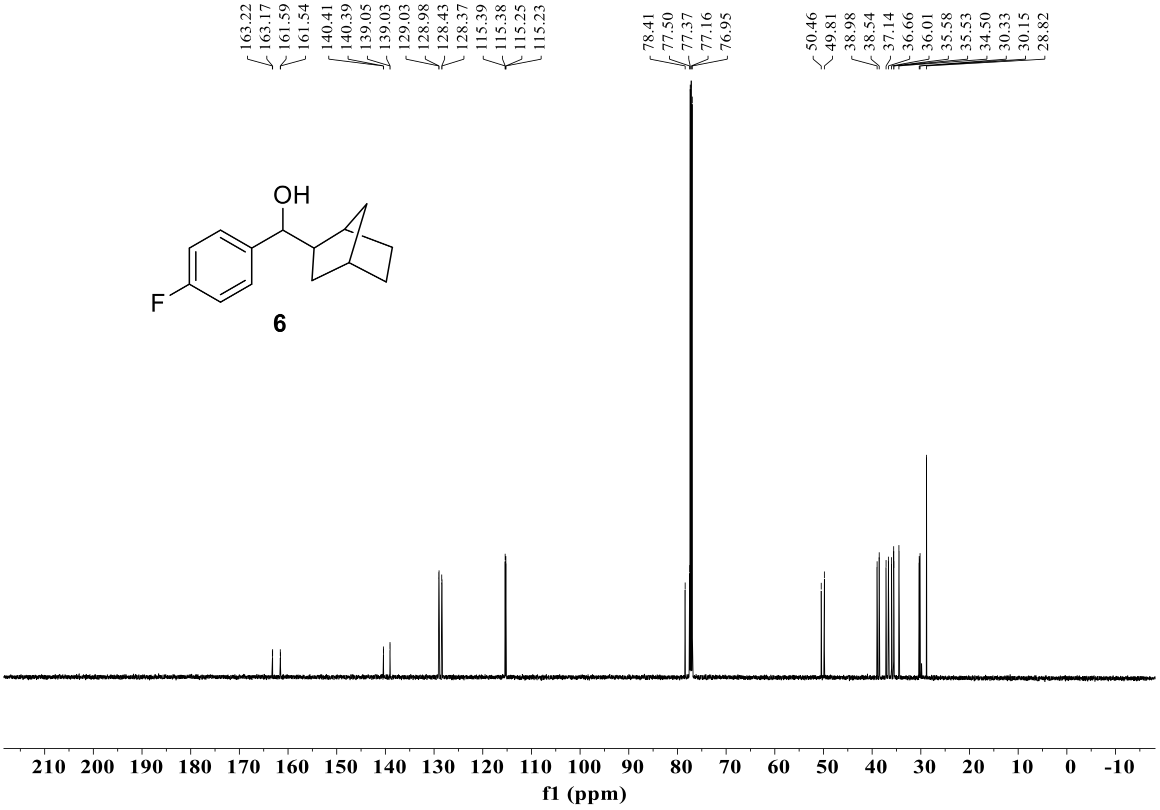
163.22 163.17 161.59 161.54 140.41 140.39 139.05 139.03 129.03 128.98 128.43 128.37 115.39 115.38 115.25 115.23
78.41 77.50 77.37 77.16 76.95
50.46 49.81 38.98 38.54 37.14 36.66 36.01 35.58 35.53 34.50 30.33 30.15 28.82
OH
F
6
210 200 190 180 170 160 150 140 130 120 110 100 90 80 70 60 50 40 30 20 10 0 -10
f1 (ppm)

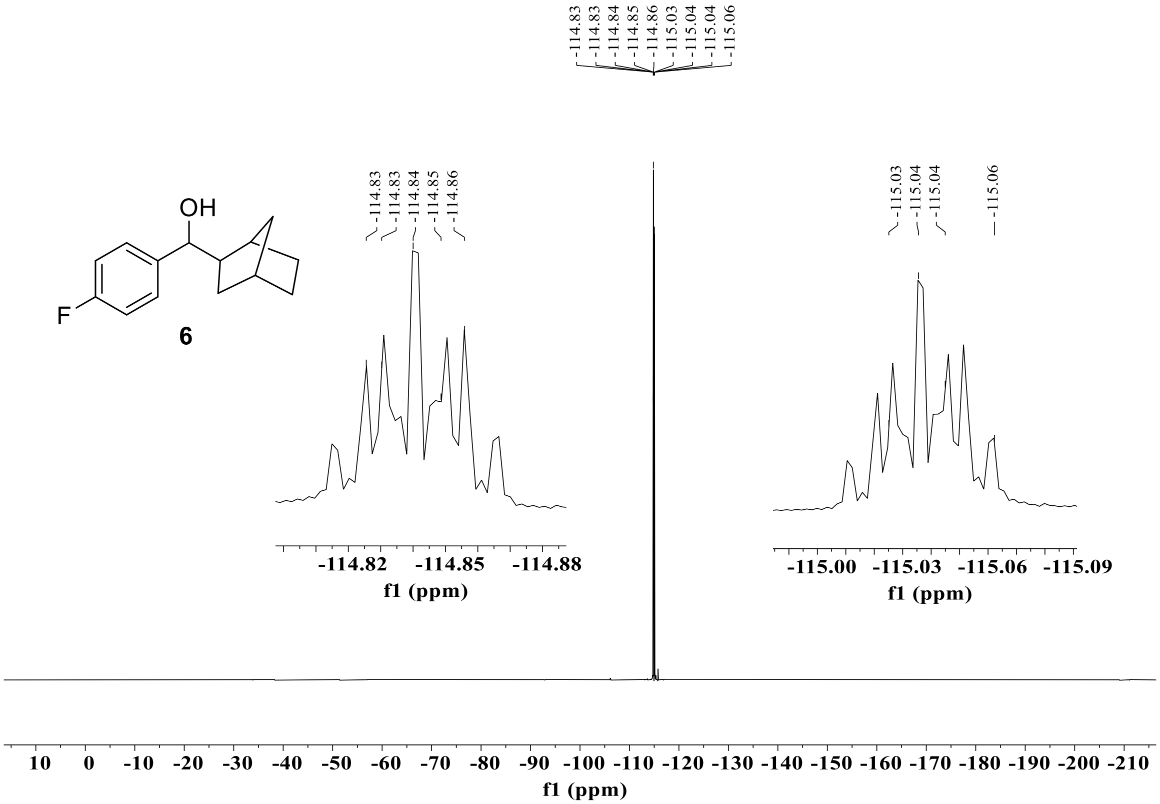
-114.83
-114.83
-114.84
-114.85
-114.86
-115.03
-115.04
-115.04
-115.06
OH
F
6
-114.82 -114.85 -114.88
f1 (ppm)
-115.00 -115.03 -115.06 -115.09
f1 (ppm)
10 0 -10 -20 -30 -40 -50 -60 -70 -80 -90 -100 -110 -120 -130 -140 -150 -160 -170 -180 -190 -200 -210
f1 (ppm)

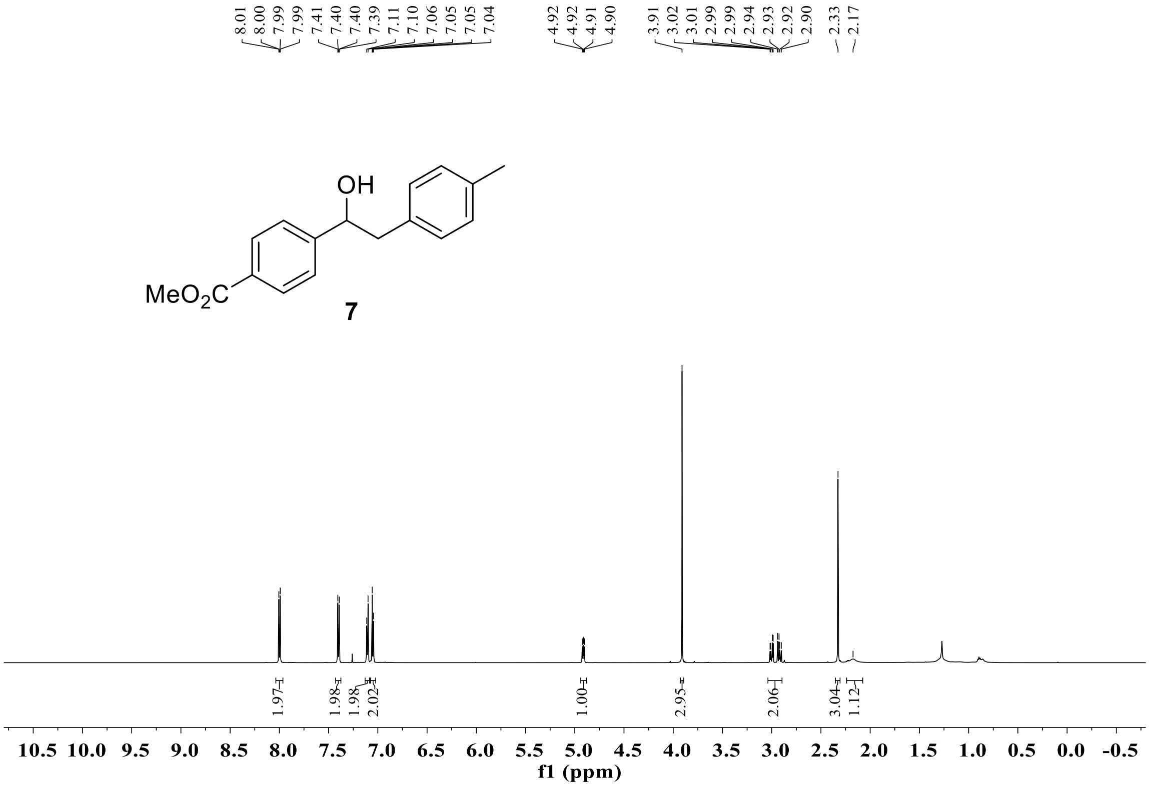
8.01
8.00
7.99
7.99
7.41
7.40
7.40
7.39
7.11
7.10
7.06
7.05
7.05
7.04
4.92
4.92
4.91
4.90
3.91
3.02
3.01
2.99
2.99
2.94
2.93
2.92
2.90
2.33
2.17
OH
MeO2C
1.97
1.98
1.98
2.02
1.00
2.95
2.06
3.04
1.12
10.5 10.0 9.5 9.0 8.5 8.0 7.5 7.0 6.5 6.0 5.5 5.0 4.5 4.0 3.5 3.0 2.5 2.0 1.5 1.0 0.5 0.0 -0.5
f1 (ppm)

7

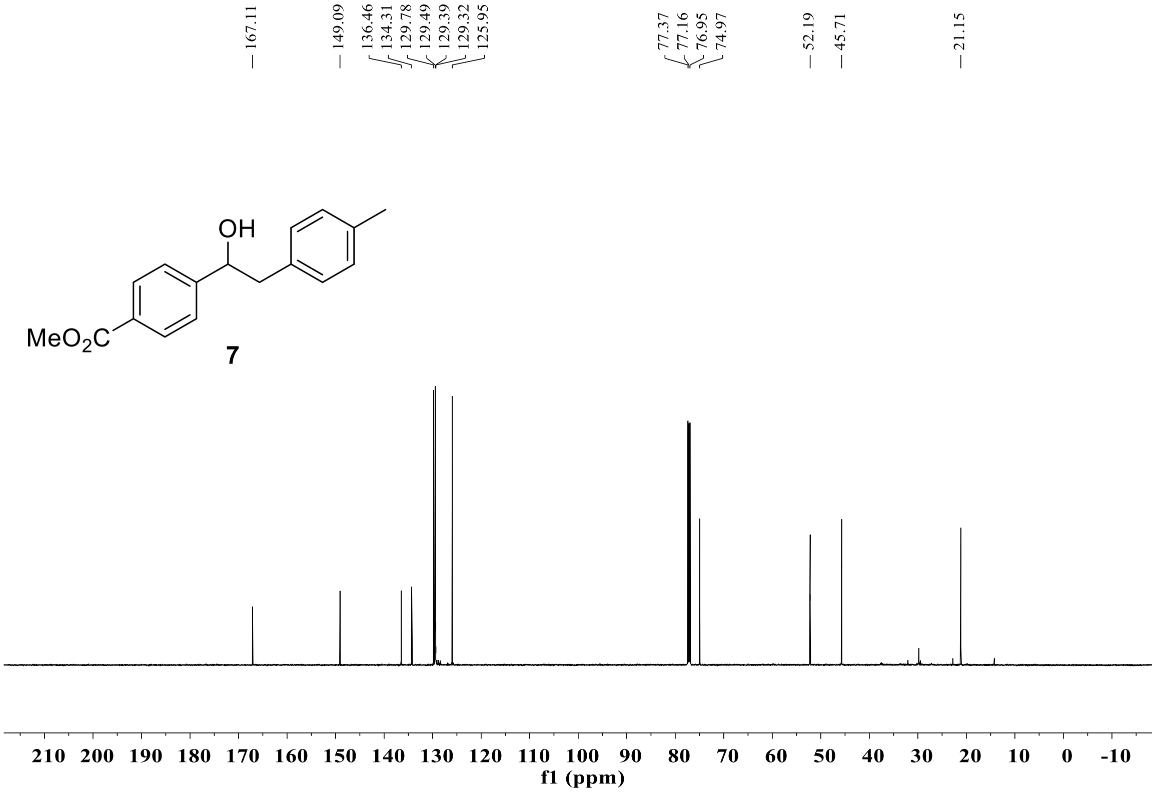
167.11
149.09
136.46
134.31
129.78
129.49
129.39
129.32
125.95
77.37
77.16
76.95
74.97
52.19
45.71
21.15
OH
MeO2C
210 200 190 180 170 160 150 140 130 120 110 100 90 80 70 60 50 40 30 20 10 0 -10
f1 (ppm)

7

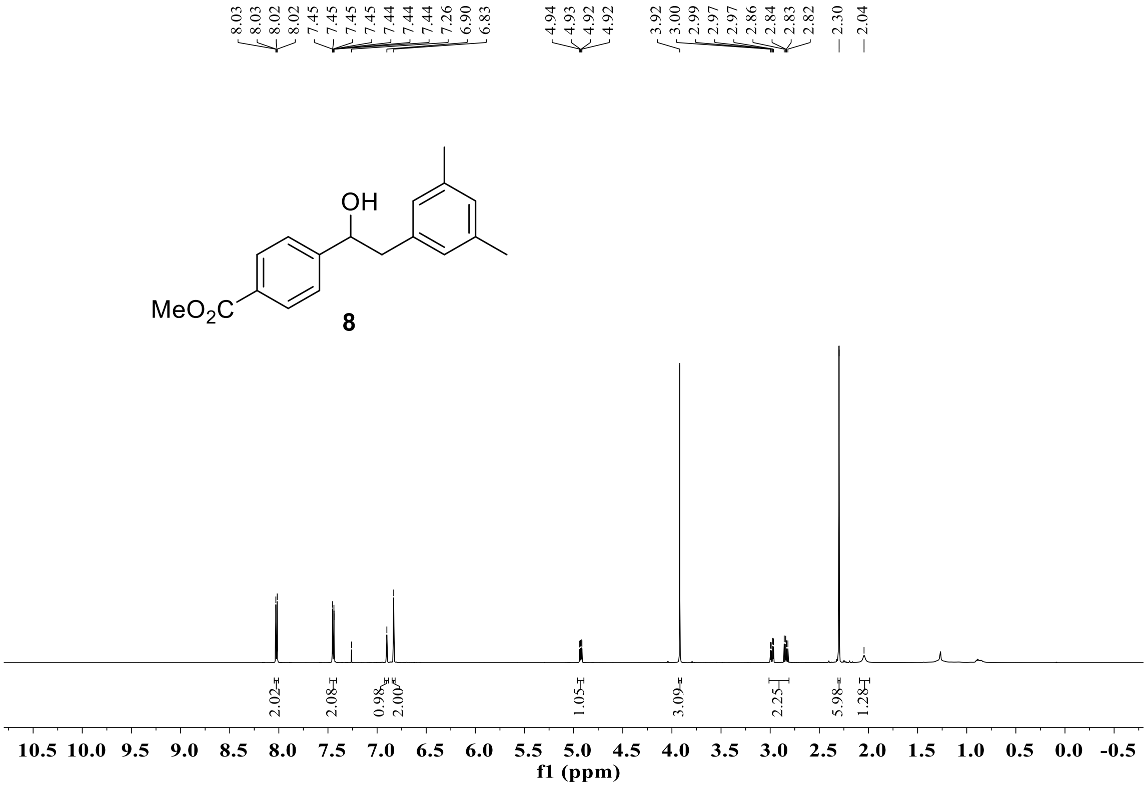

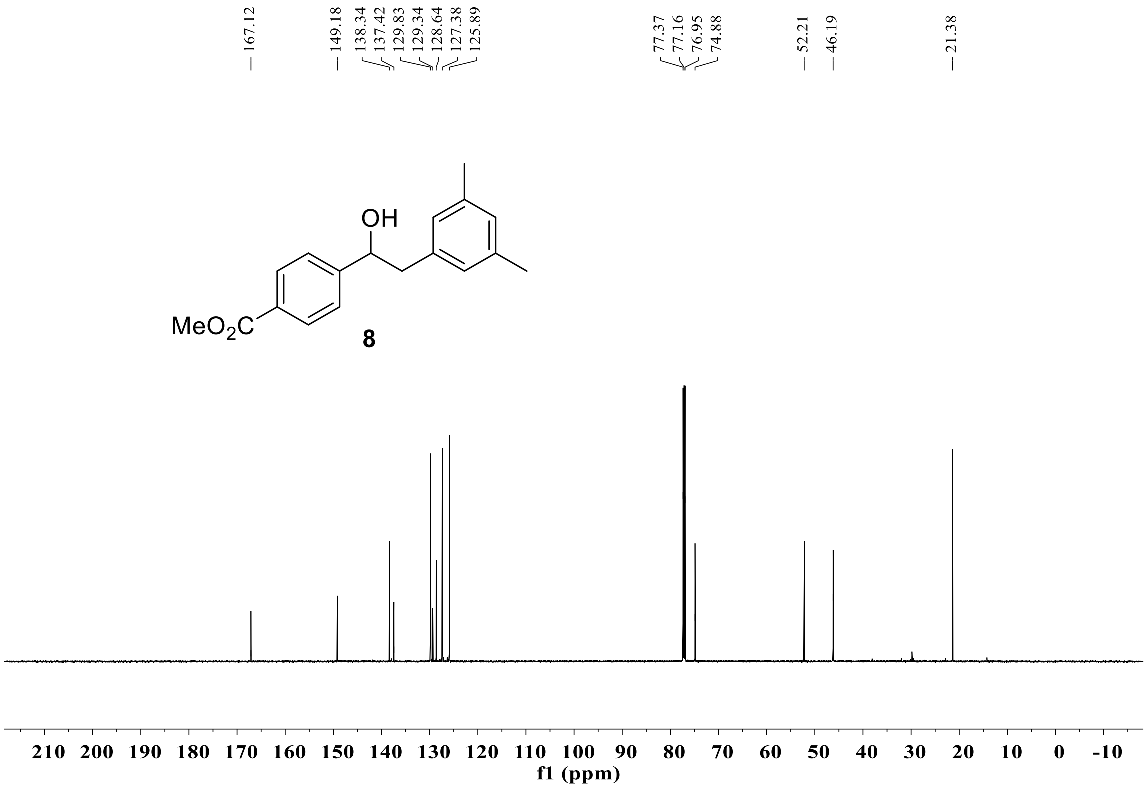

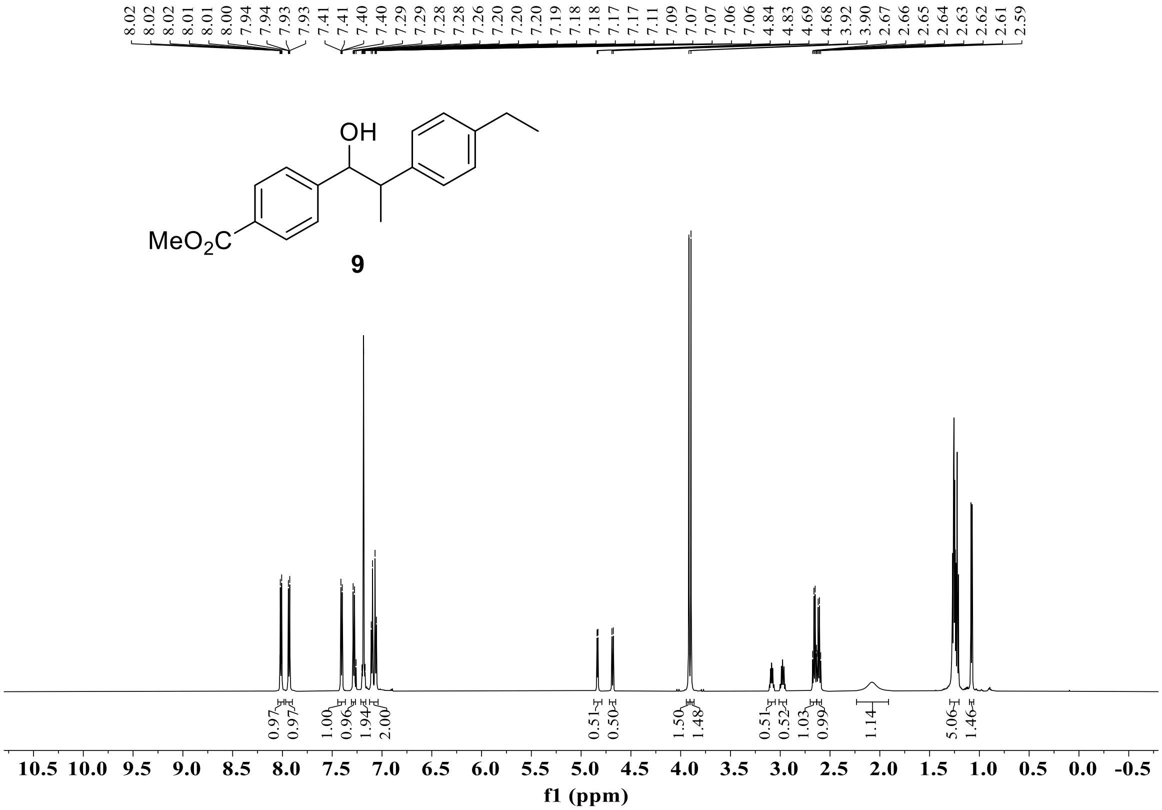
8.02
8.02
8.02
8.01
8.00
7.94
7.94
7.93
7.93
7.41
7.41
7.40
7.40
7.29
7.29
7.28
7.26
7.20
7.20
7.20
7.19
7.18
7.18
7.17
7.17
7.11
7.09
7.07
7.07
7.06
4.84
4.83
4.69
4.68
3.92
3.90
2.67
2.66
2.65
2.64
2.63
2.62
2.61
2.59
OH
MeO2C
9
0.97
0.97
1.00
0.96
1.94
2.00
0.51
0.50
1.50
1.48
0.51
0.52
1.03
0.99
1.14
5.06
1.46
10.5 10.0 9.5 9.0 8.5 8.0 7.5 7.0 6.5 6.0 5.5 5.0 4.5 4.0 3.5 3.0 2.5 2.0 1.5 1.0 0.5 0.0 -0.5
f1 (ppm)

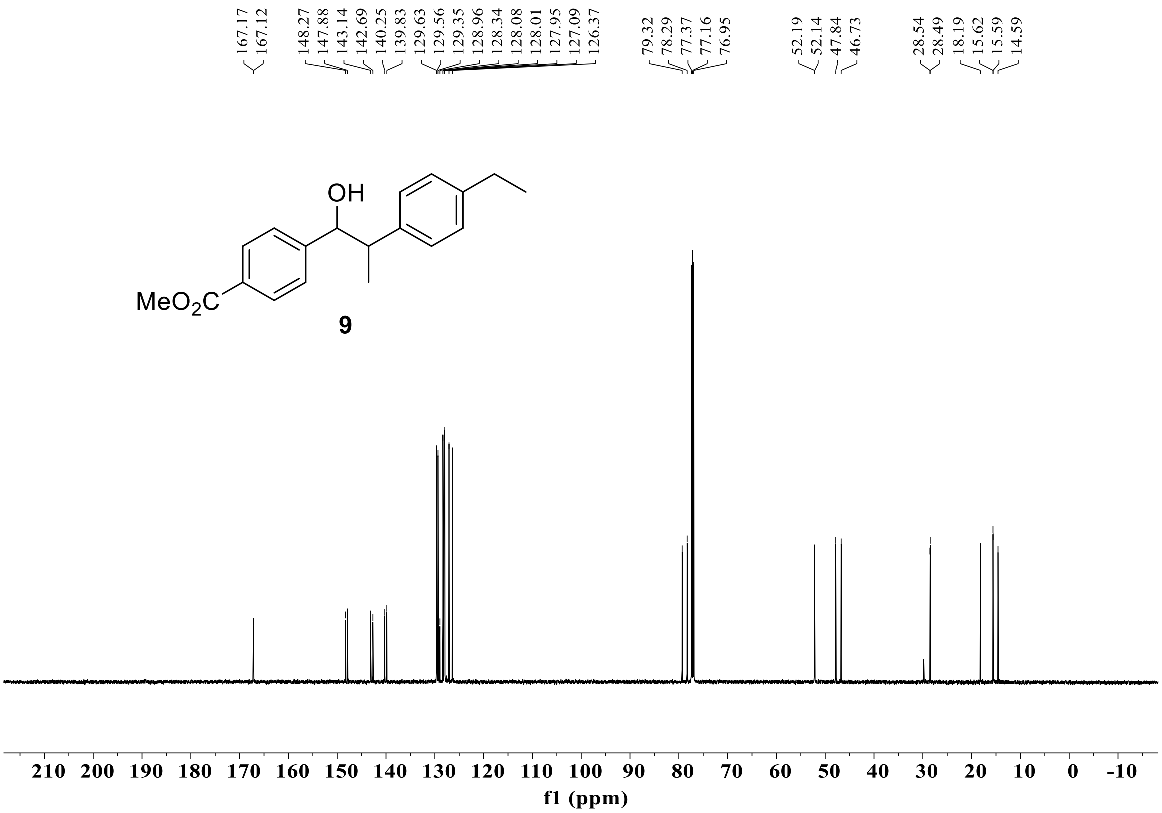
167.17
167.12
148.27
147.88
143.14
142.69
140.25
139.83
129.63
129.56
129.35
128.96
128.34
128.08
128.01
127.95
127.09
126.37
79.32
78.29
77.37
77.16
76.95
52.19
52.14
47.84
46.73
28.54
28.49
18.19
15.62
15.59
14.59
OH
MeO2C
9
210 200 190 180 170 160 150 140 130 120 110 100 90 80 70 60 50 40 30 20 10 0 -10
f1 (ppm)

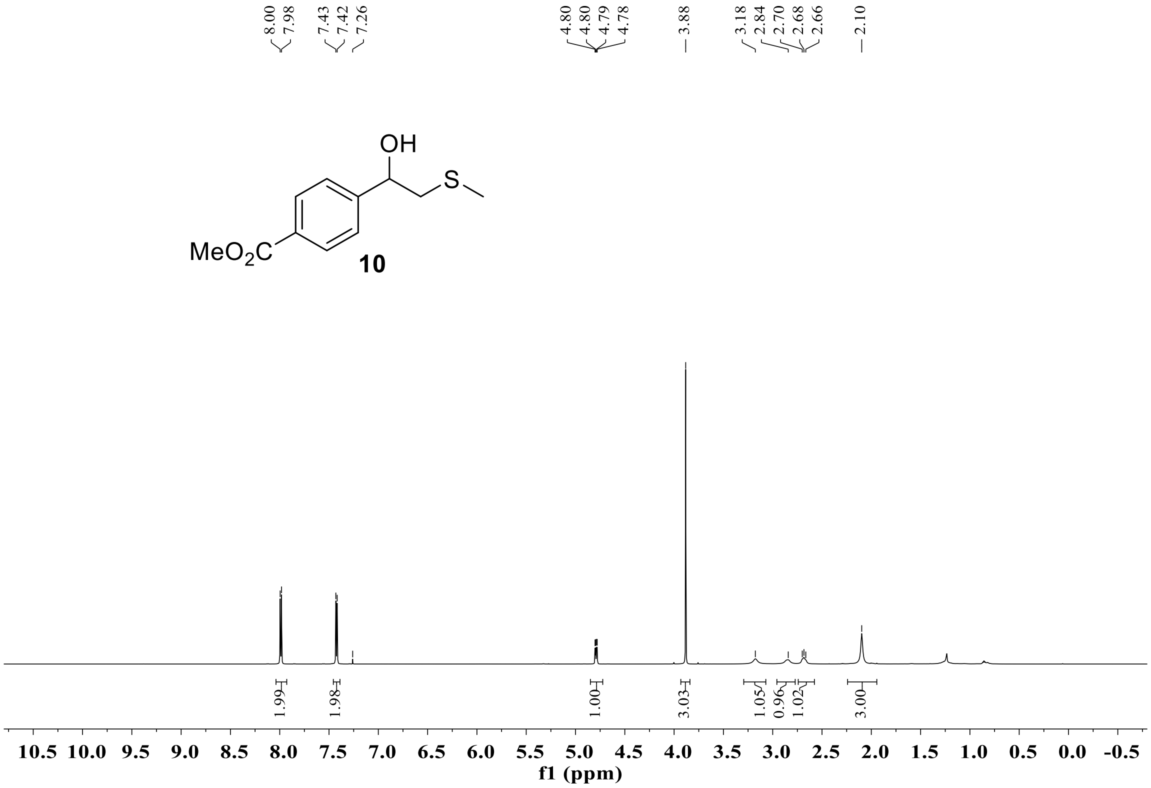
8.00
7.98
7.43
7.42
7.26
4.80
4.80
4.79
4.78
3.88
3.18
2.84
2.70
2.68
2.66
2.10
OH
S
MeO2C
10
1.99
1.98
1.00
3.03
1.05
0.96
1.02
3.00
10.5 10.0 9.5 9.0 8.5 8.0 7.5 7.0 6.5 6.0 5.5 5.0 4.5 4.0 3.5 3.0 2.5 2.0 1.5 1.0 0.5 0.0 -0.5
f1 (ppm)

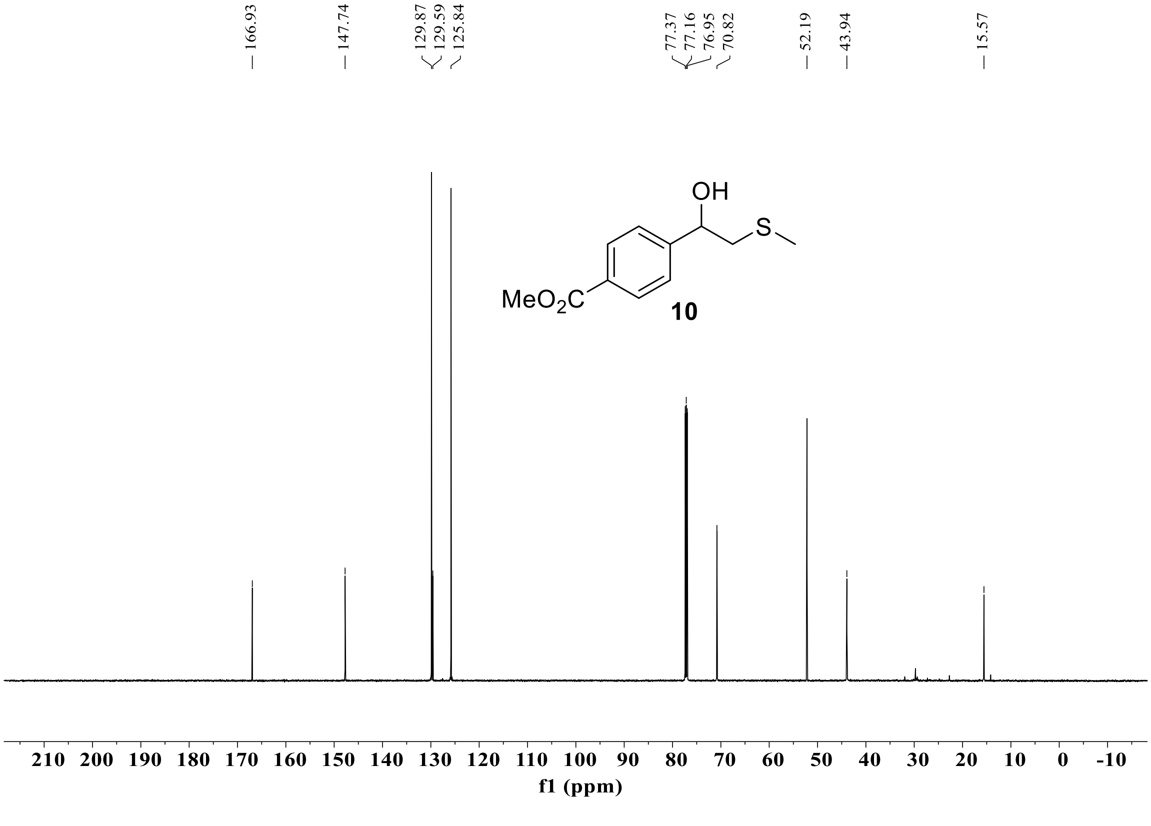
166.93
147.74
129.87
129.59
125.84
77.37
77.16
76.95
70.82
52.19
43.94
15.57
OH
S
MeO2C
10
210 200 190 180 170 160 150 140 130 120 110 100 90 80 70 60 50 40 30 20 10 0 -10
f1 (ppm)

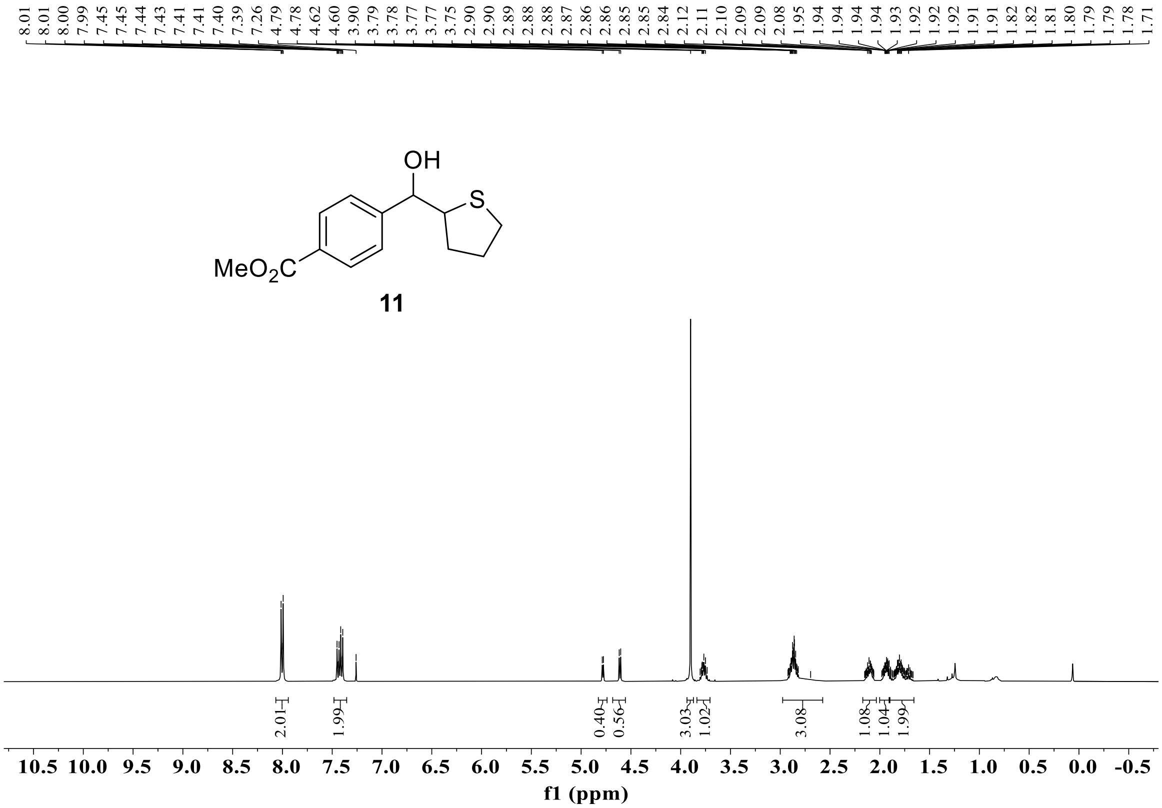
8.01 8.01 8.00 7.99 7.45 7.45 7.44 7.43 7.41 7.41 7.40 7.39 7.26 4.79 4.78 4.62 4.60 3.90 3.79 3.78 3.77 3.77 3.75 2.90 2.90 2.89 2.88 2.88 2.87 2.86 2.86 2.85 2.85 2.84 2.12 2.11 2.10 2.09 2.09 2.08 1.95 1.94 1.94 1.94 1.94 1.93 1.92 1.92 1.92 1.91 1.91 1.82 1.82 1.81 1.80 1.79 1.78 1.71
OH
S
MeO2C
11
2.01
1.99
0.40
0.56
3.03
1.02
3.08
1.08
1.04
1.99
10.5 10.0 9.5 9.0 8.5 8.0 7.5 7.0 6.5 6.0 5.5 5.0 4.5 4.0 3.5 3.0 2.5 2.0 1.5 1.0 0.5 0.0 -0.5
f1 (ppm)

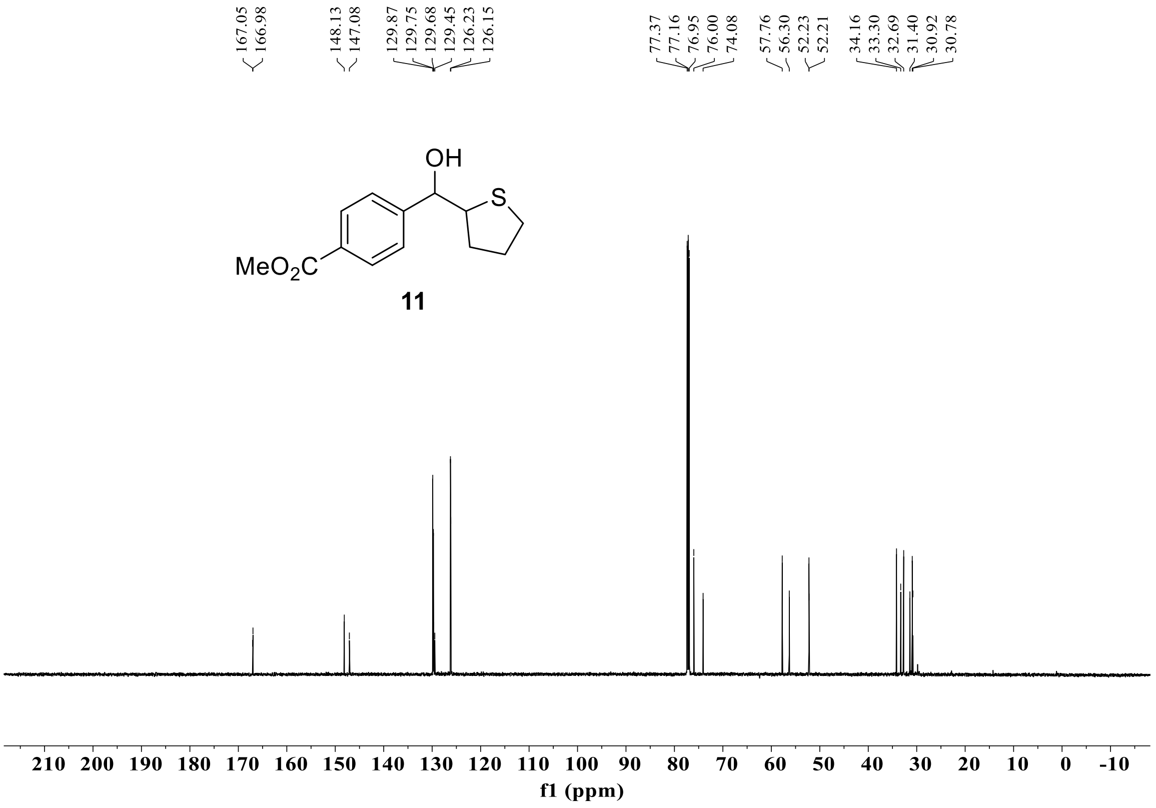
167.05 166.98
148.13 147.08
129.87 129.75 129.68 129.45 126.23 126.15
77.37 77.16 76.95 76.00 74.08
57.76 56.30 52.23 52.21
34.16 33.30 32.69 31.40 30.92 30.78
OH
S
MeO2C
11
210 200 190 180 170 160 150 140 130 120 110 100 90 80 70 60 50 40 30 20 10 0 -10
f1 (ppm)

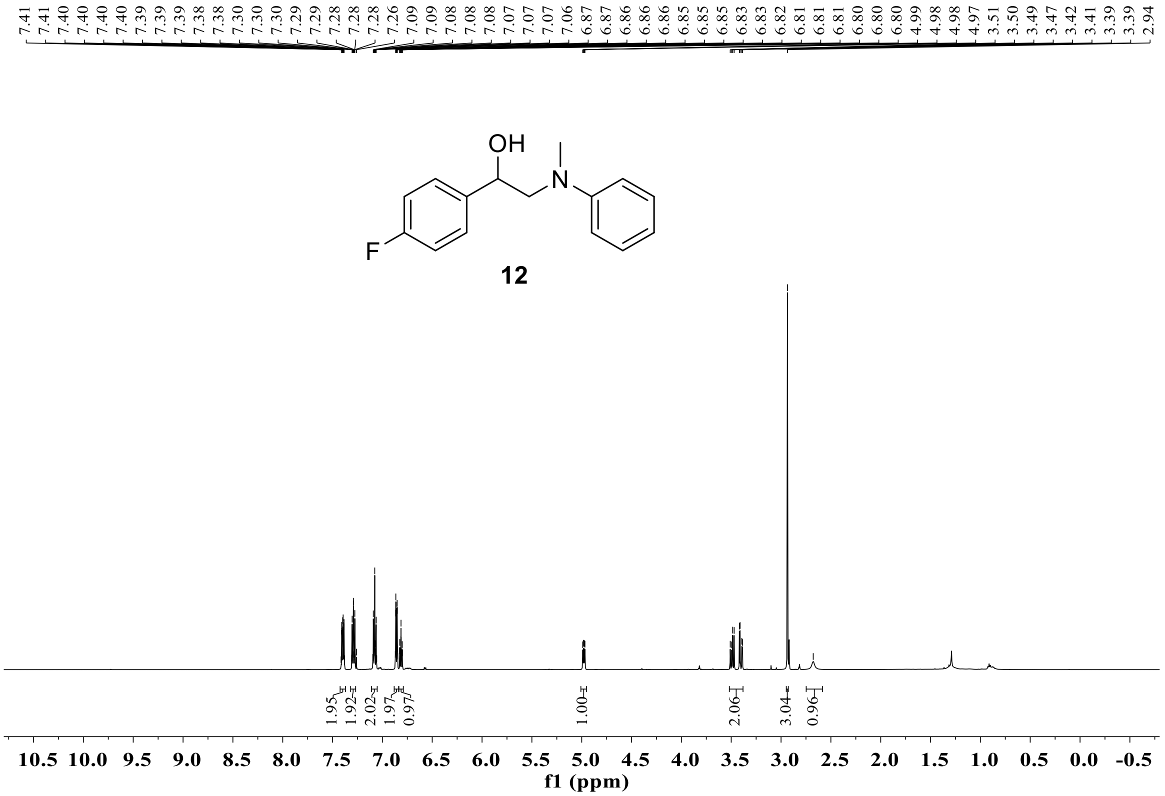
7.41 7.41 7.40 7.40 7.40 7.39 7.39 7.39 7.38 7.38 7.30 7.30 7.29 7.29 7.28 7.28 7.28 7.26 7.09 7.09 7.08 7.08 7.08 7.07 7.07 7.07 7.06 6.87 6.87 6.86 6.86 6.86 6.85 6.85 6.85 6.83 6.83 6.82 6.81 6.81 6.81 6.80 6.80 6.80 4.99 4.98 4.98 4.97 3.51 3.50 3.49 3.47 3.42 3.41 3.39 3.39 2.94
OH
N
F
12
1.95
1.92
2.02
1.97
0.97
1.00
2.06
3.04
0.96
10.5 10.0 9.5 9.0 8.5 8.0 7.5 7.0 6.5 6.0 5.5 5.0 4.5 4.0 3.5 3.0 2.5 2.0 1.5 1.0 0.5 0.0 -0.5
f1 (ppm)

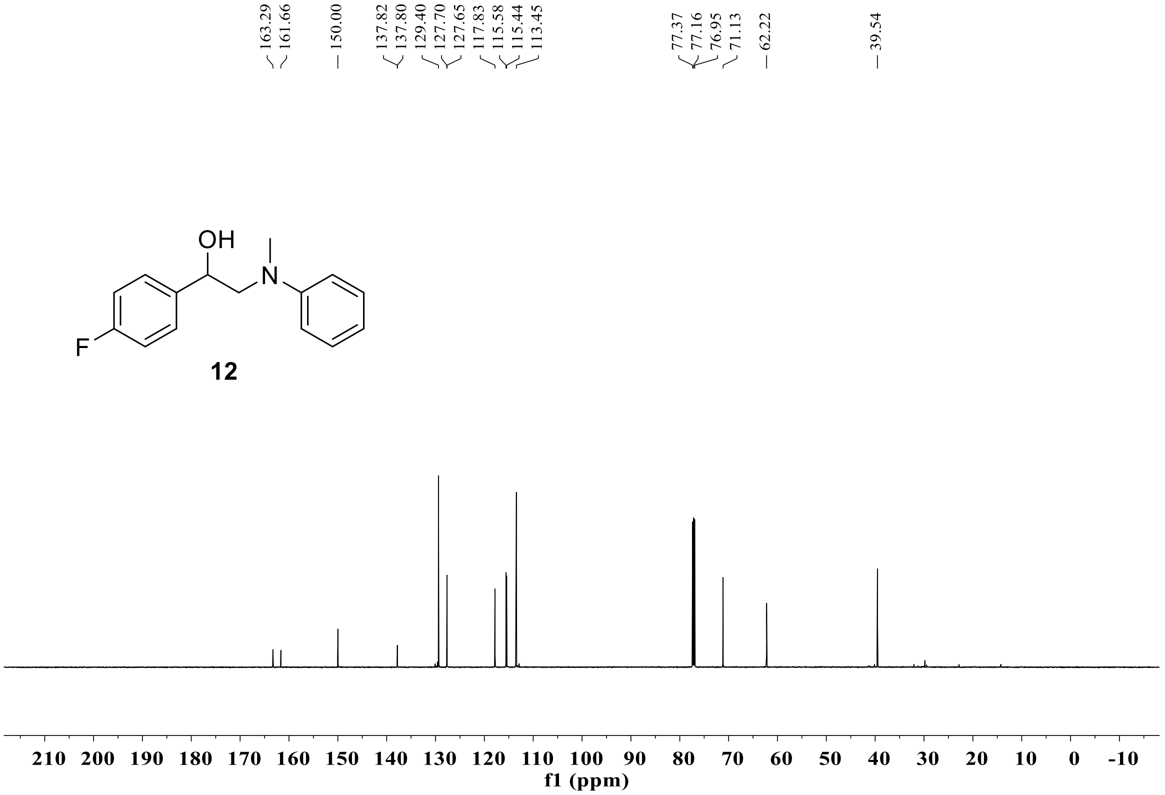
163.29 161.66 150.00 137.82 137.80 129.40 127.70 127.65 117.83 115.58 115.44 113.45 77.37 77.16 76.95 71.13 62.22 39.54
OH
N
F
12
210 200 190 180 170 160 150 140 130 120 110 100 90 80 70 60 50 40 30 20 10 0 -10
f1 (ppm)

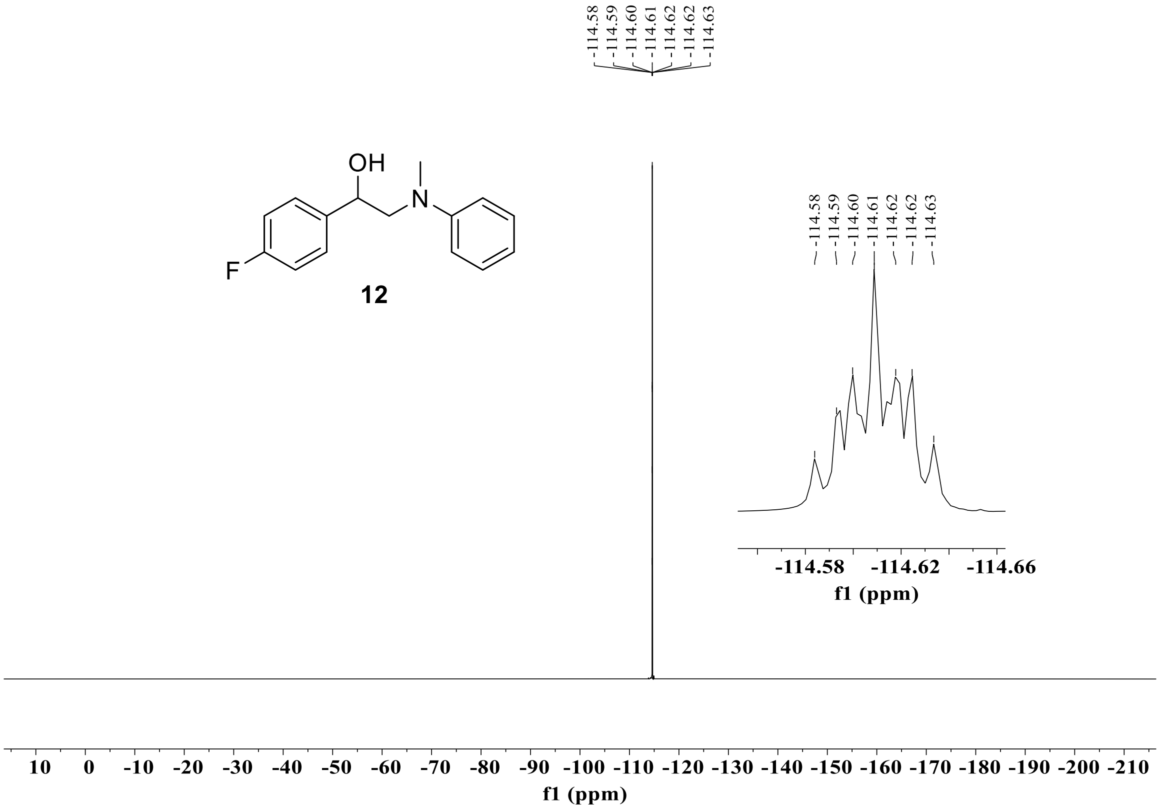
-114.58
-114.59
-114.60
-114.61
-114.62
-114.62
-114.63
OH
N
F
12
-114.58
-114.62
-114.66
f1 (ppm)
10 0 -10 -20 -30 -40 -50 -60 -70 -80 -90 -100 -110 -120 -130 -140 -150 -160 -170 -180 -190 -200 -210
f1 (ppm)

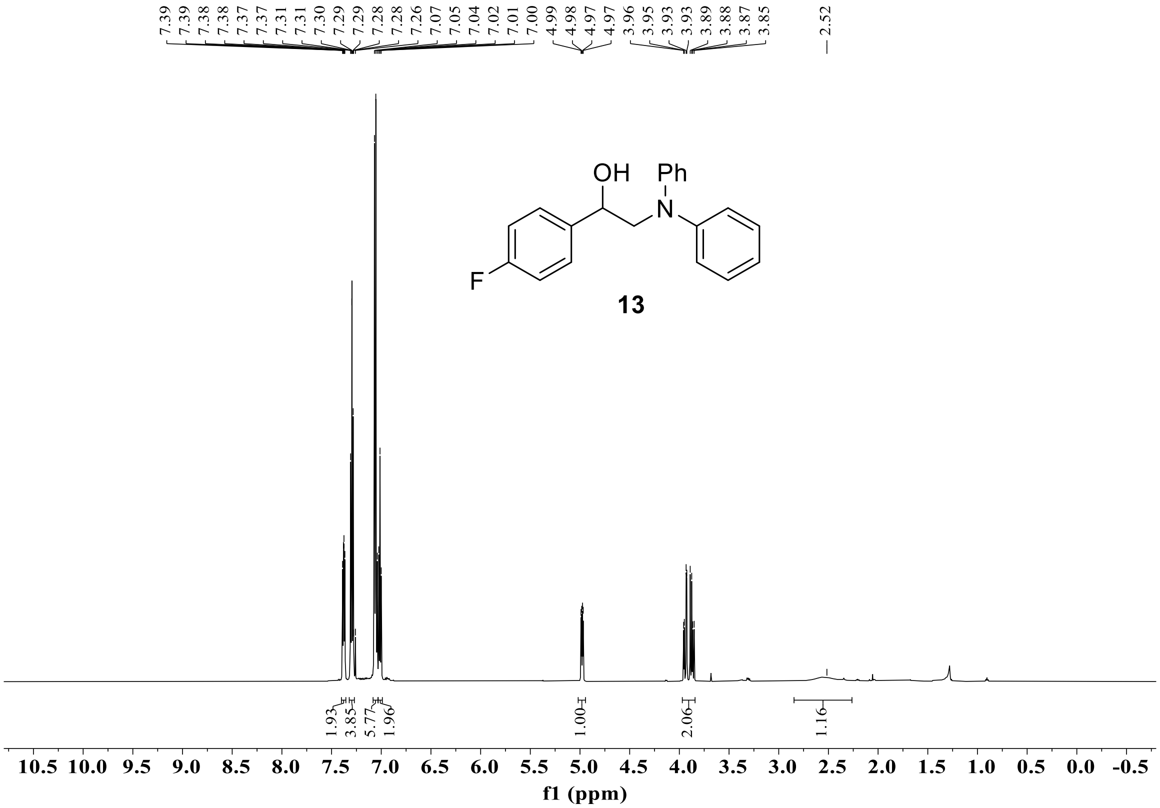
7.39 7.39 7.38 7.38 7.37 7.37 7.31 7.31 7.30 7.29 7.29 7.28 7.28 7.26 7.07 7.05 7.04 7.02 7.01 7.00 4.99 4.98 4.97 4.97 3.96 3.95 3.93 3.93 3.89 3.88 3.87 3.85 2.52
OH
Ph
N
F
13
1.93
3.85
5.77
1.96
1.00
2.06
1.16
10.5 10.0 9.5 9.0 8.5 8.0 7.5 7.0 6.5 6.0 5.5 5.0 4.5 4.0 3.5 3.0 2.5 2.0 1.5 1.0 0.5 0.0 -0.5
f1 (ppm)

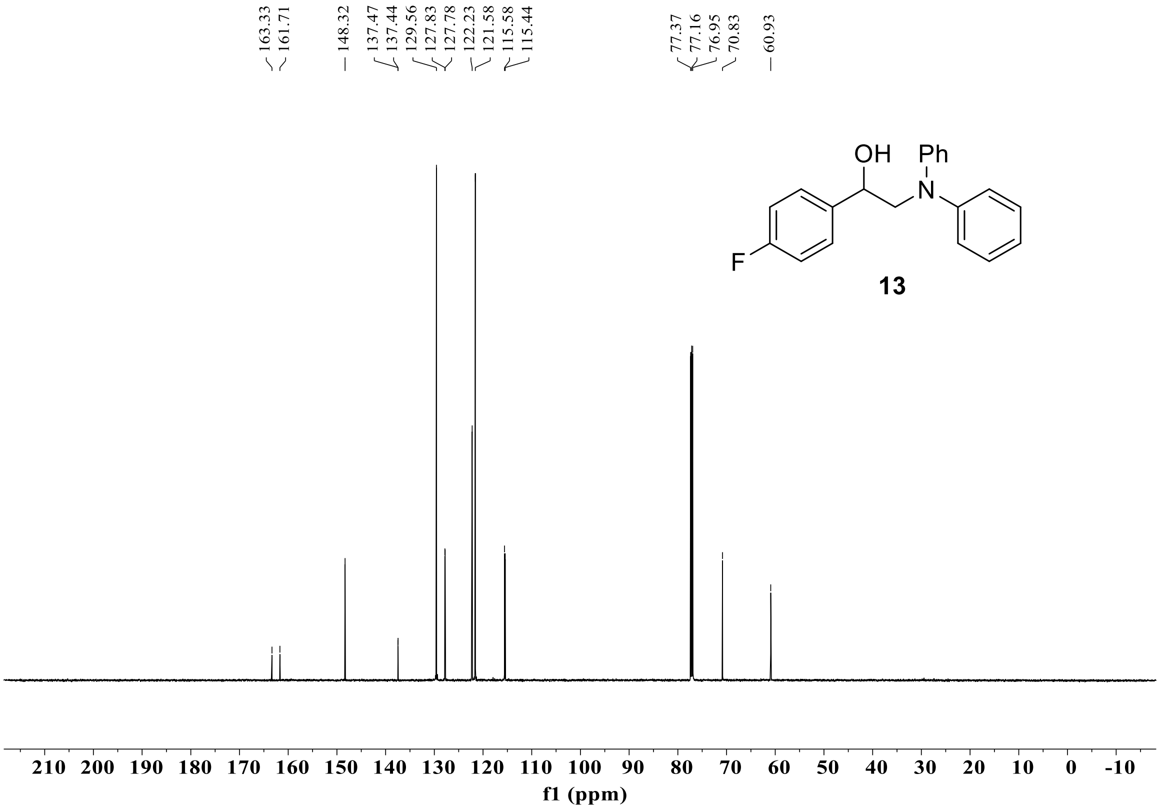
163.33 161.71 148.32 137.47 137.44 129.56 127.83 127.78 122.23 121.58 115.58 115.44 77.37 77.16 76.95 70.83 60.93
OH
Ph
N
F
13
210 200 190 180 170 160 150 140 130 120 110 100 90 80 70 60 50 40 30 20 10 0 -10
f1 (ppm)

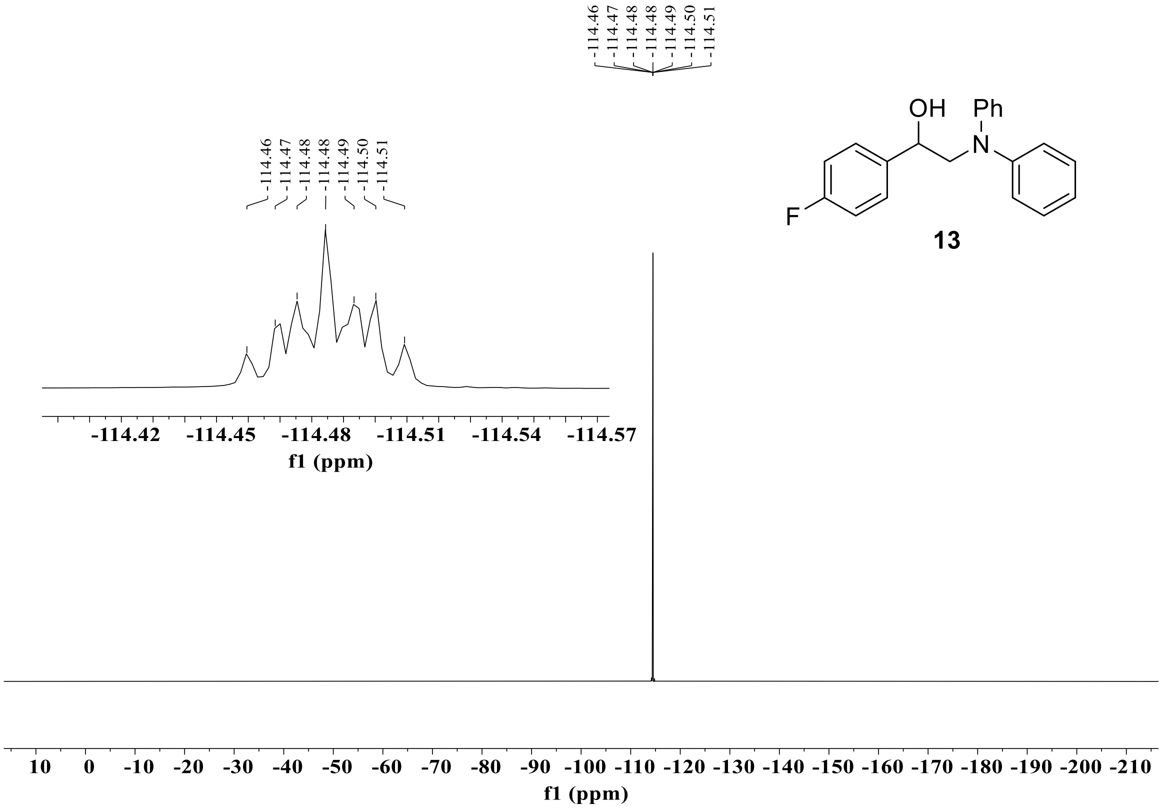
-114.46
-114.47
-114.48
-114.48
-114.49
-114.50
-114.51
OH
Ph
N
F
13
-114.42
-114.45
-114.48
-114.51
-114.54
-114.57
f1 (ppm)
10 0 -10 -20 -30 -40 -50 -60 -70 -80 -90 -100 -110 -120 -130 -140 -150 -160 -170 -180 -190 -200 -210
f1 (ppm)

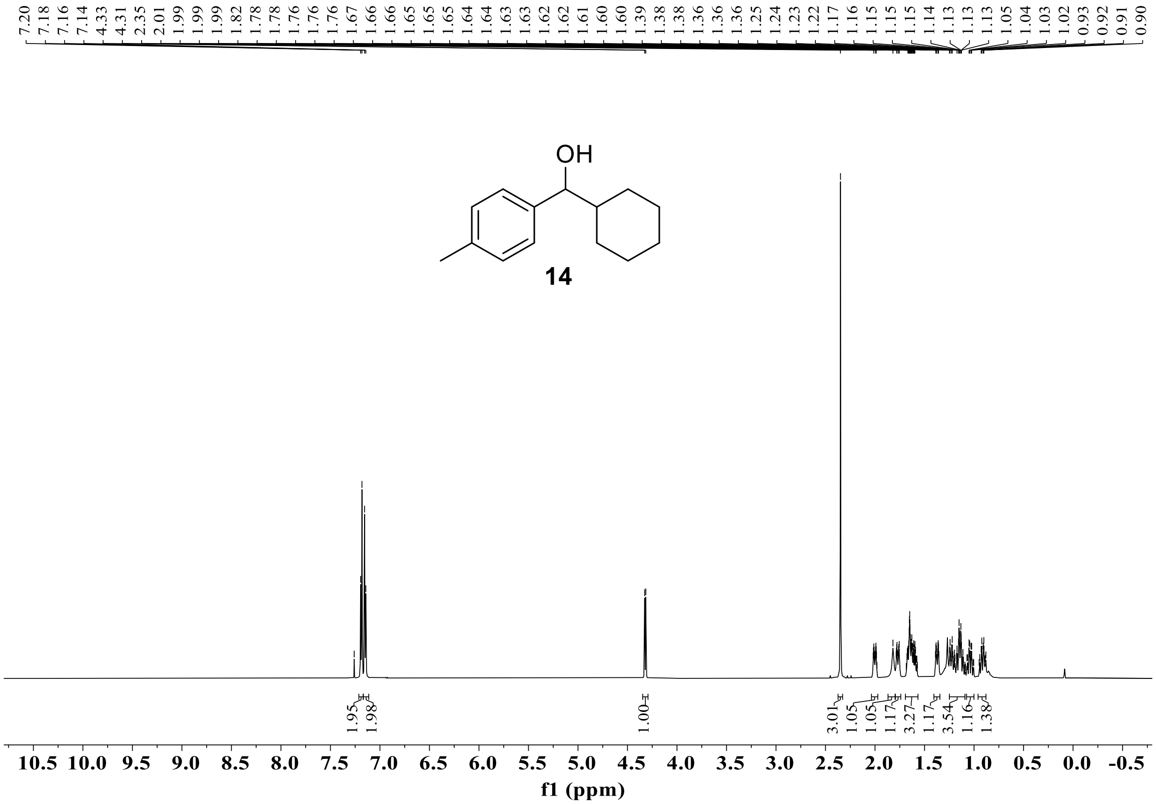
OH
14
f1 (ppm)

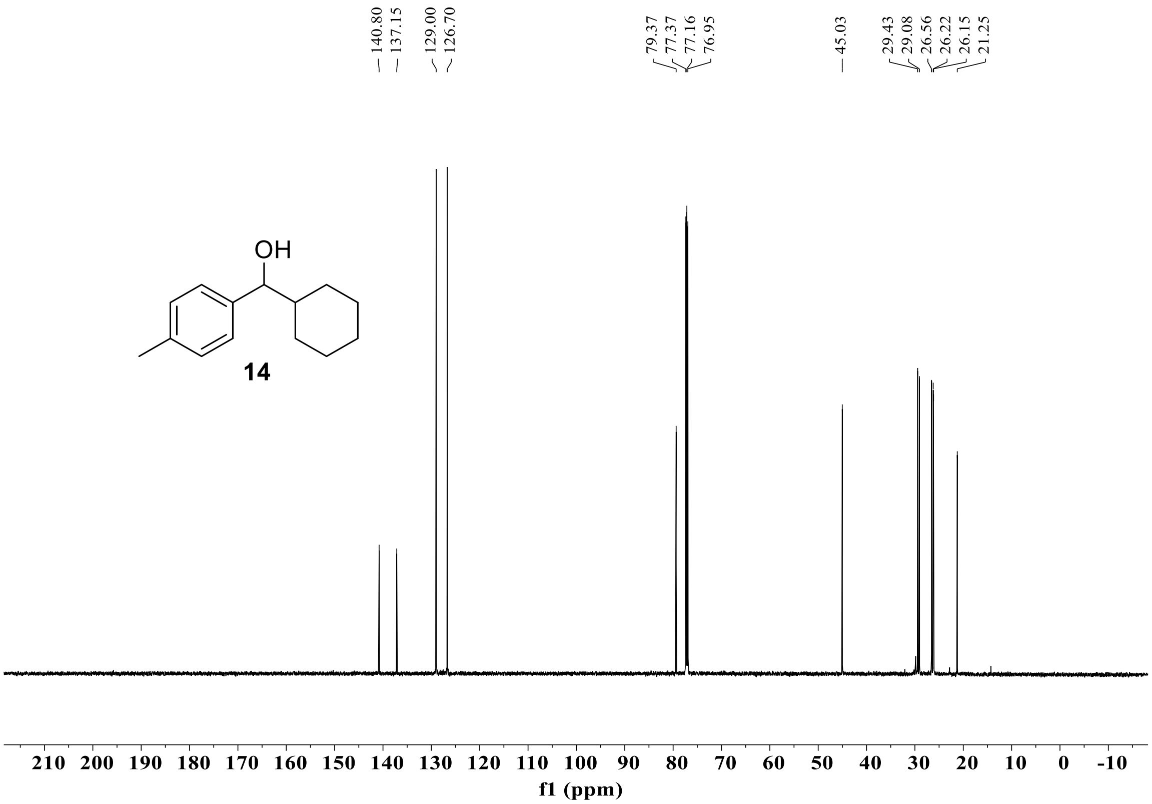
OH
14
f1 (ppm)

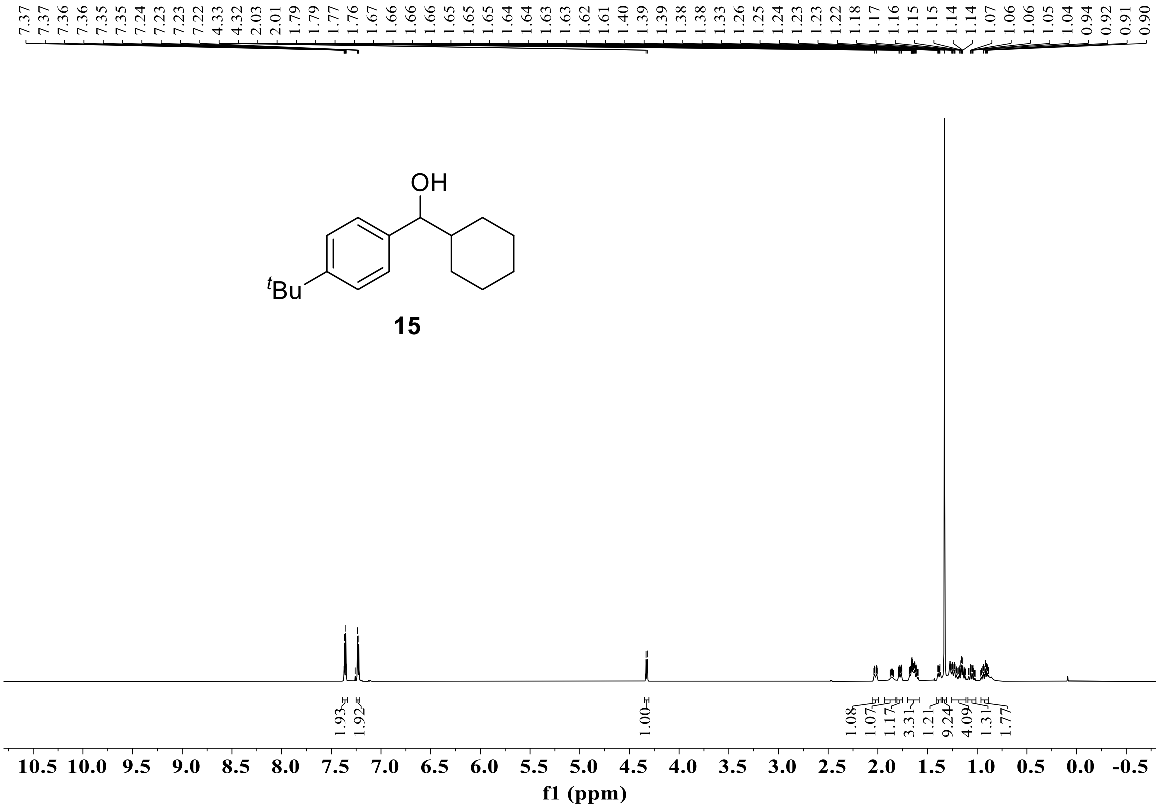
7.37 7.37 7.36 7.36 7.35 7.35 7.24 7.23 7.23 7.22 4.33 4.32 2.03 2.01 1.79 1.79 1.77 1.76 1.67 1.66 1.66 1.65 1.65 1.65 1.64 1.64 1.63 1.63 1.62 1.61 1.40 1.39 1.39 1.38 1.38 1.33 1.26 1.25 1.24 1.23 1.23 1.22 1.18 1.17 1.16 1.15 1.15 1.14 1.14 1.07 1.06 1.06 1.05 1.04 0.94 0.92 0.91 0.90
OH
tBu
15
1.93 1.92 1.00 1.08 1.07 1.17 3.31 1.21 9.24 4.09 1.31 1.77
10.5 10.0 9.5 9.0 8.5 8.0 7.5 7.0 6.5 6.0 5.5 5.0 4.5 4.0 3.5 3.0 2.5 2.0 1.5 1.0 0.5 0.0 -0.5
f1 (ppm)

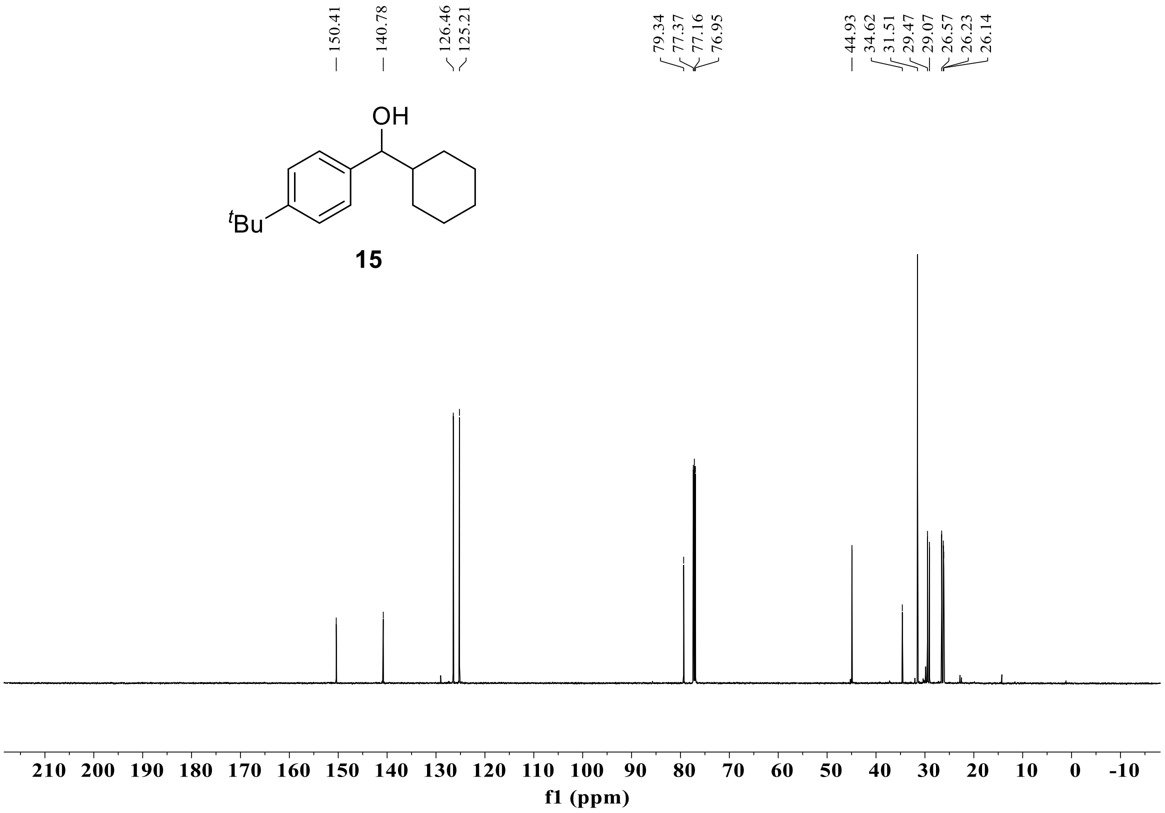
150.41 140.78 126.46 125.21 79.34 77.37 77.16 76.95 44.93 34.62 31.51 29.47 29.07 26.57 26.23 26.14
OH
tBu
15
210 200 190 180 170 160 150 140 130 120 110 100 90 80 70 60 50 40 30 20 10 0 -10
f1 (ppm)

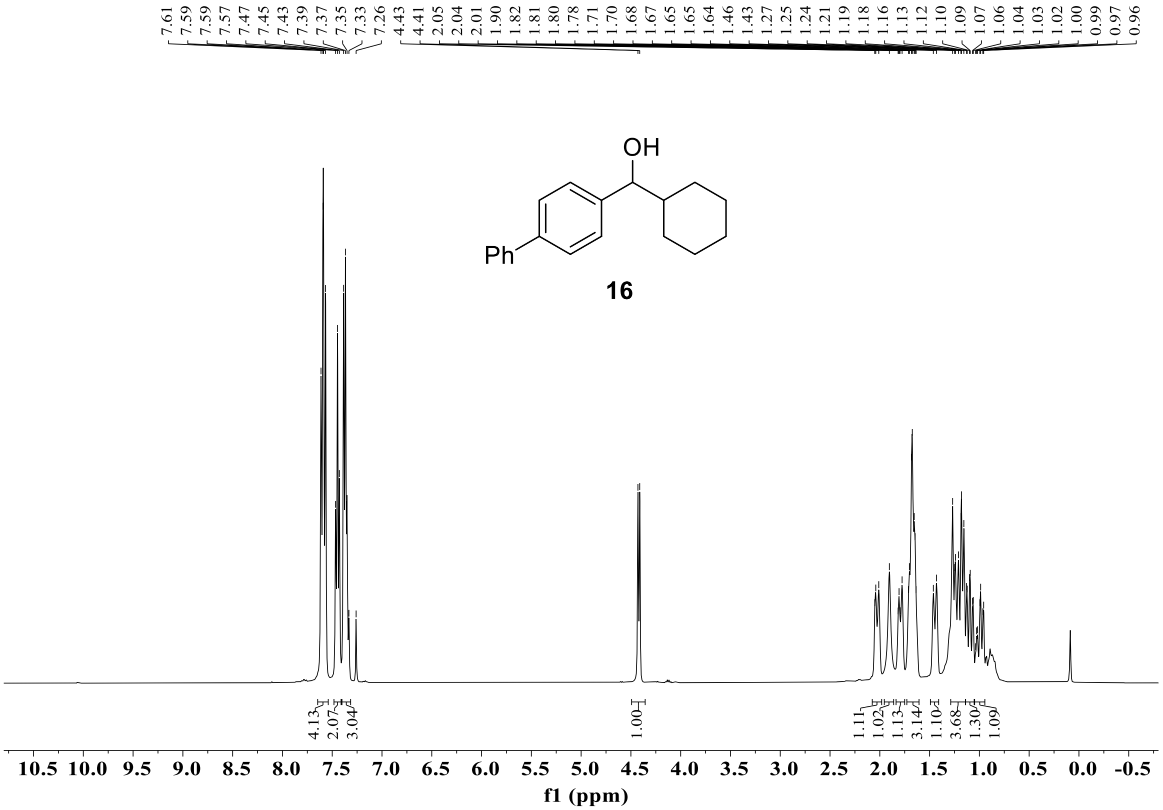
OH
Ph
16
f1 (ppm)

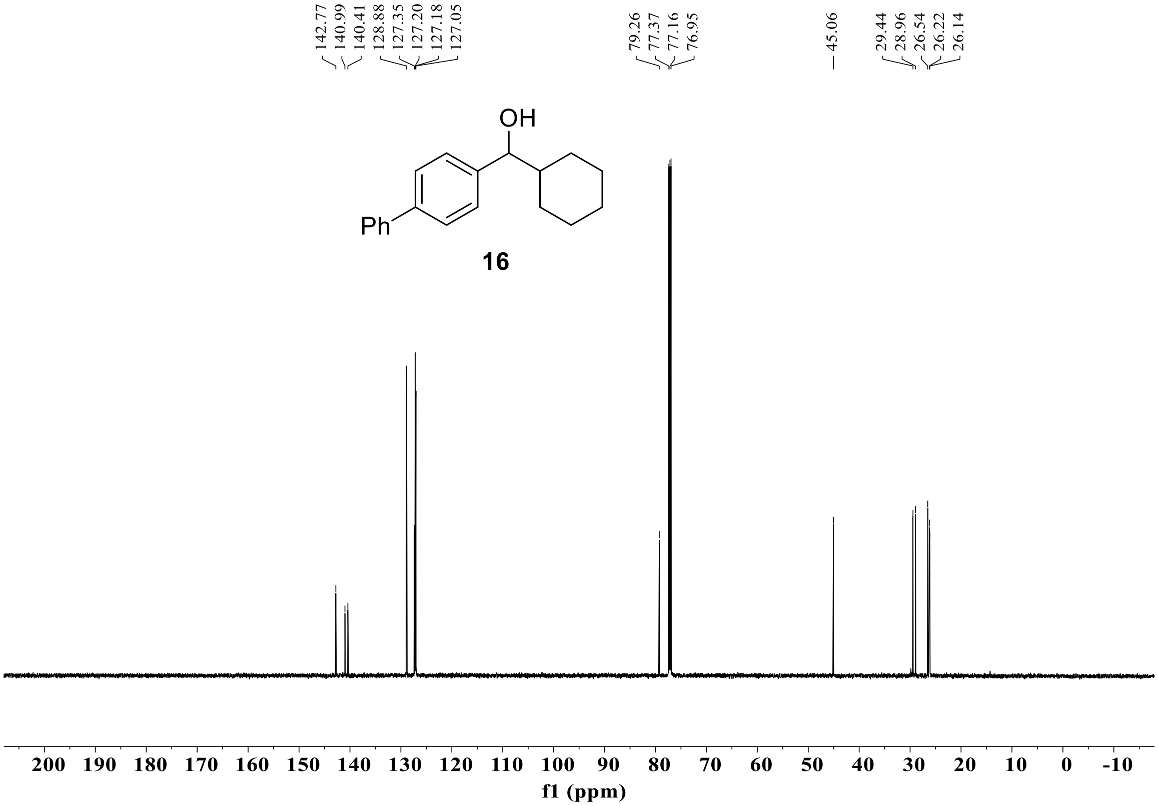
OH
Ph
16
f1 (ppm)

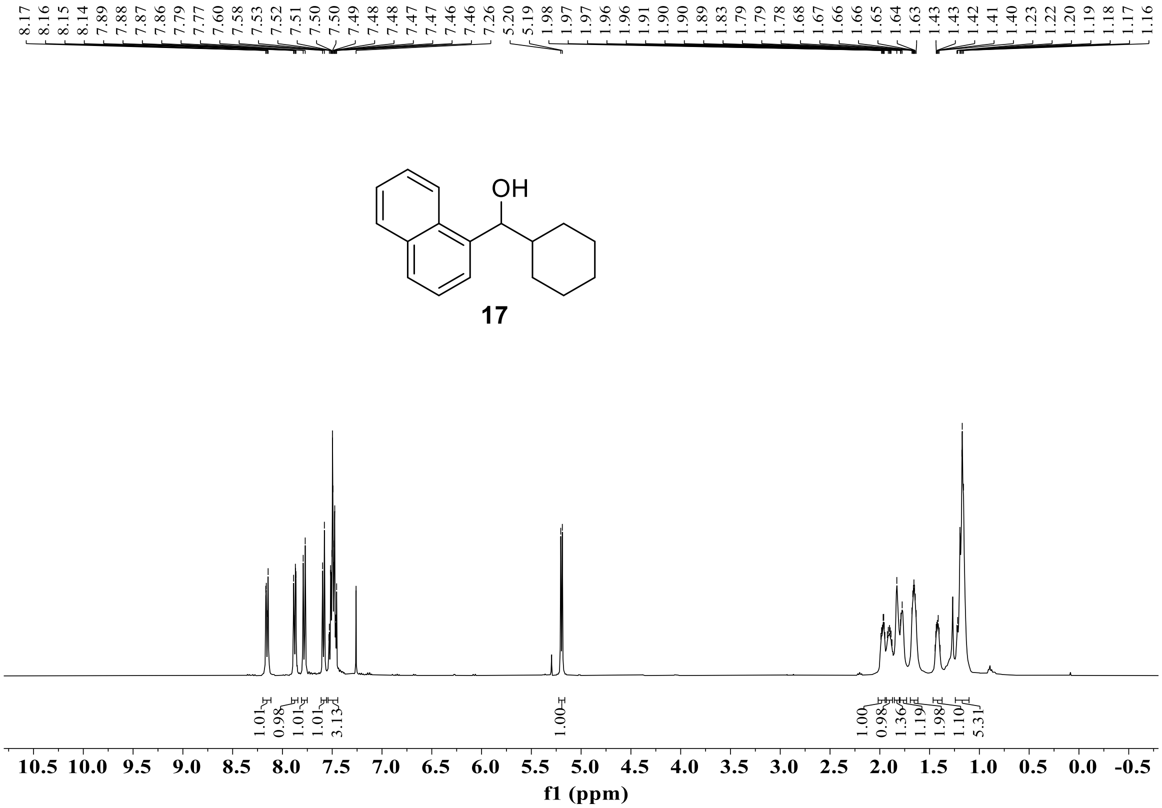
8.17 8.16 8.15 8.14 7.89 7.88 7.87 7.86 7.79 7.77 7.60 7.58 7.53 7.52 7.51 7.50 7.50 7.49 7.48 7.48 7.47 7.47 7.46 7.46 7.26 5.20 5.19 1.98 1.97 1.97 1.96 1.96 1.91 1.90 1.90 1.89 1.83 1.79 1.79 1.78 1.68 1.67 1.66 1.66 1.65 1.64 1.63 1.43 1.43 1.42 1.41 1.40 1.23 1.22 1.20 1.19 1.18 1.17 1.16
OH
17
1.01 0.98 1.01 1.01 3.13 1.00 1.00 0.98 1.36 1.19 1.98 1.10 5.31
10.5 10.0 9.5 9.0 8.5 8.0 7.5 7.0 6.5 6.0 5.5 5.0 4.5 4.0 3.5 3.0 2.5 2.0 1.5 1.0 0.5 0.0 -0.5
f1 (ppm)

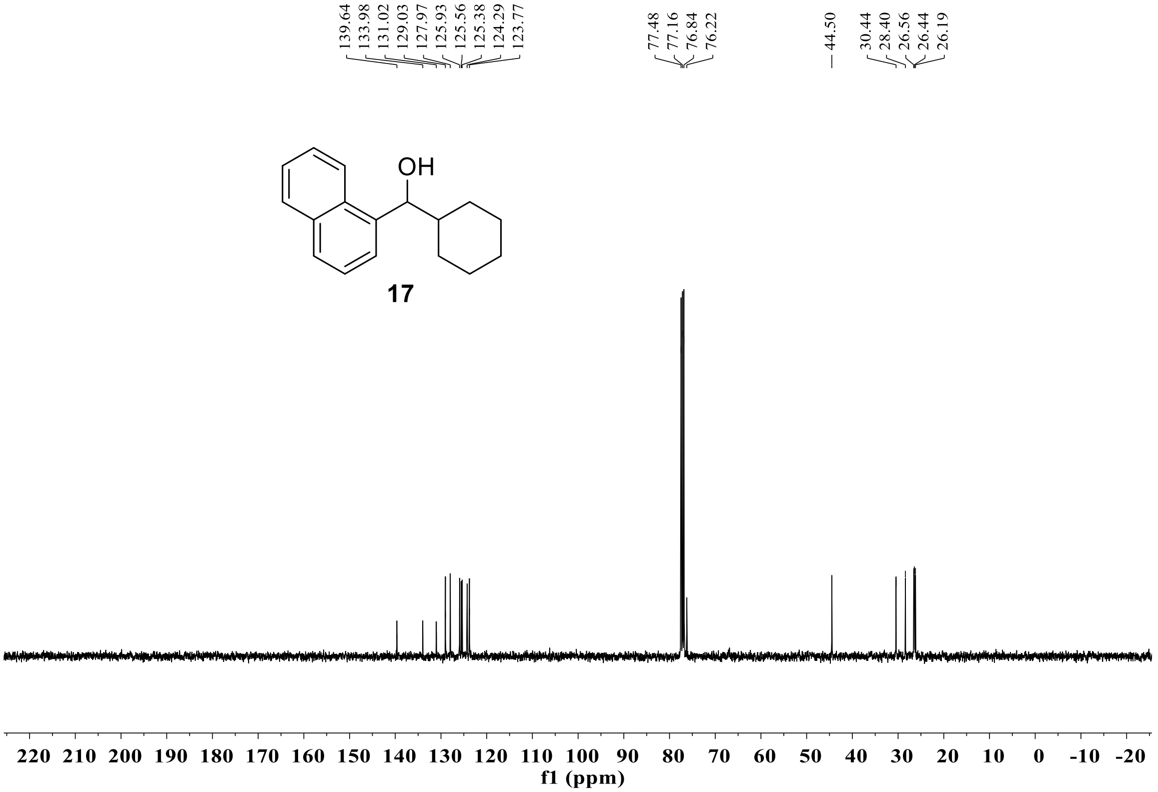
139.64 133.98 131.02 129.03 127.97 125.93 125.56 125.38 124.29 123.77
77.48 77.16 76.84 76.22
44.50
30.44 28.40 26.56 26.44 26.19
OH
17
220 210 200 190 180 170 160 150 140 130 120 110 100 90 80 70 60 50 40 30 20 10 0 -10 -20
f1 (ppm)

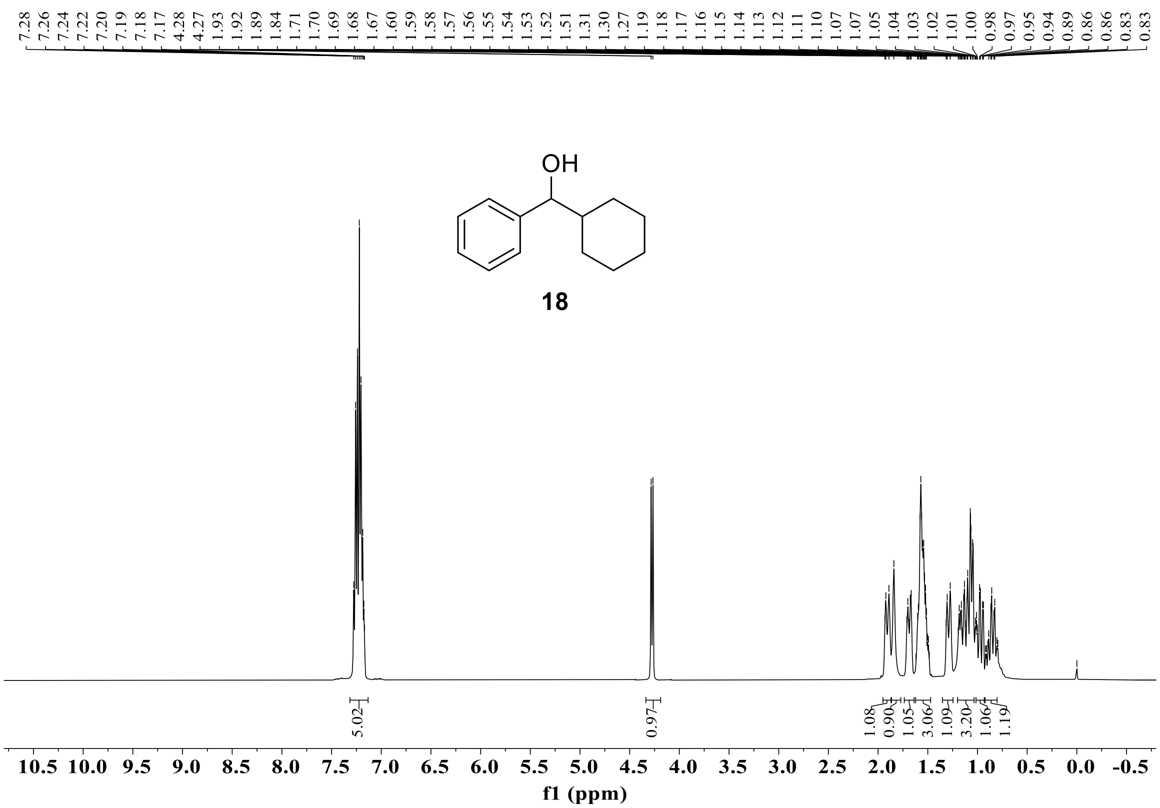
7.28 7.26 7.24 7.22 7.20 7.19 7.18 7.17 4.28 4.27 1.93 1.92 1.89 1.84 1.71 1.70 1.69 1.68 1.67 1.60 1.59 1.58 1.57 1.56 1.55 1.54 1.53 1.52 1.51 1.31 1.30 1.27 1.19 1.18 1.17 1.16 1.15 1.14 1.13 1.12 1.11 1.10 1.07 1.07 1.05 1.04 1.03 1.02 1.01 1.00 0.98 0.97 0.95 0.94 0.89 0.86 0.83 0.83
OH
18
5.02
0.97
1.08
0.90
1.05
3.06
1.09
3.20
1.06
1.19
10.5 10.0 9.5 9.0 8.5 8.0 7.5 7.0 6.5 6.0 5.5 5.0 4.5 4.0 3.5 3.0 2.5 2.0 1.5 1.0 0.5 0.0 -0.5
f1 (ppm)

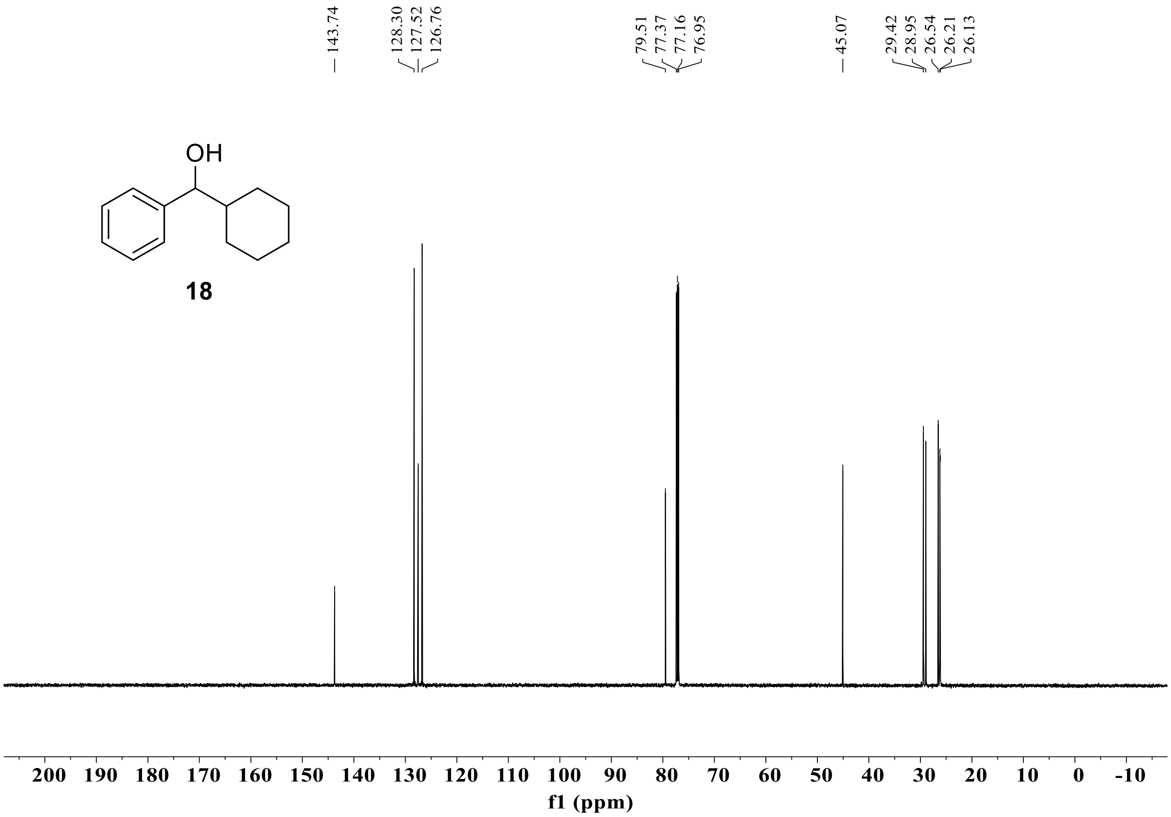
143.74
128.30 127.52 126.76
79.51 77.37 77.16 76.95
45.07
29.42 28.95 26.54 26.21 26.13
OH
18
200 190 180 170 160 150 140 130 120 110 100 90 80 70 60 50 40 30 20 10 0 -10
f1 (ppm)

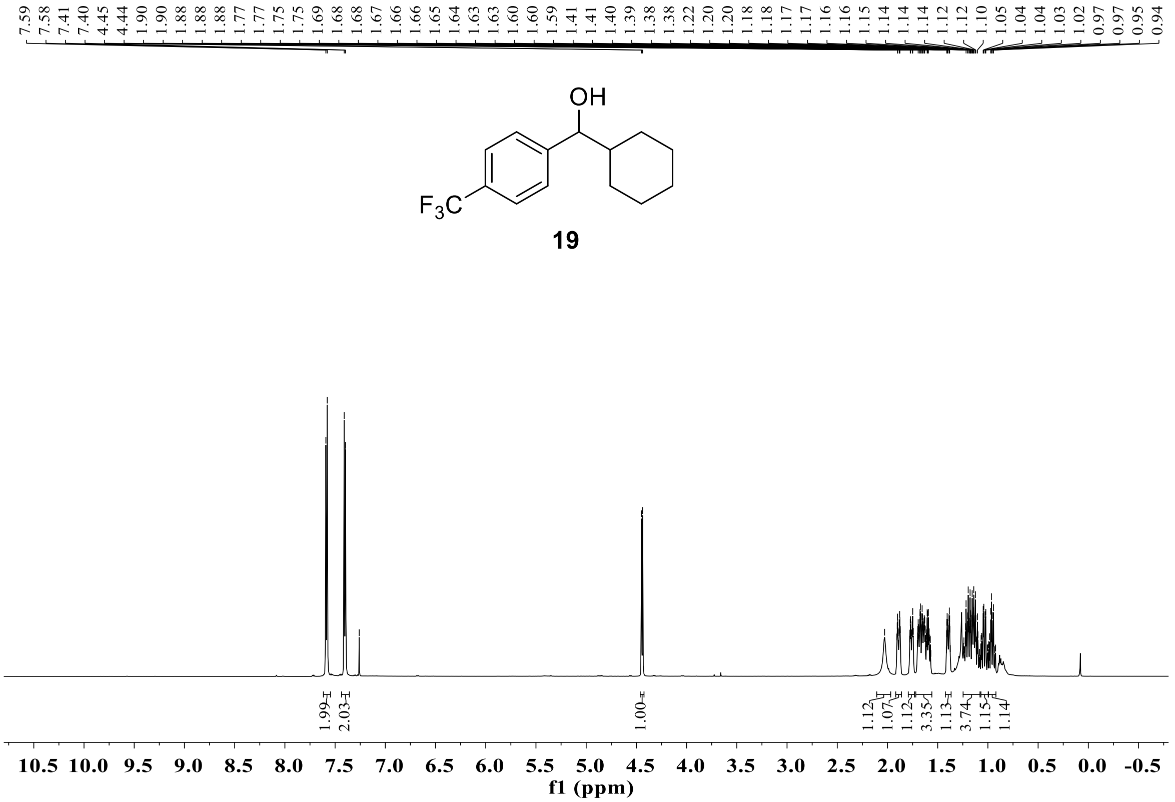
7.59 7.58 7.41 7.40 4.45 4.44 1.90 1.90 1.88 1.88 1.88 1.77 1.77 1.75 1.69 1.68 1.68 1.67 1.66 1.66 1.65 1.64 1.63 1.63 1.60 1.60 1.59 1.41 1.41 1.40 1.39 1.38 1.38 1.22 1.20 1.20 1.18 1.18 1.17 1.17 1.16 1.16 1.15 1.14 1.14 1.14 1.12 1.12 1.10 1.05 1.04 1.04 1.03 1.02 0.97 0.97 0.95 0.94
OH
F3C
19
1.99 2.03 1.00 1.12 1.07 1.12 3.35 1.13 3.74 1.15 1.14
10.5 10.0 9.5 9.0 8.5 8.0 7.5 7.0 6.5 6.0 5.5 5.0 4.5 4.0 3.5 3.0 2.5 2.0 1.5 1.0 0.5 0.0 -0.5
f1 (ppm)

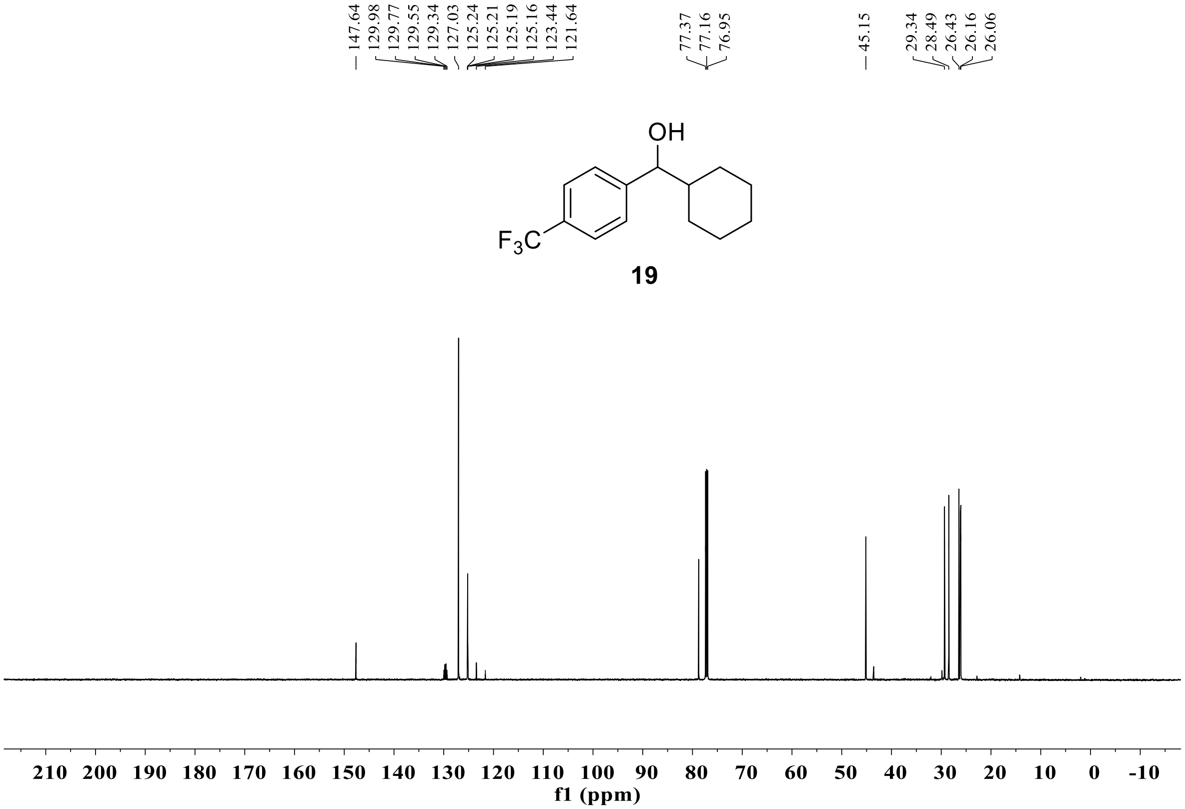
147.64 129.98 129.77 129.55 129.34 127.03 125.24 125.21 125.19 125.16 123.44 121.64
77.37 77.16 76.95
45.15
29.34 28.49 26.43 26.16 26.06
OH
F3C
19
210 200 190 180 170 160 150 140 130 120 110 100 90 80 70 60 50 40 30 20 10 0 -10
f1 (ppm)

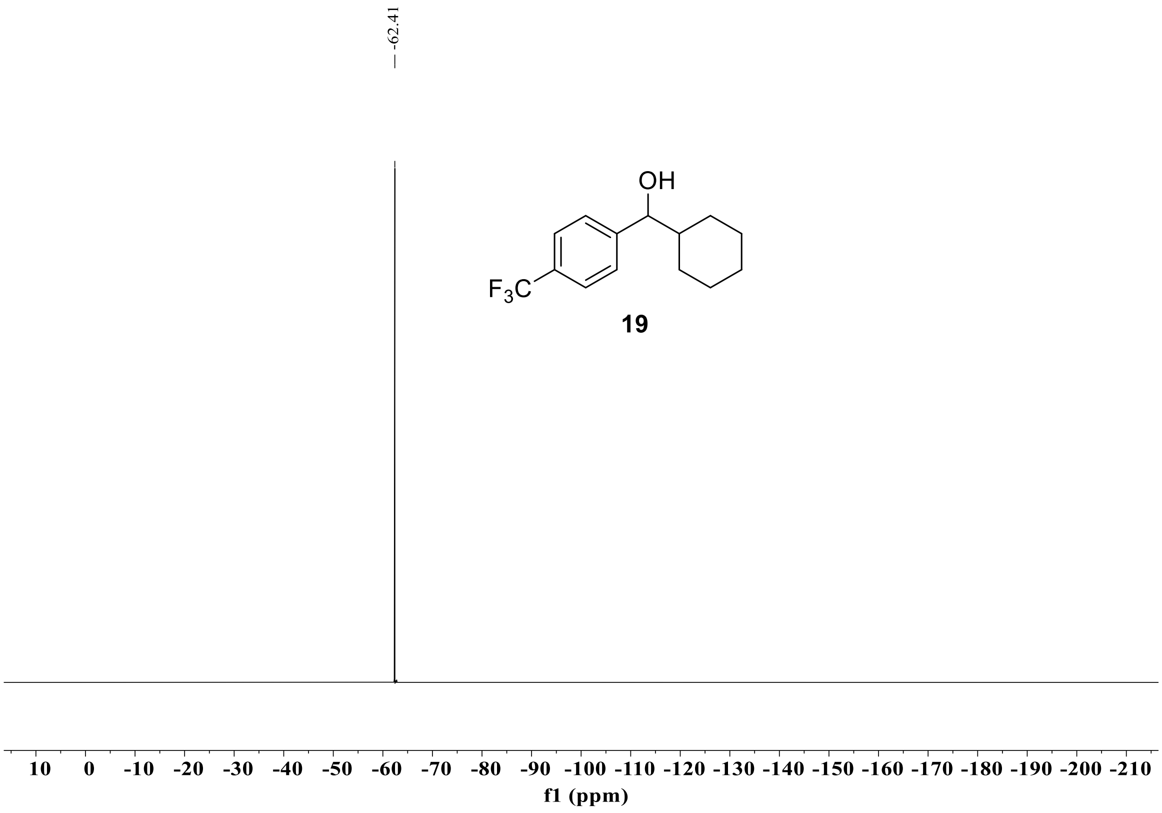

-62.41
OH
F3C
10 0 -10 -20 -30 -40 -50 -60 -70 -80 -90 -100 -110 -120 -130 -140 -150 -160 -170 -180 -190 -200 -210
f1 (ppm)

19

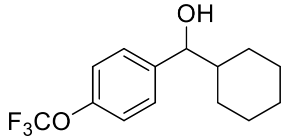

20

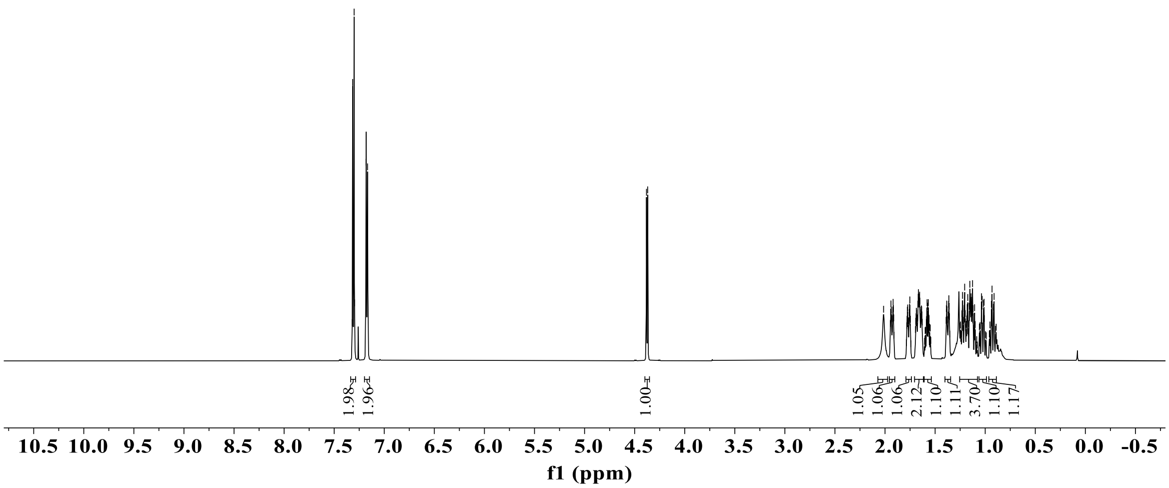

7.32 7.31 7.30 7.30 7.30 7.18 7.18 7.17 4.38 4.37 2.01 1.94 1.92 1.92 1.77 1.76 1.75 1.67 1.67 1.66 1.66 1.66 1.65 1.65 1.64 1.58 1.57 1.57 1.39 1.38 1.37 1.36 1.36 1.23 1.21 1.20 1.18 1.17 1.16 1.15 1.15 1.15 1.14 1.14 1.13 1.13 1.12 1.11 1.04 1.03 1.03 1.02 1.01 0.94 0.93 0.92 0.91
1.98
1.96
1.00
1.05 1.06 1.06 2.12 1.10 1.11 3.70 1.10 1.17
10.5 10.0 9.5 9.0 8.5 8.0 7.5 7.0 6.5 6.0 5.5 5.0 4.5 4.0 3.5 3.0 2.5 2.0 1.5 1.0 0.5 0.0 -0.5
f1 (ppm)

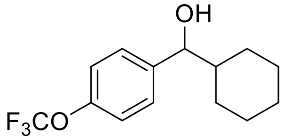

20

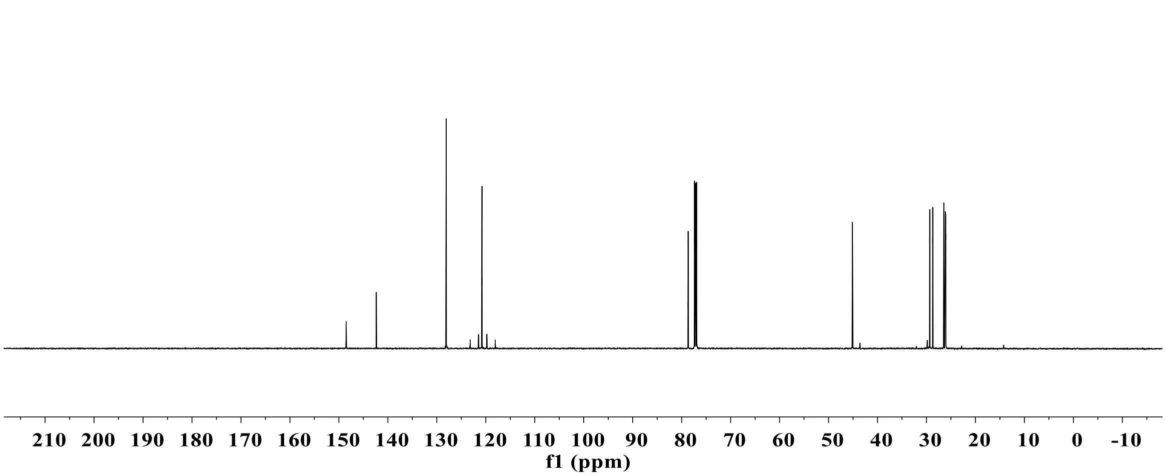

148.52 148.51 142.36 128.08 123.18 121.48 120.78 119.77 118.07 78.68 77.37 77.16 76.95 45.13 29.33 28.73 26.46 26.16 26.06
210 200 190 180 170 160 150 140 130 120 110 100 90 80 70 60 50 40 30 20 10 0 -10
f1 (ppm)

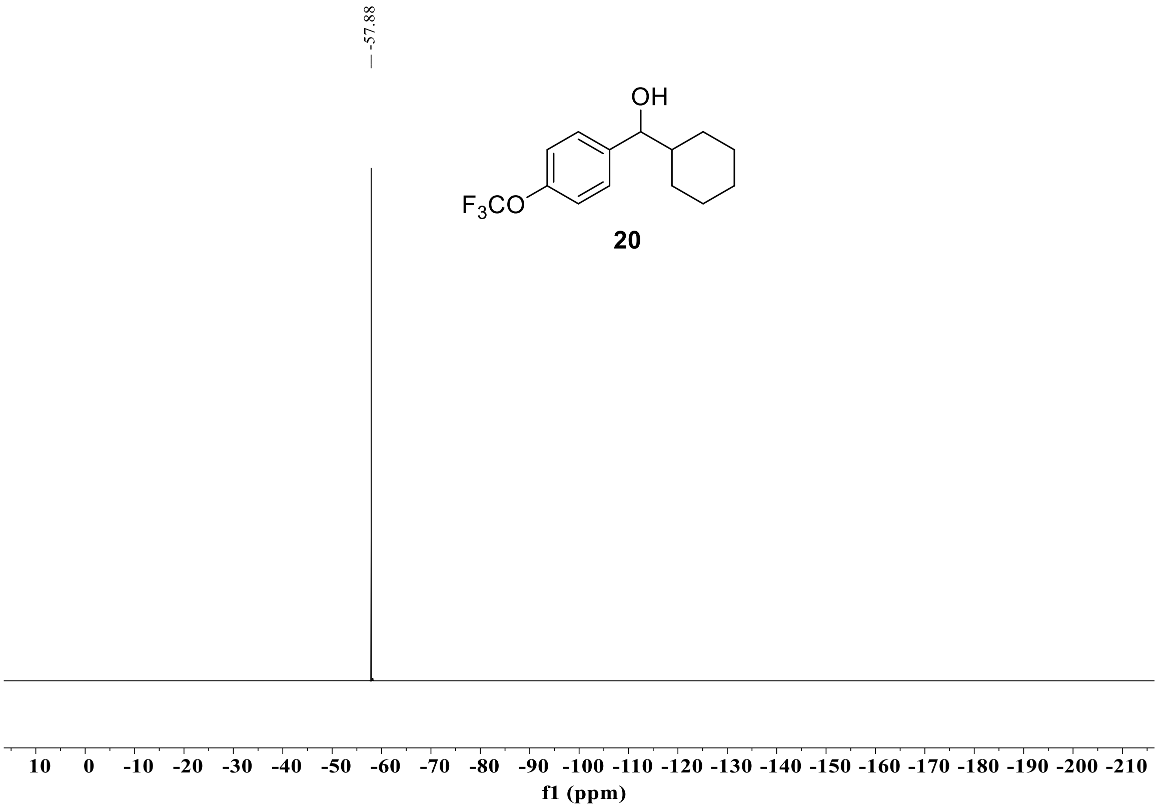
-57.88
OH
F3CO
20
10 0 -10 -20 -30 -40 -50 -60 -70 -80 -90 -100 -110 -120 -130 -140 -150 -160 -170 -180 -190 -200 -210
f1 (ppm)

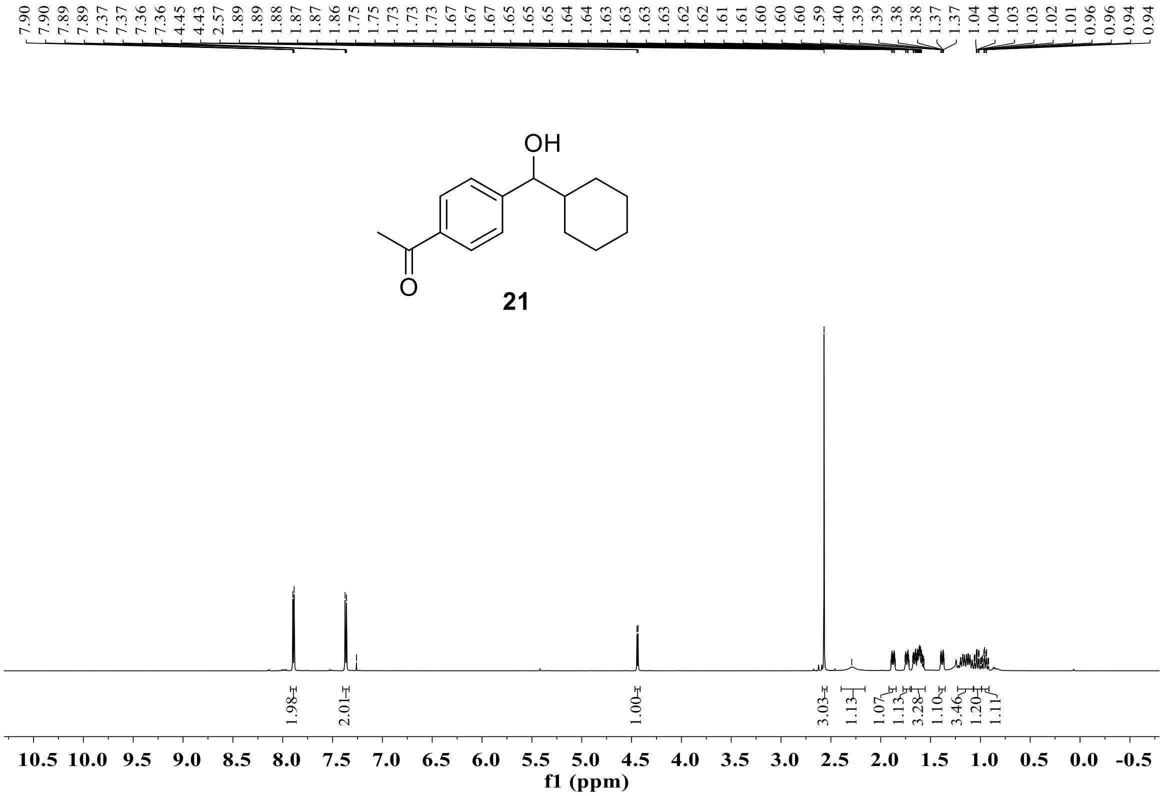
7.90 7.90 7.89 7.89 7.37 7.37 7.36 7.36 4.45 4.43 2.57 1.89 1.89 1.88 1.87 1.87 1.86 1.75 1.75 1.73 1.73 1.73 1.67 1.67 1.67 1.65 1.65 1.65 1.64 1.64 1.63 1.63 1.63 1.63 1.62 1.62 1.61 1.61 1.60 1.60 1.60 1.59 1.40 1.39 1.39 1.38 1.38 1.37 1.37 1.04 1.04 1.03 1.03 1.02 1.01 0.96 0.96 0.94 0.94
OH
O
21
1.98
2.01
1.00
3.03
1.13
1.07
1.13
3.28
1.10
3.46
1.20
1.11
10.5 10.0 9.5 9.0 8.5 8.0 7.5 7.0 6.5 6.0 5.5 5.0 4.5 4.0 3.5 3.0 2.5 2.0 1.5 1.0 0.5 0.0 -0.5
f1 (ppm)

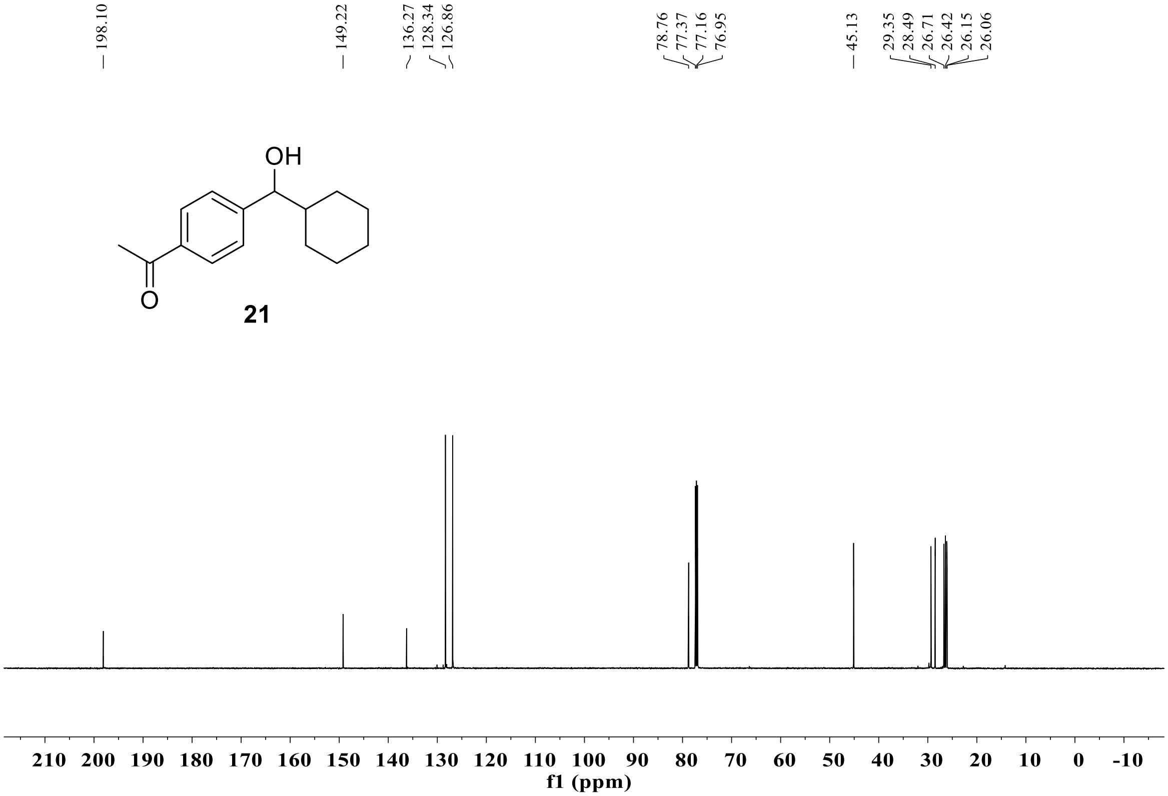
198.10
149.22
136.27 128.34 126.86
78.76 77.37 77.16 76.95
45.13
29.35 28.49 26.71 26.42 26.15 26.06
OH
O
21
210 200 190 180 170 160 150 140 130 120 110 100 90 80 70 60 50 40 30 20 10 0 -10
f1 (ppm)

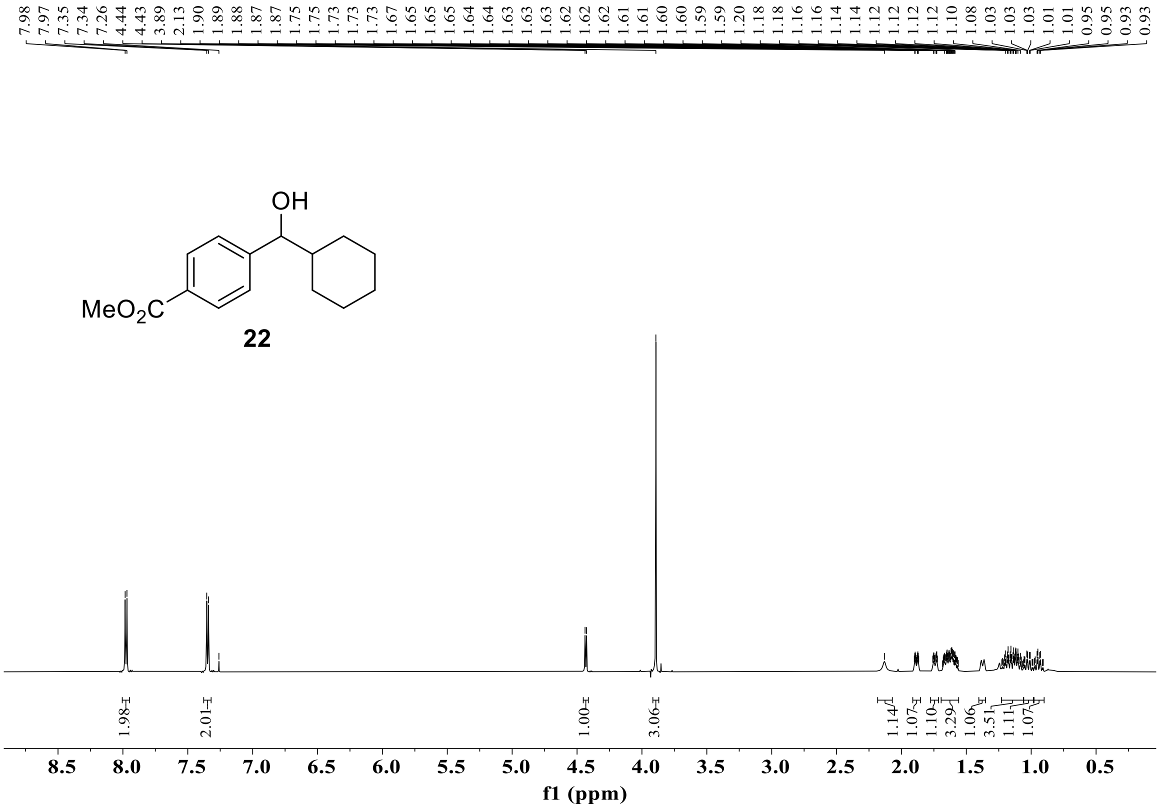
7.98 7.97 7.35 7.34 7.26 4.44 4.43 3.89 2.13 1.90 1.89 1.88 1.87 1.87 1.75 1.75 1.73 1.73 1.73 1.67 1.65 1.65 1.64 1.64 1.63 1.63 1.63 1.62 1.62 1.61 1.61 1.60 1.60 1.59 1.59 1.20 1.18 1.18 1.16 1.16 1.14 1.14 1.12 1.12 1.12 1.12 1.10 1.08 1.03 1.03 1.03 1.01 1.01 0.95 0.95 0.93 0.93
OH
MeO2C
22
1.98 2.01 1.00 3.06 1.14 1.07 1.10 3.29 1.06 3.51 1.11 1.07
8.5 8.0 7.5 7.0 6.5 6.0 5.5 5.0 4.5 4.0 3.5 3.0 2.5 2.0 1.5 1.0 0.5
f1 (ppm)

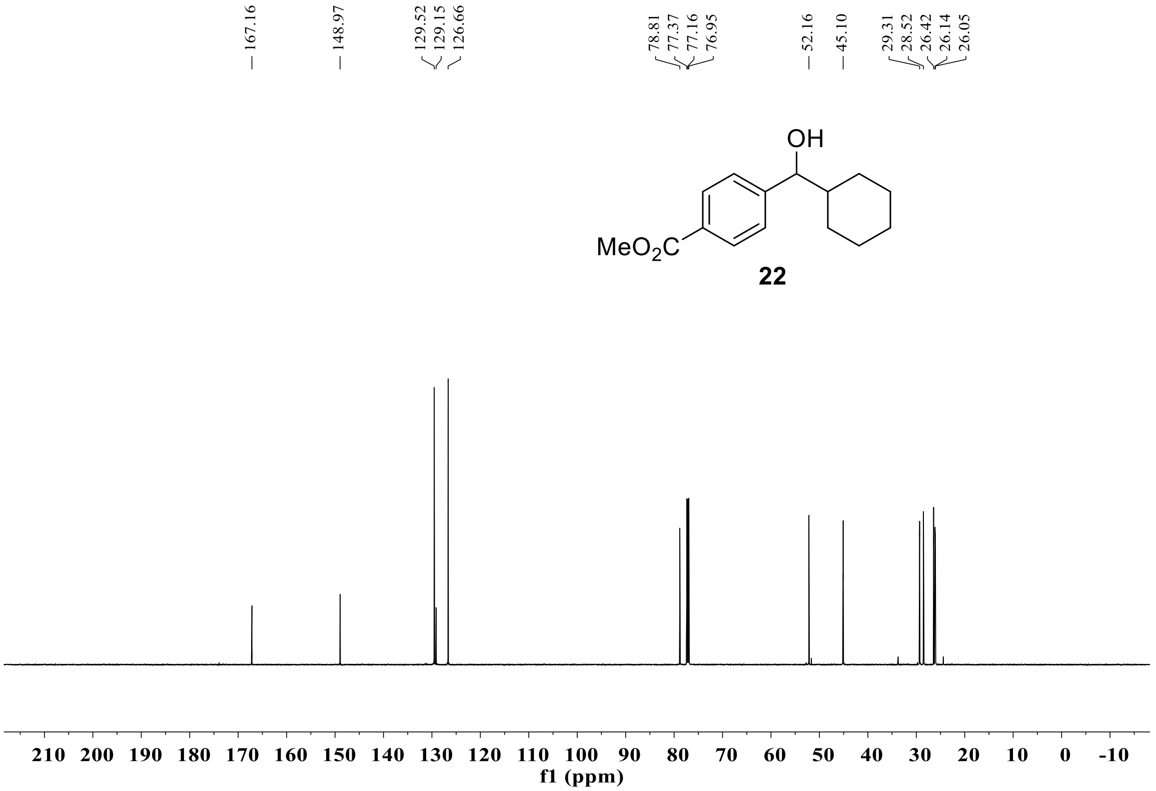
167.16
148.97
129.52 129.15 126.66
78.81 77.37 77.16 76.95
52.16
45.10
29.31 28.52 26.42 26.14 26.05
OH
MeO2C
22
210 200 190 180 170 160 150 140 130 120 110 100 90 80 70 60 50 40 30 20 10 0 -10
f1 (ppm)

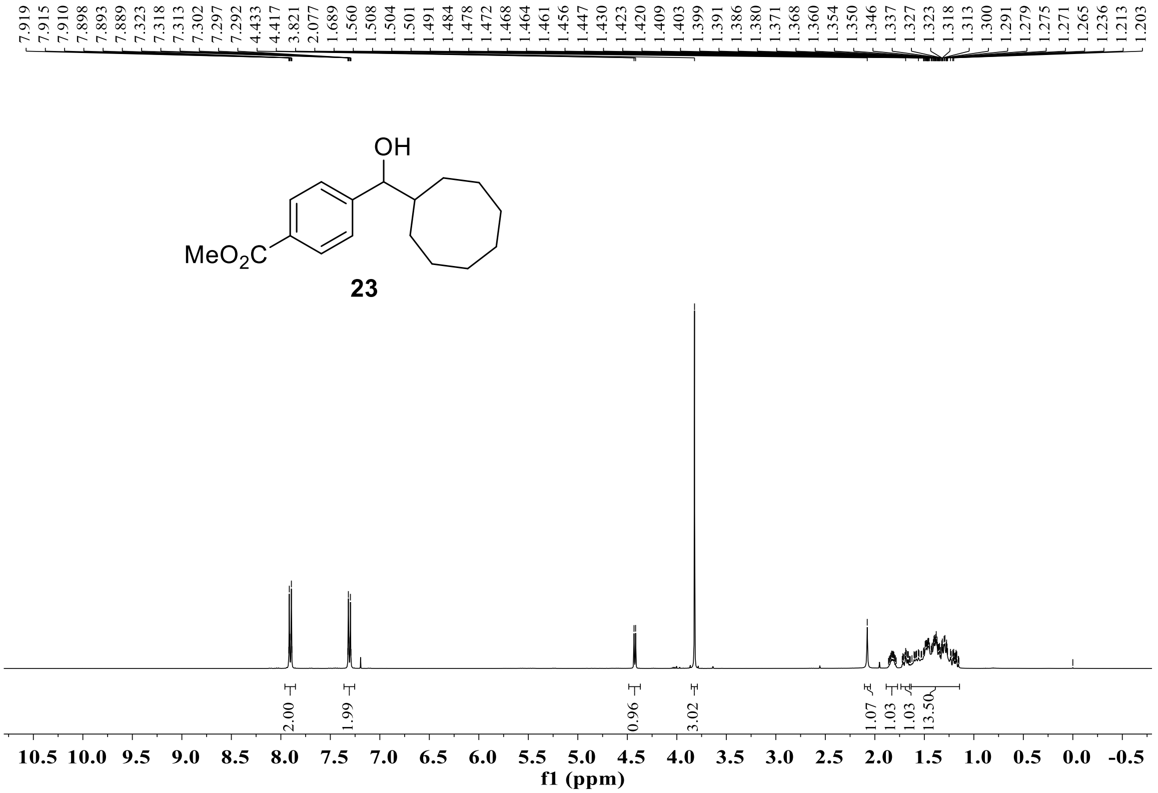

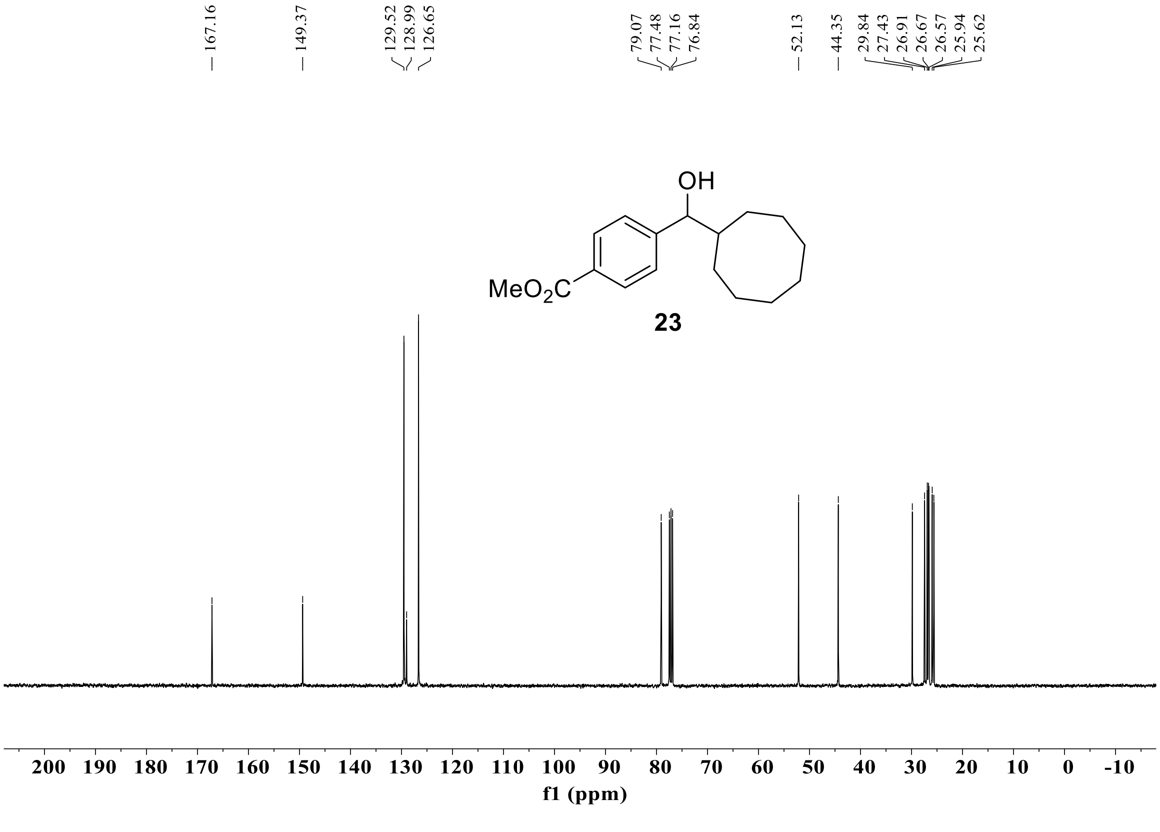

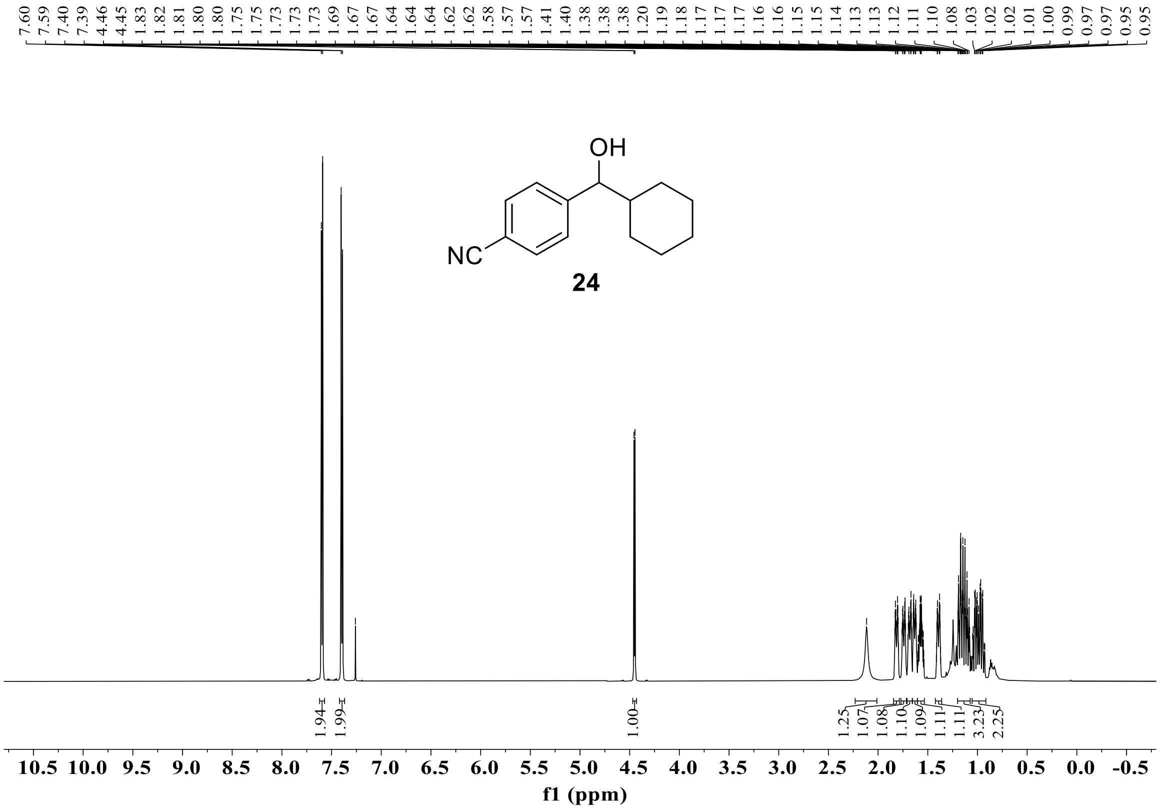

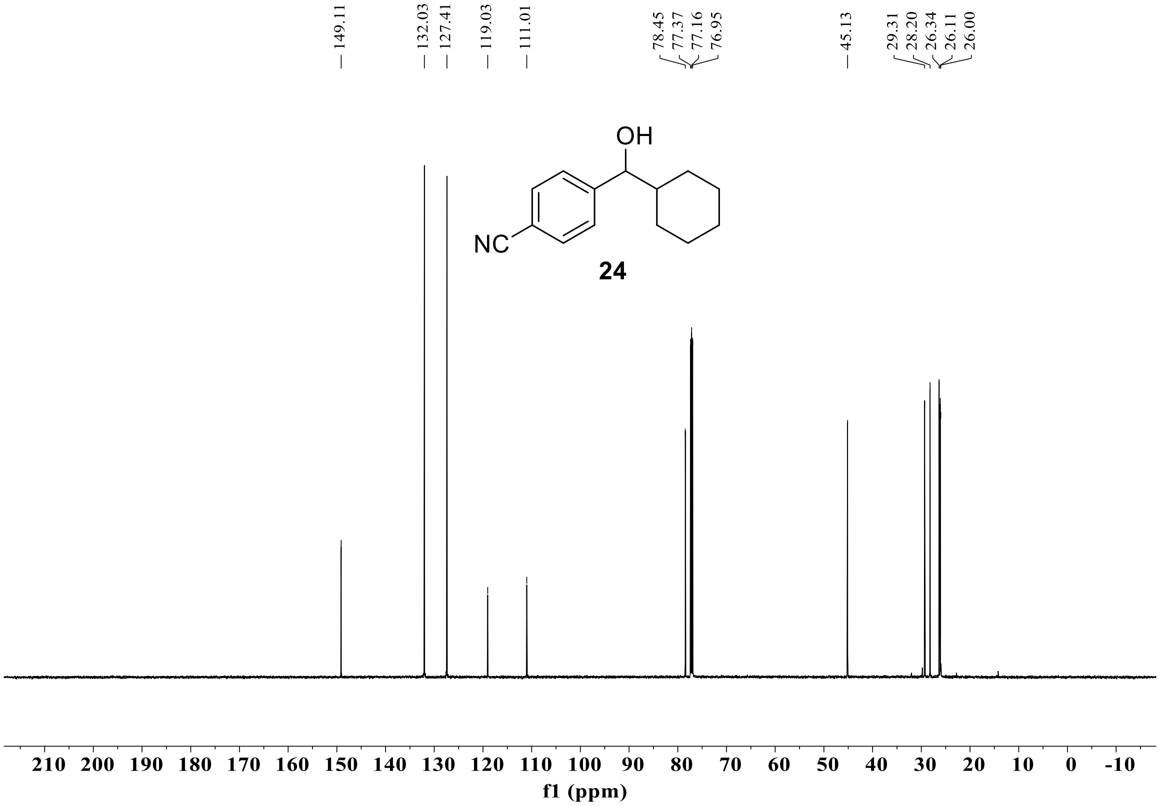

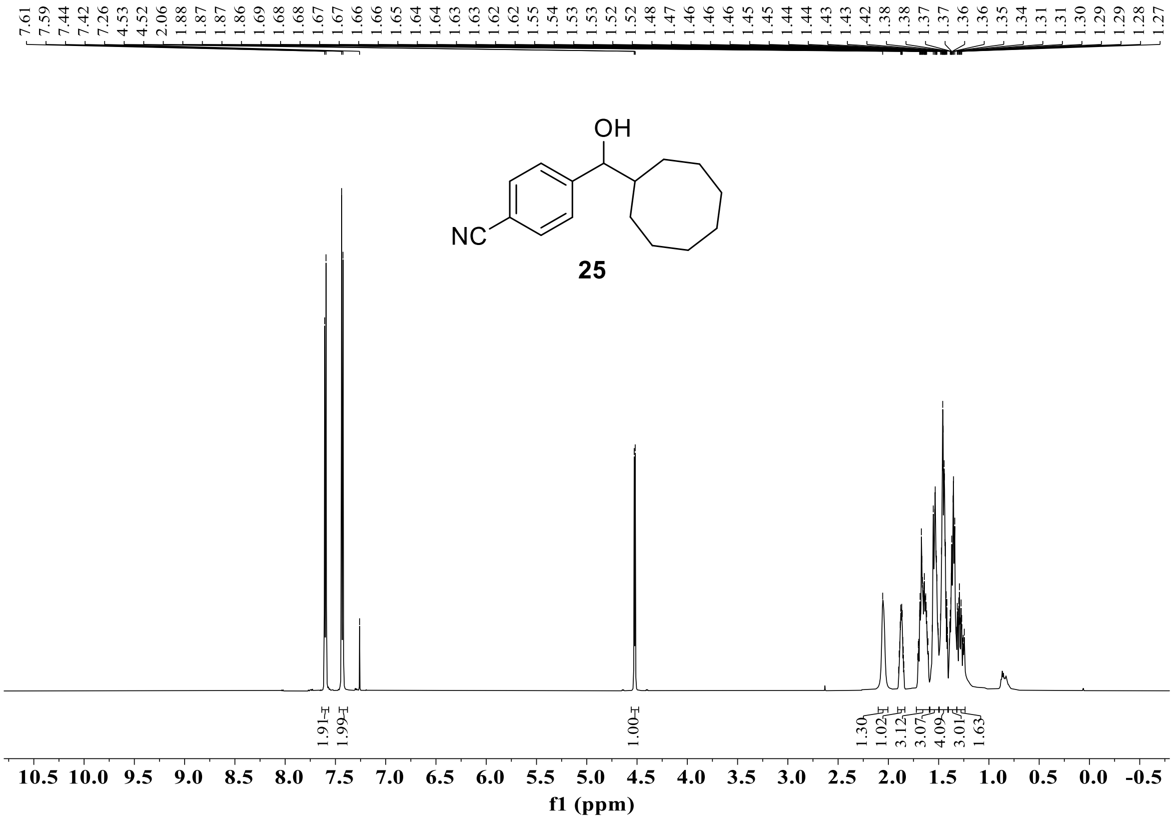
7.61 7.59 7.44 7.42 7.26 4.53 4.52 2.06 1.88 1.87 1.87 1.86 1.69 1.68 1.68 1.67 1.67 1.66 1.66 1.65 1.64 1.64 1.63 1.63 1.62 1.62 1.55 1.54 1.53 1.53 1.52 1.52 1.48 1.47 1.46 1.46 1.45 1.45 1.44 1.44 1.43 1.43 1.42 1.38 1.38 1.37 1.37 1.36 1.36 1.35 1.34 1.31 1.31 1.30 1.29 1.29 1.28 1.27
OH
NC
25
1.91 1.99 1.00 1.30 1.02 3.12 3.07 4.09 3.01 1.63
10.5 10.0 9.5 9.0 8.5 8.0 7.5 7.0 6.5 6.0 5.5 5.0 4.5 4.0 3.5 3.0 2.5 2.0 1.5 1.0 0.5 0.0 -0.5
f1 (ppm)

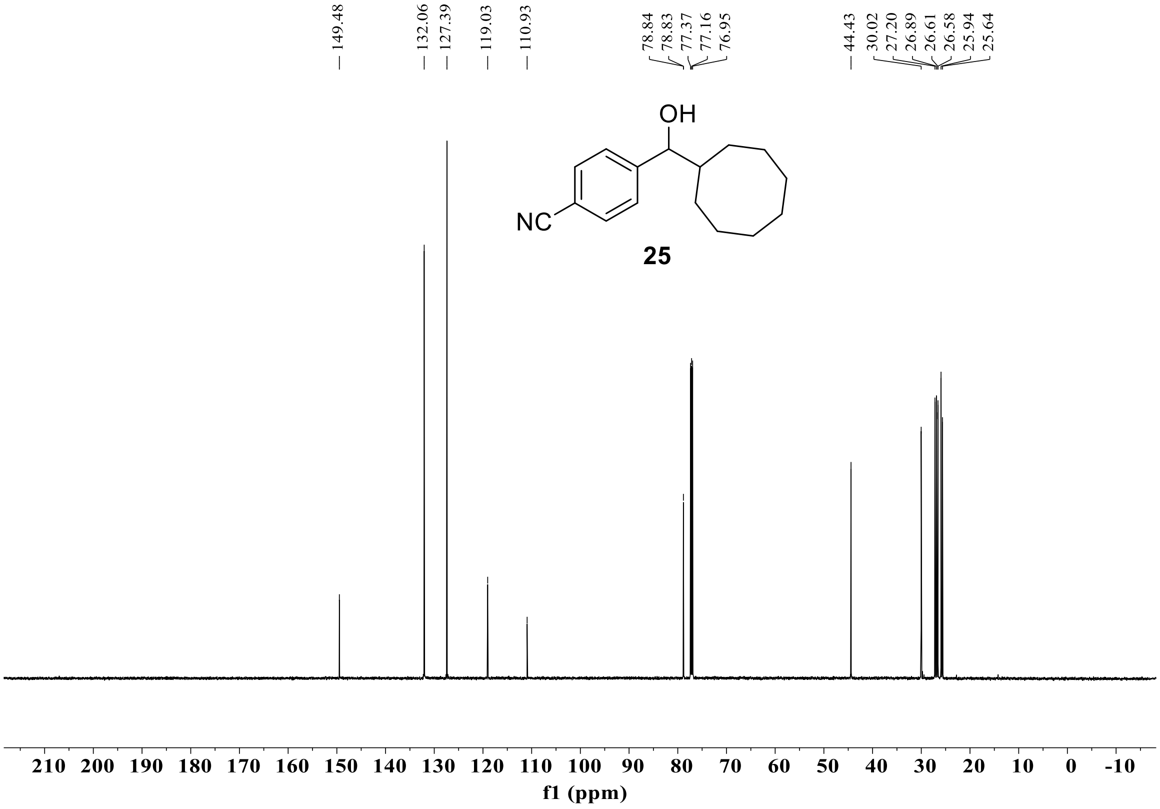
149.48 132.06 127.39 119.03 110.93 78.84 78.83 77.37 77.16 76.95 44.43 30.02 27.20 26.89 26.61 26.58 25.94 25.64
OH
NC
25
210 200 190 180 170 160 150 140 130 120 110 100 90 80 70 60 50 40 30 20 10 0 -10
f1 (ppm)

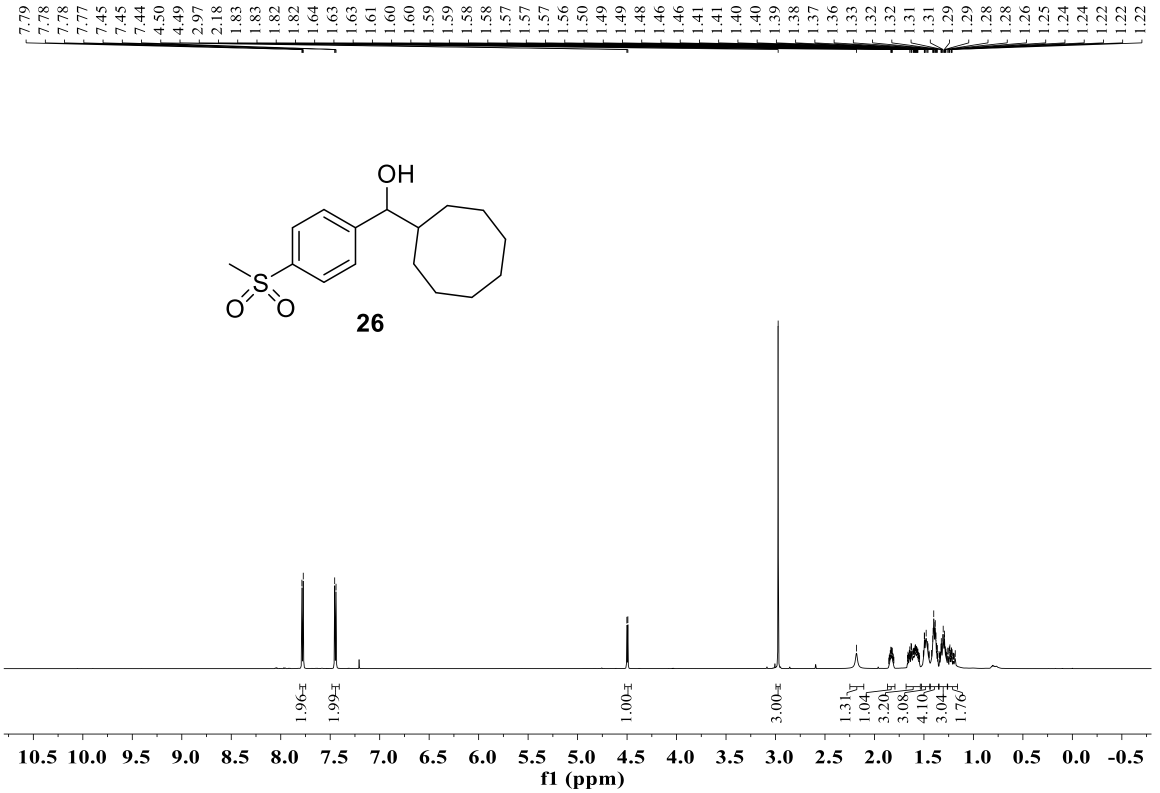
7.79 7.78 7.78 7.77 7.45 7.45 7.44 4.50 4.49 2.97 2.18 1.83 1.83 1.82 1.64 1.63 1.63 1.61 1.60 1.60 1.59 1.59 1.58 1.58 1.57 1.57 1.57 1.56 1.50 1.49 1.49 1.48 1.46 1.46 1.41 1.41 1.40 1.40 1.39 1.39 1.38 1.37 1.36 1.33 1.32 1.32 1.31 1.31 1.29 1.29 1.28 1.28 1.26 1.25 1.24 1.24 1.22 1.22 1.22
OH
O S O
26
1.96 1.99 1.00 3.00 1.31 1.04 3.20 3.08 4.10 3.04 1.76
10.5 10.0 9.5 9.0 8.5 8.0 7.5 7.0 6.5 6.0 5.5 5.0 4.5 4.0 3.5 3.0 2.5 2.0 1.5 1.0 0.5 0.0 -0.5
f1 (ppm)

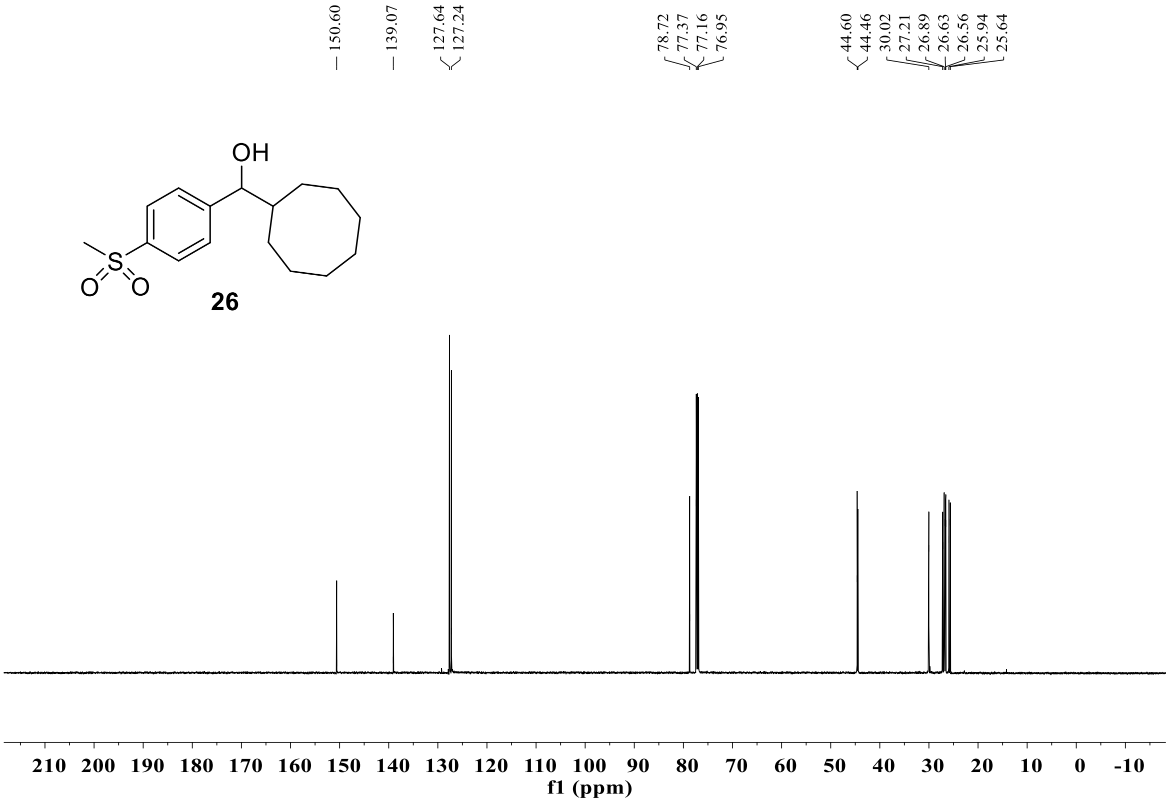
150.60 139.07 127.64 127.24 78.72 77.37 77.16 76.95 44.60 44.46 30.02 27.21 26.89 26.63 26.56 25.94 25.64
OH
O S O
26
210 200 190 180 170 160 150 140 130 120 110 100 90 80 70 60 50 40 30 20 10 0 -10
f1 (ppm)

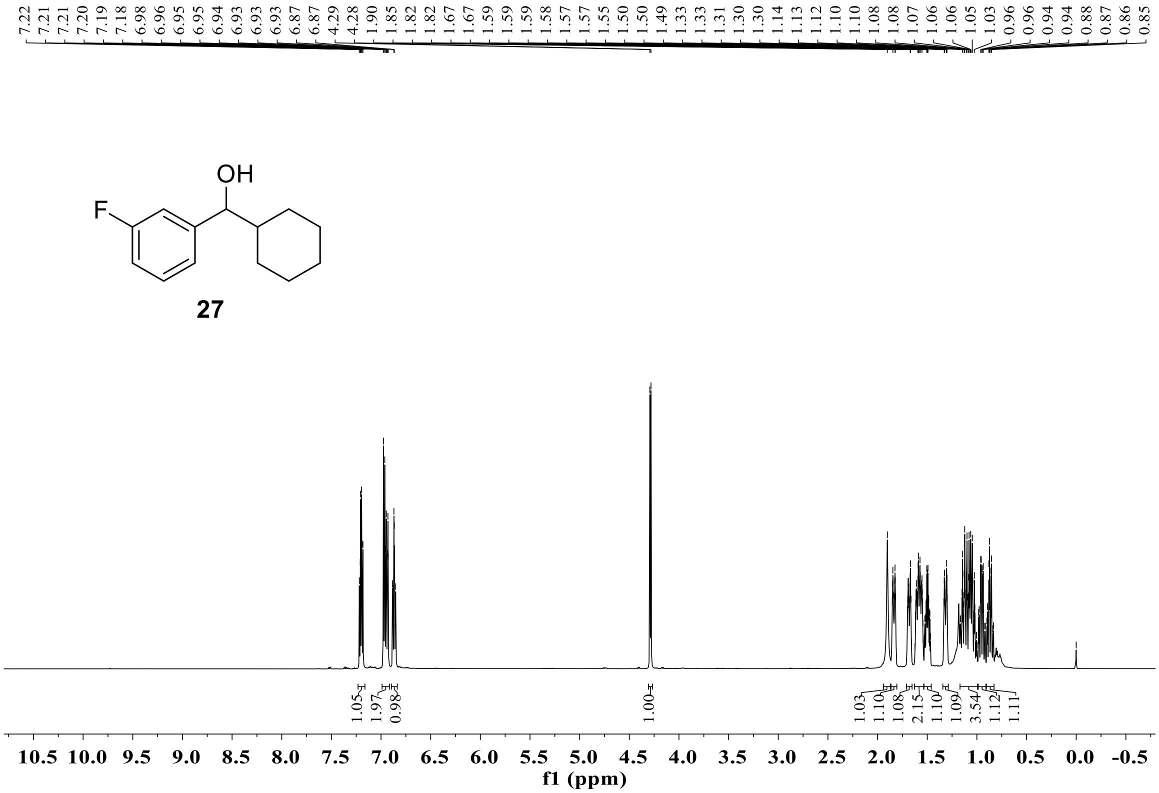
7.22 7.21 7.21 7.20 7.19 7.18 6.98 6.96 6.95 6.95 6.94 6.93 6.93 6.93 6.87 6.87 4.29 4.28 1.90 1.85 1.82 1.82 1.67 1.67 1.59 1.59 1.59 1.58 1.57 1.57 1.55 1.50 1.50 1.49 1.33 1.33 1.31 1.30 1.30 1.14 1.13 1.12 1.10 1.10 1.08 1.08 1.07 1.06 1.06 1.05 1.03 0.96 0.96 0.94 0.94 0.88 0.87 0.86 0.85
OH
F
27
1.05 1.97 0.98
1.00
1.03 1.10 1.08 2.15 1.10 1.09 3.54 1.12 1.11
10.5 10.0 9.5 9.0 8.5 8.0 7.5 7.0 6.5 6.0 5.5 5.0 4.5 4.0 3.5 3.0 2.5 2.0 1.5 1.0 0.5 0.0 -0.5
f1 (ppm)

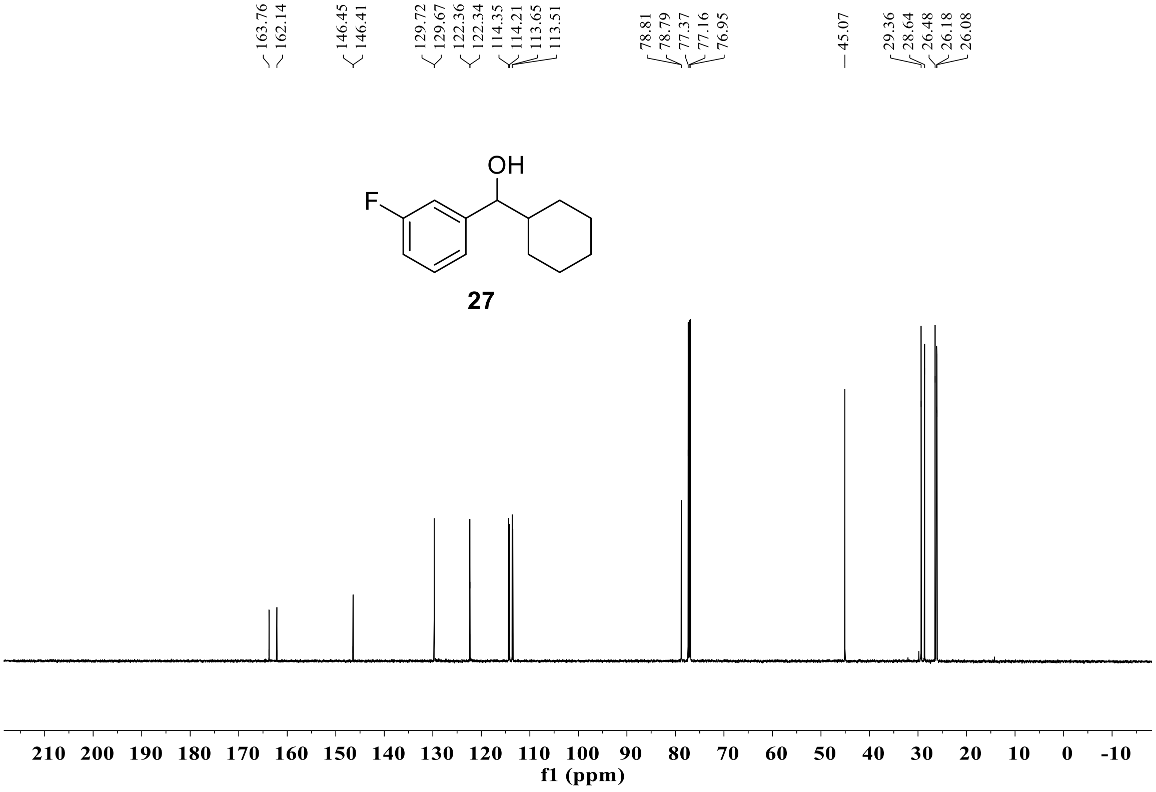
163.76 162.14 146.45 146.41 129.72 129.67 122.36 122.34 114.35 114.21 113.65 113.51 78.81 78.79 77.37 77.16 76.95 45.07 29.36 28.64 26.48 26.18 26.08
OH
F
27
210 200 190 180 170 160 150 140 130 120 110 100 90 80 70 60 50 40 30 20 10 0 -10
f1 (ppm)

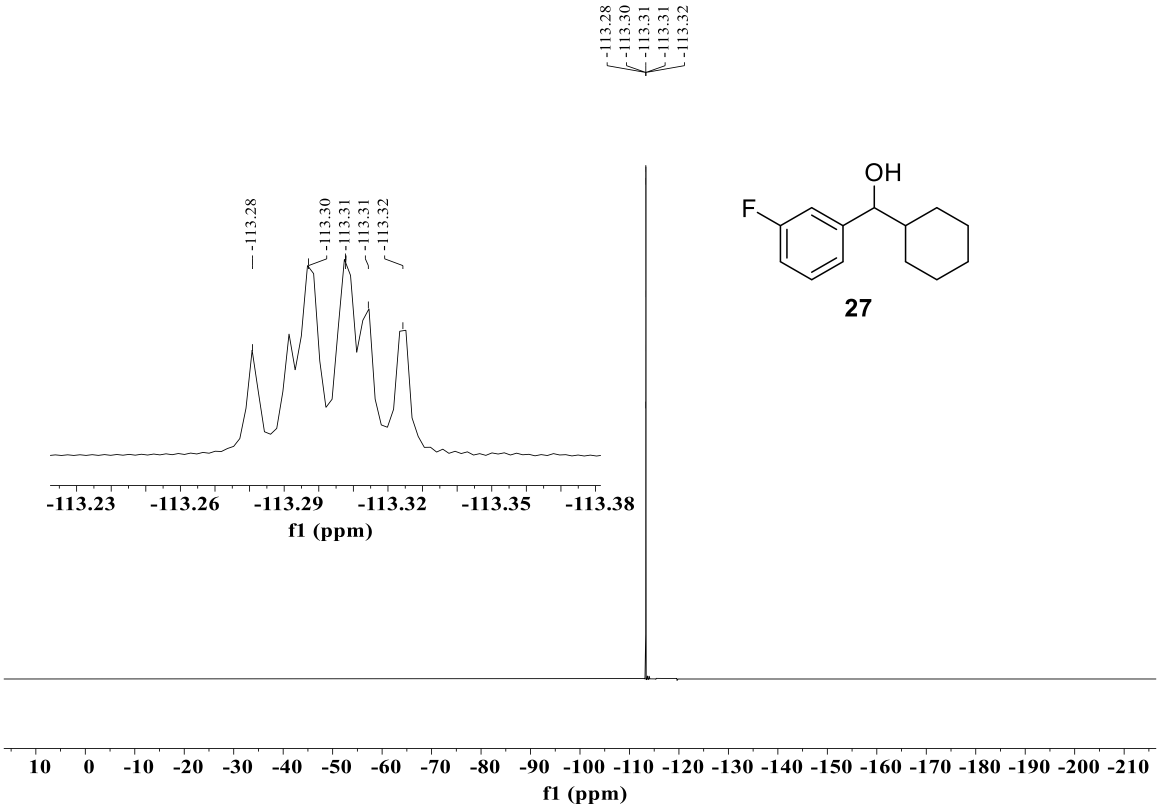
-113.28
-113.30
-113.31
-113.31
-113.32
OH
F
27
-113.23 -113.26 -113.29 -113.32 -113.35 -113.38
f1 (ppm)
10 0 -10 -20 -30 -40 -50 -60 -70 -80 -90 -100 -110 -120 -130 -140 -150 -160 -170 -180 -190 -200 -210
f1 (ppm)

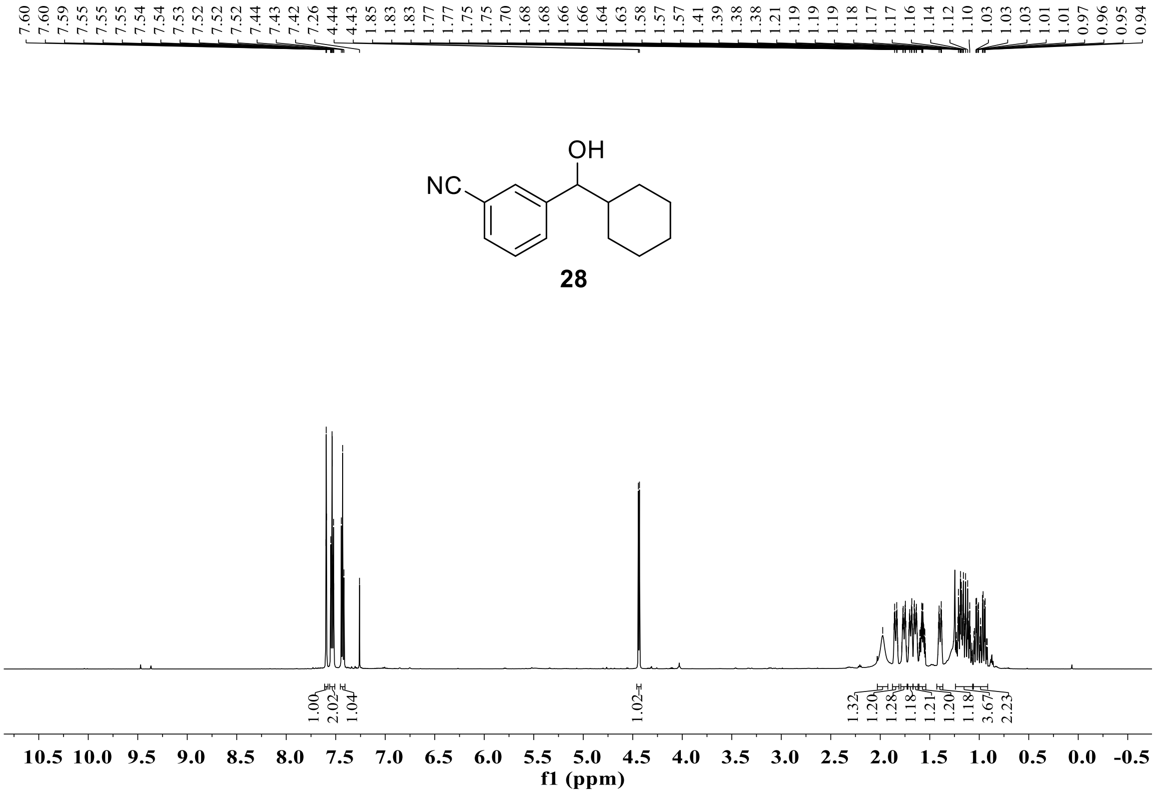

7.60 7.60 7.59 7.55 7.55 7.55 7.54 7.54 7.53 7.52 7.52 7.52 7.44 7.43 7.42 7.26 4.44 4.43 1.85 1.83 1.83 1.77 1.77 1.75 1.75 1.70 1.68 1.68 1.66 1.64 1.63 1.58 1.57 1.57 1.41 1.39 1.38 1.38 1.21 1.19 1.19 1.19 1.18 1.17 1.17 1.16 1.14 1.12 1.10 1.03 1.03 1.03 1.01 1.01 0.97 0.96 0.95 0.94
OH
NC
28
1.00 2.02 1.04
1.02
1.32 1.20 1.28 1.18 1.21 1.20 1.18 3.67 2.23
10.5 10.0 9.5 9.0 8.5 8.0 7.5 7.0 6.5 6.0 5.5 5.0 4.5 4.0 3.5 3.0 2.5 2.0 1.5 1.0 0.5 0.0 -0.5
f1 (ppm)

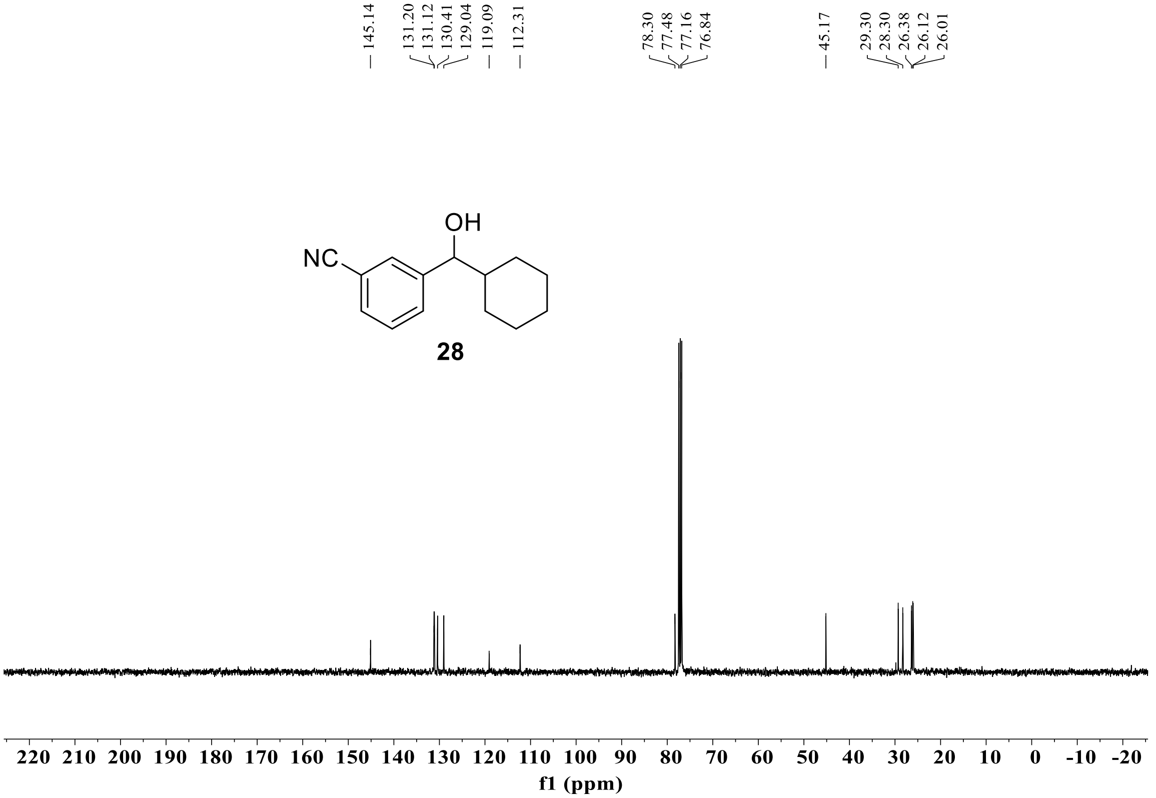

145.14 131.20 131.12 130.41 129.04 119.09 112.31 78.30 77.48 77.16 76.84 45.17 29.30 28.30 26.38 26.12 26.01
OH
NC
28
220 210 200 190 180 170 160 150 140 130 120 110 100 90 80 70 60 50 40 30 20 10 0 -10 -20
f1 (ppm)

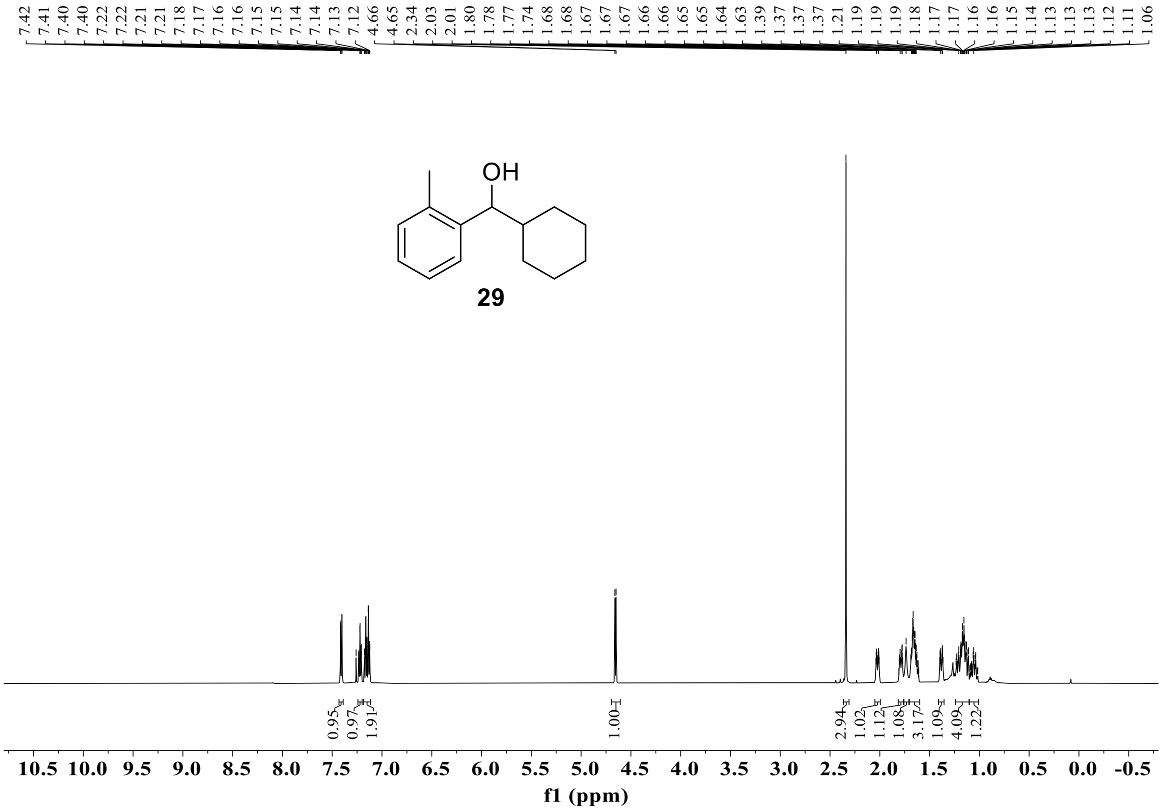
7.42 7.41 7.40 7.40 7.22 7.22 7.21 7.21 7.18 7.17 7.16 7.16 7.15 7.15 7.14 7.14 7.13 7.12 4.66 4.65 2.34 2.03 2.01 1.80 1.78 1.77 1.74 1.68 1.68 1.67 1.67 1.67 1.66 1.66 1.65 1.65 1.64 1.63 1.39 1.37 1.37 1.37 1.21 1.19 1.19 1.19 1.18 1.17 1.17 1.16 1.16 1.15 1.14 1.13 1.13 1.13 1.12 1.11 1.06
OH
29
0.95 0.97 1.91 1.00 2.94 1.02 1.12 1.08 3.17 1.09 4.09 1.22
10.5 10.0 9.5 9.0 8.5 8.0 7.5 7.0 6.5 6.0 5.5 5.0 4.5 4.0 3.5 3.0 2.5 2.0 1.5 1.0 0.5 0.0 -0.5
f1 (ppm)

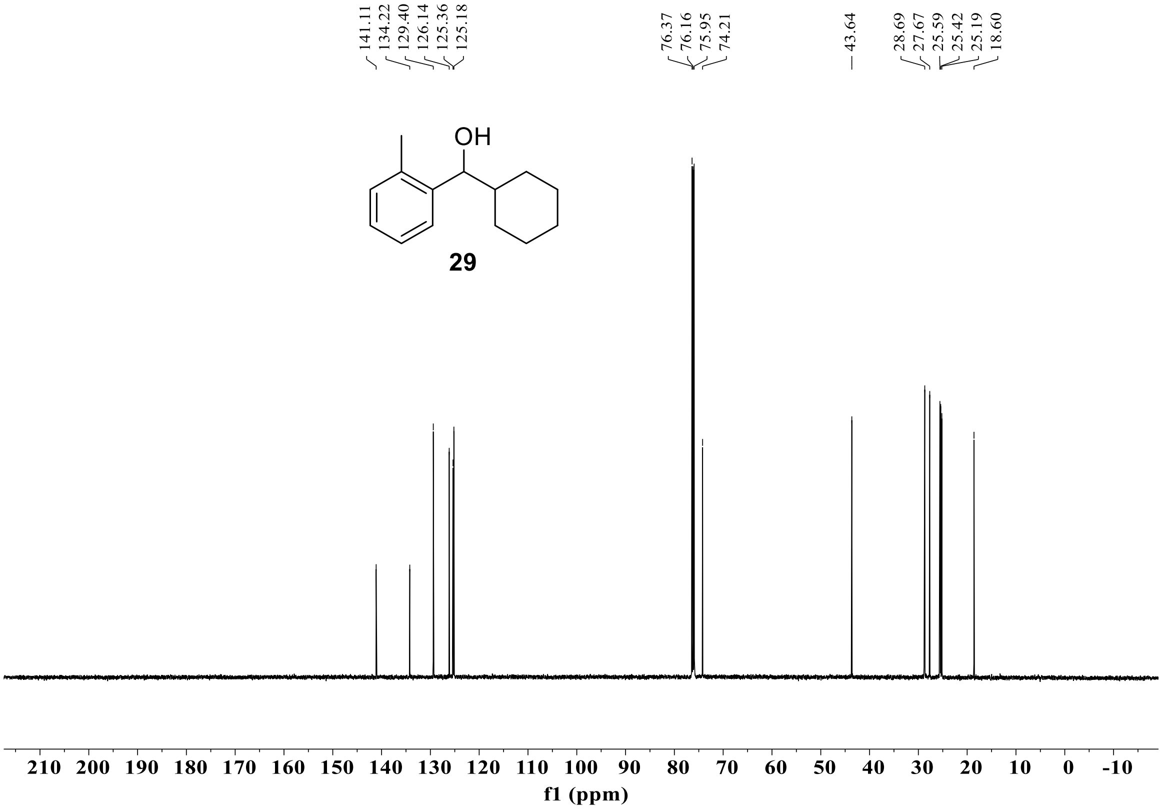
141.11 134.22 129.40 126.14 125.36 125.18
76.37 76.16 75.95 74.21
43.64
28.69 27.67 25.59 25.42 25.19 18.60
OH
29
210 200 190 180 170 160 150 140 130 120 110 100 90 80 70 60 50 40 30 20 10 0 -10
f1 (ppm)

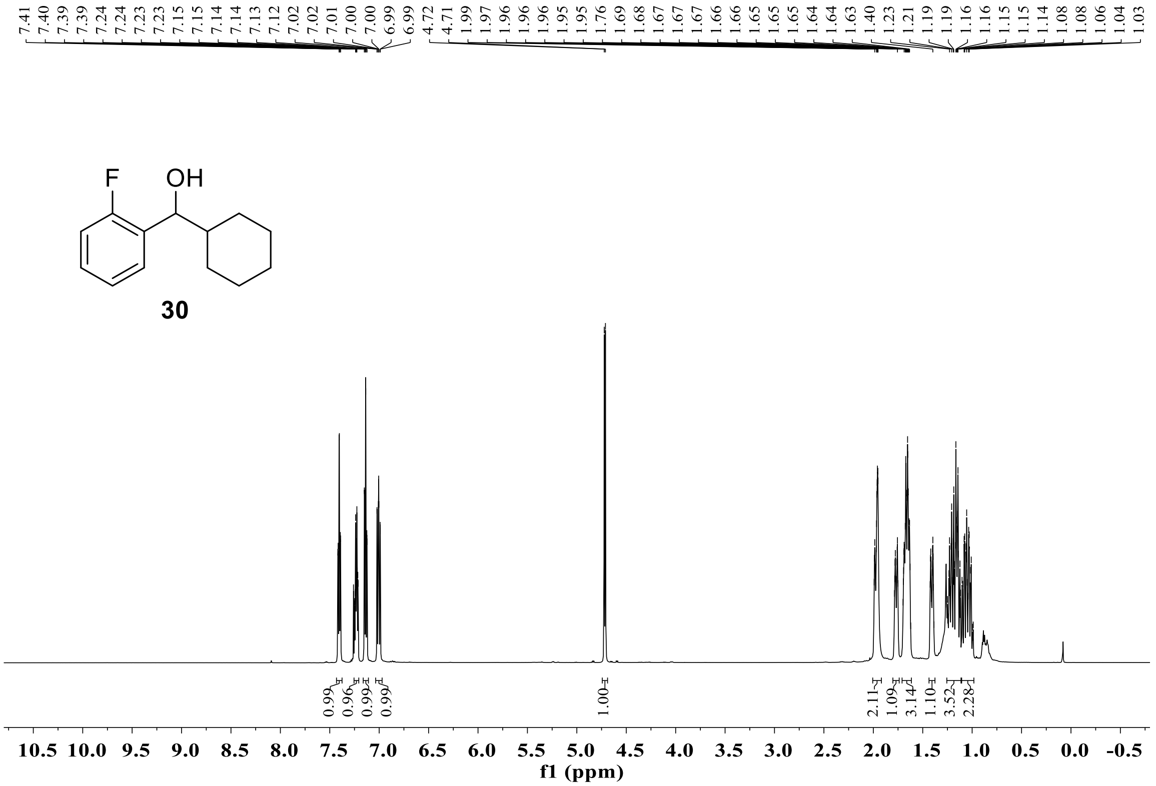
F
OH
30
f1 (ppm)

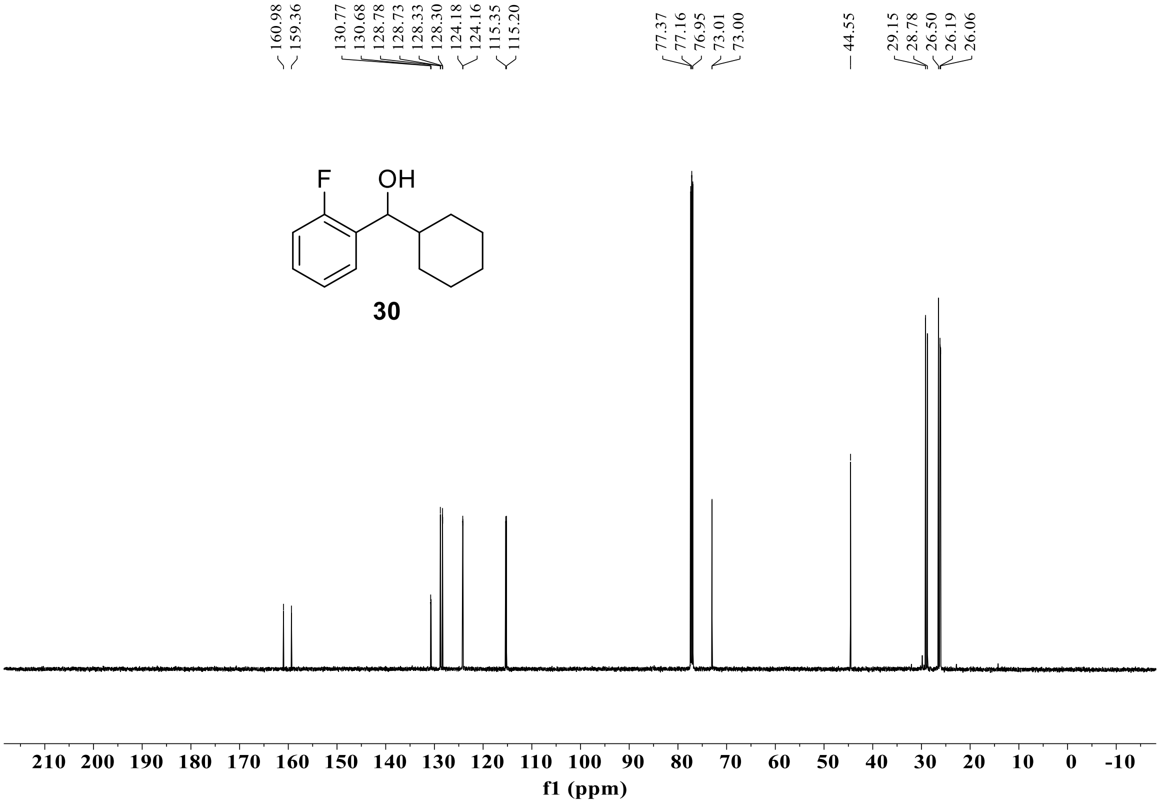
F
OH
30
f1 (ppm)

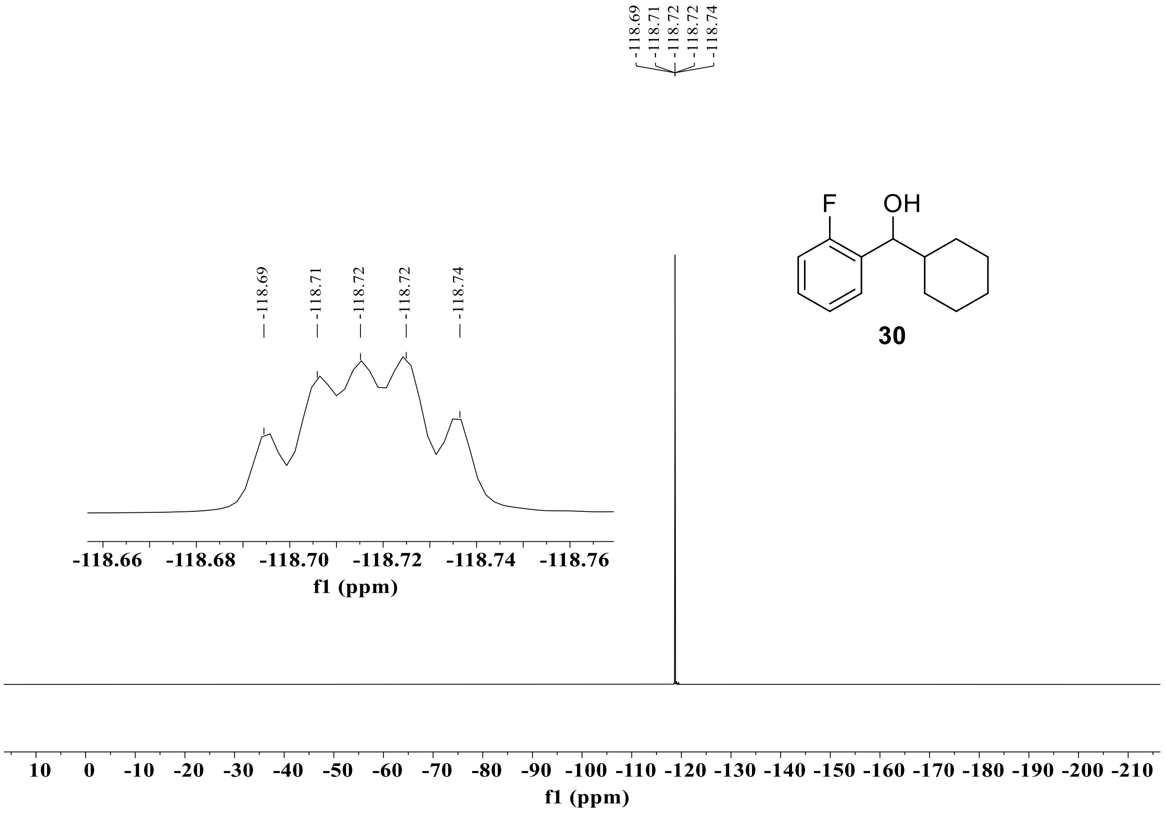

-118.69
-118.71
-118.72
-118.72
-118.74
-118.66 -118.68 -118.70 -118.72 -118.74 -118.76
f1 (ppm)
10 0 -10 -20 -30 -40 -50 -60 -70 -80 -90 -100 -110 -120 -130 -140 -150 -160 -170 -180 -190 -200 -210
f1 (ppm)
F
OH

30

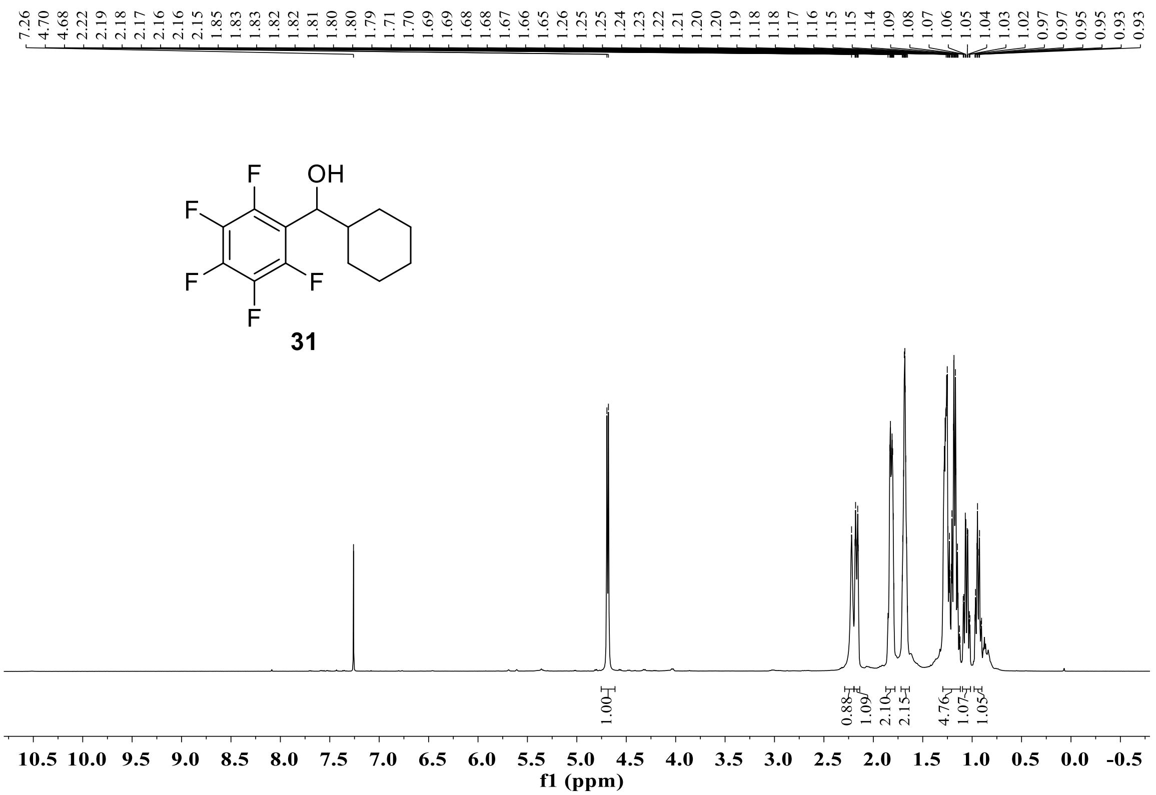
7.26 4.70 4.68 2.22 2.19 2.18 2.17 2.16 2.16 2.15 1.85 1.83 1.83 1.82 1.82 1.81 1.80 1.80 1.79 1.71 1.70 1.69 1.69 1.68 1.68 1.67 1.66 1.65 1.26 1.25 1.25 1.24 1.23 1.22 1.21 1.20 1.20 1.19 1.18 1.18 1.17 1.16 1.15 1.15 1.14 1.09 1.08 1.07 1.06 1.05 1.04 1.03 1.02 0.97 0.97 0.95 0.93 0.93
F
OH
F
F
F
F
31
1.00
0.88
1.09
2.10
2.15
4.76
1.07
1.05
10.5 10.0 9.5 9.0 8.5 8.0 7.5 7.0 6.5 6.0 5.5 5.0 4.5 4.0 3.5 3.0 2.5 2.0 1.5 1.0 0.5 0.0 -0.5
f1 (ppm)

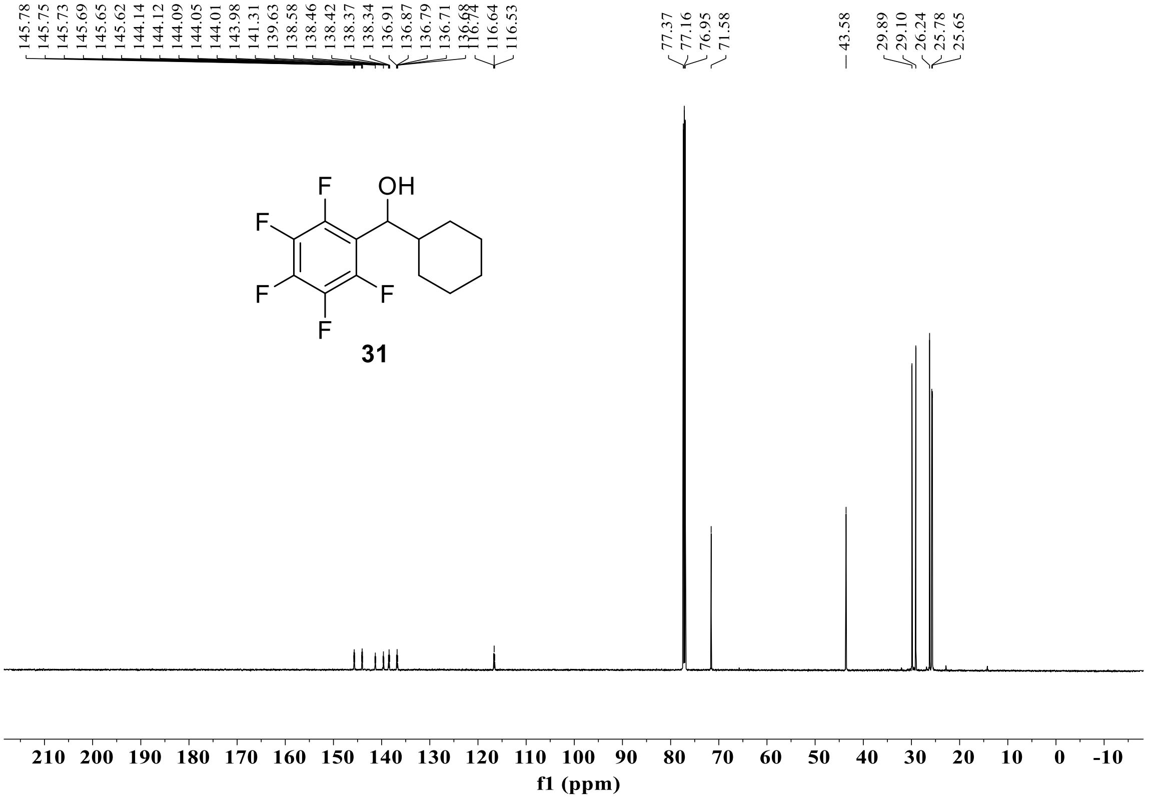
145.78 145.75 145.73 145.69 145.65 145.62 144.14 144.12 144.09 144.05 144.01 143.98 141.31 139.63 138.58 138.46 138.42 138.37 138.34 136.91 136.87 136.79 136.71 136.68 116.74 116.64 116.53
77.37 77.16 76.95 71.58
43.58
29.89 29.10 26.24 25.78 25.65
F
OH
F
F
F
F
31
210 200 190 180 170 160 150 140 130 120 110 100 90 80 70 60 50 40 30 20 10 0 -10
f1 (ppm)

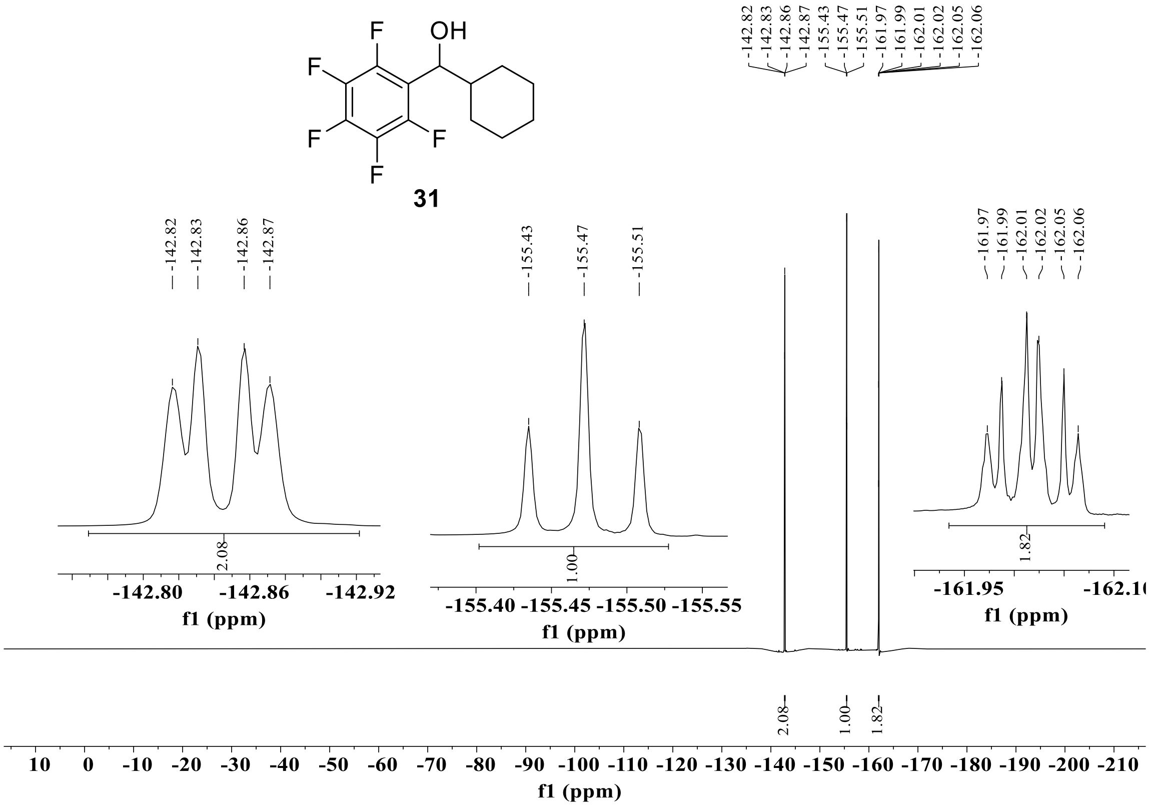

OH
F
F
F
F
F
31
-142.82
-142.83
-142.86
-142.87
-155.43
-155.47
-155.51
-161.97
-161.99
-162.01
-162.02
-162.05
-162.06
2.08
1.00
1.82
-142.80 -142.86 -142.92
-155.40 -155.45 -155.50 -155.55
-161.95
f1 (ppm)
10 0 -10 -20 -30 -40 -50 -60 -70 -80 -90 -100 -110 -120 -130 -140 -150 -160 -170 -180 -190 -200 -210

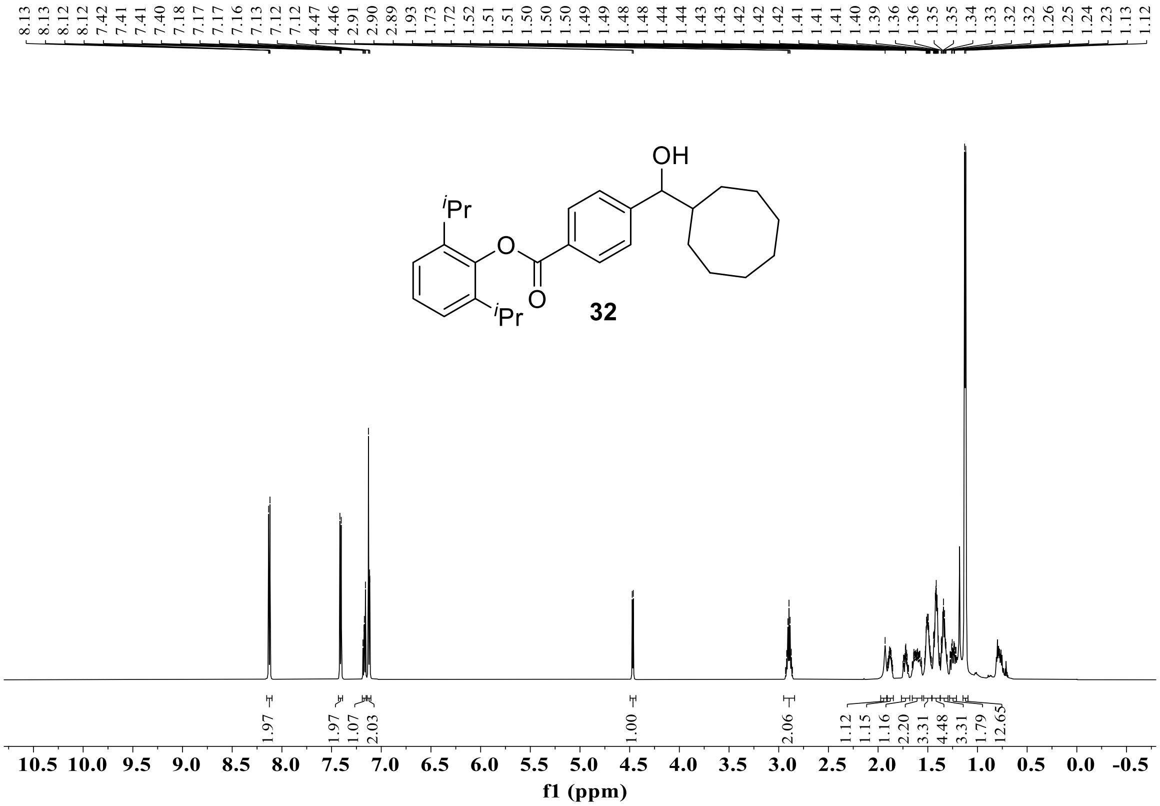
8.13 8.13 8.12 8.12 7.42 7.41 7.41 7.40 7.18 7.17 7.17 7.16 7.13 7.12 7.12 4.47 4.46 2.91 2.90 2.89 1.93 1.73 1.72 1.52 1.51 1.51 1.50 1.50 1.49 1.49 1.48 1.48 1.44 1.44 1.43 1.43 1.42 1.42 1.42 1.41 1.41 1.41 1.40 1.39 1.36 1.36 1.35 1.35 1.34 1.33 1.32 1.32 1.26 1.25 1.24 1.23 1.13 1.12
OH
iPr
O
O
iPr
32
1.97 1.97 1.07 2.03 1.00 2.06 1.12 1.15 1.16 2.20 3.31 4.48 3.31 1.79 12.65
10.5 10.0 9.5 9.0 8.5 8.0 7.5 7.0 6.5 6.0 5.5 5.0 4.5 4.0 3.5 3.0 2.5 2.0 1.5 1.0 0.5 0.0 -0.5
f1 (ppm)

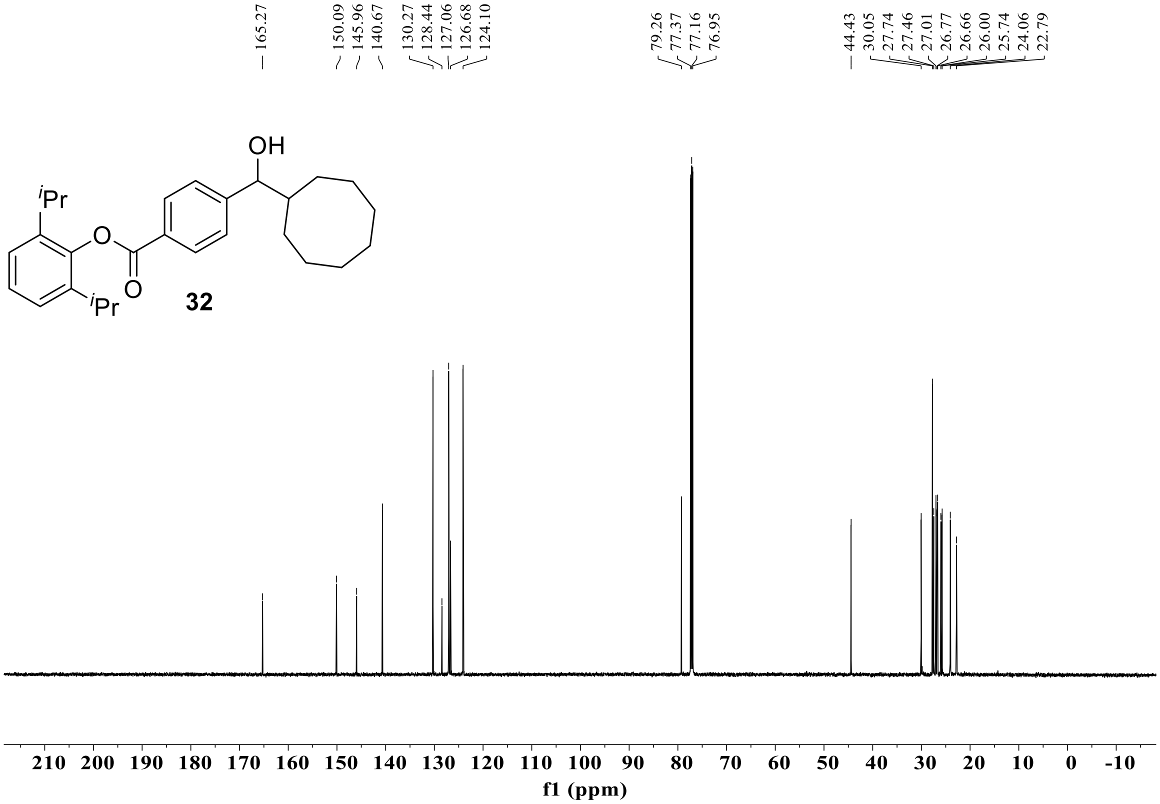
165.27 150.09 145.96 140.67 130.27 128.44 127.06 126.68 124.10 79.26 77.37 77.16 76.95 44.43 30.05 27.74 27.46 27.01 26.77 26.66 26.00 25.74 24.06 22.79
OH
iPr
O
O
iPr
32
210 200 190 180 170 160 150 140 130 120 110 100 90 80 70 60 50 40 30 20 10 0 -10
f1 (ppm)

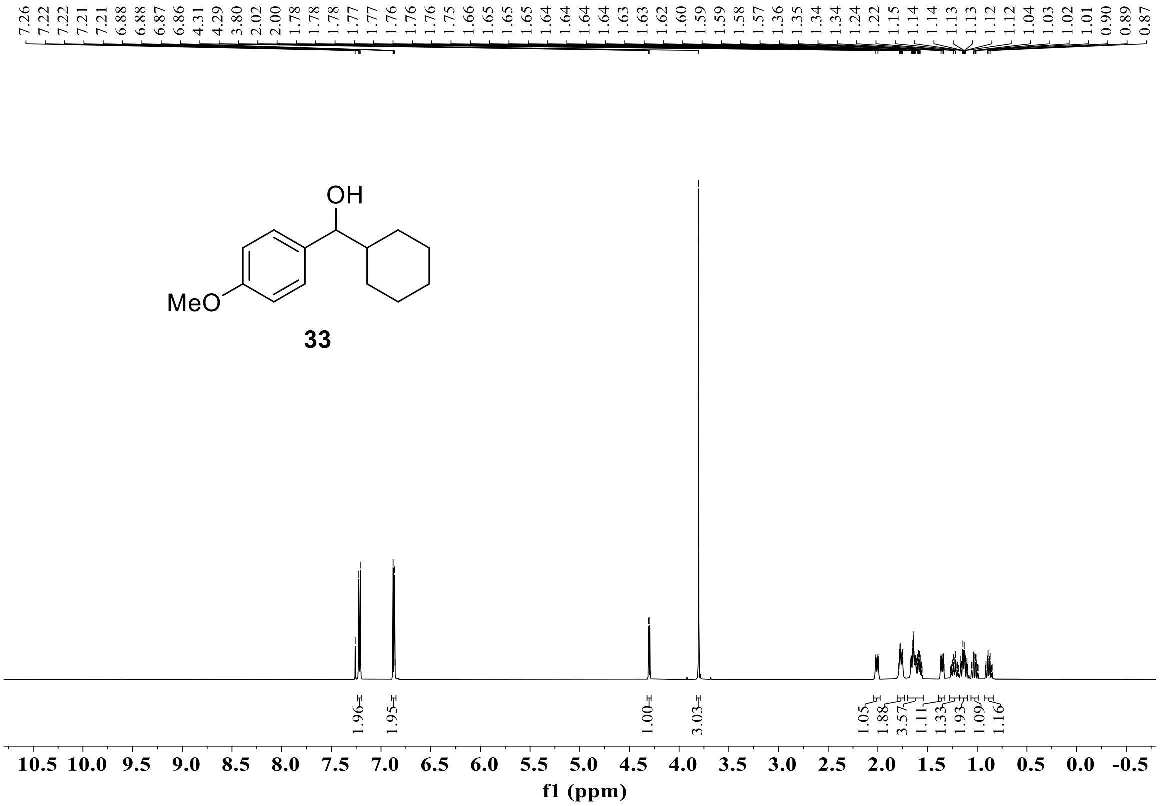

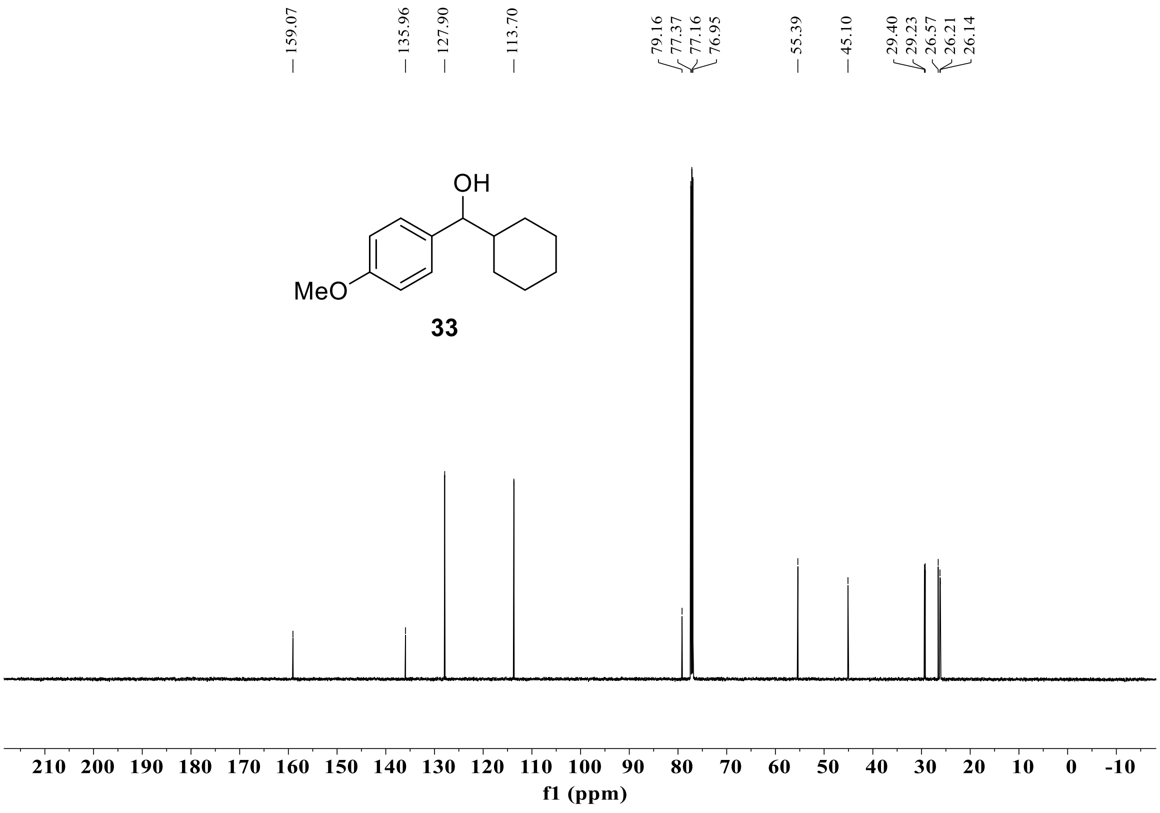

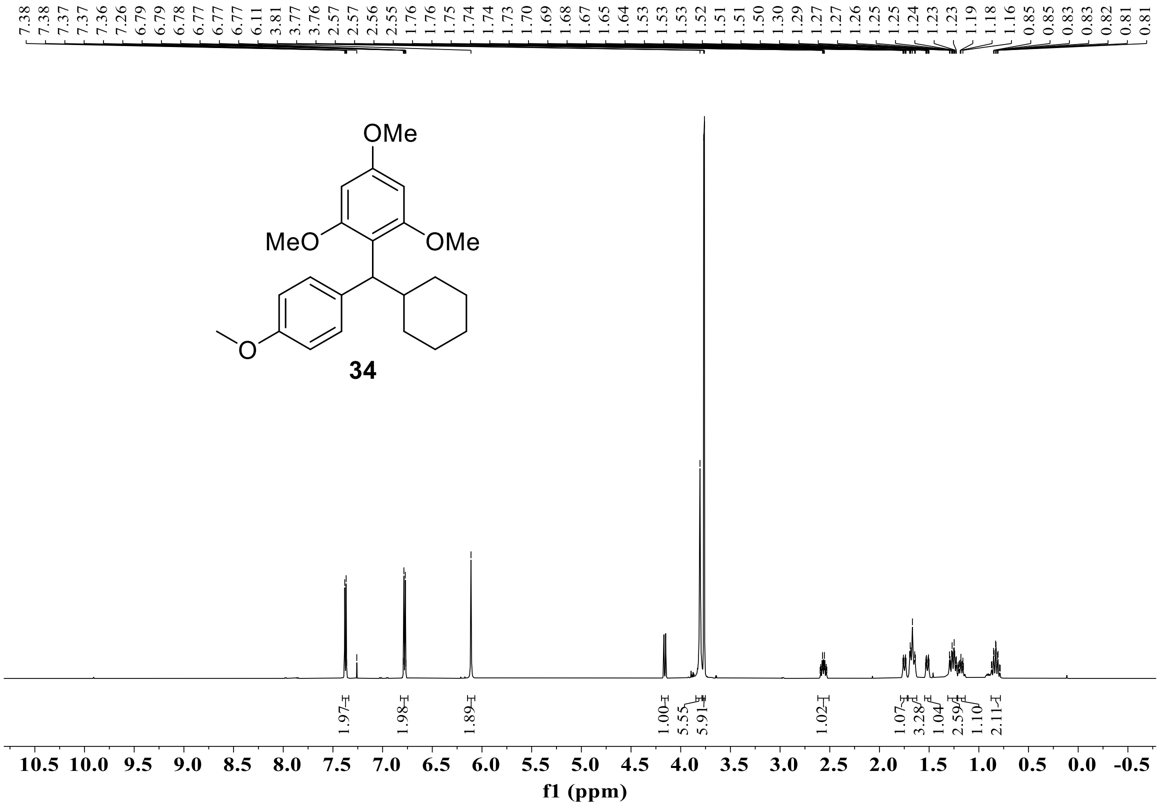
7.38 7.38 7.37 7.37 7.36 7.26 6.79 6.79 6.78 6.77 6.77 6.77 6.11 3.81 3.77 3.76 2.57 2.57 2.56 2.55 1.76 1.76 1.75 1.74 1.74 1.73 1.70 1.69 1.68 1.67 1.65 1.64 1.53 1.53 1.53 1.52 1.51 1.51 1.50 1.30 1.29 1.27 1.27 1.26 1.25 1.25 1.24 1.23 1.23 1.19 1.18 1.16 0.85 0.85 0.83 0.83 0.82 0.81 0.81
OMe
MeO
OMe
O
34
1.97 1.98 1.89 1.00 5.55 5.91 1.02 1.07 3.28 1.04 2.59 1.10 2.11
10.5 10.0 9.5 9.0 8.5 8.0 7.5 7.0 6.5 6.0 5.5 5.0 4.5 4.0 3.5 3.0 2.5 2.0 1.5 1.0 0.5 0.0 -0.5
f1 (ppm)

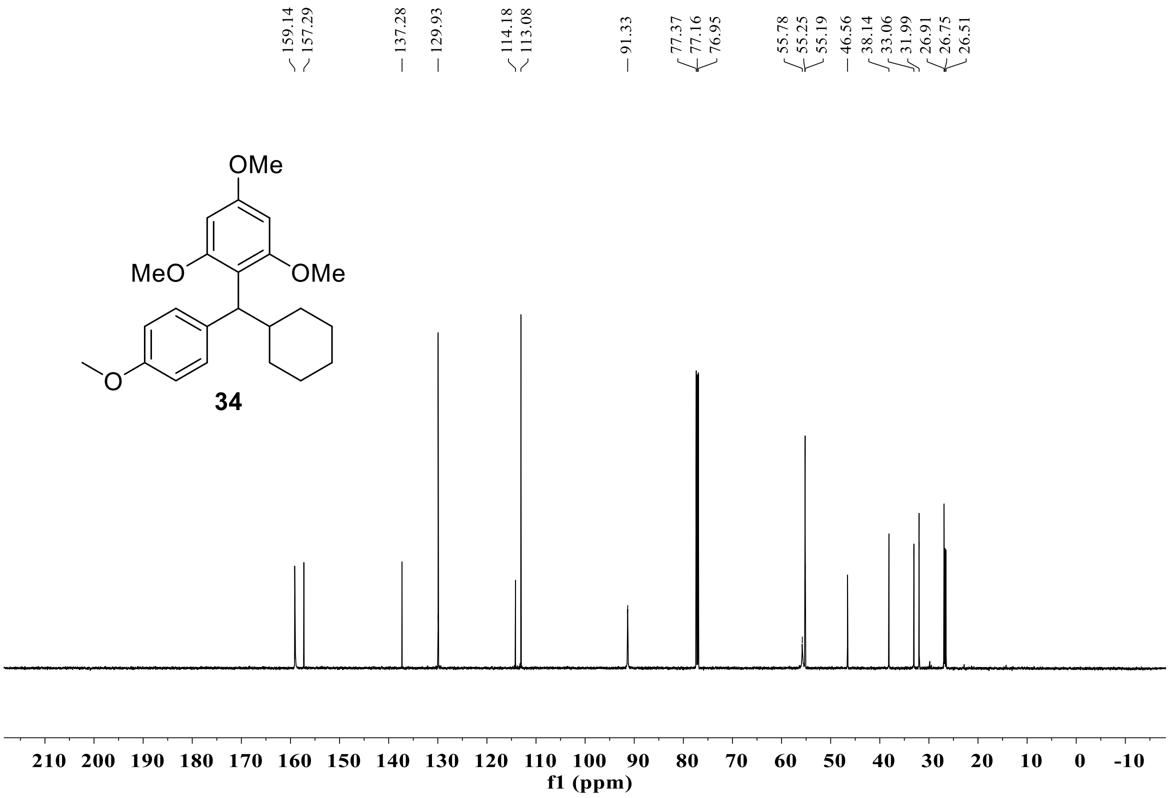
159.14 157.29 137.28 129.93 114.18 113.08 91.33 77.37 77.16 76.95 55.78 55.25 55.19 46.56 38.14 33.06 31.99 26.91 26.75 26.51
OMe
MeO
OMe
O
34
210 200 190 180 170 160 150 140 130 120 110 100 90 80 70 60 50 40 30 20 10 0 -10
f1 (ppm)

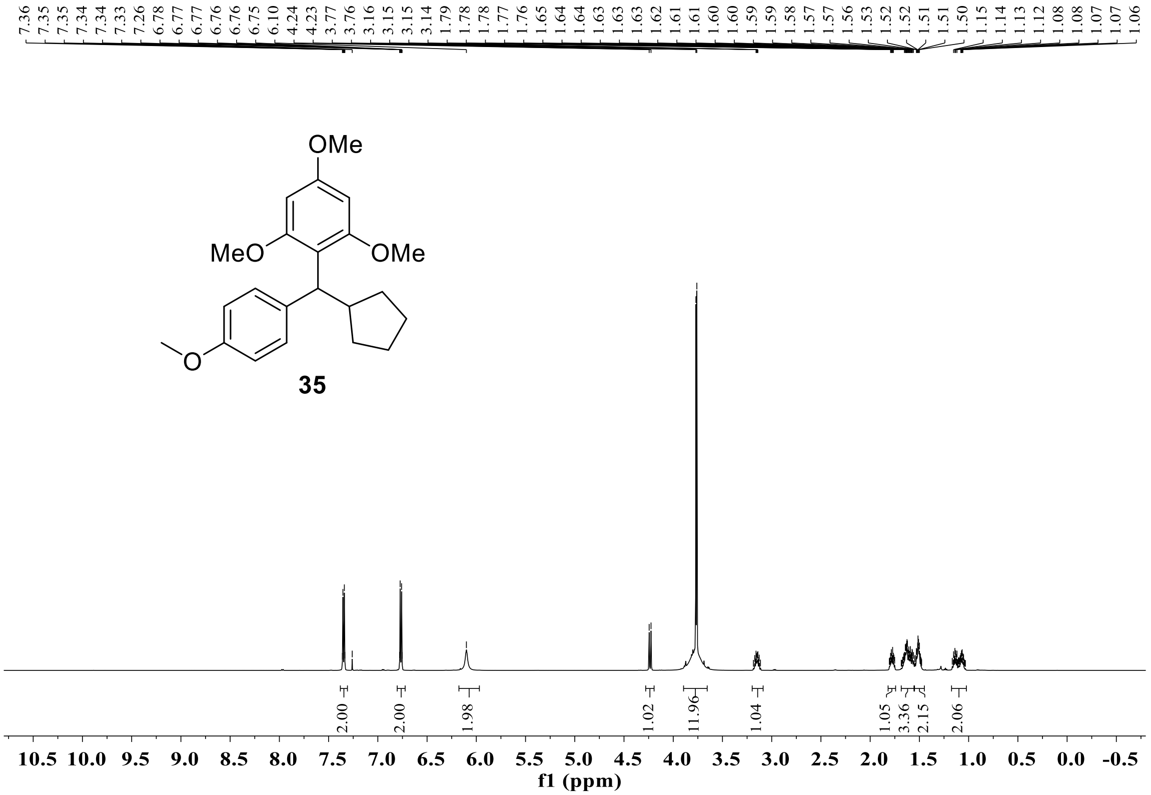
7.36 7.35 7.35 7.34 7.34 7.33 7.26 6.78 6.77 6.77 6.76 6.76 6.75 6.10 4.24 4.23 3.77 3.76 3.16 3.15 3.15 3.14 1.79 1.78 1.78 1.77 1.76 1.65 1.64 1.64 1.63 1.63 1.62 1.61 1.61 1.60 1.60 1.59 1.59 1.58 1.57 1.57 1.56 1.53 1.52 1.52 1.51 1.51 1.50 1.15 1.14 1.13 1.12 1.08 1.07 1.07 1.06
OMe
MeO
OMe
O
35
2.00
2.00
1.98
1.02
11.96
1.04
1.05
3.36
2.15
2.06
10.5 10.0 9.5 9.0 8.5 8.0 7.5 7.0 6.5 6.0 5.5 5.0 4.5 4.0 3.5 3.0 2.5 2.0 1.5 1.0 0.5 0.0 -0.5
f1 (ppm)

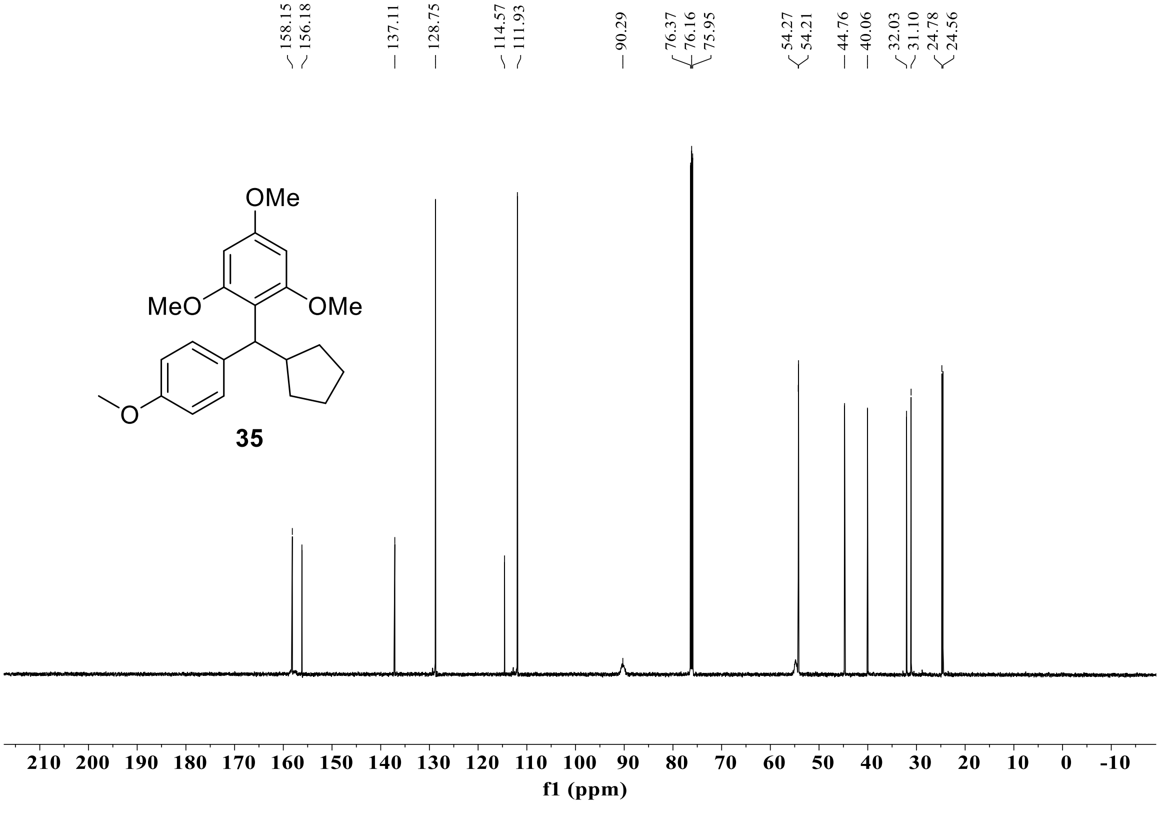
158.15 156.18 137.11 128.75 114.57 111.93 90.29 76.37 76.16 75.95 54.27 54.21 44.76 40.06 32.03 31.10 24.78 24.56
OMe
MeO
OMe
O
35
210 200 190 180 170 160 150 140 130 120 110 100 90 80 70 60 50 40 30 20 10 0 -10
f1 (ppm)

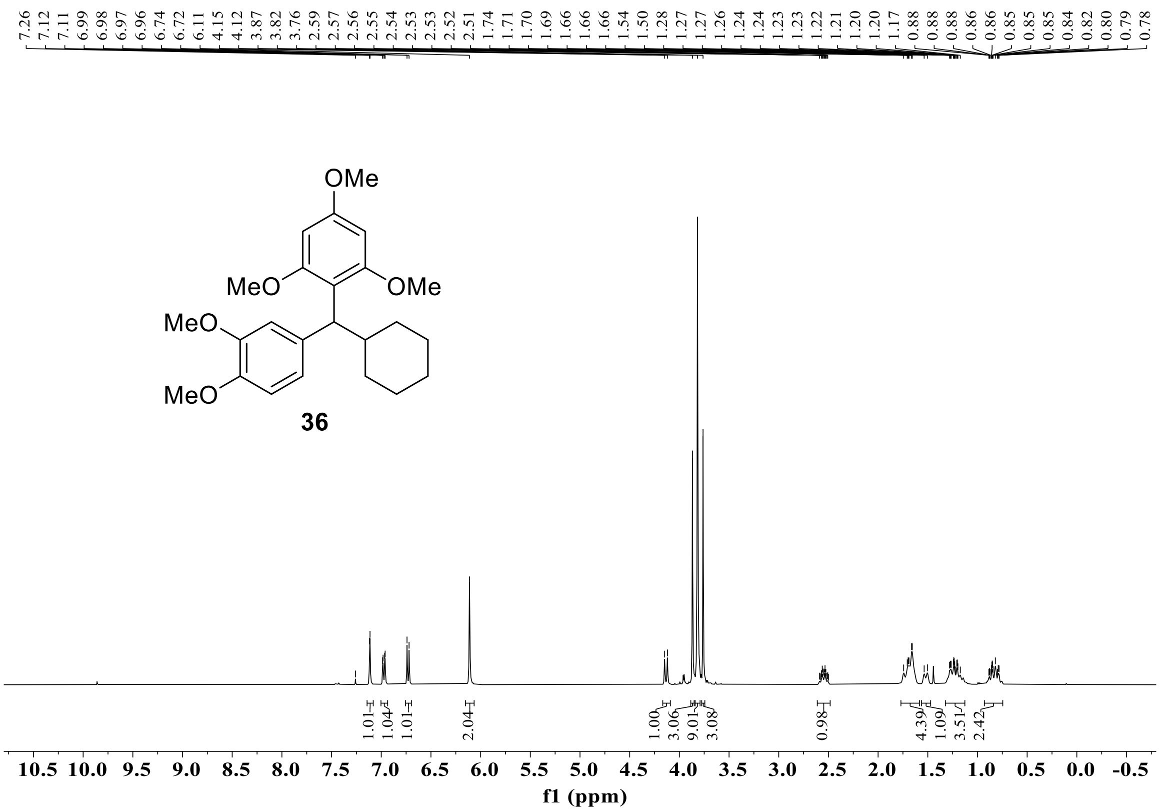
7.26 7.12 7.11 6.99 6.98 6.97 6.96 6.74 6.72 6.11 4.15 4.12 3.87 3.82 3.76 2.59 2.57 2.56 2.55 2.54 2.53 2.53 2.52 2.51 1.74 1.71 1.70 1.69 1.66 1.66 1.66 1.54 1.50 1.28 1.27 1.27 1.26 1.24 1.24 1.23 1.23 1.22 1.21 1.20 1.20 1.17 0.88 0.88 0.88 0.86 0.86 0.85 0.85 0.85 0.84 0.82 0.80 0.79 0.78
OMe
MeO
OMe
MeO
MeO
36
1.01 1.04 1.01 2.04 1.00 3.06 9.01 3.08 0.98 4.39 1.09 3.51 2.42
10.5 10.0 9.5 9.0 8.5 8.0 7.5 7.0 6.5 6.0 5.5 5.0 4.5 4.0 3.5 3.0 2.5 2.0 1.5 1.0 0.5 0.0 -0.5
f1 (ppm)

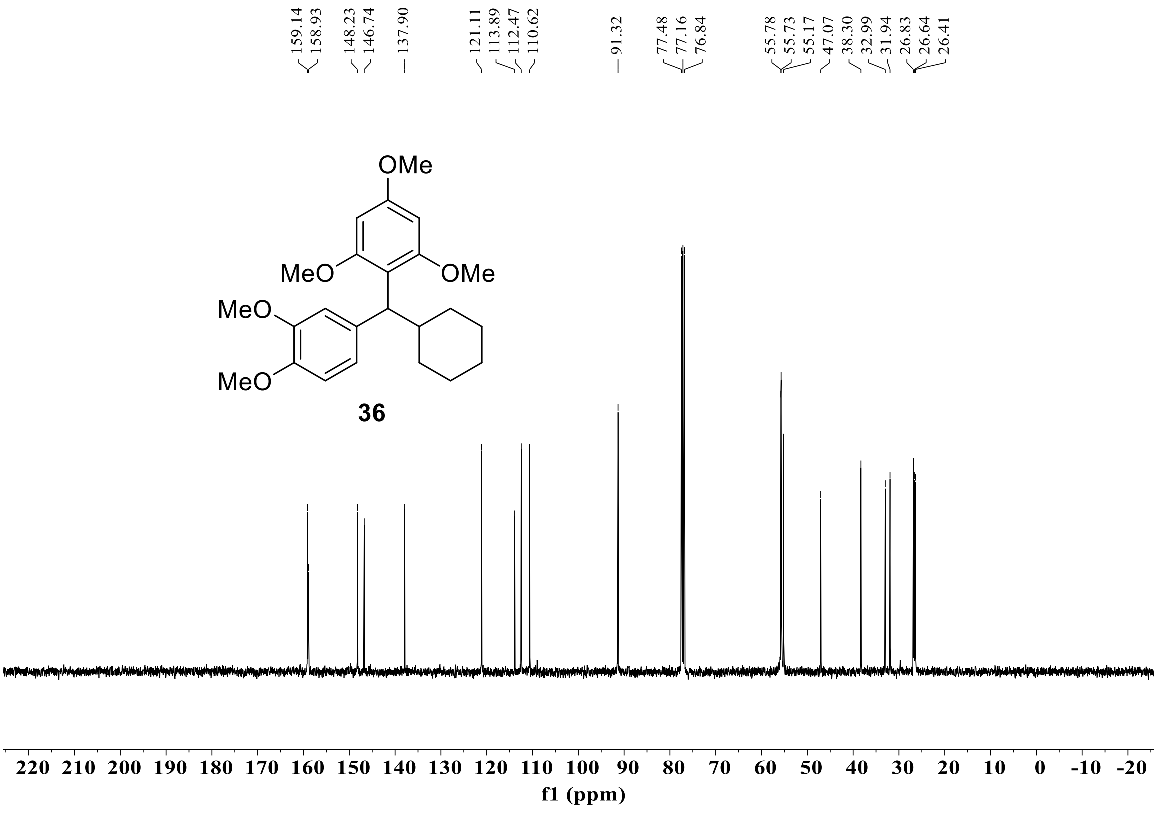
159.14 158.93 148.23 146.74 137.90 121.11 113.89 112.47 110.62 91.32 77.48 77.16 76.84 55.78 55.73 55.17 47.07 38.30 32.99 31.94 26.83 26.64 26.41
OMe
MeO
OMe
MeO
MeO
36
220 210 200 190 180 170 160 150 140 130 120 110 100 90 80 70 60 50 40 30 20 10 0 -10 -20
f1 (ppm)

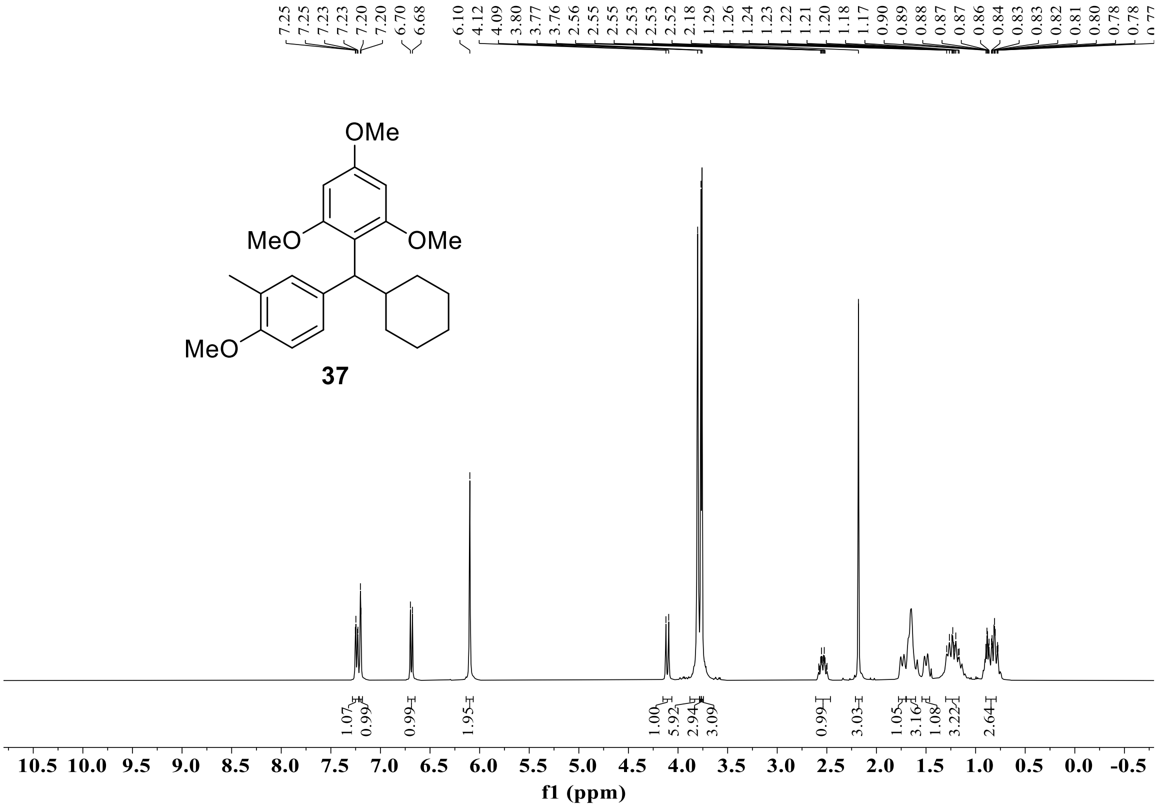

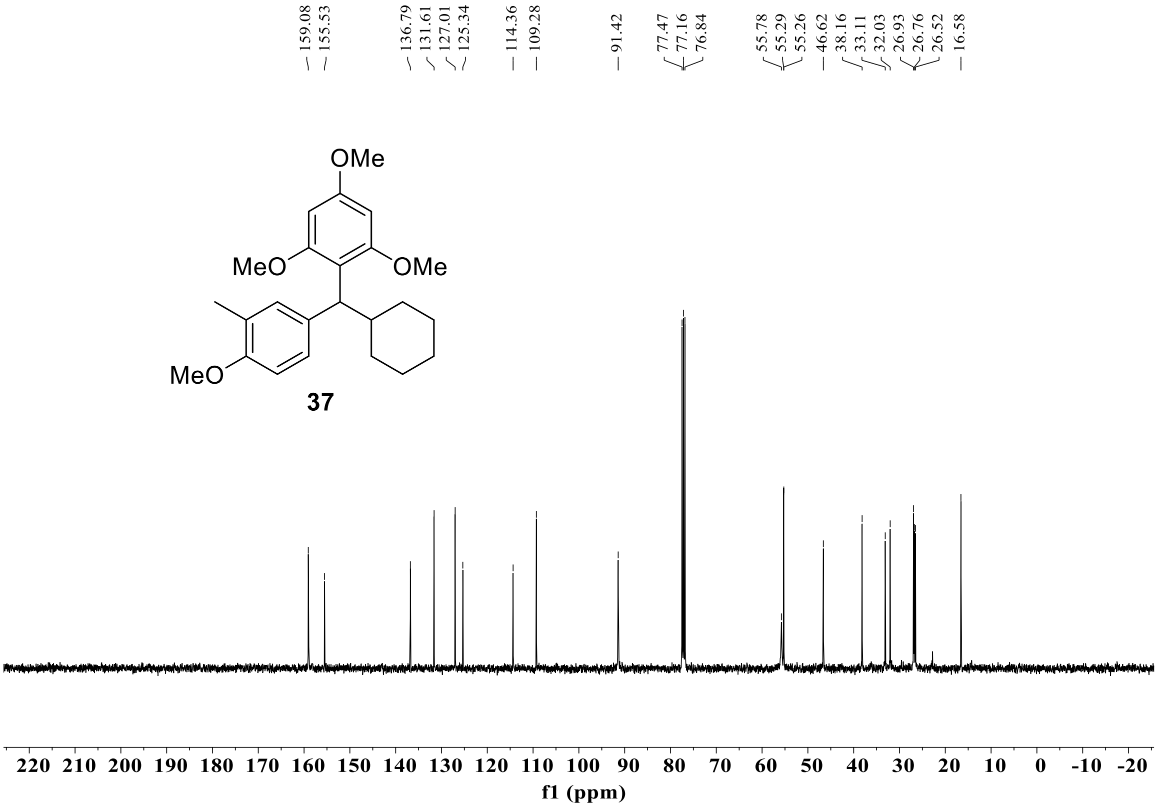

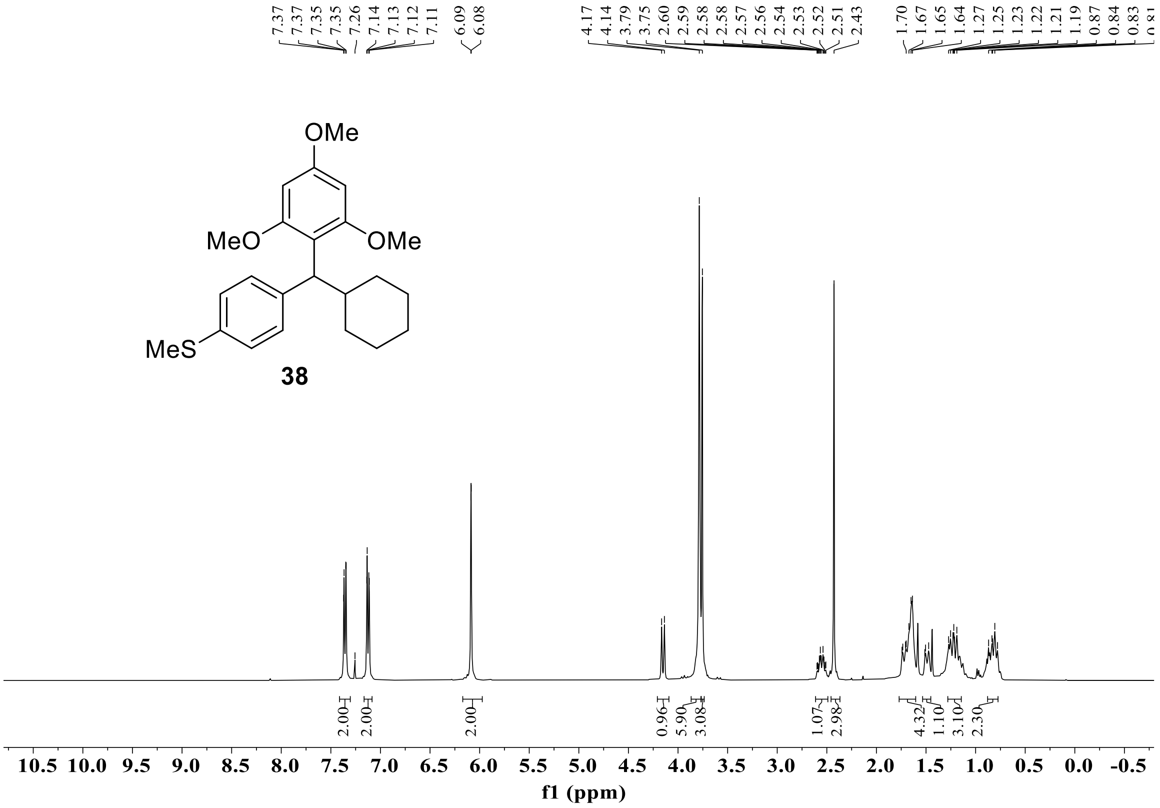
7.37
7.37
7.35
7.35
7.26
7.14
7.13
7.12
7.11
6.09
6.08
4.17
4.14
3.79
3.75
2.60
2.59
2.58
2.58
2.57
2.56
2.54
2.53
2.52
2.51
2.43
1.70
1.67
1.65
1.64
1.27
1.25
1.23
1.22
1.21
1.19
0.87
0.84
0.83
OMe
MeO
OMe
MeS
38
2.00
2.00
2.00
0.96
5.90
3.08
1.07
2.98
4.32
1.10
3.10
2.30
10.5 10.0 9.5 9.0 8.5 8.0 7.5 7.0 6.5 6.0 5.5 5.0 4.5 4.0 3.5 3.0 2.5 2.0 1.5 1.0 0.5 0.0 -0.5
f1 (ppm)

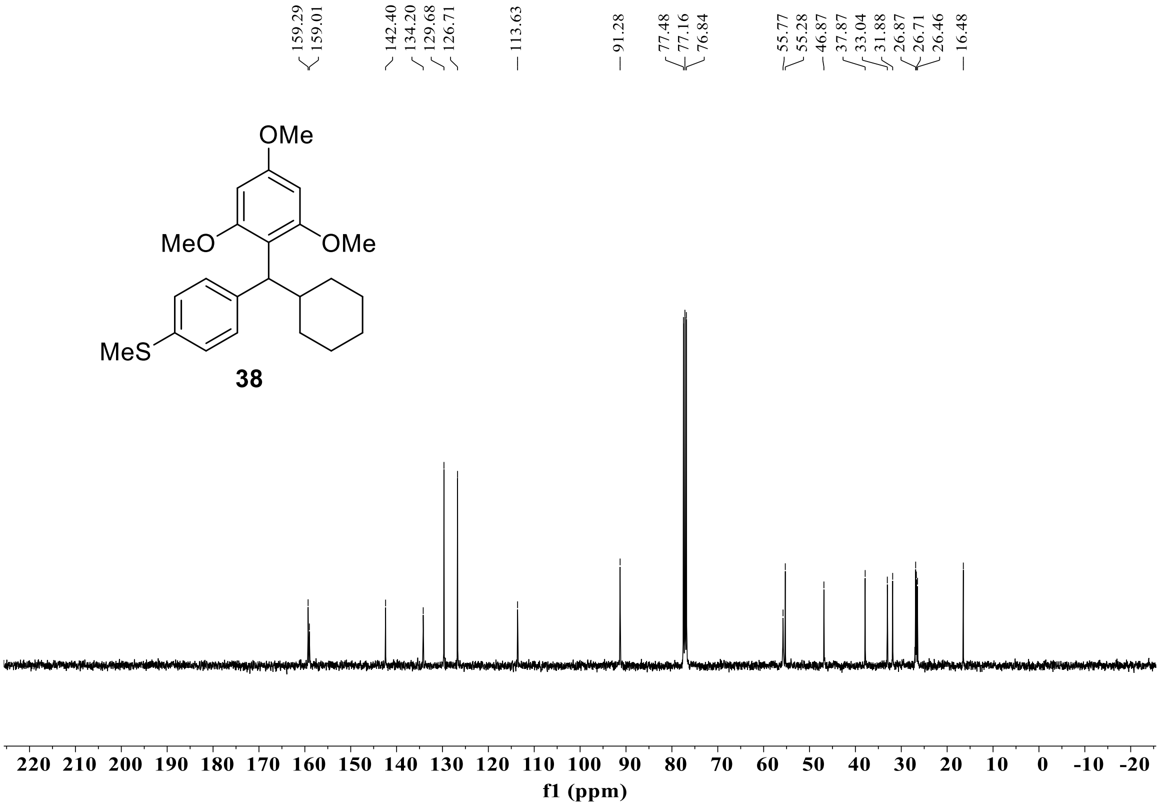
159.29
159.01
142.40
134.20
129.68
126.71
113.63
91.28
77.48
77.16
76.84
55.77
55.28
46.87
37.87
33.04
31.88
26.87
26.71
26.46
16.48
OMe
MeO
OMe
MeS
38
220 210 200 190 180 170 160 150 140 130 120 110 100 90 80 70 60 50 40 30 20 10 0 -10 -20
f1 (ppm)

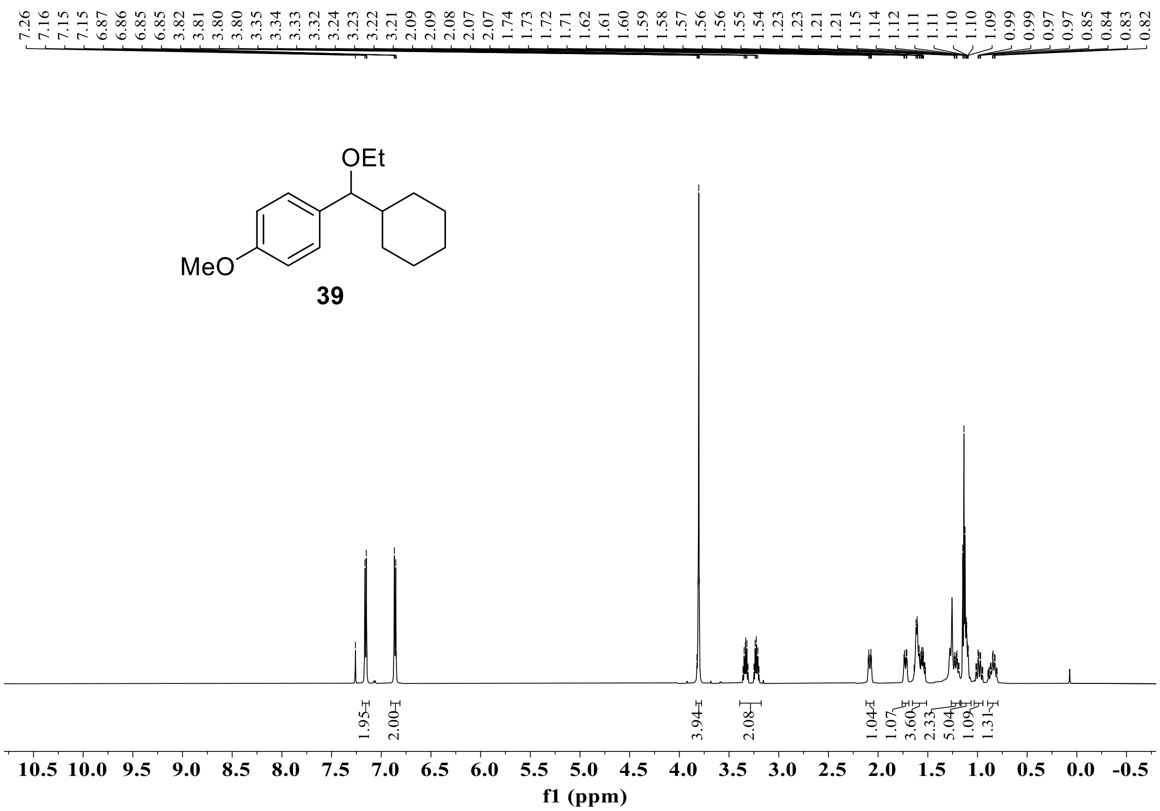
7.26
7.16
7.15
7.15
6.87
6.86
6.85
6.85
3.82
3.81
3.80
3.80
3.35
3.34
3.33
3.32
3.24
3.23
3.22
3.21
2.09
2.09
2.08
2.07
2.07
1.74
1.73
1.72
1.71
1.62
1.61
1.60
1.59
1.58
1.57
1.56
1.56
1.55
1.54
1.23
1.23
1.21
1.21
1.15
1.14
1.12
1.11
1.11
1.10
1.10
1.09
0.99
0.99
0.97
0.97
0.85
0.84
0.83
0.82
OEt
MeO
39
1.95
2.00
3.94
2.08
1.04
1.07
3.60
2.33
5.04
1.09
1.31
10.5 10.0 9.5 9.0 8.5 8.0 7.5 7.0 6.5 6.0 5.5 5.0 4.5 4.0 3.5 3.0 2.5 2.0 1.5 1.0 0.5 0.0 -0.5
f1 (ppm)

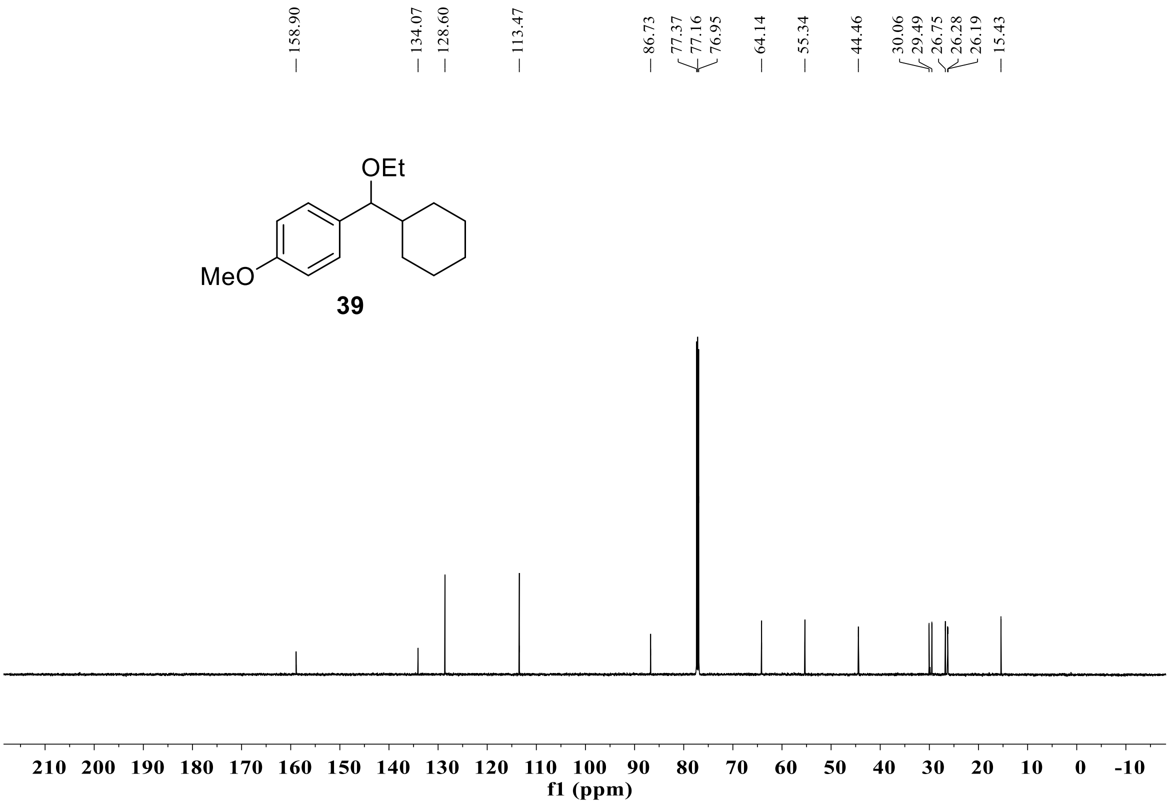
158.90
134.07
128.60
113.47
86.73
77.37
77.16
76.95
64.14
55.34
44.46
30.06
29.49
26.75
26.28
26.19
15.43
OEt
MeO
39
210 200 190 180 170 160 150 140 130 120 110 100 90 80 70 60 50 40 30 20 10 0 -10
f1 (ppm)
